# Supplementary material for: Deletion of wfs1 Impairs Oligodendrocyte Precursor Cells Dorsal Distribution and Myelination Through the wfs1-hmgcs1 Axis in Zebrafish
Source: Biology (Basel). 2026 Aug 16;15(16):1402. doi: 10.3390/biology15161402 (PMC13510151; doi:10.3390/biology15161402)
Supplement: Supplementary file 1 [file biology-15-01402-s001.zip › File S1-Transcriptome sequencing data and differentially expressed genes (DEGs) in wfs1-- zebrafish at 5 dpf/1.GO/wfs1KOvsWT/GSEA/WT/gsea_report_for_WT_1700447271245.html]

Report for WT 1700447271245 [GSEA]


| GS   follow link to MSigDB | GS DETAILS | SIZE | ES | NES | NOM p-val | FDR q-val | FWER p-val | RANK AT MAX | LEADING EDGE || 1 | CELL\_DIVISION\_SITE(GO:0032153) | Details ... | 48 | -0.49 | -2.30 | 0.000 | 0.061 | 0.012 | 1071 | tags=10%, list=3%, signal=11% |
| 2 | INTRAMOLECULAR\_OXIDOREDUCTASE\_ACTIVITY(GO:0016860) | Details ... | 33 | -0.55 | -2.18 | 0.000 | 0.086 | 0.060 | 844 | tags=15%, list=3%, signal=16% |
| 3 | REGULATION\_OF\_CELL\_SHAPE(GO:0008360) | Details ... | 80 | -0.44 | -2.15 | 0.000 | 0.061 | 0.060 | 5313 | tags=26%, list=17%, signal=31% |
| 4 | CLEAVAGE\_FURROW(GO:0032154) | Details ... | 27 | -0.53 | -2.08 | 0.000 | 0.072 | 0.067 | 1071 | tags=15%, list=3%, signal=15% |
| 5 | NUCLEAR\_HORMONE\_RECEPTOR\_BINDING(GO:0035257) | Details ... | 19 | -0.65 | -2.00 | 0.000 | 0.136 | 0.183 | 947 | tags=16%, list=3%, signal=16% |
| 6 | EPITHELIAL\_STRUCTURE\_MAINTENANCE(GO:0010669) | Details ... | 16 | -0.72 | -1.96 | 0.000 | 0.164 | 0.261 | 1567 | tags=25%, list=5%, signal=26% |
| 7 | REGULATION\_OF\_DNA\_REPLICATION(GO:0006275) | Details ... | 26 | -0.55 | -1.96 | 0.000 | 0.145 | 0.261 | 5151 | tags=23%, list=16%, signal=27% |
| 8 | CELL\_DIVISION\_SITE\_PART(GO:0032155) | Details ... | 36 | -0.47 | -1.95 | 0.000 | 0.137 | 0.261 | 1071 | tags=11%, list=3%, signal=11% |
| 9 | MIDBODY(GO:0030496) | Details ... | 39 | -0.50 | -1.92 | 0.000 | 0.182 | 0.352 | 1071 | tags=10%, list=3%, signal=11% |
| 10 | SEPTIN\_COMPLEX(GO:0031105) | Details ... | 17 | -0.62 | -1.89 | 0.000 | 0.240 | 0.406 | 537 | tags=12%, list=2%, signal=12% |
| 11 | SEPTIN\_CYTOSKELETON(GO:0032156) | Details ... | 17 | -0.62 | -1.89 | 0.000 | 0.218 | 0.406 | 537 | tags=12%, list=2%, signal=12% |
| 12 | NEGATIVE\_REGULATION\_OF\_IMMUNE\_RESPONSE(GO:0050777) | Details ... | 19 | -0.55 | -1.87 | 0.000 | 0.226 | 0.497 | 1287 | tags=26%, list=4%, signal=27% |
| 13 | SEMAPHORIN\_RECEPTOR\_ACTIVITY(GO:0017154) | Details ... | 19 | -0.62 | -1.86 | 0.000 | 0.236 | 0.538 | 2283 | tags=21%, list=7%, signal=23% |
| 14 | SEPTIN\_RING(GO:0005940) | Details ... | 16 | -0.65 | -1.84 | 0.000 | 0.257 | 0.564 | 537 | tags=13%, list=2%, signal=13% |
| 15 | TISSUE\_HOMEOSTASIS(GO:0001894) | Details ... | 62 | -0.46 | -1.84 | 0.000 | 0.245 | 0.564 | 1567 | tags=11%, list=5%, signal=12% |
| 16 | SCF\_DEPENDENT\_PROTEASOMAL\_UBIQUITIN\_DEPENDENT\_PROTEIN\_CATABOLIC\_PROCESS(GO:0031146) | Details ... | 46 | -0.52 | -1.84 | 0.000 | 0.248 | 0.588 | 4466 | tags=33%, list=14%, signal=38% |
| 17 | ASPARTATE\_FAMILY\_AMINO\_ACID\_METABOLIC\_PROCESS(GO:0009066) | Details ... | 35 | -0.47 | -1.83 | 0.000 | 0.240 | 0.588 | 4660 | tags=29%, list=15%, signal=33% |
| 18 | CELL\_CORTEX\_PART(GO:0044448) | Details ... | 89 | -0.41 | -1.83 | 0.000 | 0.227 | 0.588 | 1670 | tags=10%, list=5%, signal=11% |
| 19 | AUTOPHAGOSOME\_MATURATION(GO:0097352) | Details ... | 24 | -0.55 | -1.82 | 0.000 | 0.236 | 0.607 | 4872 | tags=21%, list=15%, signal=25% |
| 20 | UBIQUITIN\_LIKE\_PROTEIN\_BINDING(GO:0032182) | Details ... | 53 | -0.41 | -1.81 | 0.000 | 0.255 | 0.659 | 2891 | tags=15%, list=9%, signal=17% |
| 21 | METHIONINE\_METABOLIC\_PROCESS(GO:0006555) | Details ... | 15 | -0.48 | -1.81 | 0.000 | 0.252 | 0.659 | 3859 | tags=27%, list=12%, signal=30% |
| 22 | PURINE\_RIBONUCLEOSIDE\_METABOLIC\_PROCESS(GO:0046128) | Details ... | 45 | -0.47 | -1.79 | 0.000 | 0.277 | 0.689 | 7271 | tags=38%, list=23%, signal=49% |
| 23 | CELL\_CORTEX(GO:0005938) | Details ... | 163 | -0.36 | -1.79 | 0.000 | 0.283 | 0.707 | 7119 | tags=23%, list=22%, signal=30% |
| 24 | ESTABLISHMENT\_OF\_CELL\_POLARITY(GO:0030010) | Details ... | 46 | -0.40 | -1.79 | 0.000 | 0.287 | 0.707 | 1743 | tags=9%, list=5%, signal=9% |
| 25 | UBIQUITIN\_BINDING(GO:0043130) | Details ... | 44 | -0.45 | -1.78 | 0.000 | 0.299 | 0.717 | 2891 | tags=16%, list=9%, signal=17% |
| 26 | REGULATION\_OF\_DNA\_REPAIR(GO:0006282) | Details ... | 45 | -0.39 | -1.77 | 0.000 | 0.309 | 0.717 | 2574 | tags=13%, list=8%, signal=14% |
| 27 | CARDIAC\_MUSCLE\_CELL\_DEVELOPMENT(GO:0055013) | Details ... | 39 | -0.51 | -1.76 | 0.000 | 0.332 | 0.729 | 6492 | tags=38%, list=20%, signal=48% |
| 28 | NEGATIVE\_REGULATION\_OF\_CELL\_CYCLE\_PROCESS(GO:0010948) | Details ... | 66 | -0.38 | -1.75 | 0.031 | 0.359 | 0.782 | 1574 | tags=11%, list=5%, signal=11% |
| 29 | MACROAUTOPHAGY(GO:0016236) | Details ... | 103 | -0.39 | -1.74 | 0.000 | 0.367 | 0.782 | 5326 | tags=21%, list=17%, signal=26% |
| 30 | ASPARTATE\_FAMILY\_AMINO\_ACID\_BIOSYNTHETIC\_PROCESS(GO:0009067) | Details ... | 15 | -0.61 | -1.73 | 0.032 | 0.366 | 0.782 | 3859 | tags=40%, list=12%, signal=45% |
| 31 | UBIQUITIN\_DEPENDENT\_ERAD\_PATHWAY(GO:0030433) | Details ... | 60 | -0.42 | -1.73 | 0.000 | 0.355 | 0.782 | 4466 | tags=22%, list=14%, signal=25% |
| 32 | NEUROMUSCULAR\_PROCESS(GO:0050905) | Details ... | 19 | -0.56 | -1.73 | 0.000 | 0.350 | 0.811 | 1842 | tags=26%, list=6%, signal=28% |
| 33 | HISTONE\_ACETYLTRANSFERASE\_COMPLEX(GO:0000123) | Details ... | 56 | -0.39 | -1.73 | 0.016 | 0.345 | 0.811 | 779 | tags=5%, list=2%, signal=5% |
| 34 | TRANSCRIPTIONAL\_ACTIVATOR\_ACTIVITY\_\_RNA\_POLYMERASE\_II\_TRANSCRIPTION\_REGULATORY\_REGION\_SEQUENCE\_SPECIFIC\_DNA\_BINDING(GO:0001228) | Details ... | 78 | -0.41 | -1.73 | 0.000 | 0.337 | 0.827 | 3861 | tags=19%, list=12%, signal=22% |
| 35 | CELLULAR\_RESPONSE\_TO\_OXYGEN\_CONTAINING\_COMPOUND(GO:1901701) | Details ... | 190 | -0.35 | -1.73 | 0.000 | 0.332 | 0.827 | 4643 | tags=20%, list=15%, signal=23% |
| 36 | SITE\_OF\_DNA\_DAMAGE(GO:0090734) | Details ... | 24 | -0.44 | -1.73 | 0.035 | 0.336 | 0.827 | 1149 | tags=13%, list=4%, signal=13% |
| 37 | SAGA\_TYPE\_COMPLEX(GO:0070461) | Details ... | 16 | -0.45 | -1.72 | 0.013 | 0.329 | 0.827 | 2697 | tags=13%, list=8%, signal=14% |
| 38 | CELLULAR\_RESPONSE\_TO\_EXTRACELLULAR\_STIMULUS(GO:0031668) | Details ... | 66 | -0.48 | -1.72 | 0.000 | 0.323 | 0.842 | 5141 | tags=27%, list=16%, signal=32% |
| 39 | PURINE\_NUCLEOSIDE\_METABOLIC\_PROCESS(GO:0042278) | Details ... | 47 | -0.43 | -1.72 | 0.000 | 0.321 | 0.842 | 7271 | tags=36%, list=23%, signal=47% |
| 40 | CELLULAR\_RESPONSE\_TO\_EXTERNAL\_STIMULUS(GO:0071496) | Details ... | 68 | -0.48 | -1.72 | 0.000 | 0.315 | 0.842 | 5141 | tags=26%, list=16%, signal=31% |
| 41 | 3'\_5'\_EXORIBONUCLEASE\_ACTIVITY(GO:0000175) | Details ... | 23 | -0.59 | -1.70 | 0.000 | 0.350 | 0.890 | 2664 | tags=22%, list=8%, signal=24% |
| 42 | EXORIBONUCLEASE\_ACTIVITY(GO:0004532) | Details ... | 23 | -0.59 | -1.70 | 0.000 | 0.342 | 0.890 | 2664 | tags=22%, list=8%, signal=24% |
| 43 | EXORIBONUCLEASE\_ACTIVITY\_\_PRODUCING\_5'\_PHOSPHOMONOESTERS(GO:0016896) | Details ... | 23 | -0.59 | -1.70 | 0.000 | 0.334 | 0.890 | 2664 | tags=22%, list=8%, signal=24% |
| 44 | EXTRACELLULAR\_MATRIX\_ASSEMBLY(GO:0085029) | Details ... | 35 | -0.50 | -1.70 | 0.000 | 0.328 | 0.890 | 2546 | tags=20%, list=8%, signal=22% |
| 45 | CELLULAR\_RESPONSE\_TO\_NUTRIENT\_LEVELS(GO:0031669) | Details ... | 64 | -0.47 | -1.70 | 0.000 | 0.335 | 0.909 | 5141 | tags=25%, list=16%, signal=30% |
| 46 | STRIATED\_MUSCLE\_CELL\_PROLIFERATION(GO:0014855) | Details ... | 19 | -0.59 | -1.70 | 0.000 | 0.332 | 0.909 | 4290 | tags=37%, list=13%, signal=43% |
| 47 | MUSCLE\_CELL\_PROLIFERATION(GO:0033002) | Details ... | 25 | -0.49 | -1.70 | 0.000 | 0.329 | 0.927 | 4290 | tags=28%, list=13%, signal=32% |
| 48 | REGULATION\_OF\_FATTY\_ACID\_METABOLIC\_PROCESS(GO:0019217) | Details ... | 17 | -0.55 | -1.70 | 0.019 | 0.325 | 0.927 | 7779 | tags=41%, list=24%, signal=54% |
| 49 | HABENULA\_DEVELOPMENT(GO:0021986) | Details ... | 19 | -0.53 | -1.70 | 0.000 | 0.320 | 0.927 | 4864 | tags=21%, list=15%, signal=25% |
| 50 | PEROXISOMAL\_MEMBRANE(GO:0005778) | Details ... | 30 | -0.47 | -1.70 | 0.000 | 0.315 | 0.927 | 2519 | tags=17%, list=8%, signal=18% |
| 51 | MICROBODY\_MEMBRANE(GO:0031903) |  | 30 | -0.47 | -1.70 | 0.000 | 0.309 | 0.927 | 2519 | tags=17%, list=8%, signal=18% |
| 52 | CELLULAR\_RESPONSE\_TO\_STARVATION(GO:0009267) |  | 57 | -0.46 | -1.70 | 0.000 | 0.304 | 0.927 | 5141 | tags=25%, list=16%, signal=29% |
| 53 | FUCOSYLATION(GO:0036065) |  | 18 | -0.60 | -1.69 | 0.000 | 0.301 | 0.927 | 2096 | tags=28%, list=7%, signal=30% |
| 54 | GRANULOCYTE\_DIFFERENTIATION(GO:0030851) |  | 27 | -0.41 | -1.69 | 0.000 | 0.302 | 0.927 | 527 | tags=7%, list=2%, signal=8% |
| 55 | FAT\_SOLUBLE\_VITAMIN\_METABOLIC\_PROCESS(GO:0006775) |  | 15 | -0.53 | -1.69 | 0.000 | 0.299 | 0.927 | 3696 | tags=27%, list=12%, signal=30% |
| 56 | TRANSFERASE\_ACTIVITY\_\_TRANSFERRING\_NITROGENOUS\_GROUPS(GO:0016769) |  | 26 | -0.45 | -1.69 | 0.024 | 0.299 | 0.938 | 1665 | tags=12%, list=5%, signal=12% |
| 57 | PROTEIN\_ADP\_RIBOSYLATION(GO:0006471) |  | 19 | -0.60 | -1.69 | 0.000 | 0.293 | 0.938 | 4387 | tags=37%, list=14%, signal=43% |
| 58 | SNORNA\_BINDING(GO:0030515) |  | 18 | -0.60 | -1.69 | 0.000 | 0.294 | 0.938 | 9846 | tags=67%, list=31%, signal=96% |
| 59 | CELLULAR\_TRANSITION\_METAL\_ION\_HOMEOSTASIS(GO:0046916) |  | 46 | -0.37 | -1.68 | 0.000 | 0.296 | 0.938 | 876 | tags=9%, list=3%, signal=9% |
| 60 | NUCLEOLAR\_PART(GO:0044452) |  | 46 | -0.49 | -1.68 | 0.000 | 0.303 | 0.938 | 9608 | tags=37%, list=30%, signal=53% |
| 61 | CONVERGENT\_EXTENSION(GO:0060026) |  | 151 | -0.30 | -1.68 | 0.000 | 0.298 | 0.938 | 4959 | tags=14%, list=16%, signal=16% |
| 62 | SCF\_UBIQUITIN\_LIGASE\_COMPLEX(GO:0019005) |  | 57 | -0.43 | -1.68 | 0.000 | 0.295 | 0.938 | 4466 | tags=28%, list=14%, signal=33% |
| 63 | MATURATION\_OF\_SSU\_RRNA(GO:0030490) |  | 41 | -0.52 | -1.68 | 0.029 | 0.294 | 0.938 | 9140 | tags=46%, list=29%, signal=65% |
| 64 | MYOBLAST\_DIFFERENTIATION(GO:0045445) |  | 16 | -0.59 | -1.68 | 0.049 | 0.290 | 0.938 | 446 | tags=13%, list=1%, signal=13% |
| 65 | PROTEIN\_DEACETYLASE\_ACTIVITY(GO:0033558) |  | 17 | -0.64 | -1.68 | 0.000 | 0.288 | 0.938 | 2931 | tags=29%, list=9%, signal=32% |
| 66 | PCG\_PROTEIN\_COMPLEX(GO:0031519) |  | 23 | -0.45 | -1.67 | 0.000 | 0.291 | 0.938 | 123 | tags=4%, list=0%, signal=4% |
| 67 | DEACETYLASE\_ACTIVITY(GO:0019213) |  | 22 | -0.61 | -1.67 | 0.000 | 0.289 | 0.938 | 2931 | tags=27%, list=9%, signal=30% |
| 68 | CYTOPLASMIC\_REGION(GO:0099568) |  | 221 | -0.31 | -1.67 | 0.000 | 0.293 | 0.938 | 6116 | tags=20%, list=19%, signal=25% |
| 69 | TRANSAMINASE\_ACTIVITY(GO:0008483) |  | 24 | -0.46 | -1.67 | 0.024 | 0.289 | 0.938 | 1665 | tags=13%, list=5%, signal=13% |
| 70 | CARDIAC\_MUSCLE\_CELL\_DIFFERENTIATION(GO:0055007) |  | 53 | -0.45 | -1.67 | 0.000 | 0.288 | 0.938 | 6529 | tags=34%, list=20%, signal=43% |
| 71 | ERAD\_PATHWAY(GO:0036503) |  | 65 | -0.40 | -1.66 | 0.000 | 0.294 | 0.938 | 4466 | tags=20%, list=14%, signal=23% |
| 72 | PROTEIN\_ACETYLTRANSFERASE\_COMPLEX(GO:0031248) |  | 65 | -0.36 | -1.66 | 0.043 | 0.290 | 0.938 | 779 | tags=5%, list=2%, signal=5% |
| 73 | ACETYLTRANSFERASE\_COMPLEX(GO:1902493) |  | 65 | -0.36 | -1.66 | 0.043 | 0.286 | 0.938 | 779 | tags=5%, list=2%, signal=5% |
| 74 | CELLULAR\_COMPONENT\_DISASSEMBLY(GO:0022411) |  | 174 | -0.29 | -1.66 | 0.000 | 0.283 | 0.938 | 5326 | tags=17%, list=17%, signal=21% |
| 75 | HEAT\_SHOCK\_PROTEIN\_BINDING(GO:0031072) |  | 46 | -0.36 | -1.66 | 0.000 | 0.283 | 0.938 | 6908 | tags=28%, list=22%, signal=36% |
| 76 | EPIDERMIS\_DEVELOPMENT(GO:0008544) |  | 103 | -0.35 | -1.65 | 0.000 | 0.295 | 0.951 | 1945 | tags=11%, list=6%, signal=11% |
| 77 | POTASSIUM\_CHANNEL\_REGULATOR\_ACTIVITY(GO:0015459) |  | 18 | -0.58 | -1.65 | 0.026 | 0.294 | 0.951 | 2957 | tags=28%, list=9%, signal=31% |
| 78 | ION\_CHANNEL\_REGULATOR\_ACTIVITY(GO:0099106) |  | 46 | -0.43 | -1.65 | 0.000 | 0.291 | 0.951 | 3144 | tags=20%, list=10%, signal=22% |
| 79 | ORGANELLE\_DISASSEMBLY(GO:1903008) |  | 52 | -0.39 | -1.65 | 0.000 | 0.301 | 0.951 | 5326 | tags=21%, list=17%, signal=25% |
| 80 | ANATOMICAL\_STRUCTURE\_HOMEOSTASIS(GO:0060249) |  | 131 | -0.34 | -1.65 | 0.000 | 0.298 | 0.951 | 3460 | tags=12%, list=11%, signal=14% |
| 81 | NEGATIVE\_REGULATION\_OF\_EXTRINSIC\_APOPTOTIC\_SIGNALING\_PATHWAY(GO:2001237) |  | 15 | -0.45 | -1.64 | 0.000 | 0.304 | 0.971 | 2579 | tags=13%, list=8%, signal=14% |
| 82 | TRANSCRIPTION\_COREPRESSOR\_ACTIVITY(GO:0003714) |  | 95 | -0.34 | -1.64 | 0.000 | 0.304 | 0.987 | 4433 | tags=17%, list=14%, signal=20% |
| 83 | PROTEASOME\_MEDIATED\_UBIQUITIN\_DEPENDENT\_PROTEIN\_CATABOLIC\_PROCESS(GO:0043161) |  | 232 | -0.30 | -1.64 | 0.000 | 0.300 | 0.987 | 3988 | tags=16%, list=13%, signal=18% |
| 84 | ACTIN\_FILAMENT\_SEVERING(GO:0051014) |  | 19 | -0.54 | -1.64 | 0.000 | 0.299 | 0.987 | 6816 | tags=42%, list=21%, signal=54% |
| 85 | RESPONSE\_TO\_NITROGEN\_COMPOUND(GO:1901698) |  | 239 | -0.34 | -1.64 | 0.000 | 0.296 | 0.987 | 4466 | tags=19%, list=14%, signal=22% |
| 86 | REGULATION\_OF\_PROTEIN\_LOCALIZATION(GO:0032880) |  | 91 | -0.36 | -1.64 | 0.000 | 0.299 | 0.987 | 5163 | tags=20%, list=16%, signal=24% |
| 87 | NEUTROPHIL\_DIFFERENTIATION(GO:0030223) |  | 21 | -0.44 | -1.64 | 0.000 | 0.298 | 0.987 | 527 | tags=10%, list=2%, signal=10% |
| 88 | ISOPEPTIDASE\_ACTIVITY(GO:0070122) |  | 15 | -0.41 | -1.64 | 0.000 | 0.299 | 0.987 | 229 | tags=7%, list=1%, signal=7% |
| 89 | EXTRACELLULAR\_MATRIX\_BINDING(GO:0050840) |  | 23 | -0.43 | -1.63 | 0.000 | 0.297 | 0.987 | 3841 | tags=17%, list=12%, signal=20% |
| 90 | PML\_BODY(GO:0016605) |  | 22 | -0.48 | -1.63 | 0.015 | 0.299 | 0.987 | 2436 | tags=18%, list=8%, signal=20% |
| 91 | ESTABLISHMENT\_OR\_MAINTENANCE\_OF\_CELL\_POLARITY(GO:0007163) |  | 113 | -0.31 | -1.63 | 0.000 | 0.297 | 0.987 | 4471 | tags=15%, list=14%, signal=17% |
| 92 | CELL\_MATRIX\_ADHESION(GO:0007160) |  | 62 | -0.36 | -1.63 | 0.000 | 0.295 | 0.987 | 4471 | tags=23%, list=14%, signal=26% |
| 93 | EXOCYST(GO:0000145) |  | 19 | -0.58 | -1.63 | 0.000 | 0.301 | 0.987 | 8664 | tags=53%, list=27%, signal=72% |
| 94 | RESPONSE\_TO\_PEPTIDE\_HORMONE(GO:0043434) |  | 84 | -0.36 | -1.63 | 0.000 | 0.301 | 0.987 | 3975 | tags=20%, list=12%, signal=23% |
| 95 | POSITIVE\_REGULATION\_OF\_HEMOPOIESIS(GO:1903708) |  | 26 | -0.41 | -1.63 | 0.000 | 0.305 | 0.987 | 4370 | tags=23%, list=14%, signal=27% |
| 96 | RESPONSE\_TO\_PEPTIDE(GO:1901652) |  | 85 | -0.36 | -1.62 | 0.000 | 0.305 | 0.987 | 3975 | tags=20%, list=12%, signal=23% |
| 97 | CELLULAR\_RESPONSE\_TO\_PEPTIDE\_HORMONE\_STIMULUS(GO:0071375) |  | 71 | -0.39 | -1.62 | 0.000 | 0.303 | 0.987 | 3975 | tags=20%, list=12%, signal=22% |
| 98 | CELLULAR\_RESPONSE\_TO\_PEPTIDE(GO:1901653) |  | 71 | -0.39 | -1.62 | 0.000 | 0.300 | 0.987 | 3975 | tags=20%, list=12%, signal=22% |
| 99 | MICROTUBULE\_CYTOSKELETON\_ORGANIZATION\_INVOLVED\_IN\_MITOSIS(GO:1902850) |  | 75 | -0.30 | -1.62 | 0.000 | 0.301 | 0.987 | 987 | tags=5%, list=3%, signal=5% |
| 100 | REGULATION\_OF\_DOUBLE\_STRAND\_BREAK\_REPAIR(GO:2000779) |  | 27 | -0.47 | -1.62 | 0.024 | 0.309 | 0.987 | 2574 | tags=19%, list=8%, signal=20% |
| 101 | VESICLE\_COAT(GO:0030120) |  | 47 | -0.33 | -1.61 | 0.026 | 0.313 | 0.987 | 3210 | tags=13%, list=10%, signal=14% |
| 102 | REGULATION\_OF\_CELLULAR\_KETONE\_METABOLIC\_PROCESS(GO:0010565) |  | 27 | -0.41 | -1.61 | 0.000 | 0.311 | 0.987 | 7779 | tags=30%, list=24%, signal=39% |
| 103 | ORGAN\_GROWTH(GO:0035265) |  | 40 | -0.49 | -1.61 | 0.039 | 0.308 | 0.987 | 5603 | tags=35%, list=18%, signal=42% |
| 104 | CONVERGENT\_EXTENSION\_INVOLVED\_IN\_GASTRULATION(GO:0060027) |  | 82 | -0.32 | -1.61 | 0.000 | 0.305 | 0.987 | 4959 | tags=16%, list=16%, signal=19% |
| 105 | EXONUCLEASE\_ACTIVITY\_\_ACTIVE\_WITH\_EITHER\_RIBO\_\_OR\_DEOXYRIBONUCLEIC\_ACIDS\_AND\_PRODUCING\_5'\_PHOSPHOMONOESTERS(GO:0016796) |  | 37 | -0.44 | -1.61 | 0.000 | 0.304 | 0.987 | 2664 | tags=14%, list=8%, signal=15% |
| 106 | AUTOPHAGY(GO:0006914) |  | 196 | -0.27 | -1.61 | 0.000 | 0.310 | 0.987 | 5440 | tags=16%, list=17%, signal=20% |
| 107 | PROCESS\_UTILIZING\_AUTOPHAGIC\_MECHANISM(GO:0061919) |  | 196 | -0.27 | -1.61 | 0.000 | 0.307 | 0.987 | 5440 | tags=16%, list=17%, signal=20% |
| 108 | NEGATIVE\_REGULATION\_OF\_TRANSPORT(GO:0051051) |  | 62 | -0.43 | -1.61 | 0.000 | 0.307 | 0.987 | 4370 | tags=24%, list=14%, signal=28% |
| 109 | MATURATION\_OF\_SSU\_RRNA\_FROM\_TRICISTRONIC\_RRNA\_TRANSCRIPT\_(SSU\_RRNA\_\_5.8S\_RRNA\_\_LSU\_RRNA)(GO:0000462) |  | 27 | -0.51 | -1.61 | 0.069 | 0.309 | 0.987 | 8681 | tags=41%, list=27%, signal=56% |
| 110 | NEGATIVE\_REGULATION\_OF\_CELL\_ADHESION(GO:0007162) |  | 64 | -0.35 | -1.60 | 0.000 | 0.311 | 0.987 | 1360 | tags=16%, list=4%, signal=16% |
| 111 | 90S\_PRERIBOSOME(GO:0030686) |  | 23 | -0.55 | -1.60 | 0.000 | 0.311 | 0.987 | 9140 | tags=48%, list=29%, signal=67% |
| 112 | RESPONSE\_TO\_ORGANONITROGEN\_COMPOUND(GO:0010243) |  | 198 | -0.33 | -1.60 | 0.000 | 0.309 | 0.987 | 4466 | tags=19%, list=14%, signal=22% |
| 113 | STEROID\_HORMONE\_MEDIATED\_SIGNALING\_PATHWAY(GO:0043401) |  | 20 | -0.46 | -1.60 | 0.000 | 0.307 | 0.987 | 947 | tags=15%, list=3%, signal=15% |
| 114 | RIBONUCLEOPROTEIN\_COMPLEX\_ASSEMBLY(GO:0022618) |  | 155 | -0.29 | -1.60 | 0.094 | 0.304 | 1.000 | 10800 | tags=32%, list=34%, signal=48% |
| 115 | HEART\_GROWTH(GO:0060419) |  | 20 | -0.55 | -1.60 | 0.000 | 0.305 | 1.000 | 4290 | tags=35%, list=13%, signal=40% |
| 116 | CARDIAC\_CELL\_DEVELOPMENT(GO:0055006) |  | 42 | -0.43 | -1.60 | 0.050 | 0.305 | 1.000 | 6492 | tags=36%, list=20%, signal=45% |
| 117 | NEGATIVE\_REGULATION\_OF\_DNA\_METABOLIC\_PROCESS(GO:0051053) |  | 49 | -0.42 | -1.60 | 0.000 | 0.314 | 1.000 | 3703 | tags=16%, list=12%, signal=18% |
| 118 | LYMPHOCYTE\_DIFFERENTIATION(GO:0030098) |  | 62 | -0.38 | -1.59 | 0.000 | 0.315 | 1.000 | 2961 | tags=15%, list=9%, signal=16% |
| 119 | RESPONSE\_TO\_OSMOTIC\_STRESS(GO:0006970) |  | 16 | -0.63 | -1.59 | 0.000 | 0.323 | 1.000 | 3475 | tags=38%, list=11%, signal=42% |
| 120 | CELLULAR\_RESPONSE\_TO\_NITROGEN\_COMPOUND(GO:1901699) |  | 128 | -0.36 | -1.59 | 0.000 | 0.322 | 1.000 | 4352 | tags=18%, list=14%, signal=21% |
| 121 | PROTEASOMAL\_PROTEIN\_CATABOLIC\_PROCESS(GO:0010498) |  | 262 | -0.28 | -1.59 | 0.000 | 0.323 | 1.000 | 4285 | tags=15%, list=13%, signal=17% |
| 122 | PROTEIN\_CONTAINING\_COMPLEX\_DISASSEMBLY(GO:0032984) |  | 128 | -0.29 | -1.59 | 0.000 | 0.326 | 1.000 | 5041 | tags=16%, list=16%, signal=19% |
| 123 | POSITIVE\_REGULATION\_OF\_TRANSMEMBRANE\_TRANSPORT(GO:0034764) |  | 25 | -0.47 | -1.58 | 0.000 | 0.329 | 1.000 | 4939 | tags=32%, list=15%, signal=38% |
| 124 | REGULATION\_OF\_RESPONSE\_TO\_DNA\_DAMAGE\_STIMULUS(GO:2001020) |  | 66 | -0.33 | -1.58 | 0.000 | 0.333 | 1.000 | 2574 | tags=12%, list=8%, signal=13% |
| 125 | INTRACILIARY\_TRANSPORT(GO:0042073) |  | 25 | -0.59 | -1.58 | 0.000 | 0.335 | 1.000 | 1757 | tags=20%, list=6%, signal=21% |
| 126 | RIBOSOMAL\_LARGE\_SUBUNIT\_ASSEMBLY(GO:0000027) |  | 16 | -0.56 | -1.58 | 0.029 | 0.339 | 1.000 | 10371 | tags=50%, list=33%, signal=74% |
| 127 | STEREOCILIUM\_BUNDLE(GO:0032421) |  | 16 | -0.61 | -1.57 | 0.000 | 0.339 | 1.000 | 9012 | tags=50%, list=28%, signal=70% |
| 128 | POSTREPLICATION\_REPAIR(GO:0006301) |  | 18 | -0.56 | -1.57 | 0.022 | 0.356 | 1.000 | 5595 | tags=39%, list=18%, signal=47% |
| 129 | MULTICELLULAR\_ORGANISMAL\_HOMEOSTASIS(GO:0048871) |  | 81 | -0.36 | -1.57 | 0.000 | 0.353 | 1.000 | 1567 | tags=9%, list=5%, signal=9% |
| 130 | TRANS\_GOLGI\_NETWORK\_MEMBRANE(GO:0032588) |  | 15 | -0.52 | -1.57 | 0.000 | 0.354 | 1.000 | 4549 | tags=27%, list=14%, signal=31% |
| 131 | UBIQUITIN\_LIKE\_PROTEIN\_LIGASE\_BINDING(GO:0044389) |  | 63 | -0.37 | -1.57 | 0.000 | 0.355 | 1.000 | 5161 | tags=17%, list=16%, signal=21% |
| 132 | NEGATIVE\_REGULATION\_OF\_SECRETION(GO:0051048) |  | 19 | -0.57 | -1.56 | 0.014 | 0.362 | 1.000 | 4370 | tags=32%, list=14%, signal=37% |
| 133 | CELLULAR\_RESPONSE\_TO\_AMINO\_ACID\_STARVATION(GO:0034198) |  | 17 | -0.61 | -1.56 | 0.030 | 0.360 | 1.000 | 7422 | tags=35%, list=23%, signal=46% |
| 134 | INTERMEMBRANE\_LIPID\_TRANSFER\_ACTIVITY(GO:0120013) |  | 17 | -0.55 | -1.56 | 0.000 | 0.360 | 1.000 | 2629 | tags=29%, list=8%, signal=32% |
| 135 | UBIQUITIN\_PROTEIN\_LIGASE\_BINDING(GO:0031625) |  | 57 | -0.38 | -1.56 | 0.000 | 0.372 | 1.000 | 809 | tags=7%, list=3%, signal=7% |
| 136 | OLIGOSACCHARIDE\_METABOLIC\_PROCESS(GO:0009311) |  | 27 | -0.47 | -1.55 | 0.000 | 0.378 | 1.000 | 5012 | tags=30%, list=16%, signal=35% |
| 137 | POLYUBIQUITIN\_MODIFICATION\_DEPENDENT\_PROTEIN\_BINDING(GO:0031593) |  | 32 | -0.34 | -1.55 | 0.061 | 0.378 | 1.000 | 1838 | tags=6%, list=6%, signal=7% |
| 138 | NEGATIVE\_REGULATION\_OF\_ION\_TRANSPORT(GO:0043271) |  | 28 | -0.49 | -1.55 | 0.017 | 0.380 | 1.000 | 2957 | tags=21%, list=9%, signal=24% |
| 139 | AUTOPHAGOSOME\_MEMBRANE(GO:0000421) |  | 21 | -0.44 | -1.55 | 0.048 | 0.382 | 1.000 | 5326 | tags=24%, list=17%, signal=29% |
| 140 | FUCOSYLTRANSFERASE\_ACTIVITY(GO:0008417) |  | 20 | -0.54 | -1.55 | 0.033 | 0.380 | 1.000 | 2096 | tags=25%, list=7%, signal=27% |
| 141 | INSULIN\_LIKE\_GROWTH\_FACTOR\_RECEPTOR\_SIGNALING\_PATHWAY(GO:0048009) |  | 15 | -0.56 | -1.55 | 0.000 | 0.380 | 1.000 | 134 | tags=13%, list=0%, signal=13% |
| 142 | NEGATIVE\_REGULATION\_OF\_RESPONSE\_TO\_DNA\_DAMAGE\_STIMULUS(GO:2001021) |  | 17 | -0.50 | -1.54 | 0.056 | 0.388 | 1.000 | 2574 | tags=24%, list=8%, signal=26% |
| 143 | PROTEIN\_QUALITY\_CONTROL\_FOR\_MISFOLDED\_OR\_INCOMPLETELY\_SYNTHESIZED\_PROTEINS(GO:0006515) |  | 18 | -0.39 | -1.54 | 0.145 | 0.392 | 1.000 | 120 | tags=6%, list=0%, signal=6% |
| 144 | PEPTIDYL\_LYSINE\_MODIFICATION(GO:0018205) |  | 162 | -0.25 | -1.54 | 0.000 | 0.392 | 1.000 | 1206 | tags=5%, list=4%, signal=5% |
| 145 | AUTOPHAGOSOME(GO:0005776) |  | 38 | -0.38 | -1.54 | 0.085 | 0.401 | 1.000 | 5326 | tags=24%, list=17%, signal=28% |
| 146 | MODIFICATION\_DEPENDENT\_PROTEIN\_CATABOLIC\_PROCESS(GO:0019941) |  | 402 | -0.23 | -1.53 | 0.000 | 0.407 | 1.000 | 3988 | tags=11%, list=13%, signal=13% |
| 147 | RIBONUCLEOPROTEIN\_COMPLEX\_SUBUNIT\_ORGANIZATION(GO:0071826) |  | 166 | -0.28 | -1.53 | 0.141 | 0.406 | 1.000 | 10436 | tags=31%, list=33%, signal=45% |
| 148 | RESPONSE\_TO\_HEAT(GO:0009408) |  | 21 | -0.50 | -1.53 | 0.031 | 0.404 | 1.000 | 6376 | tags=38%, list=20%, signal=48% |
| 149 | EPIDERMAL\_CELL\_DIFFERENTIATION(GO:0009913) |  | 79 | -0.33 | -1.53 | 0.000 | 0.403 | 1.000 | 1945 | tags=9%, list=6%, signal=9% |
| 150 | LIGAND\_DEPENDENT\_NUCLEAR\_RECEPTOR\_TRANSCRIPTION\_COACTIVATOR\_ACTIVITY(GO:0030374) |  | 19 | -0.42 | -1.53 | 0.095 | 0.408 | 1.000 | 4548 | tags=16%, list=14%, signal=18% |
| 151 | SMALL\_SUBUNIT\_PROCESSOME(GO:0032040) |  | 35 | -0.45 | -1.53 | 0.029 | 0.414 | 1.000 | 10488 | tags=46%, list=33%, signal=68% |
| 152 | GROWTH\_FACTOR\_BINDING(GO:0019838) |  | 75 | -0.36 | -1.52 | 0.020 | 0.426 | 1.000 | 2686 | tags=12%, list=8%, signal=13% |
| 153 | REGULATION\_OF\_DNA\_METABOLIC\_PROCESS(GO:0051052) |  | 121 | -0.30 | -1.52 | 0.000 | 0.424 | 1.000 | 4044 | tags=12%, list=13%, signal=14% |
| 154 | MELANOSOME\_TRANSPORT(GO:0032402) |  | 27 | -0.44 | -1.52 | 0.000 | 0.429 | 1.000 | 2498 | tags=22%, list=8%, signal=24% |
| 155 | PIGMENT\_GRANULE\_TRANSPORT(GO:0051904) |  | 27 | -0.44 | -1.52 | 0.000 | 0.426 | 1.000 | 2498 | tags=22%, list=8%, signal=24% |
| 156 | ESTABLISHMENT\_OF\_MELANOSOME\_LOCALIZATION(GO:0032401) |  | 28 | -0.43 | -1.52 | 0.000 | 0.426 | 1.000 | 2498 | tags=21%, list=8%, signal=23% |
| 157 | MITOCHONDRIAL\_SMALL\_RIBOSOMAL\_SUBUNIT(GO:0005763) |  | 19 | -0.57 | -1.52 | 0.036 | 0.424 | 1.000 | 3528 | tags=21%, list=11%, signal=24% |
| 158 | ORGANELLAR\_SMALL\_RIBOSOMAL\_SUBUNIT(GO:0000314) |  | 19 | -0.57 | -1.52 | 0.036 | 0.422 | 1.000 | 3528 | tags=21%, list=11%, signal=24% |
| 159 | LIPID\_STORAGE(GO:0019915) |  | 20 | -0.44 | -1.52 | 0.029 | 0.419 | 1.000 | 4295 | tags=20%, list=13%, signal=23% |
| 160 | RIBOSOME\_ASSEMBLY(GO:0042255) |  | 39 | -0.45 | -1.52 | 0.127 | 0.418 | 1.000 | 10800 | tags=44%, list=34%, signal=66% |
| 161 | MEMBRANE\_COAT(GO:0030117) |  | 71 | -0.26 | -1.52 | 0.026 | 0.421 | 1.000 | 3210 | tags=11%, list=10%, signal=13% |
| 162 | COATED\_MEMBRANE(GO:0048475) |  | 71 | -0.26 | -1.52 | 0.026 | 0.419 | 1.000 | 3210 | tags=11%, list=10%, signal=13% |
| 163 | TELENCEPHALON\_DEVELOPMENT(GO:0021537) |  | 24 | -0.42 | -1.52 | 0.000 | 0.417 | 1.000 | 9566 | tags=46%, list=30%, signal=65% |
| 164 | REGULATION\_OF\_PROTEIN\_SECRETION(GO:0050708) |  | 27 | -0.47 | -1.51 | 0.000 | 0.421 | 1.000 | 5163 | tags=26%, list=16%, signal=31% |
| 165 | REGULATION\_OF\_CELLULAR\_LOCALIZATION(GO:0060341) |  | 124 | -0.31 | -1.51 | 0.000 | 0.420 | 1.000 | 4939 | tags=18%, list=15%, signal=21% |
| 166 | NEGATIVE\_REGULATION\_OF\_SECRETION\_BY\_CELL(GO:1903531) |  | 17 | -0.55 | -1.51 | 0.014 | 0.417 | 1.000 | 4370 | tags=29%, list=14%, signal=34% |
| 167 | REGULATION\_OF\_LEUKOCYTE\_CHEMOTAXIS(GO:0002688) |  | 20 | -0.44 | -1.51 | 0.092 | 0.417 | 1.000 | 446 | tags=10%, list=1%, signal=10% |
| 168 | MITOTIC\_SPINDLE\_ORGANIZATION(GO:0007052) |  | 53 | -0.31 | -1.51 | 0.028 | 0.416 | 1.000 | 987 | tags=6%, list=3%, signal=6% |
| 169 | DETERMINATION\_OF\_LIVER\_LEFT\_RIGHT\_ASYMMETRY(GO:0071910) |  | 19 | -0.41 | -1.51 | 0.000 | 0.415 | 1.000 | 4939 | tags=26%, list=15%, signal=31% |
| 170 | POSITIVE\_REGULATION\_OF\_AXONOGENESIS(GO:0050772) |  | 35 | -0.36 | -1.51 | 0.000 | 0.413 | 1.000 | 5213 | tags=23%, list=16%, signal=27% |
| 171 | REGULATION\_OF\_SODIUM\_ION\_TRANSMEMBRANE\_TRANSPORTER\_ACTIVITY(GO:2000649) |  | 34 | -0.45 | -1.51 | 0.000 | 0.412 | 1.000 | 6295 | tags=38%, list=20%, signal=48% |
| 172 | POSITIVE\_REGULATION\_OF\_ION\_TRANSPORT(GO:0043270) |  | 24 | -0.44 | -1.51 | 0.000 | 0.410 | 1.000 | 4939 | tags=33%, list=15%, signal=39% |
| 173 | RIBONUCLEOPROTEIN\_COMPLEX\_BINDING(GO:0043021) |  | 59 | -0.30 | -1.51 | 0.032 | 0.409 | 1.000 | 1387 | tags=7%, list=4%, signal=7% |
| 174 | HIPPO\_SIGNALING(GO:0035329) |  | 23 | -0.47 | -1.51 | 0.032 | 0.409 | 1.000 | 5713 | tags=30%, list=18%, signal=37% |
| 175 | AGING(GO:0007568) |  | 21 | -0.49 | -1.51 | 0.029 | 0.412 | 1.000 | 1567 | tags=14%, list=5%, signal=15% |
| 176 | SKELETAL\_MUSCLE\_TISSUE\_DEVELOPMENT(GO:0007519) |  | 128 | -0.28 | -1.51 | 0.022 | 0.411 | 1.000 | 1481 | tags=7%, list=5%, signal=7% |
| 177 | POSITIVE\_REGULATION\_OF\_VASCULATURE\_DEVELOPMENT(GO:1904018) |  | 43 | -0.38 | -1.51 | 0.031 | 0.410 | 1.000 | 4939 | tags=23%, list=15%, signal=27% |
| 178 | NEGATIVE\_REGULATION\_OF\_TRANSFORMING\_GROWTH\_FACTOR\_BETA\_RECEPTOR\_SIGNALING\_PATHWAY(GO:0030512) |  | 17 | -0.41 | -1.50 | 0.055 | 0.416 | 1.000 | 1934 | tags=12%, list=6%, signal=13% |
| 179 | NEGATIVE\_REGULATION\_OF\_CELLULAR\_RESPONSE\_TO\_TRANSFORMING\_GROWTH\_FACTOR\_BETA\_STIMULUS(GO:1903845) |  | 17 | -0.41 | -1.50 | 0.055 | 0.414 | 1.000 | 1934 | tags=12%, list=6%, signal=13% |
| 180 | UBIQUITIN\_LIKE\_PROTEIN\_CONJUGATING\_ENZYME\_BINDING(GO:0044390) |  | 25 | -0.42 | -1.50 | 0.000 | 0.413 | 1.000 | 5663 | tags=28%, list=18%, signal=34% |
| 181 | LEUKOCYTE\_DIFFERENTIATION(GO:0002521) |  | 116 | -0.30 | -1.50 | 0.030 | 0.411 | 1.000 | 3216 | tags=12%, list=10%, signal=13% |
| 182 | CARDIAC\_MUSCLE\_CELL\_PROLIFERATION(GO:0060038) |  | 16 | -0.54 | -1.50 | 0.000 | 0.419 | 1.000 | 4290 | tags=38%, list=13%, signal=43% |
| 183 | 3'\_5'\_EXONUCLEASE\_ACTIVITY(GO:0008408) |  | 41 | -0.38 | -1.50 | 0.038 | 0.419 | 1.000 | 5829 | tags=24%, list=18%, signal=30% |
| 184 | REGULATION\_OF\_PROTEASOMAL\_PROTEIN\_CATABOLIC\_PROCESS(GO:0061136) |  | 45 | -0.30 | -1.50 | 0.035 | 0.418 | 1.000 | 3960 | tags=13%, list=12%, signal=15% |
| 185 | REGULATION\_OF\_CELLULAR\_PROTEIN\_LOCALIZATION(GO:1903827) |  | 52 | -0.36 | -1.50 | 0.000 | 0.418 | 1.000 | 3880 | tags=17%, list=12%, signal=20% |
| 186 | MODIFICATION\_DEPENDENT\_MACROMOLECULE\_CATABOLIC\_PROCESS(GO:0043632) |  | 409 | -0.23 | -1.50 | 0.000 | 0.419 | 1.000 | 3988 | tags=11%, list=13%, signal=13% |
| 187 | CELLULAR\_IRON\_ION\_HOMEOSTASIS(GO:0006879) |  | 28 | -0.40 | -1.50 | 0.056 | 0.417 | 1.000 | 876 | tags=11%, list=3%, signal=11% |
| 188 | PROTEIN\_METHYLTRANSFERASE\_ACTIVITY(GO:0008276) |  | 74 | -0.32 | -1.50 | 0.074 | 0.421 | 1.000 | 1027 | tags=5%, list=3%, signal=6% |
| 189 | PROTEASOME\_ACCESSORY\_COMPLEX(GO:0022624) |  | 22 | -0.54 | -1.50 | 0.019 | 0.420 | 1.000 | 2107 | tags=18%, list=7%, signal=19% |
| 190 | HORMONE\_RECEPTOR\_BINDING(GO:0051427) |  | 39 | -0.43 | -1.50 | 0.026 | 0.419 | 1.000 | 947 | tags=8%, list=3%, signal=8% |
| 191 | UBIQUITIN\_CONJUGATING\_ENZYME\_BINDING(GO:0031624) |  | 24 | -0.43 | -1.49 | 0.000 | 0.421 | 1.000 | 5663 | tags=29%, list=18%, signal=35% |
| 192 | B\_CELL\_DIFFERENTIATION(GO:0030183) |  | 16 | -0.50 | -1.49 | 0.000 | 0.419 | 1.000 | 2961 | tags=19%, list=9%, signal=21% |
| 193 | REGULATION\_OF\_METAL\_ION\_TRANSPORT(GO:0010959) |  | 90 | -0.37 | -1.49 | 0.042 | 0.418 | 1.000 | 5936 | tags=30%, list=19%, signal=37% |
| 194 | MAINTENANCE\_OF\_LOCATION(GO:0051235) |  | 82 | -0.34 | -1.49 | 0.000 | 0.417 | 1.000 | 5493 | tags=24%, list=17%, signal=29% |
| 195 | MICROTUBULE\_POLYMERIZATION\_OR\_DEPOLYMERIZATION(GO:0031109) |  | 57 | -0.32 | -1.49 | 0.000 | 0.416 | 1.000 | 3272 | tags=14%, list=10%, signal=16% |
| 196 | CELLULAR\_KETONE\_METABOLIC\_PROCESS(GO:0042180) |  | 68 | -0.34 | -1.49 | 0.037 | 0.416 | 1.000 | 4947 | tags=19%, list=16%, signal=23% |
| 197 | MUSCLE\_TISSUE\_DEVELOPMENT(GO:0060537) |  | 223 | -0.29 | -1.49 | 0.017 | 0.416 | 1.000 | 6852 | tags=26%, list=21%, signal=33% |
| 198 | ICOSANOID\_BIOSYNTHETIC\_PROCESS(GO:0046456) |  | 16 | -0.51 | -1.49 | 0.000 | 0.417 | 1.000 | 2397 | tags=25%, list=8%, signal=27% |
| 199 | BASEMENT\_MEMBRANE\_ORGANIZATION(GO:0071711) |  | 18 | -0.49 | -1.49 | 0.101 | 0.416 | 1.000 | 8372 | tags=39%, list=26%, signal=53% |
| 200 | MORPHOGENESIS\_OF\_EMBRYONIC\_EPITHELIUM(GO:0016331) |  | 63 | -0.36 | -1.49 | 0.096 | 0.421 | 1.000 | 1218 | tags=6%, list=4%, signal=7% |
| 201 | POSITIVE\_REGULATION\_OF\_EPITHELIAL\_CELL\_MIGRATION(GO:0010634) |  | 18 | -0.52 | -1.49 | 0.099 | 0.420 | 1.000 | 6514 | tags=39%, list=20%, signal=49% |
| 202 | HISTONE\_METHYLTRANSFERASE\_COMPLEX(GO:0035097) |  | 46 | -0.28 | -1.49 | 0.031 | 0.422 | 1.000 | 4303 | tags=9%, list=13%, signal=10% |
| 203 | REGULATION\_OF\_POTASSIUM\_ION\_TRANSMEMBRANE\_TRANSPORT(GO:1901379) |  | 17 | -0.56 | -1.49 | 0.045 | 0.422 | 1.000 | 3762 | tags=29%, list=12%, signal=33% |
| 204 | CELLULAR\_RESPONSE\_TO\_ORGANONITROGEN\_COMPOUND(GO:0071417) |  | 97 | -0.34 | -1.48 | 0.035 | 0.422 | 1.000 | 5660 | tags=23%, list=18%, signal=27% |
| 205 | MELANOSOME(GO:0042470) |  | 26 | -0.51 | -1.48 | 0.050 | 0.428 | 1.000 | 6848 | tags=46%, list=21%, signal=59% |
| 206 | PIGMENT\_GRANULE(GO:0048770) |  | 26 | -0.51 | -1.48 | 0.050 | 0.426 | 1.000 | 6848 | tags=46%, list=21%, signal=59% |
| 207 | CELLULAR\_RESPONSE\_TO\_HORMONE\_STIMULUS(GO:0032870) |  | 169 | -0.31 | -1.48 | 0.000 | 0.424 | 1.000 | 3975 | tags=17%, list=12%, signal=19% |
| 208 | NEGATIVE\_REGULATION\_OF\_MITOTIC\_NUCLEAR\_DIVISION(GO:0045839) |  | 23 | -0.45 | -1.48 | 0.066 | 0.423 | 1.000 | 1574 | tags=13%, list=5%, signal=14% |
| 209 | MITOTIC\_SPINDLE\_ASSEMBLY(GO:0090307) |  | 28 | -0.38 | -1.48 | 0.080 | 0.424 | 1.000 | 987 | tags=7%, list=3%, signal=7% |
| 210 | TUBE\_CLOSURE(GO:0060606) |  | 16 | -0.47 | -1.48 | 0.052 | 0.424 | 1.000 | 894 | tags=6%, list=3%, signal=6% |
| 211 | EPITHALAMUS\_DEVELOPMENT(GO:0021538) |  | 22 | -0.49 | -1.48 | 0.025 | 0.423 | 1.000 | 7911 | tags=32%, list=25%, signal=42% |
| 212 | REGULATION\_OF\_PROTEASOMAL\_UBIQUITIN\_DEPENDENT\_PROTEIN\_CATABOLIC\_PROCESS(GO:0032434) |  | 41 | -0.32 | -1.48 | 0.035 | 0.421 | 1.000 | 3960 | tags=15%, list=12%, signal=17% |
| 213 | CELLULAR\_RESPONSE\_TO\_LIPID(GO:0071396) |  | 74 | -0.35 | -1.48 | 0.051 | 0.421 | 1.000 | 1735 | tags=14%, list=5%, signal=14% |
| 214 | REGULATION\_OF\_FILOPODIUM\_ASSEMBLY(GO:0051489) |  | 15 | -0.52 | -1.48 | 0.068 | 0.421 | 1.000 | 342 | tags=7%, list=1%, signal=7% |
| 215 | RIBONUCLEOPROTEIN\_COMPLEX\_BIOGENESIS(GO:0022613) |  | 328 | -0.29 | -1.48 | 0.135 | 0.420 | 1.000 | 9140 | tags=30%, list=29%, signal=42% |
| 216 | RRNA\_PROCESSING(GO:0006364) |  | 151 | -0.37 | -1.47 | 0.175 | 0.423 | 1.000 | 9140 | tags=40%, list=29%, signal=55% |
| 217 | RIBONUCLEOSIDE\_METABOLIC\_PROCESS(GO:0009119) |  | 64 | -0.30 | -1.47 | 0.000 | 0.423 | 1.000 | 6520 | tags=28%, list=20%, signal=35% |
| 218 | TRANSCRIPTION\_FACTOR\_ACTIVITY\_\_TRANSCRIPTION\_FACTOR\_BINDING(GO:0000989) |  | 288 | -0.25 | -1.47 | 0.047 | 0.423 | 1.000 | 3959 | tags=11%, list=12%, signal=13% |
| 219 | TRANSCRIPTION\_COFACTOR\_ACTIVITY(GO:0003712) |  | 288 | -0.25 | -1.47 | 0.047 | 0.421 | 1.000 | 3959 | tags=11%, list=12%, signal=13% |
| 220 | RESPONSE\_TO\_OXYGEN\_CONTAINING\_COMPOUND(GO:1901700) |  | 288 | -0.27 | -1.47 | 0.000 | 0.420 | 1.000 | 4810 | tags=19%, list=15%, signal=22% |
| 221 | CARDIAC\_MUSCLE\_CONTRACTION(GO:0060048) |  | 44 | -0.45 | -1.47 | 0.000 | 0.425 | 1.000 | 5990 | tags=41%, list=19%, signal=50% |
| 222 | HEART\_VALVE\_DEVELOPMENT(GO:0003170) |  | 32 | -0.44 | -1.47 | 0.026 | 0.425 | 1.000 | 5013 | tags=25%, list=16%, signal=30% |
| 223 | POSITIVE\_REGULATION\_OF\_ION\_TRANSMEMBRANE\_TRANSPORT(GO:0034767) |  | 20 | -0.46 | -1.47 | 0.000 | 0.423 | 1.000 | 4939 | tags=35%, list=15%, signal=41% |
| 224 | REGULATION\_OF\_PROTEOLYSIS\_INVOLVED\_IN\_CELLULAR\_PROTEIN\_CATABOLIC\_PROCESS(GO:1903050) |  | 47 | -0.30 | -1.47 | 0.000 | 0.425 | 1.000 | 3960 | tags=13%, list=12%, signal=15% |
| 225 | MELANOSOME\_LOCALIZATION(GO:0032400) |  | 30 | -0.42 | -1.47 | 0.021 | 0.424 | 1.000 | 2498 | tags=20%, list=8%, signal=22% |
| 226 | PRERIBOSOME(GO:0030684) |  | 69 | -0.44 | -1.47 | 0.108 | 0.425 | 1.000 | 10256 | tags=45%, list=32%, signal=66% |
| 227 | SULFUR\_AMINO\_ACID\_METABOLIC\_PROCESS(GO:0000096) |  | 24 | -0.33 | -1.47 | 0.035 | 0.427 | 1.000 | 2232 | tags=13%, list=7%, signal=13% |
| 228 | REGULATION\_OF\_CELLULAR\_PROTEIN\_CATABOLIC\_PROCESS(GO:1903362) |  | 48 | -0.29 | -1.47 | 0.000 | 0.425 | 1.000 | 3960 | tags=13%, list=12%, signal=14% |
| 229 | CARDIAC\_MUSCLE\_TISSUE\_GROWTH(GO:0055017) |  | 18 | -0.50 | -1.47 | 0.069 | 0.425 | 1.000 | 4290 | tags=33%, list=13%, signal=38% |
| 230 | NEGATIVE\_REGULATION\_OF\_CELL\_CYCLE(GO:0045786) |  | 121 | -0.28 | -1.47 | 0.063 | 0.426 | 1.000 | 1590 | tags=9%, list=5%, signal=10% |
| 231 | LAMELLIPODIUM\_ORGANIZATION(GO:0097581) |  | 16 | -0.52 | -1.47 | 0.087 | 0.425 | 1.000 | 1030 | tags=13%, list=3%, signal=13% |
| 232 | REGULATION\_OF\_SODIUM\_ION\_TRANSMEMBRANE\_TRANSPORT(GO:1902305) |  | 35 | -0.43 | -1.46 | 0.000 | 0.426 | 1.000 | 6295 | tags=37%, list=20%, signal=46% |
| 233 | STRIATED\_MUSCLE\_TISSUE\_DEVELOPMENT(GO:0014706) |  | 215 | -0.29 | -1.46 | 0.039 | 0.426 | 1.000 | 6771 | tags=25%, list=21%, signal=32% |
| 234 | MODIFICATION\_DEPENDENT\_PROTEIN\_BINDING(GO:0140030) |  | 64 | -0.29 | -1.46 | 0.061 | 0.429 | 1.000 | 4272 | tags=9%, list=13%, signal=11% |
| 235 | STRIATED\_MUSCLE\_CONTRACTION(GO:0006941) |  | 58 | -0.41 | -1.46 | 0.058 | 0.430 | 1.000 | 6330 | tags=40%, list=20%, signal=49% |
| 236 | REGULATION\_OF\_PEPTIDE\_SECRETION(GO:0002791) |  | 28 | -0.46 | -1.46 | 0.000 | 0.432 | 1.000 | 5163 | tags=25%, list=16%, signal=30% |
| 237 | MAGNESIUM\_ION\_BINDING(GO:0000287) |  | 105 | -0.30 | -1.46 | 0.016 | 0.437 | 1.000 | 5117 | tags=22%, list=16%, signal=26% |
| 238 | CARDIAC\_CONDUCTION(GO:0061337) |  | 15 | -0.44 | -1.46 | 0.097 | 0.439 | 1.000 | 3144 | tags=33%, list=10%, signal=37% |
| 239 | REGULATION\_OF\_PROTEIN\_TRANSPORT(GO:0051223) |  | 50 | -0.34 | -1.45 | 0.000 | 0.443 | 1.000 | 2489 | tags=12%, list=8%, signal=13% |
| 240 | REGULATION\_OF\_ESTABLISHMENT\_OF\_PROTEIN\_LOCALIZATION(GO:0070201) |  | 50 | -0.34 | -1.45 | 0.000 | 0.441 | 1.000 | 2489 | tags=12%, list=8%, signal=13% |
| 241 | REGULATION\_OF\_CALCIUM\_ION\_TRANSPORT(GO:0051924) |  | 42 | -0.42 | -1.45 | 0.061 | 0.442 | 1.000 | 5936 | tags=33%, list=19%, signal=41% |
| 242 | RECYCLING\_ENDOSOME(GO:0055037) |  | 78 | -0.30 | -1.45 | 0.000 | 0.445 | 1.000 | 4229 | tags=21%, list=13%, signal=24% |
| 243 | UBIQUITIN\_DEPENDENT\_PROTEIN\_CATABOLIC\_PROCESS(GO:0006511) |  | 393 | -0.22 | -1.45 | 0.000 | 0.448 | 1.000 | 3988 | tags=11%, list=13%, signal=13% |
| 244 | POSITIVE\_REGULATION\_OF\_PROTEIN\_COMPLEX\_ASSEMBLY(GO:0031334) |  | 87 | -0.30 | -1.45 | 0.000 | 0.448 | 1.000 | 3541 | tags=15%, list=11%, signal=17% |
| 245 | CELLULAR\_PROTEIN\_CATABOLIC\_PROCESS(GO:0044257) |  | 458 | -0.22 | -1.45 | 0.000 | 0.453 | 1.000 | 4285 | tags=12%, list=13%, signal=13% |
| 246 | TRANSCRIPTION\_FACTOR\_ACTIVITY\_\_PROTEIN\_BINDING(GO:0000988) |  | 293 | -0.24 | -1.45 | 0.048 | 0.452 | 1.000 | 3959 | tags=11%, list=12%, signal=12% |
| 247 | DIGESTIVE\_SYSTEM\_PROCESS(GO:0022600) |  | 21 | -0.42 | -1.45 | 0.000 | 0.455 | 1.000 | 3710 | tags=24%, list=12%, signal=27% |
| 248 | POSTERIOR\_LATERAL\_LINE\_SYSTEM\_DEVELOPMENT(GO:0048915) |  | 76 | -0.28 | -1.44 | 0.032 | 0.462 | 1.000 | 5980 | tags=22%, list=19%, signal=27% |
| 249 | NUCLEOTIDE\_DIPHOSPHATASE\_ACTIVITY(GO:0004551) |  | 17 | -0.51 | -1.44 | 0.087 | 0.461 | 1.000 | 7904 | tags=41%, list=25%, signal=55% |
| 250 | RESPIRATORY\_SYSTEM\_DEVELOPMENT(GO:0060541) |  | 16 | -0.53 | -1.44 | 0.109 | 0.463 | 1.000 | 1206 | tags=19%, list=4%, signal=19% |
| 251 | RESPONSE\_TO\_EXTRACELLULAR\_STIMULUS(GO:0009991) |  | 113 | -0.34 | -1.44 | 0.019 | 0.461 | 1.000 | 5141 | tags=24%, list=16%, signal=28% |
| 252 | PROTEOLYSIS\_INVOLVED\_IN\_CELLULAR\_PROTEIN\_CATABOLIC\_PROCESS(GO:0051603) |  | 454 | -0.22 | -1.44 | 0.000 | 0.461 | 1.000 | 4285 | tags=12%, list=13%, signal=14% |
| 253 | SMALL\_RIBOSOMAL\_SUBUNIT(GO:0015935) |  | 57 | -0.45 | -1.44 | 0.196 | 0.459 | 1.000 | 11045 | tags=47%, list=35%, signal=72% |
| 254 | PROTEASOME\_REGULATORY\_PARTICLE(GO:0005838) |  | 20 | -0.57 | -1.44 | 0.100 | 0.467 | 1.000 | 2107 | tags=20%, list=7%, signal=21% |
| 255 | INTRACELLULAR\_STEROID\_HORMONE\_RECEPTOR\_SIGNALING\_PATHWAY(GO:0030518) |  | 17 | -0.44 | -1.44 | 0.061 | 0.466 | 1.000 | 947 | tags=12%, list=3%, signal=12% |
| 256 | GTPASE\_ACTIVITY(GO:0003924) |  | 319 | -0.26 | -1.44 | 0.000 | 0.465 | 1.000 | 4996 | tags=18%, list=16%, signal=21% |
| 257 | REGULATION\_OF\_VASCULATURE\_DEVELOPMENT(GO:1901342) |  | 102 | -0.28 | -1.44 | 0.050 | 0.466 | 1.000 | 6960 | tags=23%, list=22%, signal=29% |
| 258 | REGULATION\_OF\_CELL\_MORPHOGENESIS(GO:0022604) |  | 207 | -0.27 | -1.43 | 0.017 | 0.468 | 1.000 | 5618 | tags=18%, list=18%, signal=22% |
| 259 | RNA\_PHOSPHODIESTER\_BOND\_HYDROLYSIS\_\_ENDONUCLEOLYTIC(GO:0090502) |  | 19 | -0.56 | -1.43 | 0.051 | 0.467 | 1.000 | 10189 | tags=74%, list=32%, signal=108% |
| 260 | DNA\_BIOSYNTHETIC\_PROCESS(GO:0071897) |  | 39 | -0.40 | -1.43 | 0.052 | 0.473 | 1.000 | 6127 | tags=23%, list=19%, signal=29% |
| 261 | METHYLTRANSFERASE\_COMPLEX(GO:0034708) |  | 57 | -0.24 | -1.43 | 0.021 | 0.473 | 1.000 | 9632 | tags=26%, list=30%, signal=38% |
| 262 | ADENYLYLTRANSFERASE\_ACTIVITY(GO:0070566) |  | 24 | -0.35 | -1.43 | 0.069 | 0.480 | 1.000 | 8226 | tags=33%, list=26%, signal=45% |
| 263 | INSULIN\_SECRETION(GO:0030073) |  | 27 | -0.43 | -1.43 | 0.064 | 0.479 | 1.000 | 4587 | tags=22%, list=14%, signal=26% |
| 264 | EMBRYONIC\_RETINA\_MORPHOGENESIS\_IN\_CAMERA\_TYPE\_EYE(GO:0060059) |  | 29 | -0.45 | -1.43 | 0.087 | 0.481 | 1.000 | 10926 | tags=59%, list=34%, signal=89% |
| 265 | NUCLEAR\_TRANSCRIBED\_MRNA\_POLY(A)\_TAIL\_SHORTENING(GO:0000289) |  | 19 | -0.42 | -1.43 | 0.023 | 0.480 | 1.000 | 2968 | tags=21%, list=9%, signal=23% |
| 266 | RESPONSE\_TO\_STARVATION(GO:0042594) |  | 67 | -0.39 | -1.43 | 0.074 | 0.481 | 1.000 | 5141 | tags=24%, list=16%, signal=28% |
| 267 | CYTOSKELETON\_DEPENDENT\_CYTOKINESIS(GO:0061640) |  | 57 | -0.33 | -1.43 | 0.055 | 0.480 | 1.000 | 977 | tags=5%, list=3%, signal=5% |
| 268 | MELANOCYTE\_DIFFERENTIATION(GO:0030318) |  | 41 | -0.38 | -1.43 | 0.076 | 0.478 | 1.000 | 4519 | tags=22%, list=14%, signal=26% |
| 269 | CYTOSOL(GO:0005829) |  | 971 | -0.21 | -1.43 | 0.000 | 0.477 | 1.000 | 6520 | tags=19%, list=20%, signal=23% |
| 270 | SITE\_OF\_DOUBLE\_STRAND\_BREAK(GO:0035861) |  | 23 | -0.39 | -1.42 | 0.036 | 0.476 | 1.000 | 1069 | tags=9%, list=3%, signal=9% |
| 271 | STEROL\_TRANSPORTER\_ACTIVITY(GO:0015248) |  | 22 | -0.47 | -1.42 | 0.024 | 0.475 | 1.000 | 2977 | tags=23%, list=9%, signal=25% |
| 272 | NAD\_BINDING(GO:0051287) |  | 45 | -0.33 | -1.42 | 0.150 | 0.474 | 1.000 | 7144 | tags=29%, list=22%, signal=37% |
| 273 | CELL\_SUBSTRATE\_ADHESION(GO:0031589) |  | 107 | -0.29 | -1.42 | 0.049 | 0.475 | 1.000 | 4471 | tags=18%, list=14%, signal=21% |
| 274 | T\_CELL\_DIFFERENTIATION(GO:0030217) |  | 43 | -0.38 | -1.42 | 0.000 | 0.477 | 1.000 | 2961 | tags=14%, list=9%, signal=15% |
| 275 | CARDIAC\_MUSCLE\_TISSUE\_DEVELOPMENT(GO:0048738) |  | 100 | -0.36 | -1.42 | 0.122 | 0.479 | 1.000 | 6771 | tags=31%, list=21%, signal=39% |
| 276 | POSITIVE\_REGULATION\_OF\_CATION\_TRANSMEMBRANE\_TRANSPORT(GO:1904064) |  | 18 | -0.49 | -1.42 | 0.000 | 0.477 | 1.000 | 5831 | tags=44%, list=18%, signal=54% |
| 277 | SULFURIC\_ESTER\_HYDROLASE\_ACTIVITY(GO:0008484) |  | 15 | -0.50 | -1.42 | 0.064 | 0.480 | 1.000 | 3654 | tags=20%, list=11%, signal=23% |
| 278 | CARDIOCYTE\_DIFFERENTIATION(GO:0035051) |  | 71 | -0.36 | -1.42 | 0.079 | 0.481 | 1.000 | 6529 | tags=31%, list=20%, signal=39% |
| 279 | RIBOSOME\_BINDING(GO:0043022) |  | 34 | -0.31 | -1.42 | 0.098 | 0.481 | 1.000 | 1387 | tags=9%, list=4%, signal=9% |
| 280 | MUSCLE\_CELL\_DEVELOPMENT(GO:0055001) |  | 201 | -0.26 | -1.42 | 0.052 | 0.480 | 1.000 | 6492 | tags=22%, list=20%, signal=28% |
| 281 | POSITIVE\_REGULATION\_OF\_ENDOTHELIAL\_CELL\_MIGRATION(GO:0010595) |  | 15 | -0.52 | -1.42 | 0.160 | 0.481 | 1.000 | 4370 | tags=33%, list=14%, signal=39% |
| 282 | HEART\_PROCESS(GO:0003015) |  | 157 | -0.29 | -1.42 | 0.000 | 0.482 | 1.000 | 6334 | tags=24%, list=20%, signal=29% |
| 283 | PEPTIDYL\_THREONINE\_PHOSPHORYLATION(GO:0018107) |  | 24 | -0.47 | -1.42 | 0.119 | 0.480 | 1.000 | 1638 | tags=13%, list=5%, signal=13% |
| 284 | NEGATIVE\_REGULATION\_OF\_DNA\_BINDING\_TRANSCRIPTION\_FACTOR\_ACTIVITY(GO:0043433) |  | 18 | -0.42 | -1.42 | 0.111 | 0.480 | 1.000 | 6145 | tags=28%, list=19%, signal=34% |
| 285 | ENDODERMAL\_CELL\_DIFFERENTIATION(GO:0035987) |  | 30 | -0.44 | -1.41 | 0.084 | 0.480 | 1.000 | 5220 | tags=23%, list=16%, signal=28% |
| 286 | REGULATION\_OF\_ARF\_PROTEIN\_SIGNAL\_TRANSDUCTION(GO:0032012) |  | 18 | -0.39 | -1.41 | 0.000 | 0.480 | 1.000 | 5018 | tags=28%, list=16%, signal=33% |
| 287 | ARF\_PROTEIN\_SIGNAL\_TRANSDUCTION(GO:0032011) |  | 18 | -0.39 | -1.41 | 0.000 | 0.479 | 1.000 | 5018 | tags=28%, list=16%, signal=33% |
| 288 | CHANNEL\_REGULATOR\_ACTIVITY(GO:0016247) |  | 69 | -0.36 | -1.41 | 0.055 | 0.478 | 1.000 | 5938 | tags=28%, list=19%, signal=34% |
| 289 | ISOMERASE\_ACTIVITY(GO:0016853) |  | 136 | -0.32 | -1.41 | 0.025 | 0.477 | 1.000 | 6895 | tags=23%, list=22%, signal=29% |
| 290 | APICAL\_PLASMA\_MEMBRANE(GO:0016324) |  | 90 | -0.30 | -1.41 | 0.025 | 0.478 | 1.000 | 3684 | tags=16%, list=12%, signal=18% |
| 291 | REGULATION\_OF\_PEPTIDE\_TRANSPORT(GO:0090087) |  | 51 | -0.33 | -1.41 | 0.000 | 0.482 | 1.000 | 2489 | tags=12%, list=8%, signal=13% |
| 292 | PROTEIN\_CONTAINING\_COMPLEX\_BINDING(GO:0044877) |  | 514 | -0.20 | -1.41 | 0.000 | 0.482 | 1.000 | 2584 | tags=7%, list=8%, signal=7% |
| 293 | PROTEIN\_FOLDING(GO:0006457) |  | 127 | -0.30 | -1.41 | 0.093 | 0.481 | 1.000 | 6472 | tags=21%, list=20%, signal=27% |
| 294 | CELLULAR\_RESPONSE\_TO\_STEROID\_HORMONE\_STIMULUS(GO:0071383) |  | 28 | -0.36 | -1.41 | 0.000 | 0.480 | 1.000 | 1287 | tags=14%, list=4%, signal=15% |
| 295 | EXTRINSIC\_APOPTOTIC\_SIGNALING\_PATHWAY(GO:0097191) |  | 48 | -0.31 | -1.41 | 0.033 | 0.480 | 1.000 | 182 | tags=4%, list=1%, signal=4% |
| 296 | REGULATION\_OF\_POTASSIUM\_ION\_TRANSPORT(GO:0043266) |  | 24 | -0.50 | -1.41 | 0.081 | 0.479 | 1.000 | 5664 | tags=33%, list=18%, signal=41% |
| 297 | PEROXISOMAL\_PART(GO:0044439) |  | 36 | -0.41 | -1.41 | 0.018 | 0.480 | 1.000 | 2519 | tags=14%, list=8%, signal=15% |
| 298 | MICROBODY\_PART(GO:0044438) |  | 36 | -0.41 | -1.41 | 0.018 | 0.479 | 1.000 | 2519 | tags=14%, list=8%, signal=15% |
| 299 | SULFATION(GO:0051923) |  | 23 | -0.52 | -1.41 | 0.078 | 0.479 | 1.000 | 5477 | tags=35%, list=17%, signal=42% |
| 300 | CORTICAL\_CYTOSKELETON(GO:0030863) |  | 44 | -0.35 | -1.41 | 0.025 | 0.480 | 1.000 | 1670 | tags=9%, list=5%, signal=10% |
| 301 | RESPONSE\_TO\_NUTRIENT\_LEVELS(GO:0031667) |  | 111 | -0.33 | -1.41 | 0.019 | 0.478 | 1.000 | 5141 | tags=23%, list=16%, signal=27% |
| 302 | GANGLIOSIDE\_METABOLIC\_PROCESS(GO:0001573) |  | 16 | -0.49 | -1.40 | 0.000 | 0.486 | 1.000 | 99 | tags=6%, list=0%, signal=6% |
| 303 | PRIMARY\_NEURAL\_TUBE\_FORMATION(GO:0014020) |  | 15 | -0.48 | -1.40 | 0.098 | 0.484 | 1.000 | 894 | tags=7%, list=3%, signal=7% |
| 304 | PROTEIN\_CATABOLIC\_PROCESS(GO:0030163) |  | 501 | -0.21 | -1.40 | 0.000 | 0.496 | 1.000 | 4285 | tags=11%, list=13%, signal=13% |
| 305 | REGULATION\_OF\_SODIUM\_ION\_TRANSPORT(GO:0002028) |  | 39 | -0.40 | -1.40 | 0.050 | 0.498 | 1.000 | 6295 | tags=33%, list=20%, signal=41% |
| 306 | SMALL\_GTPASE\_BINDING(GO:0031267) |  | 16 | -0.50 | -1.40 | 0.064 | 0.502 | 1.000 | 2317 | tags=31%, list=7%, signal=34% |
| 307 | HEART\_VALVE\_MORPHOGENESIS(GO:0003179) |  | 19 | -0.38 | -1.40 | 0.069 | 0.501 | 1.000 | 5013 | tags=21%, list=16%, signal=25% |
| 308 | CARBOHYDRATE\_BIOSYNTHETIC\_PROCESS(GO:0016051) |  | 94 | -0.27 | -1.39 | 0.000 | 0.503 | 1.000 | 5494 | tags=24%, list=17%, signal=29% |
| 309 | PEPTIDYL\_THREONINE\_MODIFICATION(GO:0018210) |  | 25 | -0.46 | -1.39 | 0.161 | 0.502 | 1.000 | 1638 | tags=12%, list=5%, signal=13% |
| 310 | REGULATION\_OF\_UBIQUITIN\_DEPENDENT\_PROTEIN\_CATABOLIC\_PROCESS(GO:2000058) |  | 43 | -0.31 | -1.39 | 0.056 | 0.504 | 1.000 | 3960 | tags=14%, list=12%, signal=16% |
| 311 | POSITIVE\_REGULATION\_OF\_ANGIOGENESIS(GO:0045766) |  | 40 | -0.35 | -1.39 | 0.058 | 0.504 | 1.000 | 4939 | tags=23%, list=15%, signal=27% |
| 312 | CARDIAC\_MYOFIBRIL\_ASSEMBLY(GO:0055003) |  | 17 | -0.49 | -1.39 | 0.129 | 0.504 | 1.000 | 6492 | tags=47%, list=20%, signal=59% |
| 313 | CELLULAR\_RESPONSE\_TO\_MISFOLDED\_PROTEIN(GO:0071218) |  | 15 | -0.42 | -1.39 | 0.208 | 0.504 | 1.000 | 120 | tags=7%, list=0%, signal=7% |
| 314 | RESPONSE\_TO\_MISFOLDED\_PROTEIN(GO:0051788) |  | 15 | -0.42 | -1.39 | 0.208 | 0.503 | 1.000 | 120 | tags=7%, list=0%, signal=7% |
| 315 | NUCLEOLUS(GO:0005730) |  | 228 | -0.28 | -1.39 | 0.104 | 0.505 | 1.000 | 9214 | tags=31%, list=29%, signal=43% |
| 316 | NEURON\_PROJECTION\_MEMBRANE(GO:0032589) |  | 24 | -0.46 | -1.39 | 0.101 | 0.504 | 1.000 | 5629 | tags=29%, list=18%, signal=35% |
| 317 | VACUOLAR\_PART(GO:0044437) |  | 142 | -0.28 | -1.39 | 0.000 | 0.503 | 1.000 | 8796 | tags=28%, list=28%, signal=39% |
| 318 | RRNA\_METABOLIC\_PROCESS(GO:0016072) |  | 164 | -0.33 | -1.39 | 0.159 | 0.502 | 1.000 | 9140 | tags=37%, list=29%, signal=52% |
| 319 | GUANOSINE\_CONTAINING\_COMPOUND\_METABOLIC\_PROCESS(GO:1901068) |  | 23 | -0.39 | -1.39 | 0.093 | 0.502 | 1.000 | 7211 | tags=35%, list=23%, signal=45% |
| 320 | OPTIC\_CUP\_MORPHOGENESIS\_INVOLVED\_IN\_CAMERA\_TYPE\_EYE\_DEVELOPMENT(GO:0002072) |  | 24 | -0.43 | -1.39 | 0.099 | 0.504 | 1.000 | 10972 | tags=46%, list=34%, signal=70% |
| 321 | DEVELOPMENT\_OF\_PRIMARY\_SEXUAL\_CHARACTERISTICS(GO:0045137) |  | 17 | -0.44 | -1.39 | 0.068 | 0.503 | 1.000 | 877 | tags=12%, list=3%, signal=12% |
| 322 | ESTABLISHMENT\_OF\_PIGMENT\_GRANULE\_LOCALIZATION(GO:0051905) |  | 31 | -0.42 | -1.39 | 0.031 | 0.503 | 1.000 | 3047 | tags=23%, list=10%, signal=25% |
| 323 | MUSCLE\_STRUCTURE\_DEVELOPMENT(GO:0061061) |  | 370 | -0.24 | -1.38 | 0.090 | 0.508 | 1.000 | 6330 | tags=19%, list=20%, signal=24% |
| 324 | APICAL\_PART\_OF\_CELL(GO:0045177) |  | 94 | -0.29 | -1.38 | 0.024 | 0.511 | 1.000 | 3684 | tags=15%, list=12%, signal=17% |
| 325 | PERINUCLEAR\_REGION\_OF\_CYTOPLASM(GO:0048471) |  | 112 | -0.28 | -1.38 | 0.092 | 0.510 | 1.000 | 3827 | tags=11%, list=12%, signal=12% |
| 326 | PROTEIN\_PROCESSING(GO:0016485) |  | 87 | -0.33 | -1.38 | 0.069 | 0.515 | 1.000 | 4482 | tags=17%, list=14%, signal=20% |
| 327 | SKELETAL\_MUSCLE\_ORGAN\_DEVELOPMENT(GO:0060538) |  | 141 | -0.26 | -1.38 | 0.071 | 0.517 | 1.000 | 1481 | tags=6%, list=5%, signal=7% |
| 328 | MECHANOSENSORY\_LATERAL\_LINE\_SYSTEM\_DEVELOPMENT(GO:0048881) |  | 79 | -0.25 | -1.38 | 0.031 | 0.519 | 1.000 | 3852 | tags=15%, list=12%, signal=17% |
| 329 | CYTOPLASMIC\_TRANSLATION(GO:0002181) |  | 71 | -0.34 | -1.38 | 0.219 | 0.522 | 1.000 | 10745 | tags=44%, list=34%, signal=66% |
| 330 | MITOCHONDRIAL\_RESPIRATORY\_CHAIN\_COMPLEX\_ASSEMBLY(GO:0033108) |  | 49 | -0.40 | -1.38 | 0.213 | 0.521 | 1.000 | 4267 | tags=14%, list=13%, signal=16% |
| 331 | MITOCHONDRIAL\_MATRIX(GO:0005759) |  | 144 | -0.27 | -1.38 | 0.248 | 0.521 | 1.000 | 2934 | tags=10%, list=9%, signal=11% |
| 332 | PROTEIN\_SUMOYLATION(GO:0016925) |  | 17 | -0.47 | -1.38 | 0.000 | 0.521 | 1.000 | 177 | tags=6%, list=1%, signal=6% |
| 333 | RIBOSOME\_BIOGENESIS(GO:0042254) |  | 218 | -0.33 | -1.38 | 0.221 | 0.520 | 1.000 | 9140 | tags=37%, list=29%, signal=52% |
| 334 | STRIATED\_MUSCLE\_CELL\_DEVELOPMENT(GO:0055002) |  | 165 | -0.26 | -1.37 | 0.128 | 0.523 | 1.000 | 6852 | tags=25%, list=21%, signal=31% |
| 335 | REGULATION\_OF\_EPITHELIAL\_CELL\_MIGRATION(GO:0010632) |  | 25 | -0.44 | -1.37 | 0.238 | 0.523 | 1.000 | 6528 | tags=36%, list=20%, signal=45% |
| 336 | SWIMMING\_BEHAVIOR(GO:0036269) |  | 28 | -0.40 | -1.37 | 0.014 | 0.522 | 1.000 | 7946 | tags=36%, list=25%, signal=48% |
| 337 | G\_PROTEIN\_BETA\_GAMMA\_SUBUNIT\_COMPLEX\_BINDING(GO:0031683) |  | 23 | -0.39 | -1.37 | 0.108 | 0.526 | 1.000 | 3378 | tags=13%, list=11%, signal=15% |
| 338 | MOLECULAR\_ADAPTOR\_ACTIVITY(GO:0060090) |  | 113 | -0.31 | -1.37 | 0.072 | 0.526 | 1.000 | 4228 | tags=15%, list=13%, signal=17% |
| 339 | EXONUCLEASE\_ACTIVITY(GO:0004527) |  | 60 | -0.30 | -1.37 | 0.105 | 0.527 | 1.000 | 5829 | tags=18%, list=18%, signal=22% |
| 340 | DEVELOPMENTAL\_GROWTH(GO:0048589) |  | 361 | -0.24 | -1.37 | 0.041 | 0.527 | 1.000 | 5841 | tags=17%, list=18%, signal=20% |
| 341 | EPITHELIAL\_CILIUM\_MOVEMENT\_INVOLVED\_IN\_DETERMINATION\_OF\_LEFT\_RIGHT\_ASYMMETRY(GO:0060287) |  | 17 | -0.56 | -1.37 | 0.075 | 0.526 | 1.000 | 8185 | tags=47%, list=26%, signal=63% |
| 342 | CELLULAR\_RESPONSE\_TO\_ENDOGENOUS\_STIMULUS(GO:0071495) |  | 430 | -0.25 | -1.37 | 0.000 | 0.527 | 1.000 | 3160 | tags=10%, list=10%, signal=11% |
| 343 | REGULATION\_OF\_INSULIN\_SECRETION(GO:0050796) |  | 18 | -0.45 | -1.37 | 0.061 | 0.526 | 1.000 | 4587 | tags=22%, list=14%, signal=26% |
| 344 | DOPAMINE\_RECEPTOR\_SIGNALING\_PATHWAY(GO:0007212) |  | 27 | -0.45 | -1.37 | 0.000 | 0.528 | 1.000 | 4340 | tags=26%, list=14%, signal=30% |
| 345 | CARDIAC\_MUSCLE\_CELL\_ACTION\_POTENTIAL(GO:0086001) |  | 17 | -0.48 | -1.37 | 0.070 | 0.530 | 1.000 | 4160 | tags=29%, list=13%, signal=34% |
| 346 | MULTICELLULAR\_ORGANISMAL\_MOVEMENT(GO:0050879) |  | 31 | -0.43 | -1.36 | 0.060 | 0.531 | 1.000 | 5189 | tags=39%, list=16%, signal=46% |
| 347 | MUSCULOSKELETAL\_MOVEMENT(GO:0050881) |  | 31 | -0.43 | -1.36 | 0.060 | 0.530 | 1.000 | 5189 | tags=39%, list=16%, signal=46% |
| 348 | SMALL\_GTPASE\_MEDIATED\_SIGNAL\_TRANSDUCTION(GO:0007264) |  | 290 | -0.26 | -1.36 | 0.060 | 0.528 | 1.000 | 5420 | tags=18%, list=17%, signal=21% |
| 349 | RRNA\_MODIFICATION(GO:0000154) |  | 25 | -0.37 | -1.36 | 0.059 | 0.528 | 1.000 | 12800 | tags=60%, list=40%, signal=100% |
| 350 | TRANSCRIPTION\_INITIATION\_FROM\_RNA\_POLYMERASE\_II\_PROMOTER(GO:0006367) |  | 30 | -0.39 | -1.36 | 0.035 | 0.528 | 1.000 | 5427 | tags=27%, list=17%, signal=32% |
| 351 | REGULATION\_OF\_HEMATOPOIETIC\_STEM\_CELL\_DIFFERENTIATION(GO:1902036) |  | 28 | -0.40 | -1.36 | 0.131 | 0.527 | 1.000 | 6090 | tags=32%, list=19%, signal=40% |
| 352 | GROWTH(GO:0040007) |  | 403 | -0.23 | -1.36 | 0.041 | 0.528 | 1.000 | 5841 | tags=17%, list=18%, signal=20% |
| 353 | STRIATED\_MUSCLE\_CELL\_DIFFERENTIATION(GO:0051146) |  | 195 | -0.26 | -1.36 | 0.079 | 0.527 | 1.000 | 8014 | tags=29%, list=25%, signal=38% |
| 354 | DEFINITIVE\_HEMOPOIESIS(GO:0060216) |  | 61 | -0.31 | -1.36 | 0.165 | 0.528 | 1.000 | 5889 | tags=21%, list=18%, signal=26% |
| 355 | CLOSURE\_OF\_OPTIC\_FISSURE(GO:0061386) |  | 21 | -0.43 | -1.36 | 0.185 | 0.528 | 1.000 | 10955 | tags=43%, list=34%, signal=65% |
| 356 | METHYLATED\_HISTONE\_BINDING(GO:0035064) |  | 23 | -0.37 | -1.36 | 0.172 | 0.527 | 1.000 | 2961 | tags=9%, list=9%, signal=10% |
| 357 | METHYLATION\_DEPENDENT\_PROTEIN\_BINDING(GO:0140034) |  | 23 | -0.37 | -1.36 | 0.172 | 0.525 | 1.000 | 2961 | tags=9%, list=9%, signal=10% |
| 358 | POSITIVE\_REGULATION\_OF\_DNA\_REPAIR(GO:0045739) |  | 25 | -0.32 | -1.36 | 0.110 | 0.525 | 1.000 | 977 | tags=8%, list=3%, signal=8% |
| 359 | NEGATIVE\_REGULATION\_OF\_CELL\_PROLIFERATION(GO:0008285) |  | 88 | -0.28 | -1.36 | 0.000 | 0.523 | 1.000 | 3763 | tags=16%, list=12%, signal=18% |
| 360 | MUSCLE\_ORGAN\_DEVELOPMENT(GO:0007517) |  | 195 | -0.26 | -1.36 | 0.122 | 0.526 | 1.000 | 1481 | tags=6%, list=5%, signal=6% |
| 361 | REGULATION\_OF\_SPROUTING\_ANGIOGENESIS(GO:1903670) |  | 28 | -0.44 | -1.36 | 0.160 | 0.525 | 1.000 | 6528 | tags=36%, list=20%, signal=45% |
| 362 | VACUOLAR\_MEMBRANE(GO:0005774) |  | 140 | -0.27 | -1.36 | 0.047 | 0.523 | 1.000 | 8796 | tags=28%, list=28%, signal=38% |
| 363 | CULLIN\_RING\_UBIQUITIN\_LIGASE\_COMPLEX(GO:0031461) |  | 125 | -0.30 | -1.36 | 0.000 | 0.522 | 1.000 | 3988 | tags=17%, list=13%, signal=19% |
| 364 | MAINTENANCE\_OF\_LOCATION\_IN\_CELL(GO:0051651) |  | 31 | -0.41 | -1.36 | 0.000 | 0.522 | 1.000 | 2578 | tags=19%, list=8%, signal=21% |
| 365 | CELLULAR\_PIGMENTATION(GO:0033059) |  | 52 | -0.37 | -1.36 | 0.000 | 0.524 | 1.000 | 8205 | tags=40%, list=26%, signal=54% |
| 366 | GTP\_METABOLIC\_PROCESS(GO:0046039) |  | 17 | -0.45 | -1.36 | 0.091 | 0.524 | 1.000 | 8814 | tags=41%, list=28%, signal=57% |
| 367 | SKELETAL\_MUSCLE\_CONTRACTION(GO:0003009) |  | 28 | -0.45 | -1.36 | 0.126 | 0.523 | 1.000 | 5189 | tags=43%, list=16%, signal=51% |
| 368 | CORTICAL\_CYTOSKELETON\_ORGANIZATION(GO:0030865) |  | 56 | -0.29 | -1.36 | 0.000 | 0.522 | 1.000 | 6562 | tags=25%, list=21%, signal=31% |
| 369 | UBIQUITIN\_LIGASE\_COMPLEX(GO:0000151) |  | 201 | -0.27 | -1.36 | 0.000 | 0.522 | 1.000 | 5919 | tags=20%, list=19%, signal=25% |
| 370 | NUCLEOSIDE\_TRIPHOSPHATASE\_ACTIVITY(GO:0017111) |  | 750 | -0.20 | -1.36 | 0.000 | 0.523 | 1.000 | 4556 | tags=13%, list=14%, signal=14% |
| 371 | MACROPHAGE\_CHEMOTAXIS(GO:0048246) |  | 16 | -0.36 | -1.36 | 0.075 | 0.522 | 1.000 | 4402 | tags=19%, list=14%, signal=22% |
| 372 | FERROUS\_IRON\_BINDING(GO:0008198) |  | 24 | -0.44 | -1.36 | 0.103 | 0.522 | 1.000 | 5375 | tags=29%, list=17%, signal=35% |
| 373 | MACROPHAGE\_MIGRATION(GO:1905517) |  | 19 | -0.37 | -1.35 | 0.016 | 0.525 | 1.000 | 4402 | tags=21%, list=14%, signal=24% |
| 374 | CHAPERONE\_COFACTOR\_DEPENDENT\_PROTEIN\_REFOLDING(GO:0051085) |  | 27 | -0.41 | -1.35 | 0.032 | 0.526 | 1.000 | 3912 | tags=22%, list=12%, signal=25% |
| 375 | BONE\_DEVELOPMENT(GO:0060348) |  | 57 | -0.30 | -1.35 | 0.047 | 0.525 | 1.000 | 6528 | tags=25%, list=20%, signal=31% |
| 376 | ANAPHASE\_PROMOTING\_COMPLEX\_DEPENDENT\_CATABOLIC\_PROCESS(GO:0031145) |  | 17 | -0.44 | -1.35 | 0.193 | 0.524 | 1.000 | 2255 | tags=12%, list=7%, signal=13% |
| 377 | REGULATION\_OF\_NEUTROPHIL\_MIGRATION(GO:1902622) |  | 19 | -0.41 | -1.35 | 0.104 | 0.523 | 1.000 | 5120 | tags=21%, list=16%, signal=25% |
| 378 | PROTEIN\_CONTAINING\_COMPLEX\_SUBUNIT\_ORGANIZATION(GO:0043933) |  | 903 | -0.21 | -1.35 | 0.000 | 0.527 | 1.000 | 5265 | tags=14%, list=17%, signal=16% |
| 379 | NEUROMAST\_HAIR\_CELL\_DIFFERENTIATION(GO:0048886) |  | 23 | -0.34 | -1.35 | 0.081 | 0.527 | 1.000 | 1842 | tags=9%, list=6%, signal=9% |
| 380 | REGULATION\_OF\_RECEPTOR\_MEDIATED\_ENDOCYTOSIS(GO:0048259) |  | 18 | -0.34 | -1.35 | 0.061 | 0.526 | 1.000 | 4549 | tags=17%, list=14%, signal=19% |
| 381 | CELLULAR\_MACROMOLECULE\_CATABOLIC\_PROCESS(GO:0044265) |  | 611 | -0.21 | -1.35 | 0.000 | 0.525 | 1.000 | 4285 | tags=12%, list=13%, signal=13% |
| 382 | NEURAL\_TUBE\_FORMATION(GO:0001841) |  | 32 | -0.36 | -1.35 | 0.163 | 0.524 | 1.000 | 894 | tags=6%, list=3%, signal=6% |
| 383 | TROPONIN\_COMPLEX(GO:0005861) |  | 24 | -0.58 | -1.35 | 0.170 | 0.524 | 1.000 | 6330 | tags=58%, list=20%, signal=73% |
| 384 | ENDOCHONDRAL\_BONE\_MORPHOGENESIS(GO:0060350) |  | 21 | -0.34 | -1.35 | 0.122 | 0.522 | 1.000 | 6217 | tags=24%, list=19%, signal=30% |
| 385 | REPLACEMENT\_BONE\_MORPHOGENESIS(GO:0061971) |  | 21 | -0.34 | -1.35 | 0.122 | 0.521 | 1.000 | 6217 | tags=24%, list=19%, signal=30% |
| 386 | REGULATION\_OF\_TRANSCRIPTION\_ELONGATION\_FROM\_RNA\_POLYMERASE\_II\_PROMOTER(GO:0034243) |  | 19 | -0.33 | -1.35 | 0.136 | 0.520 | 1.000 | 7956 | tags=42%, list=25%, signal=56% |
| 387 | ENDODERMAL\_CELL\_FATE\_SPECIFICATION(GO:0001714) |  | 22 | -0.47 | -1.35 | 0.079 | 0.521 | 1.000 | 5220 | tags=23%, list=16%, signal=27% |
| 388 | COENZYME\_BINDING(GO:0050662) |  | 246 | -0.23 | -1.35 | 0.016 | 0.520 | 1.000 | 4702 | tags=17%, list=15%, signal=19% |
| 389 | POTASSIUM\_ION\_IMPORT(GO:0010107) |  | 60 | -0.37 | -1.35 | 0.089 | 0.521 | 1.000 | 3047 | tags=15%, list=10%, signal=17% |
| 390 | MUSCLE\_CONTRACTION(GO:0006936) |  | 112 | -0.32 | -1.35 | 0.094 | 0.520 | 1.000 | 6788 | tags=32%, list=21%, signal=41% |
| 391 | HYDROLASE\_ACTIVITY\_\_ACTING\_ON\_CARBON\_NITROGEN\_(BUT\_NOT\_PEPTIDE)\_BONDS\_\_IN\_LINEAR\_AMIDES(GO:0016811) |  | 58 | -0.31 | -1.35 | 0.048 | 0.520 | 1.000 | 5489 | tags=26%, list=17%, signal=31% |
| 392 | INTRACILIARY\_TRANSPORT\_PARTICLE(GO:0030990) |  | 19 | -0.47 | -1.35 | 0.070 | 0.522 | 1.000 | 9762 | tags=58%, list=31%, signal=83% |
| 393 | PURINE\_NUCLEOSIDE\_BIOSYNTHETIC\_PROCESS(GO:0042451) |  | 26 | -0.44 | -1.34 | 0.090 | 0.524 | 1.000 | 8814 | tags=50%, list=28%, signal=69% |
| 394 | PURINE\_RIBONUCLEOSIDE\_BIOSYNTHETIC\_PROCESS(GO:0046129) |  | 26 | -0.44 | -1.34 | 0.090 | 0.522 | 1.000 | 8814 | tags=50%, list=28%, signal=69% |
| 395 | REGULATION\_OF\_FIBROBLAST\_GROWTH\_FACTOR\_RECEPTOR\_SIGNALING\_PATHWAY(GO:0040036) |  | 24 | -0.36 | -1.34 | 0.093 | 0.522 | 1.000 | 2562 | tags=17%, list=8%, signal=18% |
| 396 | PIGMENT\_GRANULE\_LOCALIZATION(GO:0051875) |  | 34 | -0.39 | -1.34 | 0.021 | 0.532 | 1.000 | 3047 | tags=21%, list=10%, signal=23% |
| 397 | HYDROLASE\_ACTIVITY\_\_ACTING\_ON\_ACID\_ANHYDRIDES(GO:0016817) |  | 807 | -0.20 | -1.34 | 0.000 | 0.532 | 1.000 | 4996 | tags=14%, list=16%, signal=16% |
| 398 | HYDROLASE\_ACTIVITY\_\_ACTING\_ON\_ACID\_ANHYDRIDES\_\_IN\_PHOSPHORUS\_CONTAINING\_ANHYDRIDES(GO:0016818) |  | 807 | -0.20 | -1.34 | 0.000 | 0.531 | 1.000 | 4996 | tags=14%, list=16%, signal=16% |
| 399 | PROTEIN\_POLYMERIZATION(GO:0051258) |  | 174 | -0.23 | -1.34 | 0.047 | 0.530 | 1.000 | 3728 | tags=12%, list=12%, signal=14% |
| 400 | CIRCULATORY\_SYSTEM\_PROCESS(GO:0003013) |  | 243 | -0.26 | -1.34 | 0.067 | 0.529 | 1.000 | 6334 | tags=22%, list=20%, signal=27% |
| 401 | PYROPHOSPHATASE\_ACTIVITY(GO:0016462) |  | 804 | -0.20 | -1.34 | 0.000 | 0.530 | 1.000 | 4996 | tags=14%, list=16%, signal=16% |
| 402 | PEPTIDE\_HORMONE\_SECRETION(GO:0030072) |  | 30 | -0.40 | -1.34 | 0.169 | 0.532 | 1.000 | 4587 | tags=20%, list=14%, signal=23% |
| 403 | REGULATION\_OF\_ENDOTHELIAL\_CELL\_MIGRATION(GO:0010594) |  | 21 | -0.45 | -1.34 | 0.152 | 0.534 | 1.000 | 6528 | tags=38%, list=20%, signal=48% |
| 404 | MUSCLE\_SYSTEM\_PROCESS(GO:0003012) |  | 119 | -0.31 | -1.34 | 0.043 | 0.535 | 1.000 | 7157 | tags=32%, list=22%, signal=41% |
| 405 | TELOMERE\_MAINTENANCE\_VIA\_TELOMERASE(GO:0007004) |  | 17 | -0.47 | -1.33 | 0.158 | 0.539 | 1.000 | 51 | tags=6%, list=0%, signal=6% |
| 406 | RNA\_DEPENDENT\_DNA\_BIOSYNTHETIC\_PROCESS(GO:0006278) |  | 17 | -0.47 | -1.33 | 0.158 | 0.537 | 1.000 | 51 | tags=6%, list=0%, signal=6% |
| 407 | AUTOPHAGOSOME\_ORGANIZATION(GO:1905037) |  | 56 | -0.31 | -1.33 | 0.093 | 0.538 | 1.000 | 4996 | tags=20%, list=16%, signal=23% |
| 408 | CELL\_ACTIVATION(GO:0001775) |  | 184 | -0.24 | -1.33 | 0.045 | 0.538 | 1.000 | 2416 | tags=11%, list=8%, signal=12% |
| 409 | CARBOHYDRATE\_CATABOLIC\_PROCESS(GO:0016052) |  | 67 | -0.40 | -1.33 | 0.139 | 0.537 | 1.000 | 6371 | tags=33%, list=20%, signal=41% |
| 410 | ATPASE\_ACTIVITY(GO:0016887) |  | 248 | -0.21 | -1.33 | 0.000 | 0.539 | 1.000 | 2382 | tags=8%, list=7%, signal=8% |
| 411 | ENDOCYTIC\_VESICLE(GO:0030139) |  | 51 | -0.28 | -1.33 | 0.112 | 0.538 | 1.000 | 2408 | tags=10%, list=8%, signal=11% |
| 412 | NEGATIVE\_REGULATION\_OF\_NUCLEAR\_DIVISION(GO:0051784) |  | 26 | -0.38 | -1.33 | 0.128 | 0.539 | 1.000 | 1574 | tags=12%, list=5%, signal=12% |
| 413 | MANNOSYLTRANSFERASE\_ACTIVITY(GO:0000030) |  | 20 | -0.41 | -1.33 | 0.181 | 0.539 | 1.000 | 1693 | tags=10%, list=5%, signal=11% |
| 414 | REGULATION\_OF\_CATION\_TRANSMEMBRANE\_TRANSPORT(GO:1904062) |  | 102 | -0.34 | -1.33 | 0.080 | 0.538 | 1.000 | 5936 | tags=29%, list=19%, signal=36% |
| 415 | POLYAMINE\_METABOLIC\_PROCESS(GO:0006595) |  | 16 | -0.54 | -1.33 | 0.126 | 0.537 | 1.000 | 5728 | tags=44%, list=18%, signal=53% |
| 416 | REGULATION\_OF\_ANATOMICAL\_STRUCTURE\_MORPHOGENESIS(GO:0022603) |  | 401 | -0.23 | -1.33 | 0.054 | 0.536 | 1.000 | 4471 | tags=13%, list=14%, signal=15% |
| 417 | ONE\_CARBON\_METABOLIC\_PROCESS(GO:0006730) |  | 39 | -0.36 | -1.33 | 0.151 | 0.536 | 1.000 | 4087 | tags=15%, list=13%, signal=18% |
| 418 | CHAPERONE\_BINDING(GO:0051087) |  | 38 | -0.36 | -1.33 | 0.155 | 0.537 | 1.000 | 8367 | tags=32%, list=26%, signal=43% |
| 419 | GOLGI\_ASSOCIATED\_VESICLE\_MEMBRANE(GO:0030660) |  | 50 | -0.27 | -1.33 | 0.102 | 0.537 | 1.000 | 3643 | tags=14%, list=11%, signal=16% |
| 420 | REGULATION\_OF\_TORC1\_SIGNALING(GO:1903432) |  | 20 | -0.47 | -1.33 | 0.188 | 0.536 | 1.000 | 7547 | tags=30%, list=24%, signal=39% |
| 421 | LYSINE\_N\_METHYLTRANSFERASE\_ACTIVITY(GO:0016278) |  | 51 | -0.30 | -1.33 | 0.081 | 0.537 | 1.000 | 1027 | tags=6%, list=3%, signal=6% |
| 422 | PROTEIN\_LYSINE\_N\_METHYLTRANSFERASE\_ACTIVITY(GO:0016279) |  | 51 | -0.30 | -1.33 | 0.081 | 0.536 | 1.000 | 1027 | tags=6%, list=3%, signal=6% |
| 423 | TELOMERE\_MAINTENANCE\_VIA\_TELOMERE\_LENGTHENING(GO:0010833) |  | 22 | -0.41 | -1.33 | 0.137 | 0.541 | 1.000 | 51 | tags=5%, list=0%, signal=5% |
| 424 | PROTEIN\_SERINE\_THREONINE\_PHOSPHATASE\_COMPLEX(GO:0008287) |  | 45 | -0.29 | -1.33 | 0.085 | 0.544 | 1.000 | 4125 | tags=16%, list=13%, signal=18% |
| 425 | PHOSPHATASE\_COMPLEX(GO:1903293) |  | 45 | -0.29 | -1.33 | 0.085 | 0.542 | 1.000 | 4125 | tags=16%, list=13%, signal=18% |
| 426 | COATED\_VESICLE\_MEMBRANE(GO:0030662) |  | 65 | -0.23 | -1.32 | 0.064 | 0.541 | 1.000 | 3643 | tags=12%, list=11%, signal=14% |
| 427 | ENDODERMAL\_CELL\_FATE\_COMMITMENT(GO:0001711) |  | 23 | -0.45 | -1.32 | 0.079 | 0.541 | 1.000 | 5220 | tags=22%, list=16%, signal=26% |
| 428 | HEART\_CONTRACTION(GO:0060047) |  | 153 | -0.27 | -1.32 | 0.034 | 0.540 | 1.000 | 6865 | tags=25%, list=22%, signal=32% |
| 429 | MICROTUBULE\_BASED\_TRANSPORT(GO:0099111) |  | 71 | -0.32 | -1.32 | 0.164 | 0.554 | 1.000 | 3518 | tags=15%, list=11%, signal=17% |
| 430 | POSITIVE\_REGULATION\_OF\_SPROUTING\_ANGIOGENESIS(GO:1903672) |  | 16 | -0.48 | -1.32 | 0.136 | 0.553 | 1.000 | 6089 | tags=44%, list=19%, signal=54% |
| 431 | REGULATION\_OF\_PROTEIN\_STABILITY(GO:0031647) |  | 37 | -0.36 | -1.32 | 0.029 | 0.552 | 1.000 | 10092 | tags=41%, list=32%, signal=59% |
| 432 | NEGATIVE\_REGULATION\_OF\_CELL\_CYCLE\_PHASE\_TRANSITION(GO:1901988) |  | 49 | -0.33 | -1.32 | 0.139 | 0.555 | 1.000 | 1565 | tags=8%, list=5%, signal=9% |
| 433 | NEGATIVE\_REGULATION\_OF\_MITOTIC\_CELL\_CYCLE\_PHASE\_TRANSITION(GO:1901991) |  | 49 | -0.33 | -1.32 | 0.139 | 0.554 | 1.000 | 1565 | tags=8%, list=5%, signal=9% |
| 434 | ENDOSOME\_TRANSPORT\_VIA\_MULTIVESICULAR\_BODY\_SORTING\_PATHWAY(GO:0032509) |  | 24 | -0.32 | -1.32 | 0.029 | 0.554 | 1.000 | 120 | tags=4%, list=0%, signal=4% |
| 435 | MULTIVESICULAR\_BODY\_SORTING\_PATHWAY(GO:0071985) |  | 24 | -0.32 | -1.32 | 0.029 | 0.553 | 1.000 | 120 | tags=4%, list=0%, signal=4% |
| 436 | POSITIVE\_REGULATION\_OF\_TRANSCRIPTION\_BY\_RNA\_POLYMERASE\_II(GO:0045944) |  | 307 | -0.23 | -1.32 | 0.068 | 0.554 | 1.000 | 4652 | tags=14%, list=15%, signal=17% |
| 437 | REGULATION\_OF\_PROTEIN\_COMPLEX\_ASSEMBLY(GO:0043254) |  | 171 | -0.24 | -1.32 | 0.017 | 0.552 | 1.000 | 5213 | tags=16%, list=16%, signal=19% |
| 438 | CALCIUM\_DEPENDENT\_CYSTEINE\_TYPE\_ENDOPEPTIDASE\_ACTIVITY(GO:0004198) |  | 22 | -0.42 | -1.31 | 0.131 | 0.559 | 1.000 | 8883 | tags=55%, list=28%, signal=76% |
| 439 | PHOSPHATIDYLINOSITOL\_4\_5\_BISPHOSPHATE\_BINDING(GO:0005546) |  | 31 | -0.36 | -1.31 | 0.035 | 0.558 | 1.000 | 3663 | tags=16%, list=11%, signal=18% |
| 440 | REGULATION\_OF\_LOCOMOTION(GO:0040012) |  | 253 | -0.23 | -1.31 | 0.000 | 0.559 | 1.000 | 5120 | tags=16%, list=16%, signal=19% |
| 441 | NEGATIVE\_REGULATION\_OF\_SISTER\_CHROMATID\_SEGREGATION(GO:0033046) |  | 18 | -0.44 | -1.31 | 0.153 | 0.558 | 1.000 | 1574 | tags=11%, list=5%, signal=12% |
| 442 | NEGATIVE\_REGULATION\_OF\_MITOTIC\_SISTER\_CHROMATID\_SEGREGATION(GO:0033048) |  | 18 | -0.44 | -1.31 | 0.153 | 0.557 | 1.000 | 1574 | tags=11%, list=5%, signal=12% |
| 443 | NEGATIVE\_REGULATION\_OF\_CHROMOSOME\_SEGREGATION(GO:0051985) |  | 18 | -0.44 | -1.31 | 0.153 | 0.556 | 1.000 | 1574 | tags=11%, list=5%, signal=12% |
| 444 | NEGATIVE\_REGULATION\_OF\_CHROMOSOME\_SEPARATION(GO:1905819) |  | 18 | -0.44 | -1.31 | 0.153 | 0.555 | 1.000 | 1574 | tags=11%, list=5%, signal=12% |
| 445 | NEGATIVE\_REGULATION\_OF\_MITOTIC\_SISTER\_CHROMATID\_SEPARATION(GO:2000816) |  | 18 | -0.44 | -1.31 | 0.153 | 0.553 | 1.000 | 1574 | tags=11%, list=5%, signal=12% |
| 446 | NADH\_DEHYDROGENASE\_(UBIQUINONE)\_ACTIVITY(GO:0008137) |  | 20 | -0.45 | -1.31 | 0.183 | 0.556 | 1.000 | 9722 | tags=35%, list=30%, signal=50% |
| 447 | NADH\_DEHYDROGENASE\_(QUINONE)\_ACTIVITY(GO:0050136) |  | 20 | -0.45 | -1.31 | 0.183 | 0.555 | 1.000 | 9722 | tags=35%, list=30%, signal=50% |
| 448 | MUSCLE\_CELL\_DIFFERENTIATION(GO:0042692) |  | 248 | -0.25 | -1.31 | 0.192 | 0.555 | 1.000 | 8014 | tags=27%, list=25%, signal=36% |
| 449 | PHOSPHATIDYLINOSITOL\_BISPHOSPHATE\_BINDING(GO:1902936) |  | 46 | -0.29 | -1.31 | 0.000 | 0.556 | 1.000 | 6270 | tags=17%, list=20%, signal=22% |
| 450 | BONE\_MORPHOGENESIS(GO:0060349) |  | 33 | -0.34 | -1.31 | 0.142 | 0.557 | 1.000 | 6528 | tags=24%, list=20%, signal=30% |
| 451 | ACTIN\_FILAMENT\_BASED\_PROCESS(GO:0030029) |  | 548 | -0.22 | -1.31 | 0.034 | 0.555 | 1.000 | 6852 | tags=22%, list=21%, signal=27% |
| 452 | HOMEOSTATIC\_PROCESS(GO:0042592) |  | 753 | -0.21 | -1.31 | 0.000 | 0.555 | 1.000 | 5936 | tags=18%, list=19%, signal=21% |
| 453 | NON\_RECOMBINATIONAL\_REPAIR(GO:0000726) |  | 26 | -0.38 | -1.31 | 0.069 | 0.557 | 1.000 | 179 | tags=8%, list=1%, signal=8% |
| 454 | REGULATION\_OF\_RESPONSE\_TO\_STRESS(GO:0080134) |  | 273 | -0.22 | -1.31 | 0.038 | 0.557 | 1.000 | 3305 | tags=11%, list=10%, signal=13% |
| 455 | TRANSPORT\_ALONG\_MICROTUBULE(GO:0010970) |  | 70 | -0.32 | -1.31 | 0.164 | 0.556 | 1.000 | 3518 | tags=16%, list=11%, signal=18% |
| 456 | CELLULAR\_PROTEIN\_COMPLEX\_DISASSEMBLY(GO:0043624) |  | 96 | -0.25 | -1.31 | 0.060 | 0.555 | 1.000 | 4854 | tags=16%, list=15%, signal=18% |
| 457 | INTRINSIC\_APOPTOTIC\_SIGNALING\_PATHWAY\_IN\_RESPONSE\_TO\_DNA\_DAMAGE(GO:0008630) |  | 38 | -0.32 | -1.31 | 0.141 | 0.554 | 1.000 | 5229 | tags=26%, list=16%, signal=31% |
| 458 | ACTOMYOSIN\_STRUCTURE\_ORGANIZATION(GO:0031032) |  | 133 | -0.28 | -1.31 | 0.108 | 0.556 | 1.000 | 6852 | tags=28%, list=21%, signal=35% |
| 459 | SIGNAL\_TRANSDUCTION\_BY\_P53\_CLASS\_MEDIATOR(GO:0072331) |  | 36 | -0.32 | -1.30 | 0.058 | 0.559 | 1.000 | 5118 | tags=28%, list=16%, signal=33% |
| 460 | MITOCHONDRIAL\_OUTER\_MEMBRANE(GO:0005741) |  | 96 | -0.26 | -1.30 | 0.224 | 0.561 | 1.000 | 6389 | tags=21%, list=20%, signal=26% |
| 461 | NEGATIVE\_REGULATION\_OF\_PROTEIN\_UBIQUITINATION(GO:0031397) |  | 16 | -0.46 | -1.30 | 0.198 | 0.561 | 1.000 | 10563 | tags=56%, list=33%, signal=84% |
| 462 | REGULATION\_OF\_CALCIUM\_ION\_TRANSMEMBRANE\_TRANSPORT(GO:1903169) |  | 34 | -0.39 | -1.30 | 0.158 | 0.559 | 1.000 | 5936 | tags=35%, list=19%, signal=43% |
| 463 | CELLULAR\_CARBOHYDRATE\_BIOSYNTHETIC\_PROCESS(GO:0034637) |  | 55 | -0.29 | -1.30 | 0.113 | 0.558 | 1.000 | 5494 | tags=31%, list=17%, signal=37% |
| 464 | NOTOCHORD\_CELL\_DIFFERENTIATION(GO:0060034) |  | 21 | -0.41 | -1.30 | 0.097 | 0.562 | 1.000 | 12022 | tags=57%, list=38%, signal=92% |
| 465 | CHOLESTEROL\_HOMEOSTASIS(GO:0042632) |  | 22 | -0.50 | -1.30 | 0.181 | 0.562 | 1.000 | 1084 | tags=14%, list=3%, signal=14% |
| 466 | STEROL\_HOMEOSTASIS(GO:0055092) |  | 22 | -0.50 | -1.30 | 0.181 | 0.561 | 1.000 | 1084 | tags=14%, list=3%, signal=14% |
| 467 | CELLULAR\_RESPONSE\_TO\_ORGANIC\_SUBSTANCE(GO:0071310) |  | 708 | -0.22 | -1.30 | 0.021 | 0.560 | 1.000 | 3170 | tags=10%, list=10%, signal=11% |
| 468 | NUCLEOSIDE\_METABOLIC\_PROCESS(GO:0009116) |  | 81 | -0.24 | -1.30 | 0.062 | 0.559 | 1.000 | 6520 | tags=25%, list=20%, signal=31% |
| 469 | NEGATIVE\_REGULATION\_OF\_CELL\_CELL\_ADHESION(GO:0022408) |  | 51 | -0.29 | -1.30 | 0.054 | 0.561 | 1.000 | 1360 | tags=14%, list=4%, signal=14% |
| 470 | ACTIN\_CYTOSKELETON\_ORGANIZATION(GO:0030036) |  | 531 | -0.22 | -1.30 | 0.056 | 0.560 | 1.000 | 6852 | tags=21%, list=21%, signal=27% |
| 471 | REGULATION\_OF\_PEPTIDE\_HORMONE\_SECRETION(GO:0090276) |  | 19 | -0.44 | -1.30 | 0.126 | 0.560 | 1.000 | 4587 | tags=21%, list=14%, signal=25% |
| 472 | PROTEIN\_PHOSPHATASE\_BINDING(GO:0019903) |  | 39 | -0.30 | -1.30 | 0.109 | 0.559 | 1.000 | 541 | tags=5%, list=2%, signal=5% |
| 473 | GRANULOCYTE\_MIGRATION(GO:0097530) |  | 84 | -0.30 | -1.30 | 0.056 | 0.562 | 1.000 | 4402 | tags=17%, list=14%, signal=19% |
| 474 | 'DE\_NOVO'\_PROTEIN\_FOLDING(GO:0006458) |  | 28 | -0.40 | -1.30 | 0.079 | 0.561 | 1.000 | 3912 | tags=21%, list=12%, signal=24% |
| 475 | 'DE\_NOVO'\_POSTTRANSLATIONAL\_PROTEIN\_FOLDING(GO:0051084) |  | 28 | -0.40 | -1.30 | 0.079 | 0.559 | 1.000 | 3912 | tags=21%, list=12%, signal=24% |
| 476 | SKIN\_DEVELOPMENT(GO:0043588) |  | 25 | -0.37 | -1.30 | 0.173 | 0.561 | 1.000 | 1287 | tags=12%, list=4%, signal=12% |
| 477 | MYELOID\_LEUKOCYTE\_MIGRATION(GO:0097529) |  | 98 | -0.27 | -1.30 | 0.016 | 0.560 | 1.000 | 4402 | tags=16%, list=14%, signal=19% |
| 478 | EXTRACELLULAR\_STRUCTURE\_ORGANIZATION(GO:0043062) |  | 180 | -0.26 | -1.30 | 0.081 | 0.559 | 1.000 | 3418 | tags=12%, list=11%, signal=13% |
| 479 | CELLULAR\_COMPONENT\_ASSEMBLY(GO:0022607) |  | 1534 | -0.19 | -1.30 | 0.000 | 0.558 | 1.000 | 6852 | tags=19%, list=21%, signal=22% |
| 480 | G\_PROTEIN\_BETA\_SUBUNIT\_BINDING(GO:0031681) |  | 16 | -0.36 | -1.30 | 0.121 | 0.560 | 1.000 | 79 | tags=6%, list=0%, signal=6% |
| 481 | NEGATIVE\_REGULATION\_OF\_PROTEIN\_MODIFICATION\_BY\_SMALL\_PROTEIN\_CONJUGATION\_OR\_REMOVAL(GO:1903321) |  | 17 | -0.45 | -1.30 | 0.142 | 0.561 | 1.000 | 10563 | tags=53%, list=33%, signal=79% |
| 482 | LIPID\_HOMEOSTASIS(GO:0055088) |  | 55 | -0.31 | -1.29 | 0.123 | 0.562 | 1.000 | 4923 | tags=16%, list=15%, signal=19% |
| 483 | APOPTOTIC\_SIGNALING\_PATHWAY(GO:0097190) |  | 132 | -0.23 | -1.29 | 0.047 | 0.561 | 1.000 | 5229 | tags=17%, list=16%, signal=21% |
| 484 | ECTODERMAL\_PLACODE\_DEVELOPMENT(GO:0071696) |  | 39 | -0.30 | -1.29 | 0.058 | 0.562 | 1.000 | 8866 | tags=31%, list=28%, signal=43% |
| 485 | REGULATION\_OF\_ANGIOGENESIS(GO:0045765) |  | 91 | -0.25 | -1.29 | 0.087 | 0.561 | 1.000 | 4939 | tags=16%, list=15%, signal=19% |
| 486 | POSITIVE\_REGULATION\_OF\_TRANSPORT(GO:0051050) |  | 86 | -0.28 | -1.29 | 0.057 | 0.562 | 1.000 | 5831 | tags=23%, list=18%, signal=28% |
| 487 | NUCLEOSIDE\_BINDING(GO:0001882) |  | 455 | -0.23 | -1.29 | 0.029 | 0.561 | 1.000 | 4996 | tags=18%, list=16%, signal=21% |
| 488 | RIBONUCLEOSIDE\_BINDING(GO:0032549) |  | 455 | -0.23 | -1.29 | 0.029 | 0.560 | 1.000 | 4996 | tags=18%, list=16%, signal=21% |
| 489 | NIK\_NF\_KAPPAB\_SIGNALING(GO:0038061) |  | 23 | -0.43 | -1.29 | 0.134 | 0.561 | 1.000 | 809 | tags=13%, list=3%, signal=13% |
| 490 | PROTEIN\_MATURATION(GO:0051604) |  | 124 | -0.26 | -1.29 | 0.096 | 0.561 | 1.000 | 4482 | tags=17%, list=14%, signal=20% |
| 491 | PROTEIN\_PHOSPHATASE\_1\_BINDING(GO:0008157) |  | 21 | -0.40 | -1.29 | 0.143 | 0.562 | 1.000 | 4082 | tags=24%, list=13%, signal=27% |
| 492 | VACUOLE(GO:0005773) |  | 265 | -0.23 | -1.29 | 0.038 | 0.562 | 1.000 | 8796 | tags=28%, list=28%, signal=38% |
| 493 | REGULATION\_OF\_CELL\_MIGRATION(GO:0030334) |  | 217 | -0.23 | -1.29 | 0.017 | 0.561 | 1.000 | 4440 | tags=14%, list=14%, signal=16% |
| 494 | PHOSPHOLIPID\_TRANSPORTER\_ACTIVITY(GO:0005548) |  | 37 | -0.30 | -1.29 | 0.024 | 0.564 | 1.000 | 2296 | tags=14%, list=7%, signal=15% |
| 495 | RESPIRATORY\_CHAIN\_COMPLEX\_IV\_ASSEMBLY(GO:0008535) |  | 15 | -0.56 | -1.29 | 0.250 | 0.564 | 1.000 | 1201 | tags=13%, list=4%, signal=14% |
| 496 | PEPTIDYL\_PROLYL\_CIS\_TRANS\_ISOMERASE\_ACTIVITY(GO:0003755) |  | 41 | -0.37 | -1.29 | 0.194 | 0.565 | 1.000 | 8340 | tags=29%, list=26%, signal=40% |
| 497 | REGULATION\_OF\_ARP2\_3\_COMPLEX\_MEDIATED\_ACTIN\_NUCLEATION(GO:0034315) |  | 18 | -0.36 | -1.29 | 0.076 | 0.565 | 1.000 | 4549 | tags=11%, list=14%, signal=13% |
| 498 | REGULATION\_OF\_ADAPTIVE\_IMMUNE\_RESPONSE(GO:0002819) |  | 15 | -0.42 | -1.29 | 0.168 | 0.568 | 1.000 | 1287 | tags=20%, list=4%, signal=21% |
| 499 | REGULATION\_OF\_ADAPTIVE\_IMMUNE\_RESPONSE\_BASED\_ON\_SOMATIC\_RECOMBINATION\_OF\_IMMUNE\_RECEPTORS\_BUILT\_FROM\_IMMUNOGLOBULIN\_SUPERFAMILY\_DOMAINS(GO:0002822) |  | 15 | -0.42 | -1.29 | 0.168 | 0.566 | 1.000 | 1287 | tags=20%, list=4%, signal=21% |
| 500 | TRANSCRIPTION\_BY\_RNA\_POLYMERASE\_III(GO:0006383) |  | 26 | -0.43 | -1.29 | 0.184 | 0.566 | 1.000 | 10292 | tags=50%, list=32%, signal=74% |
| 501 | TRANSITION\_METAL\_ION\_HOMEOSTASIS(GO:0055076) |  | 70 | -0.24 | -1.28 | 0.111 | 0.573 | 1.000 | 2578 | tags=10%, list=8%, signal=11% |
| 502 | INTRA\_GOLGI\_VESICLE\_MEDIATED\_TRANSPORT(GO:0006891) |  | 23 | -0.32 | -1.28 | 0.106 | 0.573 | 1.000 | 146 | tags=4%, list=0%, signal=4% |
| 503 | NEGATIVE\_REGULATION\_OF\_SMALL\_MOLECULE\_METABOLIC\_PROCESS(GO:0062014) |  | 18 | -0.47 | -1.28 | 0.216 | 0.573 | 1.000 | 6328 | tags=39%, list=20%, signal=48% |
| 504 | HYDROGEN\_PEROXIDE\_CATABOLIC\_PROCESS(GO:0042744) |  | 19 | -0.40 | -1.28 | 0.141 | 0.574 | 1.000 | 1987 | tags=16%, list=6%, signal=17% |
| 505 | RESPONSE\_TO\_TEMPERATURE\_STIMULUS(GO:0009266) |  | 47 | -0.29 | -1.28 | 0.149 | 0.574 | 1.000 | 6376 | tags=28%, list=20%, signal=35% |
| 506 | CONVERGENT\_EXTENSION\_INVOLVED\_IN\_ORGANOGENESIS(GO:0060029) |  | 20 | -0.33 | -1.28 | 0.157 | 0.575 | 1.000 | 2116 | tags=10%, list=7%, signal=11% |
| 507 | REGULATION\_OF\_ACTIN\_NUCLEATION(GO:0051125) |  | 20 | -0.34 | -1.28 | 0.077 | 0.574 | 1.000 | 4549 | tags=10%, list=14%, signal=12% |
| 508 | NUCLEOTIDE\_BINDING(GO:0000166) |  | 2117 | -0.18 | -1.28 | 0.000 | 0.575 | 1.000 | 4966 | tags=13%, list=16%, signal=15% |
| 509 | NUCLEOSIDE\_PHOSPHATE\_BINDING(GO:1901265) |  | 2117 | -0.18 | -1.28 | 0.000 | 0.573 | 1.000 | 4966 | tags=13%, list=16%, signal=15% |
| 510 | GRANULOCYTE\_CHEMOTAXIS(GO:0071621) |  | 65 | -0.31 | -1.28 | 0.073 | 0.573 | 1.000 | 4402 | tags=17%, list=14%, signal=20% |
| 511 | RAS\_PROTEIN\_SIGNAL\_TRANSDUCTION(GO:0007265) |  | 195 | -0.26 | -1.28 | 0.036 | 0.573 | 1.000 | 5939 | tags=21%, list=19%, signal=25% |
| 512 | VITAMIN\_METABOLIC\_PROCESS(GO:0006766) |  | 39 | -0.35 | -1.28 | 0.208 | 0.572 | 1.000 | 3743 | tags=21%, list=12%, signal=23% |
| 513 | POSITIVE\_REGULATION\_OF\_RESPONSE\_TO\_DNA\_DAMAGE\_STIMULUS(GO:2001022) |  | 31 | -0.29 | -1.28 | 0.158 | 0.573 | 1.000 | 977 | tags=6%, list=3%, signal=7% |
| 514 | CYTOCHROME\_COMPLEX\_ASSEMBLY(GO:0017004) |  | 22 | -0.46 | -1.28 | 0.242 | 0.573 | 1.000 | 7165 | tags=27%, list=22%, signal=35% |
| 515 | CARBOHYDRATE\_DERIVATIVE\_BINDING(GO:0097367) |  | 2074 | -0.18 | -1.28 | 0.000 | 0.572 | 1.000 | 4594 | tags=12%, list=14%, signal=13% |
| 516 | NUCLEOTIDYLTRANSFERASE\_ACTIVITY(GO:0016779) |  | 108 | -0.25 | -1.28 | 0.131 | 0.572 | 1.000 | 4374 | tags=15%, list=14%, signal=17% |
| 517 | MACROMOLECULE\_CATABOLIC\_PROCESS(GO:0009057) |  | 681 | -0.19 | -1.28 | 0.050 | 0.573 | 1.000 | 4285 | tags=11%, list=13%, signal=13% |
| 518 | REGULATION\_OF\_MUSCLE\_CONTRACTION(GO:0006937) |  | 33 | -0.38 | -1.28 | 0.143 | 0.572 | 1.000 | 6330 | tags=33%, list=20%, signal=42% |
| 519 | SARCOMERE\_ORGANIZATION(GO:0045214) |  | 38 | -0.33 | -1.28 | 0.174 | 0.576 | 1.000 | 10249 | tags=53%, list=32%, signal=77% |
| 520 | CENTRAL\_NERVOUS\_SYSTEM\_NEURON\_DEVELOPMENT(GO:0021954) |  | 49 | -0.30 | -1.27 | 0.109 | 0.578 | 1.000 | 6669 | tags=29%, list=21%, signal=36% |
| 521 | CELLULAR\_RESPONSE\_TO\_INSULIN\_STIMULUS(GO:0032869) |  | 52 | -0.33 | -1.27 | 0.105 | 0.578 | 1.000 | 3951 | tags=17%, list=12%, signal=20% |
| 522 | REGULATION\_OF\_HEMATOPOIETIC\_PROGENITOR\_CELL\_DIFFERENTIATION(GO:1901532) |  | 33 | -0.33 | -1.27 | 0.167 | 0.579 | 1.000 | 6090 | tags=27%, list=19%, signal=34% |
| 523 | RIBOSOMAL\_SMALL\_SUBUNIT\_BIOGENESIS(GO:0042274) |  | 55 | -0.35 | -1.27 | 0.271 | 0.579 | 1.000 | 9140 | tags=40%, list=29%, signal=56% |
| 524 | GLANDULAR\_EPITHELIAL\_CELL\_DEVELOPMENT(GO:0002068) |  | 21 | -0.37 | -1.27 | 0.053 | 0.582 | 1.000 | 5074 | tags=24%, list=16%, signal=28% |
| 525 | SARCOMERE(GO:0030017) |  | 116 | -0.31 | -1.27 | 0.176 | 0.581 | 1.000 | 6443 | tags=29%, list=20%, signal=37% |
| 526 | CELLULAR\_COMPONENT\_BIOGENESIS(GO:0044085) |  | 1714 | -0.19 | -1.27 | 0.029 | 0.585 | 1.000 | 6852 | tags=18%, list=21%, signal=22% |
| 527 | CONTRACTILE\_FIBER\_PART(GO:0044449) |  | 120 | -0.31 | -1.27 | 0.147 | 0.585 | 1.000 | 6443 | tags=28%, list=20%, signal=35% |
| 528 | MYOFIBRIL(GO:0030016) |  | 122 | -0.30 | -1.27 | 0.150 | 0.587 | 1.000 | 6443 | tags=28%, list=20%, signal=35% |
| 529 | STRIATED\_MUSCLE\_THIN\_FILAMENT(GO:0005865) |  | 30 | -0.48 | -1.27 | 0.202 | 0.589 | 1.000 | 6330 | tags=50%, list=20%, signal=62% |
| 530 | MYOFILAMENT(GO:0036379) |  | 30 | -0.48 | -1.27 | 0.202 | 0.588 | 1.000 | 6330 | tags=50%, list=20%, signal=62% |
| 531 | SMALL\_MOLECULE\_BINDING(GO:0036094) |  | 2364 | -0.18 | -1.27 | 0.000 | 0.589 | 1.000 | 4600 | tags=13%, list=14%, signal=14% |
| 532 | HETEROTRIMERIC\_G\_PROTEIN\_COMPLEX(GO:0005834) |  | 46 | -0.31 | -1.27 | 0.219 | 0.589 | 1.000 | 219 | tags=4%, list=1%, signal=4% |
| 533 | GTPASE\_COMPLEX(GO:1905360) |  | 46 | -0.31 | -1.27 | 0.219 | 0.588 | 1.000 | 219 | tags=4%, list=1%, signal=4% |
| 534 | TRANSFERASE\_COMPLEX(GO:1990234) |  | 577 | -0.19 | -1.27 | 0.058 | 0.587 | 1.000 | 8411 | tags=23%, list=26%, signal=31% |
| 535 | CYTOPLASMIC\_TRANSLATIONAL\_INITIATION(GO:0002183) |  | 29 | -0.31 | -1.27 | 0.185 | 0.587 | 1.000 | 2500 | tags=7%, list=8%, signal=7% |
| 536 | FILOPODIUM\_ASSEMBLY(GO:0046847) |  | 23 | -0.36 | -1.27 | 0.218 | 0.586 | 1.000 | 2317 | tags=9%, list=7%, signal=9% |
| 537 | REGULATION\_OF\_CELLULAR\_COMPONENT\_MOVEMENT(GO:0051270) |  | 253 | -0.22 | -1.27 | 0.042 | 0.587 | 1.000 | 5120 | tags=16%, list=16%, signal=19% |
| 538 | CYTOSKELETON\_ORGANIZATION(GO:0007010) |  | 920 | -0.18 | -1.27 | 0.000 | 0.586 | 1.000 | 5213 | tags=13%, list=16%, signal=16% |
| 539 | BLOOD\_CIRCULATION(GO:0008015) |  | 239 | -0.25 | -1.27 | 0.084 | 0.585 | 1.000 | 6334 | tags=22%, list=20%, signal=27% |
| 540 | PROTEIN\_K63\_LINKED\_UBIQUITINATION(GO:0070534) |  | 23 | -0.38 | -1.26 | 0.129 | 0.585 | 1.000 | 11153 | tags=61%, list=35%, signal=94% |
| 541 | POLYSACCHARIDE\_BIOSYNTHETIC\_PROCESS(GO:0000271) |  | 48 | -0.29 | -1.26 | 0.141 | 0.585 | 1.000 | 5494 | tags=29%, list=17%, signal=35% |
| 542 | MATURATION\_OF\_LSU\_RRNA\_FROM\_TRICISTRONIC\_RRNA\_TRANSCRIPT\_(SSU\_RRNA\_\_5.8S\_RRNA\_\_LSU\_RRNA)(GO:0000463) |  | 15 | -0.47 | -1.26 | 0.184 | 0.586 | 1.000 | 11850 | tags=60%, list=37%, signal=95% |
| 543 | PROTEIN\_CONTAINING\_COMPLEX\_ASSEMBLY(GO:0065003) |  | 757 | -0.20 | -1.26 | 0.107 | 0.587 | 1.000 | 5264 | tags=14%, list=17%, signal=16% |
| 544 | DNA\_DIRECTED\_5'\_3'\_RNA\_POLYMERASE\_ACTIVITY(GO:0003899) |  | 29 | -0.39 | -1.26 | 0.206 | 0.586 | 1.000 | 364 | tags=7%, list=1%, signal=7% |
| 545 | CELL\_GROWTH(GO:0016049) |  | 159 | -0.25 | -1.26 | 0.231 | 0.585 | 1.000 | 5841 | tags=18%, list=18%, signal=21% |
| 546 | FOREBRAIN\_GENERATION\_OF\_NEURONS(GO:0021872) |  | 26 | -0.39 | -1.26 | 0.196 | 0.584 | 1.000 | 6848 | tags=38%, list=21%, signal=49% |
| 547 | NEGATIVE\_REGULATION\_OF\_CELL\_MOTILITY(GO:2000146) |  | 40 | -0.32 | -1.26 | 0.151 | 0.584 | 1.000 | 2317 | tags=10%, list=7%, signal=11% |
| 548 | POLYOL\_BIOSYNTHETIC\_PROCESS(GO:0046173) |  | 40 | -0.29 | -1.26 | 0.087 | 0.583 | 1.000 | 3711 | tags=25%, list=12%, signal=28% |
| 549 | AUTOPHAGOSOME\_ASSEMBLY(GO:0000045) |  | 55 | -0.29 | -1.26 | 0.224 | 0.583 | 1.000 | 4996 | tags=18%, list=16%, signal=22% |
| 550 | LEUKOCYTE\_CHEMOTAXIS(GO:0030595) |  | 84 | -0.28 | -1.26 | 0.036 | 0.582 | 1.000 | 4402 | tags=15%, list=14%, signal=18% |
| 551 | RECYCLING\_ENDOSOME\_MEMBRANE(GO:0055038) |  | 27 | -0.39 | -1.26 | 0.202 | 0.582 | 1.000 | 4206 | tags=22%, list=13%, signal=26% |
| 552 | CARBOHYDRATE\_TRANSPORT(GO:0008643) |  | 39 | -0.28 | -1.26 | 0.045 | 0.582 | 1.000 | 3853 | tags=18%, list=12%, signal=20% |
| 553 | POSITIVE\_REGULATION\_OF\_ION\_TRANSMEMBRANE\_TRANSPORTER\_ACTIVITY(GO:0032414) |  | 15 | -0.47 | -1.26 | 0.099 | 0.582 | 1.000 | 4528 | tags=33%, list=14%, signal=39% |
| 554 | TISSUE\_REMODELING(GO:0048771) |  | 21 | -0.46 | -1.26 | 0.119 | 0.581 | 1.000 | 4370 | tags=24%, list=14%, signal=28% |
| 555 | INSULIN\_LIKE\_GROWTH\_FACTOR\_BINDING(GO:0005520) |  | 31 | -0.36 | -1.26 | 0.178 | 0.580 | 1.000 | 1068 | tags=10%, list=3%, signal=10% |
| 556 | CELLULAR\_CARBOHYDRATE\_CATABOLIC\_PROCESS(GO:0044275) |  | 19 | -0.41 | -1.26 | 0.137 | 0.580 | 1.000 | 4254 | tags=32%, list=13%, signal=36% |
| 557 | MITOCHONDRIAL\_TRANSPORT(GO:0006839) |  | 109 | -0.27 | -1.26 | 0.193 | 0.580 | 1.000 | 5267 | tags=19%, list=17%, signal=23% |
| 558 | REGULATION\_OF\_HEMOPOIESIS(GO:1903706) |  | 85 | -0.30 | -1.26 | 0.183 | 0.580 | 1.000 | 6421 | tags=27%, list=20%, signal=34% |
| 559 | NUCLEUS\_ORGANIZATION(GO:0006997) |  | 37 | -0.31 | -1.26 | 0.253 | 0.580 | 1.000 | 7418 | tags=27%, list=23%, signal=35% |
| 560 | SPINDLE\_ASSEMBLY(GO:0051225) |  | 54 | -0.29 | -1.26 | 0.160 | 0.580 | 1.000 | 3830 | tags=11%, list=12%, signal=13% |
| 561 | CONTRACTILE\_FIBER(GO:0043292) |  | 123 | -0.30 | -1.26 | 0.179 | 0.580 | 1.000 | 6443 | tags=28%, list=20%, signal=35% |
| 562 | CELLULAR\_PROTEIN\_CONTAINING\_COMPLEX\_ASSEMBLY(GO:0034622) |  | 617 | -0.20 | -1.26 | 0.104 | 0.580 | 1.000 | 5213 | tags=13%, list=16%, signal=15% |
| 563 | NEGATIVE\_REGULATION\_OF\_CELLULAR\_RESPONSE\_TO\_GROWTH\_FACTOR\_STIMULUS(GO:0090288) |  | 70 | -0.29 | -1.26 | 0.129 | 0.580 | 1.000 | 2562 | tags=10%, list=8%, signal=11% |
| 564 | RIBONUCLEOTIDE\_BINDING(GO:0032553) |  | 1917 | -0.17 | -1.26 | 0.000 | 0.579 | 1.000 | 4966 | tags=13%, list=16%, signal=15% |
| 565 | NEGATIVE\_REGULATION\_OF\_SMALL\_GTPASE\_MEDIATED\_SIGNAL\_TRANSDUCTION(GO:0051058) |  | 16 | -0.44 | -1.26 | 0.229 | 0.579 | 1.000 | 1254 | tags=13%, list=4%, signal=13% |
| 566 | GOLGI\_STACK(GO:0005795) |  | 69 | -0.32 | -1.26 | 0.177 | 0.580 | 1.000 | 5932 | tags=22%, list=19%, signal=27% |
| 567 | DEVELOPMENTAL\_GROWTH\_INVOLVED\_IN\_MORPHOGENESIS(GO:0060560) |  | 183 | -0.22 | -1.26 | 0.140 | 0.580 | 1.000 | 6153 | tags=16%, list=19%, signal=20% |
| 568 | NUCLEOSIDE\_DIPHOSPHATE\_PHOSPHORYLATION(GO:0006165) |  | 57 | -0.36 | -1.26 | 0.189 | 0.581 | 1.000 | 8814 | tags=39%, list=28%, signal=53% |
| 569 | NUCLEOTIDE\_PHOSPHORYLATION(GO:0046939) |  | 57 | -0.36 | -1.26 | 0.189 | 0.580 | 1.000 | 8814 | tags=39%, list=28%, signal=53% |
| 570 | GUANYL\_NUCLEOTIDE\_BINDING(GO:0019001) |  | 477 | -0.23 | -1.26 | 0.096 | 0.579 | 1.000 | 4996 | tags=18%, list=16%, signal=21% |
| 571 | GTP\_BINDING(GO:0005525) |  | 451 | -0.23 | -1.25 | 0.094 | 0.582 | 1.000 | 4996 | tags=18%, list=16%, signal=21% |
| 572 | PURINE\_NUCLEOSIDE\_BINDING(GO:0001883) |  | 451 | -0.23 | -1.25 | 0.094 | 0.581 | 1.000 | 4996 | tags=18%, list=16%, signal=21% |
| 573 | PURINE\_RIBONUCLEOSIDE\_BINDING(GO:0032550) |  | 451 | -0.23 | -1.25 | 0.094 | 0.580 | 1.000 | 4996 | tags=18%, list=16%, signal=21% |
| 574 | PURINE\_NUCLEOTIDE\_BINDING(GO:0017076) |  | 1926 | -0.17 | -1.25 | 0.000 | 0.582 | 1.000 | 4966 | tags=13%, list=16%, signal=15% |
| 575 | CYTOSOLIC\_PART(GO:0044445) |  | 151 | -0.30 | -1.25 | 0.243 | 0.583 | 1.000 | 11045 | tags=43%, list=35%, signal=66% |
| 576 | INTEGRIN\_MEDIATED\_SIGNALING\_PATHWAY(GO:0007229) |  | 57 | -0.29 | -1.25 | 0.138 | 0.583 | 1.000 | 7863 | tags=32%, list=25%, signal=42% |
| 577 | POSITIVE\_REGULATION\_OF\_TRANSLATION(GO:0045727) |  | 38 | -0.32 | -1.25 | 0.181 | 0.583 | 1.000 | 10964 | tags=42%, list=34%, signal=64% |
| 578 | PURINE\_RIBONUCLEOTIDE\_BINDING(GO:0032555) |  | 1905 | -0.17 | -1.25 | 0.000 | 0.582 | 1.000 | 4966 | tags=13%, list=16%, signal=15% |
| 579 | CELLULAR\_COMPONENT\_ORGANIZATION\_OR\_BIOGENESIS(GO:0071840) |  | 3876 | -0.16 | -1.25 | 0.000 | 0.584 | 1.000 | 6891 | tags=18%, list=22%, signal=20% |
| 580 | REGULATION\_OF\_DNA\_TEMPLATED\_TRANSCRIPTION\_\_ELONGATION(GO:0032784) |  | 27 | -0.32 | -1.25 | 0.241 | 0.583 | 1.000 | 9838 | tags=48%, list=31%, signal=70% |
| 581 | 5'\_3'\_RNA\_POLYMERASE\_ACTIVITY(GO:0034062) |  | 30 | -0.38 | -1.25 | 0.212 | 0.583 | 1.000 | 364 | tags=7%, list=1%, signal=7% |
| 582 | RNA\_POLYMERASE\_ACTIVITY(GO:0097747) |  | 30 | -0.38 | -1.25 | 0.212 | 0.582 | 1.000 | 364 | tags=7%, list=1%, signal=7% |
| 583 | CYTOKINESIS(GO:0000910) |  | 85 | -0.23 | -1.25 | 0.113 | 0.581 | 1.000 | 1071 | tags=5%, list=3%, signal=5% |
| 584 | REGULATION\_OF\_BINDING(GO:0051098) |  | 17 | -0.34 | -1.25 | 0.156 | 0.581 | 1.000 | 4095 | tags=18%, list=13%, signal=20% |
| 585 | CYTOSKELETAL\_PROTEIN\_BINDING(GO:0008092) |  | 698 | -0.20 | -1.25 | 0.000 | 0.581 | 1.000 | 6579 | tags=20%, list=21%, signal=25% |
| 586 | PROTEIN\_DEPOLYMERIZATION(GO:0051261) |  | 75 | -0.26 | -1.25 | 0.094 | 0.582 | 1.000 | 4854 | tags=16%, list=15%, signal=19% |
| 587 | PURINE\_RIBONUCLEOSIDE\_TRIPHOSPHATE\_BINDING(GO:0035639) |  | 1877 | -0.17 | -1.25 | 0.000 | 0.582 | 1.000 | 4966 | tags=13%, list=16%, signal=14% |
| 588 | NUCLEAR\_ENVELOPE(GO:0005635) |  | 144 | -0.26 | -1.25 | 0.156 | 0.582 | 1.000 | 4366 | tags=14%, list=14%, signal=16% |
| 589 | ELECTRON\_TRANSFER\_ACTIVITY(GO:0009055) |  | 50 | -0.32 | -1.25 | 0.158 | 0.581 | 1.000 | 9107 | tags=42%, list=29%, signal=59% |
| 590 | IRON\_ION\_TRANSPORT(GO:0006826) |  | 25 | -0.36 | -1.25 | 0.225 | 0.582 | 1.000 | 5368 | tags=28%, list=17%, signal=34% |
| 591 | SIDE\_OF\_MEMBRANE(GO:0098552) |  | 248 | -0.25 | -1.25 | 0.000 | 0.582 | 1.000 | 3615 | tags=13%, list=11%, signal=14% |
| 592 | REGULATION\_OF\_SMALL\_MOLECULE\_METABOLIC\_PROCESS(GO:0062012) |  | 89 | -0.25 | -1.25 | 0.055 | 0.582 | 1.000 | 4587 | tags=19%, list=14%, signal=22% |
| 593 | EXTRINSIC\_APOPTOTIC\_SIGNALING\_PATHWAY\_IN\_ABSENCE\_OF\_LIGAND(GO:0097192) |  | 22 | -0.35 | -1.25 | 0.248 | 0.582 | 1.000 | 3630 | tags=14%, list=11%, signal=15% |
| 594 | SIGNAL\_TRANSDUCTION\_IN\_ABSENCE\_OF\_LIGAND(GO:0038034) |  | 22 | -0.35 | -1.25 | 0.248 | 0.581 | 1.000 | 3630 | tags=14%, list=11%, signal=15% |
| 595 | ACTIN\_BINDING(GO:0003779) |  | 395 | -0.20 | -1.25 | 0.111 | 0.581 | 1.000 | 6579 | tags=21%, list=21%, signal=26% |
| 596 | CELLULAR\_RESPONSE\_TO\_CHEMICAL\_STIMULUS(GO:0070887) |  | 951 | -0.20 | -1.25 | 0.020 | 0.581 | 1.000 | 3615 | tags=11%, list=11%, signal=12% |
| 597 | NEGATIVE\_REGULATION\_OF\_CELL\_MIGRATION(GO:0030336) |  | 39 | -0.33 | -1.25 | 0.187 | 0.581 | 1.000 | 2317 | tags=10%, list=7%, signal=11% |
| 598 | NEGATIVE\_REGULATION\_OF\_TOR\_SIGNALING(GO:0032007) |  | 28 | -0.38 | -1.25 | 0.148 | 0.580 | 1.000 | 5222 | tags=21%, list=16%, signal=26% |
| 599 | CELLULAR\_RESPONSE\_TO\_FIBROBLAST\_GROWTH\_FACTOR\_STIMULUS(GO:0044344) |  | 63 | -0.27 | -1.24 | 0.050 | 0.581 | 1.000 | 2706 | tags=13%, list=8%, signal=14% |
| 600 | RESPONSE\_TO\_FIBROBLAST\_GROWTH\_FACTOR(GO:0071774) |  | 63 | -0.27 | -1.24 | 0.050 | 0.580 | 1.000 | 2706 | tags=13%, list=8%, signal=14% |
| 601 | PHAGOCYTIC\_VESICLE(GO:0045335) |  | 16 | -0.48 | -1.24 | 0.223 | 0.580 | 1.000 | 2408 | tags=19%, list=8%, signal=20% |
| 602 | NUCLEAR\_DNA\_DIRECTED\_RNA\_POLYMERASE\_COMPLEX(GO:0055029) |  | 100 | -0.27 | -1.24 | 0.174 | 0.579 | 1.000 | 6949 | tags=21%, list=22%, signal=27% |
| 603 | REGULATION\_OF\_PROTEIN\_UBIQUITINATION(GO:0031396) |  | 41 | -0.28 | -1.24 | 0.184 | 0.579 | 1.000 | 3560 | tags=15%, list=11%, signal=16% |
| 604 | FIBROBLAST\_GROWTH\_FACTOR\_RECEPTOR\_SIGNALING\_PATHWAY(GO:0008543) |  | 62 | -0.27 | -1.24 | 0.050 | 0.581 | 1.000 | 2706 | tags=13%, list=8%, signal=14% |
| 605 | POSITIVE\_REGULATION\_OF\_BIOSYNTHETIC\_PROCESS(GO:0009891) |  | 520 | -0.21 | -1.24 | 0.142 | 0.581 | 1.000 | 4652 | tags=14%, list=15%, signal=16% |
| 606 | NUCLEOTIDE\_TRANSMEMBRANE\_TRANSPORTER\_ACTIVITY(GO:0015215) |  | 18 | -0.41 | -1.24 | 0.275 | 0.581 | 1.000 | 3772 | tags=17%, list=12%, signal=19% |
| 607 | CELLULAR\_METABOLIC\_COMPOUND\_SALVAGE(GO:0043094) |  | 29 | -0.34 | -1.24 | 0.119 | 0.581 | 1.000 | 4761 | tags=31%, list=15%, signal=36% |
| 608 | REGULATION\_OF\_HORMONE\_SECRETION(GO:0046883) |  | 31 | -0.42 | -1.24 | 0.241 | 0.583 | 1.000 | 5434 | tags=26%, list=17%, signal=31% |
| 609 | SUPRAMOLECULAR\_FIBER\_ORGANIZATION(GO:0097435) |  | 487 | -0.19 | -1.24 | 0.033 | 0.583 | 1.000 | 6852 | tags=21%, list=21%, signal=27% |
| 610 | REGULATION\_OF\_MUSCLE\_SYSTEM\_PROCESS(GO:0090257) |  | 38 | -0.36 | -1.24 | 0.189 | 0.583 | 1.000 | 6724 | tags=32%, list=21%, signal=40% |
| 611 | DNA\_DIRECTED\_RNA\_POLYMERASE\_COMPLEX(GO:0000428) |  | 101 | -0.27 | -1.24 | 0.174 | 0.584 | 1.000 | 6949 | tags=21%, list=22%, signal=27% |
| 612 | EPITHELIAL\_CILIUM\_MOVEMENT(GO:0003351) |  | 27 | -0.45 | -1.24 | 0.217 | 0.583 | 1.000 | 8595 | tags=48%, list=27%, signal=66% |
| 613 | CHAPERONE\_MEDIATED\_PROTEIN\_FOLDING(GO:0061077) |  | 39 | -0.34 | -1.24 | 0.084 | 0.583 | 1.000 | 3912 | tags=15%, list=12%, signal=18% |
| 614 | O\_METHYLTRANSFERASE\_ACTIVITY(GO:0008171) |  | 48 | -0.31 | -1.24 | 0.137 | 0.583 | 1.000 | 2017 | tags=10%, list=6%, signal=11% |
| 615 | REGULATION\_OF\_AUTOPHAGY(GO:0010506) |  | 74 | -0.27 | -1.24 | 0.201 | 0.582 | 1.000 | 4407 | tags=11%, list=14%, signal=13% |
| 616 | GUANYL\_RIBONUCLEOTIDE\_BINDING(GO:0032561) |  | 465 | -0.23 | -1.24 | 0.096 | 0.581 | 1.000 | 4996 | tags=18%, list=16%, signal=21% |
| 617 | POLYSOME(GO:0005844) |  | 19 | -0.33 | -1.24 | 0.145 | 0.584 | 1.000 | 6695 | tags=32%, list=21%, signal=40% |
| 618 | BLOOD\_VESSEL\_MORPHOGENESIS(GO:0048514) |  | 395 | -0.21 | -1.24 | 0.121 | 0.585 | 1.000 | 4471 | tags=13%, list=14%, signal=15% |
| 619 | AUTOPHAGY\_OF\_MITOCHONDRION(GO:0000422) |  | 34 | -0.33 | -1.24 | 0.208 | 0.587 | 1.000 | 4872 | tags=18%, list=15%, signal=21% |
| 620 | MITOCHONDRION\_DISASSEMBLY(GO:0061726) |  | 34 | -0.33 | -1.24 | 0.208 | 0.586 | 1.000 | 4872 | tags=18%, list=15%, signal=21% |
| 621 | ACTIN\_FILAMENT\_BINDING(GO:0051015) |  | 214 | -0.21 | -1.24 | 0.101 | 0.586 | 1.000 | 7105 | tags=21%, list=22%, signal=27% |
| 622 | ZINC\_ION\_TRANSMEMBRANE\_TRANSPORT(GO:0071577) |  | 15 | -0.47 | -1.24 | 0.178 | 0.585 | 1.000 | 1650 | tags=13%, list=5%, signal=14% |
| 623 | TRANSFERASE\_ACTIVITY\_\_TRANSFERRING\_ALKYL\_OR\_ARYL\_(OTHER\_THAN\_METHYL)\_GROUPS(GO:0016765) |  | 59 | -0.37 | -1.24 | 0.228 | 0.585 | 1.000 | 4356 | tags=20%, list=14%, signal=24% |
| 624 | ACTIN\_CYTOSKELETON(GO:0015629) |  | 296 | -0.22 | -1.24 | 0.235 | 0.584 | 1.000 | 6821 | tags=23%, list=21%, signal=29% |
| 625 | NEURON\_PROJECTION\_ORGANIZATION(GO:0106027) |  | 18 | -0.47 | -1.24 | 0.231 | 0.583 | 1.000 | 8934 | tags=50%, list=28%, signal=69% |
| 626 | DETERMINATION\_OF\_PANCREATIC\_LEFT\_RIGHT\_ASYMMETRY(GO:0035469) |  | 15 | -0.46 | -1.23 | 0.193 | 0.583 | 1.000 | 3491 | tags=20%, list=11%, signal=22% |
| 627 | P\_METHYLTRANSFERASE\_ACTIVITY(GO:0051994) |  | 34 | -0.34 | -1.23 | 0.165 | 0.584 | 1.000 | 1027 | tags=9%, list=3%, signal=9% |
| 628 | VACUOLE\_ORGANIZATION(GO:0007033) |  | 92 | -0.26 | -1.23 | 0.177 | 0.584 | 1.000 | 5996 | tags=17%, list=19%, signal=21% |
| 629 | POSITIVE\_REGULATION\_OF\_CELL\_MORPHOGENESIS\_INVOLVED\_IN\_DIFFERENTIATION(GO:0010770) |  | 42 | -0.27 | -1.23 | 0.109 | 0.588 | 1.000 | 5213 | tags=21%, list=16%, signal=26% |
| 630 | CLUSTER\_OF\_ACTIN\_BASED\_CELL\_PROJECTIONS(GO:0098862) |  | 29 | -0.35 | -1.23 | 0.245 | 0.588 | 1.000 | 3456 | tags=14%, list=11%, signal=15% |
| 631 | NEGATIVE\_REGULATION\_OF\_RAS\_PROTEIN\_SIGNAL\_TRANSDUCTION(GO:0046580) |  | 15 | -0.44 | -1.23 | 0.206 | 0.587 | 1.000 | 1254 | tags=13%, list=4%, signal=14% |
| 632 | 2\_POLYPRENYL\_6\_METHOXY\_1\_4\_BENZOQUINONE\_METHYLTRANSFERASE\_ACTIVITY(GO:0008425) |  | 35 | -0.34 | -1.23 | 0.165 | 0.587 | 1.000 | 1027 | tags=9%, list=3%, signal=9% |
| 633 | QUINONE\_COFACTOR\_METHYLTRANSFERASE\_ACTIVITY(GO:0030580) |  | 35 | -0.34 | -1.23 | 0.165 | 0.586 | 1.000 | 1027 | tags=9%, list=3%, signal=9% |
| 634 | REGULATION\_OF\_EXTRINSIC\_APOPTOTIC\_SIGNALING\_PATHWAY(GO:2001236) |  | 20 | -0.30 | -1.23 | 0.170 | 0.586 | 1.000 | 2579 | tags=10%, list=8%, signal=11% |
| 635 | LYSOSOMAL\_MEMBRANE(GO:0005765) |  | 95 | -0.28 | -1.23 | 0.180 | 0.585 | 1.000 | 1101 | tags=6%, list=3%, signal=7% |
| 636 | LYTIC\_VACUOLE\_MEMBRANE(GO:0098852) |  | 95 | -0.28 | -1.23 | 0.180 | 0.584 | 1.000 | 1101 | tags=6%, list=3%, signal=7% |
| 637 | PEPTIDYL\_LYSINE\_METHYLATION(GO:0018022) |  | 57 | -0.26 | -1.23 | 0.170 | 0.583 | 1.000 | 1027 | tags=5%, list=3%, signal=5% |
| 638 | RNA\_POLYMERASE\_COMPLEX(GO:0030880) |  | 102 | -0.27 | -1.23 | 0.174 | 0.584 | 1.000 | 6949 | tags=21%, list=22%, signal=26% |
| 639 | REGULATION\_OF\_TRANSLATIONAL\_INITIATION(GO:0006446) |  | 24 | -0.34 | -1.23 | 0.291 | 0.587 | 1.000 | 10601 | tags=42%, list=33%, signal=62% |
| 640 | PHOSPHATIDYLINOSITOL\_PHOSPHATE\_BINDING(GO:1901981) |  | 84 | -0.25 | -1.23 | 0.113 | 0.588 | 1.000 | 6270 | tags=18%, list=20%, signal=22% |
| 641 | PHOTORECEPTOR\_CELL\_MAINTENANCE(GO:0045494) |  | 27 | -0.32 | -1.23 | 0.160 | 0.587 | 1.000 | 7357 | tags=26%, list=23%, signal=34% |
| 642 | REGULATION\_OF\_CELL\_MOTILITY(GO:2000145) |  | 234 | -0.22 | -1.23 | 0.043 | 0.587 | 1.000 | 5120 | tags=16%, list=16%, signal=19% |
| 643 | NADP\_METABOLIC\_PROCESS(GO:0006739) |  | 16 | -0.43 | -1.23 | 0.241 | 0.586 | 1.000 | 5908 | tags=31%, list=19%, signal=38% |
| 644 | CELL\_ADHESION\_MEDIATED\_BY\_INTEGRIN(GO:0033627) |  | 20 | -0.31 | -1.23 | 0.203 | 0.587 | 1.000 | 3841 | tags=20%, list=12%, signal=23% |
| 645 | ORGANELLE\_ASSEMBLY(GO:0070925) |  | 567 | -0.21 | -1.23 | 0.158 | 0.588 | 1.000 | 6852 | tags=19%, list=21%, signal=24% |
| 646 | GLYCOSYL\_COMPOUND\_METABOLIC\_PROCESS(GO:1901657) |  | 90 | -0.23 | -1.23 | 0.099 | 0.590 | 1.000 | 6520 | tags=27%, list=20%, signal=33% |
| 647 | PURINE\_NUCLEOSIDE\_MONOPHOSPHATE\_BIOSYNTHETIC\_PROCESS(GO:0009127) |  | 98 | -0.25 | -1.23 | 0.211 | 0.589 | 1.000 | 9801 | tags=39%, list=31%, signal=56% |
| 648 | PURINE\_RIBONUCLEOSIDE\_MONOPHOSPHATE\_BIOSYNTHETIC\_PROCESS(GO:0009168) |  | 98 | -0.25 | -1.23 | 0.211 | 0.588 | 1.000 | 9801 | tags=39%, list=31%, signal=56% |
| 649 | CELL\_PROJECTION\_MEMBRANE(GO:0031253) |  | 91 | -0.27 | -1.23 | 0.166 | 0.588 | 1.000 | 5070 | tags=21%, list=16%, signal=25% |
| 650 | ESTABLISHMENT\_OR\_MAINTENANCE\_OF\_APICAL\_BASAL\_CELL\_POLARITY(GO:0035088) |  | 29 | -0.32 | -1.22 | 0.296 | 0.589 | 1.000 | 11266 | tags=52%, list=35%, signal=80% |
| 651 | ESTABLISHMENT\_OR\_MAINTENANCE\_OF\_BIPOLAR\_CELL\_POLARITY(GO:0061245) |  | 29 | -0.32 | -1.22 | 0.296 | 0.588 | 1.000 | 11266 | tags=52%, list=35%, signal=80% |
| 652 | NUCLEOBASE\_BIOSYNTHETIC\_PROCESS(GO:0046112) |  | 16 | -0.44 | -1.22 | 0.245 | 0.588 | 1.000 | 10958 | tags=56%, list=34%, signal=86% |
| 653 | REGULATION\_OF\_STEM\_CELL\_DIFFERENTIATION(GO:2000736) |  | 32 | -0.33 | -1.22 | 0.237 | 0.588 | 1.000 | 6090 | tags=28%, list=19%, signal=35% |
| 654 | CELLULAR\_COMPONENT\_ORGANIZATION(GO:0016043) |  | 3712 | -0.16 | -1.22 | 0.000 | 0.588 | 1.000 | 6891 | tags=18%, list=22%, signal=20% |
| 655 | MYOBLAST\_FUSION(GO:0007520) |  | 19 | -0.36 | -1.22 | 0.267 | 0.592 | 1.000 | 7987 | tags=32%, list=25%, signal=42% |
| 656 | POSITIVE\_REGULATION\_OF\_TRANSPORTER\_ACTIVITY(GO:0032411) |  | 17 | -0.44 | -1.22 | 0.182 | 0.592 | 1.000 | 4528 | tags=29%, list=14%, signal=34% |
| 657 | CHOLESTEROL\_BIOSYNTHETIC\_PROCESS(GO:0006695) |  | 19 | -0.37 | -1.22 | 0.229 | 0.594 | 1.000 | 1735 | tags=16%, list=5%, signal=17% |
| 658 | SECONDARY\_ALCOHOL\_BIOSYNTHETIC\_PROCESS(GO:1902653) |  | 19 | -0.37 | -1.22 | 0.229 | 0.593 | 1.000 | 1735 | tags=16%, list=5%, signal=17% |
| 659 | LEADING\_EDGE\_MEMBRANE(GO:0031256) |  | 58 | -0.29 | -1.22 | 0.136 | 0.594 | 1.000 | 5629 | tags=21%, list=18%, signal=25% |
| 660 | POSITIVE\_REGULATION\_OF\_MACROMOLECULE\_BIOSYNTHETIC\_PROCESS(GO:0010557) |  | 500 | -0.21 | -1.22 | 0.142 | 0.594 | 1.000 | 4652 | tags=14%, list=15%, signal=16% |
| 661 | GLYCOLIPID\_CATABOLIC\_PROCESS(GO:0019377) |  | 15 | -0.40 | -1.22 | 0.198 | 0.594 | 1.000 | 99 | tags=7%, list=0%, signal=7% |
| 662 | GLYCOSPHINGOLIPID\_CATABOLIC\_PROCESS(GO:0046479) |  | 15 | -0.40 | -1.22 | 0.198 | 0.593 | 1.000 | 99 | tags=7%, list=0%, signal=7% |
| 663 | TETHERING\_COMPLEX(GO:0099023) |  | 52 | -0.26 | -1.22 | 0.098 | 0.593 | 1.000 | 1655 | tags=10%, list=5%, signal=10% |
| 664 | PIGMENT\_CELL\_DIFFERENTIATION(GO:0050931) |  | 58 | -0.29 | -1.22 | 0.176 | 0.593 | 1.000 | 4519 | tags=19%, list=14%, signal=22% |
| 665 | POSITIVE\_REGULATION\_OF\_CELLULAR\_BIOSYNTHETIC\_PROCESS(GO:0031328) |  | 516 | -0.21 | -1.22 | 0.176 | 0.592 | 1.000 | 4652 | tags=14%, list=15%, signal=16% |
| 666 | KINETOCHORE(GO:0000776) |  | 60 | -0.21 | -1.22 | 0.145 | 0.592 | 1.000 | 1718 | tags=5%, list=5%, signal=5% |
| 667 | LAMELLIPODIUM(GO:0030027) |  | 64 | -0.26 | -1.22 | 0.249 | 0.592 | 1.000 | 5414 | tags=16%, list=17%, signal=19% |
| 668 | REGULATION\_OF\_PROTEIN\_MODIFICATION\_BY\_SMALL\_PROTEIN\_CONJUGATION\_OR\_REMOVAL(GO:1903320) |  | 46 | -0.27 | -1.22 | 0.141 | 0.597 | 1.000 | 3560 | tags=13%, list=11%, signal=15% |
| 669 | IRON\_ION\_HOMEOSTASIS(GO:0055072) |  | 49 | -0.26 | -1.22 | 0.127 | 0.596 | 1.000 | 2578 | tags=12%, list=8%, signal=13% |
| 670 | ANGIOBLAST\_CELL\_MIGRATION(GO:0035476) |  | 17 | -0.36 | -1.22 | 0.223 | 0.596 | 1.000 | 4939 | tags=24%, list=15%, signal=28% |
| 671 | POSITIVE\_REGULATION\_OF\_CELLULAR\_COMPONENT\_BIOGENESIS(GO:0044089) |  | 131 | -0.25 | -1.22 | 0.130 | 0.597 | 1.000 | 4549 | tags=16%, list=14%, signal=19% |
| 672 | RESPONSE\_TO\_TRANSFORMING\_GROWTH\_FACTOR\_BETA(GO:0071559) |  | 60 | -0.28 | -1.22 | 0.247 | 0.596 | 1.000 | 1934 | tags=7%, list=6%, signal=7% |
| 673 | CELLULAR\_RESPONSE\_TO\_TRANSFORMING\_GROWTH\_FACTOR\_BETA\_STIMULUS(GO:0071560) |  | 60 | -0.28 | -1.22 | 0.247 | 0.595 | 1.000 | 1934 | tags=7%, list=6%, signal=7% |
| 674 | CELLULAR\_POLYSACCHARIDE\_BIOSYNTHETIC\_PROCESS(GO:0033692) |  | 47 | -0.28 | -1.21 | 0.175 | 0.595 | 1.000 | 5494 | tags=28%, list=17%, signal=33% |
| 675 | TYPE\_B\_PANCREATIC\_CELL\_DEVELOPMENT(GO:0003323) |  | 19 | -0.38 | -1.21 | 0.142 | 0.597 | 1.000 | 5074 | tags=26%, list=16%, signal=31% |
| 676 | INTRACELLULAR\_SIGNAL\_TRANSDUCTION(GO:0035556) |  | 1379 | -0.20 | -1.21 | 0.000 | 0.599 | 1.000 | 4580 | tags=14%, list=14%, signal=16% |
| 677 | INTERMEDIATE\_FILAMENT(GO:0005882) |  | 70 | -0.32 | -1.21 | 0.104 | 0.599 | 1.000 | 6186 | tags=29%, list=19%, signal=35% |
| 678 | RESPONSE\_TO\_ENDOGENOUS\_STIMULUS(GO:0009719) |  | 483 | -0.21 | -1.21 | 0.064 | 0.599 | 1.000 | 3170 | tags=10%, list=10%, signal=10% |
| 679 | METAPHASE\_PLATE\_CONGRESSION(GO:0051310) |  | 16 | -0.35 | -1.21 | 0.250 | 0.599 | 1.000 | 987 | tags=6%, list=3%, signal=6% |
| 680 | HISTONE\_DEMETHYLATION(GO:0016577) |  | 15 | -0.31 | -1.21 | 0.159 | 0.598 | 1.000 | 2857 | tags=13%, list=9%, signal=15% |
| 681 | INTERMEDIATE\_FILAMENT\_CYTOSKELETON(GO:0045111) |  | 72 | -0.31 | -1.21 | 0.104 | 0.599 | 1.000 | 6186 | tags=28%, list=19%, signal=34% |
| 682 | DEVELOPMENTAL\_PIGMENTATION(GO:0048066) |  | 76 | -0.27 | -1.21 | 0.214 | 0.601 | 1.000 | 4519 | tags=17%, list=14%, signal=20% |
| 683 | REGULATION\_OF\_DEFENSE\_RESPONSE(GO:0031347) |  | 106 | -0.25 | -1.21 | 0.139 | 0.600 | 1.000 | 3305 | tags=13%, list=10%, signal=15% |
| 684 | TRANSFORMING\_GROWTH\_FACTOR\_BETA\_RECEPTOR\_SIGNALING\_PATHWAY(GO:0007179) |  | 59 | -0.29 | -1.21 | 0.247 | 0.602 | 1.000 | 1934 | tags=7%, list=6%, signal=7% |
| 685 | REGULATION\_OF\_COLLATERAL\_SPROUTING(GO:0048670) |  | 17 | -0.40 | -1.21 | 0.221 | 0.603 | 1.000 | 201 | tags=6%, list=1%, signal=6% |
| 686 | CYTOSOLIC\_CALCIUM\_ION\_TRANSPORT(GO:0060401) |  | 45 | -0.36 | -1.21 | 0.204 | 0.602 | 1.000 | 5936 | tags=33%, list=19%, signal=41% |
| 687 | NAD+\_KINASE\_ACTIVITY(GO:0003951) |  | 15 | -0.41 | -1.21 | 0.161 | 0.602 | 1.000 | 65 | tags=7%, list=0%, signal=7% |
| 688 | POLYSACCHARIDE\_METABOLIC\_PROCESS(GO:0005976) |  | 61 | -0.26 | -1.21 | 0.088 | 0.606 | 1.000 | 5494 | tags=28%, list=17%, signal=34% |
| 689 | TRANSCRIPTION\_BY\_RNA\_POLYMERASE\_I(GO:0006360) |  | 21 | -0.40 | -1.21 | 0.264 | 0.605 | 1.000 | 9537 | tags=48%, list=30%, signal=68% |
| 690 | DRUG\_TRANSMEMBRANE\_TRANSPORTER\_ACTIVITY(GO:0015238) |  | 70 | -0.26 | -1.21 | 0.157 | 0.605 | 1.000 | 4539 | tags=20%, list=14%, signal=23% |
| 691 | EXTRACELLULAR\_MATRIX\_ORGANIZATION(GO:0030198) |  | 173 | -0.24 | -1.21 | 0.176 | 0.604 | 1.000 | 3418 | tags=11%, list=11%, signal=12% |
| 692 | POSITIVE\_REGULATION\_OF\_CHEMOTAXIS(GO:0050921) |  | 16 | -0.46 | -1.21 | 0.175 | 0.605 | 1.000 | 2477 | tags=25%, list=8%, signal=27% |
| 693 | ENZYME\_BINDING(GO:0019899) |  | 576 | -0.18 | -1.21 | 0.022 | 0.604 | 1.000 | 6458 | tags=17%, list=20%, signal=21% |
| 694 | PURINE\_NUCLEOSIDE\_TRIPHOSPHATE\_BIOSYNTHETIC\_PROCESS(GO:0009145) |  | 80 | -0.29 | -1.21 | 0.268 | 0.605 | 1.000 | 9801 | tags=43%, list=31%, signal=61% |
| 695 | PURINE\_RIBONUCLEOSIDE\_TRIPHOSPHATE\_BIOSYNTHETIC\_PROCESS(GO:0009206) |  | 80 | -0.29 | -1.21 | 0.268 | 0.604 | 1.000 | 9801 | tags=43%, list=31%, signal=61% |
| 696 | CELLULAR\_COMPONENT\_MORPHOGENESIS(GO:0032989) |  | 736 | -0.21 | -1.21 | 0.168 | 0.604 | 1.000 | 6888 | tags=20%, list=22%, signal=25% |
| 697 | PROTEIN\_O\_LINKED\_GLYCOSYLATION(GO:0006493) |  | 25 | -0.34 | -1.20 | 0.120 | 0.604 | 1.000 | 7309 | tags=40%, list=23%, signal=52% |
| 698 | NUCLEAR\_TRANSCRIBED\_MRNA\_CATABOLIC\_PROCESS\_\_DEADENYLATION\_DEPENDENT\_DECAY(GO:0000288) |  | 37 | -0.28 | -1.20 | 0.131 | 0.604 | 1.000 | 3916 | tags=16%, list=12%, signal=18% |
| 699 | SENSORY\_PERCEPTION\_OF\_SOUND(GO:0007605) |  | 53 | -0.32 | -1.20 | 0.235 | 0.604 | 1.000 | 1382 | tags=8%, list=4%, signal=8% |
| 700 | RIBONUCLEOPROTEIN\_COMPLEX(GO:1990904) |  | 580 | -0.21 | -1.20 | 0.309 | 0.606 | 1.000 | 10615 | tags=34%, list=33%, signal=50% |
| 701 | NUCLEOPLASM\_PART(GO:0044451) |  | 433 | -0.18 | -1.20 | 0.123 | 0.607 | 1.000 | 7145 | tags=18%, list=22%, signal=22% |
| 702 | INTEGRIN\_COMPLEX(GO:0008305) |  | 32 | -0.33 | -1.20 | 0.199 | 0.607 | 1.000 | 7863 | tags=41%, list=25%, signal=54% |
| 703 | CALCIUM\_ION\_TRANSMEMBRANE\_IMPORT\_INTO\_CYTOSOL(GO:0097553) |  | 34 | -0.40 | -1.20 | 0.252 | 0.606 | 1.000 | 5936 | tags=38%, list=19%, signal=47% |
| 704 | CYTOSKELETON(GO:0005856) |  | 1199 | -0.17 | -1.20 | 0.000 | 0.607 | 1.000 | 6401 | tags=19%, list=20%, signal=23% |
| 705 | PEPTIDYL\_LYSINE\_N6\_PALMITOYLTRANSFERASE\_ACTIVITY(GO:0018031) |  | 23 | -0.36 | -1.20 | 0.192 | 0.606 | 1.000 | 9593 | tags=48%, list=30%, signal=68% |
| 706 | 2\_METHYLHEXANOYL\_COA\_C\_ACETYLTRANSFERASE\_ACTIVITY(GO:0034915) |  | 23 | -0.36 | -1.20 | 0.192 | 0.605 | 1.000 | 9593 | tags=48%, list=30%, signal=68% |
| 707 | 2\_6\_DIMETHYL\_5\_METHYLENE\_3\_OXO\_HEPTANOYL\_COA\_C\_ACETYLTRANSFERASE\_ACTIVITY(GO:0034945) |  | 23 | -0.36 | -1.20 | 0.192 | 0.604 | 1.000 | 9593 | tags=48%, list=30%, signal=68% |
| 708 | KETO\_ACID\_FORMATE\_LYASE\_ACTIVITY(GO:0043806) |  | 23 | -0.36 | -1.20 | 0.192 | 0.603 | 1.000 | 9593 | tags=48%, list=30%, signal=68% |
| 709 | N\_PALMITOYLTRANSFERASE\_ACTIVITY(GO:0019105) |  | 23 | -0.36 | -1.20 | 0.192 | 0.603 | 1.000 | 9593 | tags=48%, list=30%, signal=68% |
| 710 | REGULATION\_OF\_CELLULAR\_RESPONSE\_TO\_STRESS(GO:0080135) |  | 143 | -0.22 | -1.20 | 0.134 | 0.602 | 1.000 | 3140 | tags=10%, list=10%, signal=12% |
| 711 | REGULATION\_OF\_ION\_TRANSMEMBRANE\_TRANSPORTER\_ACTIVITY(GO:0032412) |  | 88 | -0.31 | -1.20 | 0.141 | 0.604 | 1.000 | 5885 | tags=27%, list=18%, signal=33% |
| 712 | PHOTORECEPTOR\_CELL\_OUTER\_SEGMENT\_ORGANIZATION(GO:0035845) |  | 25 | -0.33 | -1.20 | 0.209 | 0.603 | 1.000 | 119 | tags=4%, list=0%, signal=4% |
| 713 | REGULATION\_OF\_CHROMOSOME\_SEPARATION(GO:1905818) |  | 30 | -0.37 | -1.20 | 0.225 | 0.603 | 1.000 | 2255 | tags=10%, list=7%, signal=11% |
| 714 | REGULATION\_OF\_ACTIN\_CYTOSKELETON\_ORGANIZATION(GO:0032956) |  | 213 | -0.22 | -1.20 | 0.115 | 0.603 | 1.000 | 6290 | tags=20%, list=20%, signal=25% |
| 715 | POSITIVE\_REGULATION\_OF\_TRANSCRIPTION\_\_DNA\_TEMPLATED(GO:0045893) |  | 454 | -0.21 | -1.20 | 0.176 | 0.603 | 1.000 | 4652 | tags=14%, list=15%, signal=16% |
| 716 | POSITIVE\_REGULATION\_OF\_RNA\_BIOSYNTHETIC\_PROCESS(GO:1902680) |  | 454 | -0.21 | -1.20 | 0.176 | 0.602 | 1.000 | 4652 | tags=14%, list=15%, signal=16% |
| 717 | POSITIVE\_REGULATION\_OF\_NUCLEIC\_ACID\_TEMPLATED\_TRANSCRIPTION(GO:1903508) |  | 454 | -0.21 | -1.20 | 0.176 | 0.601 | 1.000 | 4652 | tags=14%, list=15%, signal=16% |
| 718 | POSITIVE\_REGULATION\_OF\_IMMUNE\_SYSTEM\_PROCESS(GO:0002684) |  | 220 | -0.25 | -1.20 | 0.076 | 0.602 | 1.000 | 3305 | tags=15%, list=10%, signal=17% |
| 719 | MYOFIBRIL\_ASSEMBLY(GO:0030239) |  | 87 | -0.26 | -1.20 | 0.162 | 0.602 | 1.000 | 6852 | tags=28%, list=21%, signal=35% |
| 720 | DEVELOPMENTAL\_CELL\_GROWTH(GO:0048588) |  | 131 | -0.24 | -1.20 | 0.210 | 0.602 | 1.000 | 5841 | tags=18%, list=18%, signal=21% |
| 721 | WHOLE\_MEMBRANE(GO:0098805) |  | 600 | -0.18 | -1.20 | 0.084 | 0.602 | 1.000 | 3952 | tags=10%, list=12%, signal=11% |
| 722 | RETINA\_HOMEOSTASIS(GO:0001895) |  | 32 | -0.30 | -1.20 | 0.137 | 0.604 | 1.000 | 7974 | tags=25%, list=25%, signal=33% |
| 723 | PROTEIN\_SECRETION(GO:0009306) |  | 64 | -0.27 | -1.20 | 0.198 | 0.603 | 1.000 | 5513 | tags=19%, list=17%, signal=23% |
| 724 | POSITIVE\_REGULATION\_OF\_INNATE\_IMMUNE\_RESPONSE(GO:0045089) |  | 48 | -0.33 | -1.20 | 0.242 | 0.603 | 1.000 | 3305 | tags=17%, list=10%, signal=19% |
| 725 | ACTIVATION\_OF\_IMMUNE\_RESPONSE(GO:0002253) |  | 128 | -0.28 | -1.20 | 0.128 | 0.603 | 1.000 | 3305 | tags=16%, list=10%, signal=17% |
| 726 | ESTABLISHMENT\_OF\_SPINDLE\_LOCALIZATION(GO:0051293) |  | 30 | -0.33 | -1.20 | 0.245 | 0.606 | 1.000 | 977 | tags=7%, list=3%, signal=7% |
| 727 | SPINDLE\_LOCALIZATION(GO:0051653) |  | 30 | -0.33 | -1.20 | 0.245 | 0.605 | 1.000 | 977 | tags=7%, list=3%, signal=7% |
| 728 | ACTIN\_FILAMENT\_ORGANIZATION(GO:0007015) |  | 320 | -0.20 | -1.20 | 0.203 | 0.605 | 1.000 | 7257 | tags=23%, list=23%, signal=30% |
| 729 | PROTEIN\_MODIFICATION\_BY\_SMALL\_PROTEIN\_CONJUGATION\_OR\_REMOVAL(GO:0070647) |  | 572 | -0.19 | -1.20 | 0.081 | 0.604 | 1.000 | 6025 | tags=16%, list=19%, signal=19% |
| 730 | LENS\_FIBER\_CELL\_DIFFERENTIATION(GO:0070306) |  | 18 | -0.41 | -1.20 | 0.243 | 0.604 | 1.000 | 6303 | tags=28%, list=20%, signal=35% |
| 731 | GUANOSINE\_CONTAINING\_COMPOUND\_BIOSYNTHETIC\_PROCESS(GO:1901070) |  | 16 | -0.42 | -1.19 | 0.327 | 0.604 | 1.000 | 9603 | tags=56%, list=30%, signal=80% |
| 732 | ESTABLISHMENT\_OR\_MAINTENANCE\_OF\_EPITHELIAL\_CELL\_APICAL\_BASAL\_POLARITY(GO:0045197) |  | 27 | -0.32 | -1.19 | 0.318 | 0.606 | 1.000 | 11266 | tags=52%, list=35%, signal=80% |
| 733 | CENTRAL\_NERVOUS\_SYSTEM\_PROJECTION\_NEURON\_AXONOGENESIS(GO:0021952) |  | 21 | -0.39 | -1.19 | 0.236 | 0.609 | 1.000 | 2761 | tags=19%, list=9%, signal=21% |
| 734 | ATPASE\_ACTIVITY\_\_COUPLED\_TO\_TRANSMEMBRANE\_MOVEMENT\_OF\_IONS\_\_ROTATIONAL\_MECHANISM(GO:0044769) |  | 17 | -0.50 | -1.19 | 0.358 | 0.608 | 1.000 | 9313 | tags=71%, list=29%, signal=100% |
| 735 | REGULATION\_OF\_LOCALIZATION(GO:0032879) |  | 763 | -0.22 | -1.19 | 0.082 | 0.608 | 1.000 | 5705 | tags=18%, list=18%, signal=21% |
| 736 | SMOOTH\_MUSCLE\_CONTRACTION(GO:0006939) |  | 27 | -0.35 | -1.19 | 0.221 | 0.607 | 1.000 | 4602 | tags=15%, list=14%, signal=17% |
| 737 | POSITIVE\_REGULATION\_OF\_I\_KAPPAB\_KINASE\_NF\_KAPPAB\_SIGNALING(GO:0043123) |  | 35 | -0.30 | -1.19 | 0.248 | 0.608 | 1.000 | 3770 | tags=17%, list=12%, signal=19% |
| 738 | APOPTOTIC\_PROCESS(GO:0006915) |  | 539 | -0.19 | -1.19 | 0.147 | 0.609 | 1.000 | 4370 | tags=13%, list=14%, signal=14% |
| 739 | OUTER\_MEMBRANE(GO:0019867) |  | 103 | -0.23 | -1.19 | 0.308 | 0.609 | 1.000 | 6389 | tags=19%, list=20%, signal=24% |
| 740 | ORGANELLE\_OUTER\_MEMBRANE(GO:0031968) |  | 103 | -0.23 | -1.19 | 0.308 | 0.608 | 1.000 | 6389 | tags=19%, list=20%, signal=24% |
| 741 | COLUMNAR\_CUBOIDAL\_EPITHELIAL\_CELL\_DEVELOPMENT(GO:0002066) |  | 42 | -0.30 | -1.19 | 0.162 | 0.609 | 1.000 | 5852 | tags=19%, list=18%, signal=23% |
| 742 | ACTIN\_FILAMENT\_POLYMERIZATION(GO:0030041) |  | 146 | -0.22 | -1.19 | 0.118 | 0.609 | 1.000 | 5213 | tags=16%, list=16%, signal=19% |
| 743 | CORTICAL\_ACTIN\_CYTOSKELETON(GO:0030864) |  | 32 | -0.34 | -1.19 | 0.217 | 0.609 | 1.000 | 8063 | tags=28%, list=25%, signal=38% |
| 744 | REGULATION\_OF\_MITOTIC\_SISTER\_CHROMATID\_SEGREGATION(GO:0033047) |  | 30 | -0.36 | -1.19 | 0.245 | 0.608 | 1.000 | 2255 | tags=10%, list=7%, signal=11% |
| 745 | GLUTATHIONE\_TRANSFERASE\_ACTIVITY(GO:0004364) |  | 24 | -0.40 | -1.19 | 0.233 | 0.608 | 1.000 | 2434 | tags=17%, list=8%, signal=18% |
| 746 | POSTERIOR\_LATERAL\_LINE\_NEUROMAST\_DEVELOPMENT(GO:0048919) |  | 32 | -0.29 | -1.19 | 0.194 | 0.609 | 1.000 | 2256 | tags=9%, list=7%, signal=10% |
| 747 | REGULATION\_OF\_ACTIN\_FILAMENT\_BASED\_PROCESS(GO:0032970) |  | 216 | -0.22 | -1.19 | 0.115 | 0.608 | 1.000 | 6290 | tags=20%, list=20%, signal=25% |
| 748 | EMBRYONIC\_EPITHELIAL\_TUBE\_FORMATION(GO:0001838) |  | 35 | -0.33 | -1.19 | 0.295 | 0.607 | 1.000 | 894 | tags=6%, list=3%, signal=6% |
| 749 | TORC1\_SIGNALING(GO:0038202) |  | 24 | -0.39 | -1.19 | 0.261 | 0.607 | 1.000 | 4352 | tags=17%, list=14%, signal=19% |
| 750 | METHYLARSONITE\_METHYLTRANSFERASE\_ACTIVITY(GO:0030792) |  | 36 | -0.33 | -1.19 | 0.232 | 0.607 | 1.000 | 1027 | tags=8%, list=3%, signal=9% |
| 751 | REGULATION\_OF\_MITOTIC\_SISTER\_CHROMATID\_SEPARATION(GO:0010965) |  | 28 | -0.38 | -1.19 | 0.248 | 0.606 | 1.000 | 2255 | tags=11%, list=7%, signal=12% |
| 752 | REGULATION\_OF\_INNATE\_IMMUNE\_RESPONSE(GO:0045088) |  | 61 | -0.30 | -1.19 | 0.277 | 0.606 | 1.000 | 3305 | tags=15%, list=10%, signal=16% |
| 753 | CELL\_PROJECTION\_ASSEMBLY(GO:0030031) |  | 358 | -0.21 | -1.19 | 0.131 | 0.607 | 1.000 | 5194 | tags=14%, list=16%, signal=16% |
| 754 | RECEPTOR\_CLUSTERING(GO:0043113) |  | 21 | -0.35 | -1.19 | 0.301 | 0.607 | 1.000 | 9083 | tags=29%, list=28%, signal=40% |
| 755 | REGULATION\_OF\_PROTEIN\_CATABOLIC\_PROCESS(GO:0042176) |  | 80 | -0.20 | -1.19 | 0.188 | 0.608 | 1.000 | 6359 | tags=15%, list=20%, signal=19% |
| 756 | GOLGI\_CISTERNA(GO:0031985) |  | 53 | -0.31 | -1.18 | 0.208 | 0.610 | 1.000 | 5932 | tags=25%, list=19%, signal=30% |
| 757 | POSITIVE\_REGULATION\_OF\_NUCLEOBASE\_CONTAINING\_COMPOUND\_METABOLIC\_PROCESS(GO:0045935) |  | 534 | -0.21 | -1.18 | 0.205 | 0.610 | 1.000 | 4577 | tags=14%, list=14%, signal=16% |
| 758 | SKELETAL\_SYSTEM\_MORPHOGENESIS(GO:0048705) |  | 200 | -0.23 | -1.18 | 0.126 | 0.609 | 1.000 | 6543 | tags=20%, list=21%, signal=24% |
| 759 | NEUROMUSCULAR\_JUNCTION\_DEVELOPMENT(GO:0007528) |  | 35 | -0.30 | -1.18 | 0.286 | 0.611 | 1.000 | 6240 | tags=29%, list=20%, signal=35% |
| 760 | POSTERIOR\_LATERAL\_LINE\_DEVELOPMENT(GO:0048916) |  | 63 | -0.23 | -1.18 | 0.181 | 0.613 | 1.000 | 5980 | tags=21%, list=19%, signal=25% |
| 761 | GLOMERULUS\_MORPHOGENESIS(GO:0072102) |  | 16 | -0.35 | -1.18 | 0.135 | 0.613 | 1.000 | 5637 | tags=25%, list=18%, signal=30% |
| 762 | PROGRAMMED\_CELL\_DEATH(GO:0012501) |  | 551 | -0.19 | -1.18 | 0.147 | 0.612 | 1.000 | 4370 | tags=12%, list=14%, signal=14% |
| 763 | ANGIOGENESIS(GO:0001525) |  | 325 | -0.21 | -1.18 | 0.153 | 0.612 | 1.000 | 3959 | tags=12%, list=12%, signal=14% |
| 764 | PHOSPHATASE\_ACTIVITY(GO:0016791) |  | 283 | -0.20 | -1.18 | 0.178 | 0.614 | 1.000 | 4405 | tags=14%, list=14%, signal=17% |
| 765 | MITOTIC\_SISTER\_CHROMATID\_SEPARATION(GO:0051306) |  | 29 | -0.37 | -1.18 | 0.284 | 0.614 | 1.000 | 2255 | tags=10%, list=7%, signal=11% |
| 766 | TRANS\_GOLGI\_NETWORK(GO:0005802) |  | 98 | -0.23 | -1.18 | 0.285 | 0.613 | 1.000 | 6445 | tags=26%, list=20%, signal=32% |
| 767 | C\_METHYLTRANSFERASE\_ACTIVITY(GO:0008169) |  | 37 | -0.31 | -1.18 | 0.224 | 0.613 | 1.000 | 1027 | tags=8%, list=3%, signal=8% |
| 768 | RETINA\_LAYER\_FORMATION(GO:0010842) |  | 46 | -0.31 | -1.18 | 0.226 | 0.612 | 1.000 | 5154 | tags=17%, list=16%, signal=21% |
| 769 | REGULATION\_OF\_ENDODERMAL\_CELL\_FATE\_SPECIFICATION(GO:0042663) |  | 16 | -0.43 | -1.18 | 0.256 | 0.614 | 1.000 | 5220 | tags=19%, list=16%, signal=22% |
| 770 | ACTIN\_POLYMERIZATION\_OR\_DEPOLYMERIZATION(GO:0008154) |  | 163 | -0.22 | -1.18 | 0.250 | 0.615 | 1.000 | 7402 | tags=23%, list=23%, signal=29% |
| 771 | CYTOPLASMIC\_SIDE\_OF\_MEMBRANE(GO:0098562) |  | 112 | -0.24 | -1.18 | 0.276 | 0.615 | 1.000 | 550 | tags=4%, list=2%, signal=4% |
| 772 | HEPARAN\_SULFATE\_PROTEOGLYCAN\_METABOLIC\_PROCESS(GO:0030201) |  | 28 | -0.31 | -1.18 | 0.178 | 0.614 | 1.000 | 1617 | tags=7%, list=5%, signal=8% |
| 773 | TOLL\_LIKE\_RECEPTOR\_SIGNALING\_PATHWAY(GO:0002224) |  | 32 | -0.33 | -1.18 | 0.309 | 0.615 | 1.000 | 3305 | tags=16%, list=10%, signal=17% |
| 774 | GLUCOSE\_HOMEOSTASIS(GO:0042593) |  | 35 | -0.35 | -1.18 | 0.288 | 0.619 | 1.000 | 212 | tags=6%, list=1%, signal=6% |
| 775 | CARBOHYDRATE\_HOMEOSTASIS(GO:0033500) |  | 35 | -0.35 | -1.18 | 0.288 | 0.619 | 1.000 | 212 | tags=6%, list=1%, signal=6% |
| 776 | POSITIVE\_REGULATION\_OF\_DEFENSE\_RESPONSE(GO:0031349) |  | 49 | -0.32 | -1.18 | 0.282 | 0.618 | 1.000 | 5854 | tags=24%, list=18%, signal=30% |
| 777 | CELLULAR\_CARBOHYDRATE\_METABOLIC\_PROCESS(GO:0044262) |  | 121 | -0.23 | -1.18 | 0.177 | 0.617 | 1.000 | 3800 | tags=18%, list=12%, signal=21% |
| 778 | CYTOSOLIC\_SMALL\_RIBOSOMAL\_SUBUNIT(GO:0022627) |  | 35 | -0.40 | -1.18 | 0.322 | 0.617 | 1.000 | 12397 | tags=54%, list=39%, signal=89% |
| 779 | HYDROGEN\_PEROXIDE\_METABOLIC\_PROCESS(GO:0042743) |  | 22 | -0.36 | -1.18 | 0.203 | 0.617 | 1.000 | 1987 | tags=14%, list=6%, signal=15% |
| 780 | PHOSPHATIDYLINOSITOL\_3\_PHOSPHATE\_BINDING(GO:0032266) |  | 20 | -0.33 | -1.18 | 0.280 | 0.617 | 1.000 | 21289 | tags=100%, list=67%, signal=301% |
| 781 | LEUKOCYTE\_ACTIVATION(GO:0045321) |  | 160 | -0.23 | -1.17 | 0.161 | 0.618 | 1.000 | 3615 | tags=14%, list=11%, signal=15% |
| 782 | COMPLEMENT\_ACTIVATION(GO:0006956) |  | 40 | -0.35 | -1.17 | 0.205 | 0.618 | 1.000 | 2218 | tags=20%, list=7%, signal=21% |
| 783 | HYALURONIC\_ACID\_BINDING(GO:0005540) |  | 22 | -0.36 | -1.17 | 0.182 | 0.618 | 1.000 | 4442 | tags=32%, list=14%, signal=37% |
| 784 | PROTEIN\_CONTAINING\_COMPLEX\_LOCALIZATION(GO:0031503) |  | 124 | -0.25 | -1.17 | 0.276 | 0.620 | 1.000 | 9170 | tags=37%, list=29%, signal=52% |
| 785 | PROTEIN\_COMPLEX\_INVOLVED\_IN\_CELL\_ADHESION(GO:0098636) |  | 33 | -0.32 | -1.17 | 0.300 | 0.620 | 1.000 | 7863 | tags=39%, list=25%, signal=52% |
| 786 | SUPRAMOLECULAR\_FIBER(GO:0099512) |  | 478 | -0.19 | -1.17 | 0.126 | 0.620 | 1.000 | 6443 | tags=20%, list=20%, signal=25% |
| 787 | CELLULAR\_METAL\_ION\_HOMEOSTASIS(GO:0006875) |  | 256 | -0.23 | -1.17 | 0.106 | 0.620 | 1.000 | 5936 | tags=20%, list=19%, signal=24% |
| 788 | PROTEIN\_MODIFICATION\_BY\_SMALL\_PROTEIN\_CONJUGATION(GO:0032446) |  | 476 | -0.19 | -1.17 | 0.102 | 0.620 | 1.000 | 6025 | tags=17%, list=19%, signal=20% |
| 789 | RIBONUCLEOSIDE\_MONOPHOSPHATE\_BIOSYNTHETIC\_PROCESS(GO:0009156) |  | 112 | -0.22 | -1.17 | 0.305 | 0.620 | 1.000 | 6789 | tags=23%, list=21%, signal=29% |
| 790 | REGULATION\_OF\_TRANSPORTER\_ACTIVITY(GO:0032409) |  | 92 | -0.30 | -1.17 | 0.213 | 0.620 | 1.000 | 6583 | tags=29%, list=21%, signal=37% |
| 791 | NUCLEUS(GO:0005634) |  | 4444 | -0.16 | -1.17 | 0.180 | 0.620 | 1.000 | 6467 | tags=16%, list=20%, signal=17% |
| 792 | REGULATION\_OF\_CELL\_PROLIFERATION(GO:0042127) |  | 289 | -0.20 | -1.17 | 0.070 | 0.619 | 1.000 | 3763 | tags=11%, list=12%, signal=13% |
| 793 | CELLULAR\_LOCALIZATION(GO:0051641) |  | 1339 | -0.17 | -1.17 | 0.070 | 0.622 | 1.000 | 5368 | tags=14%, list=17%, signal=16% |
| 794 | REGULATION\_OF\_TRANSMEMBRANE\_TRANSPORTER\_ACTIVITY(GO:0022898) |  | 91 | -0.30 | -1.17 | 0.245 | 0.622 | 1.000 | 5885 | tags=26%, list=18%, signal=32% |
| 795 | REGULATION\_OF\_ENDODERMAL\_CELL\_DIFFERENTIATION(GO:1903224) |  | 18 | -0.42 | -1.17 | 0.371 | 0.622 | 1.000 | 10821 | tags=44%, list=34%, signal=67% |
| 796 | NEGATIVE\_REGULATION\_OF\_IMMUNE\_SYSTEM\_PROCESS(GO:0002683) |  | 72 | -0.29 | -1.17 | 0.238 | 0.622 | 1.000 | 1287 | tags=13%, list=4%, signal=13% |
| 797 | GOLGI\_CISTERNA\_MEMBRANE(GO:0032580) |  | 46 | -0.31 | -1.17 | 0.221 | 0.622 | 1.000 | 5932 | tags=24%, list=19%, signal=29% |
| 798 | CELLULAR\_POLYSACCHARIDE\_METABOLIC\_PROCESS(GO:0044264) |  | 60 | -0.25 | -1.17 | 0.138 | 0.622 | 1.000 | 5494 | tags=27%, list=17%, signal=32% |
| 799 | BARBED\_END\_ACTIN\_FILAMENT\_CAPPING(GO:0051016) |  | 28 | -0.36 | -1.17 | 0.284 | 0.621 | 1.000 | 2910 | tags=14%, list=9%, signal=16% |
| 800 | METAL\_ION\_BINDING(GO:0046872) |  | 3536 | -0.17 | -1.17 | 0.120 | 0.621 | 1.000 | 6152 | tags=18%, list=19%, signal=20% |
| 801 | ACETYLCHOLINE\_RECEPTOR\_ACTIVITY(GO:0015464) |  | 23 | -0.43 | -1.17 | 0.220 | 0.620 | 1.000 | 3710 | tags=22%, list=12%, signal=25% |
| 802 | POSITIVE\_REGULATION\_OF\_RNA\_METABOLIC\_PROCESS(GO:0051254) |  | 487 | -0.21 | -1.17 | 0.208 | 0.620 | 1.000 | 4652 | tags=14%, list=15%, signal=16% |
| 803 | FILOPODIUM(GO:0030175) |  | 37 | -0.29 | -1.17 | 0.206 | 0.620 | 1.000 | 5462 | tags=19%, list=17%, signal=23% |
| 804 | POSITIVE\_REGULATION\_OF\_MAP\_KINASE\_ACTIVITY(GO:0043406) |  | 16 | -0.38 | -1.17 | 0.285 | 0.620 | 1.000 | 2376 | tags=19%, list=7%, signal=20% |
| 805 | FOREBRAIN\_NEURON\_DIFFERENTIATION(GO:0021879) |  | 23 | -0.38 | -1.17 | 0.245 | 0.620 | 1.000 | 6489 | tags=35%, list=20%, signal=44% |
| 806 | CALCIUM\_ION\_TRANSPORT\_INTO\_CYTOSOL(GO:0060402) |  | 36 | -0.38 | -1.17 | 0.291 | 0.621 | 1.000 | 5936 | tags=36%, list=19%, signal=44% |
| 807 | SUPRAMOLECULAR\_COMPLEX(GO:0099080) |  | 481 | -0.18 | -1.17 | 0.126 | 0.621 | 1.000 | 6443 | tags=20%, list=20%, signal=25% |
| 808 | SUPRAMOLECULAR\_POLYMER(GO:0099081) |  | 481 | -0.18 | -1.17 | 0.126 | 0.620 | 1.000 | 6443 | tags=20%, list=20%, signal=25% |
| 809 | LYSOSOME(GO:0005764) |  | 203 | -0.20 | -1.17 | 0.118 | 0.621 | 1.000 | 8241 | tags=24%, list=26%, signal=32% |
| 810 | HEART\_FORMATION(GO:0060914) |  | 27 | -0.33 | -1.16 | 0.281 | 0.622 | 1.000 | 4866 | tags=22%, list=15%, signal=26% |
| 811 | TRANSCRIPTION\_FACTOR\_BINDING(GO:0008134) |  | 95 | -0.25 | -1.16 | 0.230 | 0.624 | 1.000 | 3176 | tags=9%, list=10%, signal=10% |
| 812 | REGULATION\_OF\_PROTEIN\_POLYMERIZATION(GO:0032271) |  | 146 | -0.21 | -1.16 | 0.148 | 0.626 | 1.000 | 5213 | tags=15%, list=16%, signal=18% |
| 813 | COLLATERAL\_SPROUTING(GO:0048668) |  | 19 | -0.36 | -1.16 | 0.302 | 0.626 | 1.000 | 201 | tags=5%, list=1%, signal=5% |
| 814 | CD4\_POSITIVE\_\_ALPHA\_BETA\_T\_CELL\_ACTIVATION(GO:0035710) |  | 15 | -0.39 | -1.16 | 0.225 | 0.625 | 1.000 | 1287 | tags=13%, list=4%, signal=14% |
| 815 | LYTIC\_VACUOLE(GO:0000323) |  | 204 | -0.20 | -1.16 | 0.118 | 0.625 | 1.000 | 8241 | tags=24%, list=26%, signal=32% |
| 816 | NADH\_DEHYDROGENASE\_ACTIVITY(GO:0003954) |  | 23 | -0.37 | -1.16 | 0.286 | 0.625 | 1.000 | 9722 | tags=30%, list=30%, signal=44% |
| 817 | CYTOPLASMIC\_PART(GO:0044444) |  | 4559 | -0.17 | -1.16 | 0.239 | 0.625 | 1.000 | 6636 | tags=18%, list=21%, signal=20% |
| 818 | PEPTIDE\_SECRETION(GO:0002790) |  | 65 | -0.27 | -1.16 | 0.233 | 0.626 | 1.000 | 5513 | tags=18%, list=17%, signal=22% |
| 819 | CYTOPLASMIC\_SIDE\_OF\_PLASMA\_MEMBRANE(GO:0009898) |  | 106 | -0.25 | -1.16 | 0.322 | 0.625 | 1.000 | 550 | tags=4%, list=2%, signal=4% |
| 820 | NUCLEOSIDE\_MONOPHOSPHATE\_BIOSYNTHETIC\_PROCESS(GO:0009124) |  | 115 | -0.22 | -1.16 | 0.362 | 0.624 | 1.000 | 6789 | tags=23%, list=21%, signal=29% |
| 821 | CELLULAR\_RESPONSE\_TO\_REACTIVE\_OXYGEN\_SPECIES(GO:0034614) |  | 16 | -0.39 | -1.16 | 0.337 | 0.624 | 1.000 | 5141 | tags=31%, list=16%, signal=37% |
| 822 | PROTEIN\_UBIQUITINATION(GO:0016567) |  | 446 | -0.19 | -1.16 | 0.133 | 0.624 | 1.000 | 6025 | tags=17%, list=19%, signal=20% |
| 823 | POSITIVE\_REGULATION\_OF\_CELLULAR\_COMPONENT\_ORGANIZATION(GO:0051130) |  | 269 | -0.20 | -1.16 | 0.234 | 0.624 | 1.000 | 5919 | tags=17%, list=19%, signal=21% |
| 824 | CATION\_BINDING(GO:0043169) |  | 3595 | -0.17 | -1.16 | 0.120 | 0.625 | 1.000 | 6152 | tags=18%, list=19%, signal=20% |
| 825 | CHEMICAL\_HOMEOSTASIS(GO:0048878) |  | 466 | -0.21 | -1.16 | 0.067 | 0.624 | 1.000 | 5936 | tags=19%, list=19%, signal=23% |
| 826 | TYPE\_B\_PANCREATIC\_CELL\_DIFFERENTIATION(GO:0003309) |  | 29 | -0.34 | -1.16 | 0.232 | 0.624 | 1.000 | 5634 | tags=28%, list=18%, signal=33% |
| 827 | RNA\_PROCESSING(GO:0006396) |  | 643 | -0.17 | -1.16 | 0.264 | 0.623 | 1.000 | 9960 | tags=30%, list=31%, signal=42% |
| 828 | RIBONUCLEOSIDE\_TRIPHOSPHATE\_BIOSYNTHETIC\_PROCESS(GO:0009201) |  | 89 | -0.27 | -1.16 | 0.348 | 0.625 | 1.000 | 9801 | tags=42%, list=31%, signal=60% |
| 829 | MEMBRANE\_ENCLOSED\_LUMEN(GO:0031974) |  | 1175 | -0.16 | -1.16 | 0.168 | 0.627 | 1.000 | 6882 | tags=17%, list=22%, signal=21% |
| 830 | ORGANELLE\_LUMEN(GO:0043233) |  | 1175 | -0.16 | -1.16 | 0.168 | 0.627 | 1.000 | 6882 | tags=17%, list=22%, signal=21% |
| 831 | INTRACELLULAR\_ORGANELLE\_LUMEN(GO:0070013) |  | 1175 | -0.16 | -1.16 | 0.168 | 0.626 | 1.000 | 6882 | tags=17%, list=22%, signal=21% |
| 832 | AXON\_EXTENSION(GO:0048675) |  | 108 | -0.24 | -1.16 | 0.255 | 0.626 | 1.000 | 5798 | tags=19%, list=18%, signal=23% |
| 833 | PYRIDINE\_CONTAINING\_COMPOUND\_METABOLIC\_PROCESS(GO:0072524) |  | 80 | -0.29 | -1.16 | 0.355 | 0.627 | 1.000 | 6925 | tags=30%, list=22%, signal=38% |
| 834 | BLOOD\_VESSEL\_DEVELOPMENT(GO:0001568) |  | 443 | -0.20 | -1.16 | 0.199 | 0.628 | 1.000 | 4471 | tags=13%, list=14%, signal=14% |
| 835 | DOPAMINERGIC\_NEURON\_DIFFERENTIATION(GO:0071542) |  | 17 | -0.43 | -1.16 | 0.299 | 0.628 | 1.000 | 9964 | tags=59%, list=31%, signal=86% |
| 836 | REGULATION\_OF\_TOR\_SIGNALING(GO:0032006) |  | 59 | -0.29 | -1.16 | 0.197 | 0.627 | 1.000 | 2887 | tags=10%, list=9%, signal=11% |
| 837 | PROTEIN\_TYROSINE\_PHOSPHATASE\_ACTIVITY(GO:0004725) |  | 119 | -0.21 | -1.16 | 0.198 | 0.626 | 1.000 | 3632 | tags=12%, list=11%, signal=13% |
| 838 | INTRINSIC\_APOPTOTIC\_SIGNALING\_PATHWAY\_BY\_P53\_CLASS\_MEDIATOR(GO:0072332) |  | 23 | -0.37 | -1.16 | 0.241 | 0.626 | 1.000 | 5118 | tags=35%, list=16%, signal=41% |
| 839 | NUCLEAR\_BODY(GO:0016604) |  | 122 | -0.18 | -1.15 | 0.123 | 0.626 | 1.000 | 7397 | tags=22%, list=23%, signal=29% |
| 840 | PYRIDINE\_NUCLEOTIDE\_METABOLIC\_PROCESS(GO:0019362) |  | 76 | -0.29 | -1.15 | 0.369 | 0.628 | 1.000 | 6789 | tags=29%, list=21%, signal=37% |
| 841 | NICOTINAMIDE\_NUCLEOTIDE\_METABOLIC\_PROCESS(GO:0046496) |  | 76 | -0.29 | -1.15 | 0.369 | 0.627 | 1.000 | 6789 | tags=29%, list=21%, signal=37% |
| 842 | TOR\_SIGNALING(GO:0031929) |  | 70 | -0.30 | -1.15 | 0.241 | 0.627 | 1.000 | 5222 | tags=16%, list=16%, signal=19% |
| 843 | ORGANOPHOSPHATE\_ESTER\_TRANSMEMBRANE\_TRANSPORTER\_ACTIVITY(GO:0015605) |  | 22 | -0.35 | -1.15 | 0.159 | 0.626 | 1.000 | 3772 | tags=14%, list=12%, signal=15% |
| 844 | CELLULAR\_COMPONENT\_ASSEMBLY\_INVOLVED\_IN\_MORPHOGENESIS(GO:0010927) |  | 95 | -0.24 | -1.15 | 0.210 | 0.627 | 1.000 | 6852 | tags=25%, list=21%, signal=32% |
| 845 | PROTEIN\_GLYCOSYLATION(GO:0006486) |  | 187 | -0.23 | -1.15 | 0.143 | 0.628 | 1.000 | 6617 | tags=21%, list=21%, signal=26% |
| 846 | MACROMOLECULE\_GLYCOSYLATION(GO:0043413) |  | 187 | -0.23 | -1.15 | 0.143 | 0.628 | 1.000 | 6617 | tags=21%, list=21%, signal=26% |
| 847 | POSITIVE\_REGULATION\_OF\_RESPONSE\_TO\_STIMULUS(GO:0048584) |  | 595 | -0.21 | -1.15 | 0.137 | 0.628 | 1.000 | 3460 | tags=11%, list=11%, signal=12% |
| 848 | MITOTIC\_SPINDLE\_ASSEMBLY\_CHECKPOINT(GO:0007094) |  | 17 | -0.38 | -1.15 | 0.305 | 0.627 | 1.000 | 384 | tags=6%, list=1%, signal=6% |
| 849 | SPINDLE\_CHECKPOINT(GO:0031577) |  | 17 | -0.38 | -1.15 | 0.305 | 0.626 | 1.000 | 384 | tags=6%, list=1%, signal=6% |
| 850 | NEGATIVE\_REGULATION\_OF\_MITOTIC\_METAPHASE\_ANAPHASE\_TRANSITION(GO:0045841) |  | 17 | -0.38 | -1.15 | 0.305 | 0.626 | 1.000 | 384 | tags=6%, list=1%, signal=6% |
| 851 | SPINDLE\_ASSEMBLY\_CHECKPOINT(GO:0071173) |  | 17 | -0.38 | -1.15 | 0.305 | 0.625 | 1.000 | 384 | tags=6%, list=1%, signal=6% |
| 852 | MITOTIC\_SPINDLE\_CHECKPOINT(GO:0071174) |  | 17 | -0.38 | -1.15 | 0.305 | 0.624 | 1.000 | 384 | tags=6%, list=1%, signal=6% |
| 853 | NEGATIVE\_REGULATION\_OF\_METAPHASE\_ANAPHASE\_TRANSITION\_OF\_CELL\_CYCLE(GO:1902100) |  | 17 | -0.38 | -1.15 | 0.305 | 0.623 | 1.000 | 384 | tags=6%, list=1%, signal=6% |
| 854 | DNA\_TEMPLATED\_TRANSCRIPTION\_\_ELONGATION(GO:0006354) |  | 39 | -0.24 | -1.15 | 0.210 | 0.626 | 1.000 | 6090 | tags=23%, list=19%, signal=28% |
| 855 | PURINE\_CONTAINING\_COMPOUND\_BIOSYNTHETIC\_PROCESS(GO:0072522) |  | 172 | -0.22 | -1.15 | 0.143 | 0.626 | 1.000 | 6379 | tags=23%, list=20%, signal=28% |
| 856 | CARBOHYDRATE\_PHOSPHATASE\_ACTIVITY(GO:0019203) |  | 16 | -0.42 | -1.15 | 0.276 | 0.626 | 1.000 | 6163 | tags=38%, list=19%, signal=46% |
| 857 | SUGAR\_PHOSPHATASE\_ACTIVITY(GO:0050308) |  | 16 | -0.42 | -1.15 | 0.276 | 0.625 | 1.000 | 6163 | tags=38%, list=19%, signal=46% |
| 858 | RESPONSE\_TO\_MECHANICAL\_STIMULUS(GO:0009612) |  | 49 | -0.29 | -1.15 | 0.275 | 0.625 | 1.000 | 5387 | tags=20%, list=17%, signal=25% |
| 859 | MICROTUBULE\_DEPOLYMERIZATION(GO:0007019) |  | 23 | -0.35 | -1.15 | 0.281 | 0.626 | 1.000 | 4828 | tags=17%, list=15%, signal=20% |
| 860 | POSITIVE\_REGULATION\_OF\_GENE\_EXPRESSION(GO:0010628) |  | 565 | -0.19 | -1.15 | 0.207 | 0.626 | 1.000 | 4652 | tags=13%, list=15%, signal=15% |
| 861 | NEGATIVE\_REGULATION\_OF\_APOPTOTIC\_SIGNALING\_PATHWAY(GO:2001234) |  | 37 | -0.27 | -1.15 | 0.329 | 0.626 | 1.000 | 5118 | tags=19%, list=16%, signal=23% |
| 862 | REGULATION\_OF\_TRANSFORMING\_GROWTH\_FACTOR\_BETA\_RECEPTOR\_SIGNALING\_PATHWAY(GO:0017015) |  | 26 | -0.32 | -1.15 | 0.297 | 0.626 | 1.000 | 1934 | tags=8%, list=6%, signal=8% |
| 863 | REGULATION\_OF\_CELLULAR\_RESPONSE\_TO\_TRANSFORMING\_GROWTH\_FACTOR\_BETA\_STIMULUS(GO:1903844) |  | 26 | -0.32 | -1.15 | 0.297 | 0.625 | 1.000 | 1934 | tags=8%, list=6%, signal=8% |
| 864 | DIGESTIVE\_TRACT\_MORPHOGENESIS(GO:0048546) |  | 32 | -0.36 | -1.15 | 0.388 | 0.626 | 1.000 | 3062 | tags=9%, list=10%, signal=10% |
| 865 | FIN\_MORPHOGENESIS(GO:0033334) |  | 56 | -0.25 | -1.15 | 0.227 | 0.626 | 1.000 | 8153 | tags=32%, list=26%, signal=43% |
| 866 | BASAL\_PART\_OF\_CELL(GO:0045178) |  | 17 | -0.36 | -1.15 | 0.269 | 0.625 | 1.000 | 354 | tags=6%, list=1%, signal=6% |
| 867 | HISTONE\_UBIQUITINATION(GO:0016574) |  | 25 | -0.32 | -1.15 | 0.315 | 0.625 | 1.000 | 10335 | tags=40%, list=32%, signal=59% |
| 868 | NEURON\_PROJECTION\_EXTENSION(GO:1990138) |  | 116 | -0.23 | -1.15 | 0.205 | 0.627 | 1.000 | 5798 | tags=18%, list=18%, signal=22% |
| 869 | SKELETAL\_MYOFIBRIL\_ASSEMBLY(GO:0014866) |  | 21 | -0.35 | -1.15 | 0.267 | 0.627 | 1.000 | 6296 | tags=24%, list=20%, signal=30% |
| 870 | NEGATIVE\_REGULATION\_OF\_CHROMOSOME\_ORGANIZATION(GO:2001251) |  | 38 | -0.29 | -1.14 | 0.363 | 0.629 | 1.000 | 2574 | tags=11%, list=8%, signal=11% |
| 871 | SENSORY\_PERCEPTION\_OF\_MECHANICAL\_STIMULUS(GO:0050954) |  | 62 | -0.28 | -1.14 | 0.202 | 0.629 | 1.000 | 1382 | tags=6%, list=4%, signal=7% |
| 872 | PROTEIN\_ACTIVATION\_CASCADE(GO:0072376) |  | 49 | -0.35 | -1.14 | 0.291 | 0.628 | 1.000 | 2218 | tags=18%, list=7%, signal=20% |
| 873 | ARTERY\_DEVELOPMENT(GO:0060840) |  | 37 | -0.28 | -1.14 | 0.260 | 0.631 | 1.000 | 4277 | tags=16%, list=13%, signal=19% |
| 874 | NEGATIVE\_REGULATION\_OF\_ORGANELLE\_ORGANIZATION(GO:0010639) |  | 123 | -0.22 | -1.14 | 0.224 | 0.632 | 1.000 | 2910 | tags=9%, list=9%, signal=10% |
| 875 | GLYCOSAMINOGLYCAN\_BINDING(GO:0005539) |  | 103 | -0.24 | -1.14 | 0.190 | 0.634 | 1.000 | 4471 | tags=16%, list=14%, signal=18% |
| 876 | NUCLEAR\_TRANSCRIBED\_MRNA\_CATABOLIC\_PROCESS(GO:0000956) |  | 74 | -0.23 | -1.14 | 0.261 | 0.635 | 1.000 | 6074 | tags=22%, list=19%, signal=27% |
| 877 | SEMAPHORIN\_PLEXIN\_SIGNALING\_PATHWAY(GO:0071526) |  | 46 | -0.30 | -1.14 | 0.290 | 0.635 | 1.000 | 3976 | tags=15%, list=12%, signal=17% |
| 878 | EXOCYTOSIS(GO:0006887) |  | 180 | -0.24 | -1.14 | 0.228 | 0.635 | 1.000 | 6861 | tags=26%, list=22%, signal=32% |
| 879 | PROTEIN\_LIPID\_COMPLEX(GO:0032994) |  | 15 | -0.40 | -1.14 | 0.298 | 0.635 | 1.000 | 932 | tags=13%, list=3%, signal=14% |
| 880 | PLASMA\_LIPOPROTEIN\_PARTICLE(GO:0034358) |  | 15 | -0.40 | -1.14 | 0.298 | 0.634 | 1.000 | 932 | tags=13%, list=3%, signal=14% |
| 881 | LIPOPROTEIN\_PARTICLE(GO:1990777) |  | 15 | -0.40 | -1.14 | 0.298 | 0.633 | 1.000 | 932 | tags=13%, list=3%, signal=14% |
| 882 | POST\_GOLGI\_VESICLE\_MEDIATED\_TRANSPORT(GO:0006892) |  | 42 | -0.22 | -1.14 | 0.242 | 0.633 | 1.000 | 7421 | tags=31%, list=23%, signal=40% |
| 883 | GLYCOPROTEIN\_METABOLIC\_PROCESS(GO:0009100) |  | 274 | -0.22 | -1.14 | 0.274 | 0.633 | 1.000 | 6199 | tags=19%, list=19%, signal=23% |
| 884 | N\_METHYLTRANSFERASE\_ACTIVITY(GO:0008170) |  | 100 | -0.22 | -1.14 | 0.298 | 0.633 | 1.000 | 1027 | tags=4%, list=3%, signal=4% |
| 885 | ION\_HOMEOSTASIS(GO:0050801) |  | 378 | -0.21 | -1.14 | 0.104 | 0.632 | 1.000 | 5936 | tags=19%, list=19%, signal=23% |
| 886 | NUCLEOSIDE\_TRIPHOSPHATE\_BIOSYNTHETIC\_PROCESS(GO:0009142) |  | 99 | -0.26 | -1.14 | 0.370 | 0.634 | 1.000 | 9421 | tags=37%, list=30%, signal=53% |
| 887 | CELLULAR\_AROMATIC\_COMPOUND\_METABOLIC\_PROCESS(GO:0006725) |  | 4003 | -0.16 | -1.14 | 0.215 | 0.633 | 1.000 | 6532 | tags=17%, list=20%, signal=19% |
| 888 | PEPTIDASE\_COMPLEX(GO:1905368) |  | 76 | -0.23 | -1.14 | 0.307 | 0.633 | 1.000 | 2107 | tags=8%, list=7%, signal=8% |
| 889 | CELL\_ADHESION\_MEDIATOR\_ACTIVITY(GO:0098631) |  | 21 | -0.31 | -1.14 | 0.339 | 0.633 | 1.000 | 5270 | tags=19%, list=17%, signal=23% |
| 890 | GLANDULAR\_EPITHELIAL\_CELL\_DIFFERENTIATION(GO:0002067) |  | 32 | -0.32 | -1.14 | 0.248 | 0.633 | 1.000 | 5634 | tags=25%, list=18%, signal=30% |
| 891 | ENTEROENDOCRINE\_CELL\_DIFFERENTIATION(GO:0035883) |  | 32 | -0.32 | -1.14 | 0.248 | 0.632 | 1.000 | 5634 | tags=25%, list=18%, signal=30% |
| 892 | CELL\_PROLIFERATION(GO:0008283) |  | 391 | -0.19 | -1.14 | 0.105 | 0.632 | 1.000 | 4394 | tags=13%, list=14%, signal=14% |
| 893 | CELL\_CYCLE\_G1\_S\_PHASE\_TRANSITION(GO:0044843) |  | 45 | -0.27 | -1.14 | 0.261 | 0.633 | 1.000 | 4370 | tags=13%, list=14%, signal=15% |
| 894 | CARBOHYDRATE\_DERIVATIVE\_METABOLIC\_PROCESS(GO:1901135) |  | 749 | -0.20 | -1.14 | 0.223 | 0.633 | 1.000 | 6520 | tags=21%, list=20%, signal=26% |
| 895 | GDP\_BINDING(GO:0019003) |  | 28 | -0.39 | -1.14 | 0.383 | 0.632 | 1.000 | 6555 | tags=36%, list=21%, signal=45% |
| 896 | NUCLEOBASE\_CONTAINING\_COMPOUND\_METABOLIC\_PROCESS(GO:0006139) |  | 3885 | -0.16 | -1.14 | 0.217 | 0.632 | 1.000 | 6227 | tags=16%, list=20%, signal=17% |
| 897 | FATTY\_ACID\_DERIVATIVE\_BIOSYNTHETIC\_PROCESS(GO:1901570) |  | 32 | -0.37 | -1.14 | 0.312 | 0.632 | 1.000 | 2610 | tags=19%, list=8%, signal=20% |
| 898 | RELEASE\_OF\_SEQUESTERED\_CALCIUM\_ION\_INTO\_CYTOSOL(GO:0051209) |  | 23 | -0.32 | -1.14 | 0.291 | 0.632 | 1.000 | 5936 | tags=35%, list=19%, signal=43% |
| 899 | INTERSTRAND\_CROSS\_LINK\_REPAIR(GO:0036297) |  | 25 | -0.37 | -1.14 | 0.300 | 0.632 | 1.000 | 442 | tags=8%, list=1%, signal=8% |
| 900 | REGULATION\_OF\_DEVELOPMENTAL\_GROWTH(GO:0048638) |  | 105 | -0.25 | -1.14 | 0.257 | 0.631 | 1.000 | 5618 | tags=17%, list=18%, signal=21% |
| 901 | CELLULAR\_NITROGEN\_COMPOUND\_METABOLIC\_PROCESS(GO:0034641) |  | 4460 | -0.15 | -1.14 | 0.191 | 0.631 | 1.000 | 6532 | tags=17%, list=20%, signal=18% |
| 902 | SECONDARY\_ALCOHOL\_METABOLIC\_PROCESS(GO:1902652) |  | 61 | -0.32 | -1.13 | 0.279 | 0.632 | 1.000 | 3229 | tags=18%, list=10%, signal=20% |
| 903 | B\_CELL\_ACTIVATION(GO:0042113) |  | 29 | -0.31 | -1.13 | 0.288 | 0.633 | 1.000 | 9514 | tags=38%, list=30%, signal=54% |
| 904 | GPI\_ANCHOR\_METABOLIC\_PROCESS(GO:0006505) |  | 30 | -0.34 | -1.13 | 0.247 | 0.633 | 1.000 | 3226 | tags=10%, list=10%, signal=11% |
| 905 | DNA\_REPLICATION\_INITIATION(GO:0006270) |  | 23 | -0.29 | -1.13 | 0.267 | 0.635 | 1.000 | 2154 | tags=9%, list=7%, signal=9% |
| 906 | PROTEIN\_BINDING(GO:0005515) |  | 3681 | -0.17 | -1.13 | 0.230 | 0.635 | 1.000 | 6729 | tags=18%, list=21%, signal=20% |
| 907 | CELL\_LEADING\_EDGE(GO:0031252) |  | 130 | -0.22 | -1.13 | 0.278 | 0.638 | 1.000 | 5629 | tags=15%, list=18%, signal=19% |
| 908 | HEART\_DEVELOPMENT(GO:0007507) |  | 539 | -0.19 | -1.13 | 0.223 | 0.638 | 1.000 | 6302 | tags=18%, list=20%, signal=22% |
| 909 | POSITIVE\_REGULATION\_OF\_SMALL\_MOLECULE\_METABOLIC\_PROCESS(GO:0062013) |  | 16 | -0.34 | -1.13 | 0.331 | 0.637 | 1.000 | 7779 | tags=44%, list=24%, signal=58% |
| 910 | RESPONSE\_TO\_AMMONIUM\_ION(GO:0060359) |  | 19 | -0.44 | -1.13 | 0.268 | 0.637 | 1.000 | 4810 | tags=26%, list=15%, signal=31% |
| 911 | SECRETION\_BY\_CELL(GO:0032940) |  | 252 | -0.24 | -1.13 | 0.139 | 0.637 | 1.000 | 6704 | tags=24%, list=21%, signal=30% |
| 912 | ORGANIC\_CYCLIC\_COMPOUND\_METABOLIC\_PROCESS(GO:1901360) |  | 4117 | -0.16 | -1.13 | 0.218 | 0.638 | 1.000 | 6532 | tags=17%, list=20%, signal=19% |
| 913 | LOCOMOTORY\_BEHAVIOR(GO:0007626) |  | 98 | -0.26 | -1.13 | 0.363 | 0.638 | 1.000 | 1059 | tags=5%, list=3%, signal=5% |
| 914 | PURINE\_CONTAINING\_COMPOUND\_METABOLIC\_PROCESS(GO:0072521) |  | 292 | -0.20 | -1.13 | 0.256 | 0.637 | 1.000 | 6789 | tags=22%, list=21%, signal=28% |
| 915 | REGULATION\_OF\_NOTCH\_SIGNALING\_PATHWAY(GO:0008593) |  | 55 | -0.22 | -1.13 | 0.252 | 0.638 | 1.000 | 3956 | tags=13%, list=12%, signal=15% |
| 916 | OXIDOREDUCTASE\_ACTIVITY\_\_ACTING\_ON\_A\_SULFUR\_GROUP\_OF\_DONORS(GO:0016667) |  | 47 | -0.24 | -1.13 | 0.265 | 0.637 | 1.000 | 5648 | tags=26%, list=18%, signal=31% |
| 917 | SPINDLE\_ORGANIZATION(GO:0007051) |  | 86 | -0.22 | -1.13 | 0.203 | 0.637 | 1.000 | 3830 | tags=9%, list=12%, signal=11% |
| 918 | HETEROCYCLE\_METABOLIC\_PROCESS(GO:0046483) |  | 3986 | -0.16 | -1.13 | 0.215 | 0.641 | 1.000 | 6227 | tags=16%, list=20%, signal=17% |
| 919 | DIGESTION(GO:0007586) |  | 26 | -0.31 | -1.13 | 0.360 | 0.642 | 1.000 | 3710 | tags=23%, list=12%, signal=26% |
| 920 | VESICLE\_MEMBRANE(GO:0012506) |  | 185 | -0.22 | -1.13 | 0.305 | 0.642 | 1.000 | 6894 | tags=24%, list=22%, signal=30% |
| 921 | NCRNA\_PROCESSING(GO:0034470) |  | 271 | -0.23 | -1.13 | 0.375 | 0.642 | 1.000 | 9571 | tags=35%, list=30%, signal=49% |
| 922 | REGULATION\_OF\_CELLULAR\_COMPONENT\_ORGANIZATION(GO:0051128) |  | 828 | -0.17 | -1.13 | 0.202 | 0.641 | 1.000 | 5919 | tags=15%, list=19%, signal=18% |
| 923 | INORGANIC\_CATION\_IMPORT\_ACROSS\_PLASMA\_MEMBRANE(GO:0098659) |  | 81 | -0.31 | -1.13 | 0.218 | 0.642 | 1.000 | 7953 | tags=31%, list=25%, signal=41% |
| 924 | INORGANIC\_ION\_IMPORT\_ACROSS\_PLASMA\_MEMBRANE(GO:0099587) |  | 81 | -0.31 | -1.13 | 0.218 | 0.641 | 1.000 | 7953 | tags=31%, list=25%, signal=41% |
| 925 | CELL\_MORPHOGENESIS(GO:0000902) |  | 644 | -0.20 | -1.12 | 0.291 | 0.641 | 1.000 | 6888 | tags=19%, list=22%, signal=24% |
| 926 | G1\_S\_TRANSITION\_OF\_MITOTIC\_CELL\_CYCLE(GO:0000082) |  | 43 | -0.28 | -1.12 | 0.279 | 0.642 | 1.000 | 4370 | tags=14%, list=14%, signal=16% |
| 927 | NUCLEIC\_ACID\_METABOLIC\_PROCESS(GO:0090304) |  | 3472 | -0.16 | -1.12 | 0.245 | 0.642 | 1.000 | 6147 | tags=15%, list=19%, signal=17% |
| 928 | POTASSIUM\_ION\_TRANSPORT(GO:0006813) |  | 183 | -0.25 | -1.12 | 0.117 | 0.646 | 1.000 | 4614 | tags=16%, list=14%, signal=19% |
| 929 | ORGANELLE\_ORGANIZATION(GO:0006996) |  | 2344 | -0.14 | -1.12 | 0.231 | 0.646 | 1.000 | 6873 | tags=17%, list=22%, signal=20% |
| 930 | PURINE\_RIBONUCLEOSIDE\_TRIPHOSPHATE\_METABOLIC\_PROCESS(GO:0009205) |  | 154 | -0.24 | -1.12 | 0.329 | 0.646 | 1.000 | 9801 | tags=38%, list=31%, signal=55% |
| 931 | PHOSPHATASE\_REGULATOR\_ACTIVITY(GO:0019208) |  | 75 | -0.21 | -1.12 | 0.182 | 0.647 | 1.000 | 6380 | tags=23%, list=20%, signal=28% |
| 932 | S\_METHYLTRANSFERASE\_ACTIVITY(GO:0008172) |  | 39 | -0.32 | -1.12 | 0.368 | 0.647 | 1.000 | 6424 | tags=28%, list=20%, signal=35% |
| 933 | CATALYTIC\_COMPLEX(GO:1902494) |  | 1030 | -0.16 | -1.12 | 0.294 | 0.648 | 1.000 | 4303 | tags=10%, list=13%, signal=11% |
| 934 | CIS\_TRANS\_ISOMERASE\_ACTIVITY(GO:0016859) |  | 45 | -0.31 | -1.12 | 0.360 | 0.647 | 1.000 | 7611 | tags=27%, list=24%, signal=35% |
| 935 | GPI\_ANCHOR\_BIOSYNTHETIC\_PROCESS(GO:0006506) |  | 28 | -0.35 | -1.12 | 0.231 | 0.647 | 1.000 | 3226 | tags=11%, list=10%, signal=12% |
| 936 | EPITHELIAL\_CELL\_DIFFERENTIATION(GO:0030855) |  | 252 | -0.21 | -1.12 | 0.242 | 0.647 | 1.000 | 5853 | tags=17%, list=18%, signal=20% |
| 937 | NEGATIVE\_REGULATION\_OF\_HEMOPOIESIS(GO:1903707) |  | 18 | -0.35 | -1.12 | 0.281 | 0.646 | 1.000 | 4370 | tags=22%, list=14%, signal=26% |
| 938 | A\_BAND(GO:0031672) |  | 16 | -0.36 | -1.12 | 0.330 | 0.646 | 1.000 | 4703 | tags=19%, list=15%, signal=22% |
| 939 | REGULATION\_OF\_PLASMA\_MEMBRANE\_BOUNDED\_CELL\_PROJECTION\_ASSEMBLY(GO:0120032) |  | 58 | -0.28 | -1.12 | 0.298 | 0.647 | 1.000 | 4083 | tags=16%, list=13%, signal=18% |
| 940 | CELL\_PROJECTION\_ORGANIZATION(GO:0030030) |  | 943 | -0.18 | -1.12 | 0.224 | 0.648 | 1.000 | 6888 | tags=18%, list=22%, signal=22% |
| 941 | CELLULAR\_RESPONSE\_TO\_BIOTIC\_STIMULUS(GO:0071216) |  | 28 | -0.37 | -1.12 | 0.316 | 0.648 | 1.000 | 3530 | tags=21%, list=11%, signal=24% |
| 942 | GLOMERULUS\_DEVELOPMENT(GO:0032835) |  | 61 | -0.23 | -1.12 | 0.341 | 0.648 | 1.000 | 3956 | tags=10%, list=12%, signal=11% |
| 943 | VASCULATURE\_DEVELOPMENT(GO:0001944) |  | 523 | -0.19 | -1.12 | 0.322 | 0.649 | 1.000 | 4471 | tags=12%, list=14%, signal=14% |
| 944 | CARDIOVASCULAR\_SYSTEM\_DEVELOPMENT(GO:0072358) |  | 523 | -0.19 | -1.12 | 0.322 | 0.649 | 1.000 | 4471 | tags=12%, list=14%, signal=14% |
| 945 | HEMATOPOIETIC\_STEM\_CELL\_DIFFERENTIATION(GO:0060218) |  | 81 | -0.23 | -1.12 | 0.329 | 0.648 | 1.000 | 6090 | tags=22%, list=19%, signal=27% |
| 946 | PROTEIN\_SERINE\_THREONINE\_KINASE\_INHIBITOR\_ACTIVITY(GO:0030291) |  | 18 | -0.33 | -1.12 | 0.354 | 0.649 | 1.000 | 7406 | tags=33%, list=23%, signal=43% |
| 947 | IDENTICAL\_PROTEIN\_BINDING(GO:0042802) |  | 162 | -0.23 | -1.12 | 0.287 | 0.649 | 1.000 | 6072 | tags=20%, list=19%, signal=24% |
| 948 | REGULATION\_OF\_ORGANELLE\_ASSEMBLY(GO:1902115) |  | 50 | -0.23 | -1.12 | 0.354 | 0.650 | 1.000 | 3780 | tags=14%, list=12%, signal=16% |
| 949 | ANATOMICAL\_STRUCTURE\_MORPHOGENESIS(GO:0009653) |  | 2108 | -0.17 | -1.11 | 0.235 | 0.652 | 1.000 | 6303 | tags=17%, list=20%, signal=19% |
| 950 | CELLULAR\_SODIUM\_ION\_HOMEOSTASIS(GO:0006883) |  | 15 | -0.34 | -1.11 | 0.305 | 0.652 | 1.000 | 1842 | tags=13%, list=6%, signal=14% |
| 951 | CELLULAR\_POTASSIUM\_ION\_HOMEOSTASIS(GO:0030007) |  | 15 | -0.34 | -1.11 | 0.305 | 0.651 | 1.000 | 1842 | tags=13%, list=6%, signal=14% |
| 952 | SODIUM\_ION\_EXPORT\_ACROSS\_PLASMA\_MEMBRANE(GO:0036376) |  | 15 | -0.34 | -1.11 | 0.305 | 0.651 | 1.000 | 1842 | tags=13%, list=6%, signal=14% |
| 953 | CHOLESTEROL\_METABOLIC\_PROCESS(GO:0008203) |  | 60 | -0.32 | -1.11 | 0.282 | 0.650 | 1.000 | 3229 | tags=18%, list=10%, signal=20% |
| 954 | REGULATION\_OF\_ORGANELLE\_ORGANIZATION(GO:0033043) |  | 487 | -0.17 | -1.11 | 0.194 | 0.651 | 1.000 | 6290 | tags=17%, list=20%, signal=21% |
| 955 | CELLULAR\_RESPONSE\_TO\_STIMULUS(GO:0051716) |  | 4234 | -0.17 | -1.11 | 0.134 | 0.652 | 1.000 | 4449 | tags=12%, list=14%, signal=12% |
| 956 | HISTONE\_H2A\_UBIQUITINATION(GO:0033522) |  | 15 | -0.32 | -1.11 | 0.270 | 0.655 | 1.000 | 10335 | tags=40%, list=32%, signal=59% |
| 957 | RESPONSE\_TO\_ANTIBIOTIC(GO:0046677) |  | 30 | -0.29 | -1.11 | 0.350 | 0.655 | 1.000 | 5141 | tags=20%, list=16%, signal=24% |
| 958 | SIGNALING\_ADAPTOR\_ACTIVITY(GO:0035591) |  | 24 | -0.33 | -1.11 | 0.347 | 0.655 | 1.000 | 7217 | tags=29%, list=23%, signal=38% |
| 959 | INSULIN\_RECEPTOR\_SIGNALING\_PATHWAY(GO:0008286) |  | 30 | -0.31 | -1.11 | 0.281 | 0.654 | 1.000 | 968 | tags=7%, list=3%, signal=7% |
| 960 | GENE\_EXPRESSION(GO:0010467) |  | 3555 | -0.16 | -1.11 | 0.217 | 0.656 | 1.000 | 6532 | tags=16%, list=20%, signal=18% |
| 961 | BIOSYNTHETIC\_PROCESS(GO:0009058) |  | 4030 | -0.16 | -1.11 | 0.253 | 0.655 | 1.000 | 6532 | tags=17%, list=20%, signal=19% |
| 962 | HORMONE\_METABOLIC\_PROCESS(GO:0042445) |  | 68 | -0.29 | -1.11 | 0.257 | 0.657 | 1.000 | 3151 | tags=15%, list=10%, signal=16% |
| 963 | ZYMOGEN\_ACTIVATION(GO:0031638) |  | 17 | -0.45 | -1.11 | 0.327 | 0.658 | 1.000 | 4361 | tags=29%, list=14%, signal=34% |
| 964 | CELLULAR\_RESPONSE\_TO\_MOLECULE\_OF\_BACTERIAL\_ORIGIN(GO:0071219) |  | 26 | -0.38 | -1.11 | 0.341 | 0.659 | 1.000 | 3530 | tags=23%, list=11%, signal=26% |
| 965 | EMBRYONIC\_VISCEROCRANIUM\_MORPHOGENESIS(GO:0048703) |  | 107 | -0.22 | -1.11 | 0.344 | 0.658 | 1.000 | 6421 | tags=18%, list=20%, signal=22% |
| 966 | DNA\_TEMPLATED\_TRANSCRIPTION\_\_INITIATION(GO:0006352) |  | 51 | -0.28 | -1.11 | 0.395 | 0.660 | 1.000 | 5427 | tags=22%, list=17%, signal=26% |
| 967 | HEPARAN\_SULFATE\_SULFOTRANSFERASE\_ACTIVITY(GO:0034483) |  | 57 | -0.27 | -1.11 | 0.253 | 0.660 | 1.000 | 5477 | tags=21%, list=17%, signal=25% |
| 968 | NCRNA\_METABOLIC\_PROCESS(GO:0034660) |  | 346 | -0.21 | -1.11 | 0.321 | 0.660 | 1.000 | 9571 | tags=35%, list=30%, signal=49% |
| 969 | CELLULAR\_CATION\_HOMEOSTASIS(GO:0030003) |  | 294 | -0.22 | -1.11 | 0.251 | 0.660 | 1.000 | 5936 | tags=19%, list=19%, signal=24% |
| 970 | NON\_MEMBRANE\_BOUNDED\_ORGANELLE(GO:0043228) |  | 2221 | -0.15 | -1.11 | 0.255 | 0.660 | 1.000 | 6873 | tags=18%, list=22%, signal=22% |
| 971 | INTRACELLULAR\_NON\_MEMBRANE\_BOUNDED\_ORGANELLE(GO:0043232) |  | 2221 | -0.15 | -1.11 | 0.255 | 0.659 | 1.000 | 6873 | tags=18%, list=22%, signal=22% |
| 972 | NUCLEOSIDE\_DIPHOSPHATE\_METABOLIC\_PROCESS(GO:0009132) |  | 73 | -0.29 | -1.10 | 0.433 | 0.662 | 1.000 | 4213 | tags=18%, list=13%, signal=20% |
| 973 | PURINE\_NUCLEOSIDE\_MONOPHOSPHATE\_METABOLIC\_PROCESS(GO:0009126) |  | 169 | -0.21 | -1.10 | 0.370 | 0.662 | 1.000 | 9801 | tags=37%, list=31%, signal=54% |
| 974 | PURINE\_RIBONUCLEOSIDE\_MONOPHOSPHATE\_METABOLIC\_PROCESS(GO:0009167) |  | 169 | -0.21 | -1.10 | 0.370 | 0.662 | 1.000 | 9801 | tags=37%, list=31%, signal=54% |
| 975 | CELL\_DEATH(GO:0008219) |  | 578 | -0.18 | -1.10 | 0.215 | 0.662 | 1.000 | 4370 | tags=12%, list=14%, signal=13% |
| 976 | REGULATION\_OF\_CHEMOTAXIS(GO:0050920) |  | 72 | -0.25 | -1.10 | 0.277 | 0.663 | 1.000 | 4402 | tags=15%, list=14%, signal=18% |
| 977 | PYRUVATE\_BIOSYNTHETIC\_PROCESS(GO:0042866) |  | 41 | -0.34 | -1.10 | 0.339 | 0.663 | 1.000 | 8735 | tags=39%, list=27%, signal=54% |
| 978 | PIGMENTATION(GO:0043473) |  | 138 | -0.24 | -1.10 | 0.264 | 0.664 | 1.000 | 6429 | tags=23%, list=20%, signal=29% |
| 979 | MISMATCH\_REPAIR(GO:0006298) |  | 15 | -0.39 | -1.10 | 0.325 | 0.663 | 1.000 | 92 | tags=7%, list=0%, signal=7% |
| 980 | CARBOHYDRATE\_DERIVATIVE\_BIOSYNTHETIC\_PROCESS(GO:1901137) |  | 488 | -0.20 | -1.10 | 0.309 | 0.664 | 1.000 | 6381 | tags=20%, list=20%, signal=25% |
| 981 | GLUTAMATERGIC\_SYNAPSE(GO:0098978) |  | 22 | -0.41 | -1.10 | 0.281 | 0.665 | 1.000 | 8945 | tags=41%, list=28%, signal=57% |
| 982 | PEROXISOME\_ORGANIZATION(GO:0007031) |  | 22 | -0.30 | -1.10 | 0.260 | 0.664 | 1.000 | 2519 | tags=9%, list=8%, signal=10% |
| 983 | MITOCHONDRIAL\_TRANSMEMBRANE\_TRANSPORT(GO:1990542) |  | 79 | -0.24 | -1.10 | 0.333 | 0.664 | 1.000 | 6343 | tags=23%, list=20%, signal=28% |
| 984 | EMBRYONIC\_SKELETAL\_SYSTEM\_DEVELOPMENT(GO:0048706) |  | 195 | -0.20 | -1.10 | 0.229 | 0.665 | 1.000 | 6543 | tags=17%, list=21%, signal=21% |
| 985 | ATP\_BIOSYNTHETIC\_PROCESS(GO:0006754) |  | 69 | -0.27 | -1.10 | 0.380 | 0.668 | 1.000 | 9801 | tags=41%, list=31%, signal=58% |
| 986 | REGULATION\_OF\_CELL\_PROJECTION\_ASSEMBLY(GO:0060491) |  | 59 | -0.28 | -1.10 | 0.352 | 0.668 | 1.000 | 4083 | tags=15%, list=13%, signal=17% |
| 987 | PHOSPHATASE\_BINDING(GO:0019902) |  | 48 | -0.26 | -1.10 | 0.275 | 0.667 | 1.000 | 541 | tags=4%, list=2%, signal=4% |
| 988 | CYTOPLASMIC\_VESICLE\_MEMBRANE(GO:0030659) |  | 182 | -0.21 | -1.10 | 0.384 | 0.667 | 1.000 | 6894 | tags=23%, list=22%, signal=29% |
| 989 | P\_BODY(GO:0000932) |  | 51 | -0.22 | -1.10 | 0.251 | 0.668 | 1.000 | 2968 | tags=12%, list=9%, signal=13% |
| 990 | OXIDOREDUCTASE\_ACTIVITY\_\_ACTING\_ON\_PAIRED\_DONORS\_\_WITH\_INCORPORATION\_OR\_REDUCTION\_OF\_MOLECULAR\_OXYGEN\_\_2\_OXOGLUTARATE\_AS\_ONE\_DONOR\_\_AND\_INCORPORATION\_OF\_ONE\_ATOM\_EACH\_OF\_OXYGEN\_INTO\_BOTH\_DONORS(GO:0016706) |  | 45 | -0.25 | -1.10 | 0.254 | 0.668 | 1.000 | 5917 | tags=24%, list=19%, signal=30% |
| 991 | REGULATION\_OF\_I\_KAPPAB\_KINASE\_NF\_KAPPAB\_SIGNALING(GO:0043122) |  | 53 | -0.24 | -1.10 | 0.383 | 0.669 | 1.000 | 4405 | tags=15%, list=14%, signal=17% |
| 992 | METALLOENDOPEPTIDASE\_ACTIVITY(GO:0004222) |  | 101 | -0.24 | -1.10 | 0.321 | 0.668 | 1.000 | 5821 | tags=23%, list=18%, signal=28% |
| 993 | GLYCOPROTEIN\_CATABOLIC\_PROCESS(GO:0006516) |  | 16 | -0.34 | -1.10 | 0.280 | 0.668 | 1.000 | 4466 | tags=31%, list=14%, signal=36% |
| 994 | STRESS\_FIBER\_ASSEMBLY(GO:0043149) |  | 21 | -0.38 | -1.10 | 0.372 | 0.667 | 1.000 | 8089 | tags=48%, list=25%, signal=64% |
| 995 | CONTRACTILE\_ACTIN\_FILAMENT\_BUNDLE\_ASSEMBLY(GO:0030038) |  | 21 | -0.38 | -1.10 | 0.372 | 0.666 | 1.000 | 8089 | tags=48%, list=25%, signal=64% |
| 996 | RESPONSE\_TO\_GLUCOSE(GO:0009749) |  | 19 | -0.35 | -1.10 | 0.416 | 0.666 | 1.000 | 4800 | tags=26%, list=15%, signal=31% |
| 997 | NEUROPEPTIDE\_HORMONE\_ACTIVITY(GO:0005184) |  | 25 | -0.36 | -1.10 | 0.356 | 0.667 | 1.000 | 10773 | tags=52%, list=34%, signal=78% |
| 998 | GLYCOLYTIC\_PROCESS(GO:0006096) |  | 40 | -0.34 | -1.10 | 0.339 | 0.666 | 1.000 | 4213 | tags=20%, list=13%, signal=23% |
| 999 | STEROL\_BINDING(GO:0032934) |  | 36 | -0.26 | -1.10 | 0.311 | 0.666 | 1.000 | 3236 | tags=17%, list=10%, signal=19% |
| 1000 | REGULATION\_OF\_SISTER\_CHROMATID\_SEGREGATION(GO:0033045) |  | 34 | -0.35 | -1.10 | 0.402 | 0.666 | 1.000 | 2491 | tags=12%, list=8%, signal=13% |
| 1001 | REGULATION\_OF\_ACTIN\_FILAMENT\_ORGANIZATION(GO:0110053) |  | 155 | -0.21 | -1.10 | 0.222 | 0.666 | 1.000 | 7402 | tags=23%, list=23%, signal=30% |
| 1002 | ACTION\_POTENTIAL(GO:0001508) |  | 42 | -0.31 | -1.10 | 0.297 | 0.666 | 1.000 | 8520 | tags=38%, list=27%, signal=52% |
| 1003 | SPERMATID\_DEVELOPMENT(GO:0007286) |  | 22 | -0.35 | -1.10 | 0.284 | 0.665 | 1.000 | 1026 | tags=9%, list=3%, signal=9% |
| 1004 | HORMONE\_SECRETION(GO:0046879) |  | 38 | -0.35 | -1.09 | 0.309 | 0.666 | 1.000 | 4587 | tags=18%, list=14%, signal=21% |
| 1005 | ENDOSOME\_ORGANIZATION(GO:0007032) |  | 17 | -0.29 | -1.09 | 0.327 | 0.667 | 1.000 | 12553 | tags=41%, list=39%, signal=68% |
| 1006 | REGULATION\_OF\_HORMONE\_LEVELS(GO:0010817) |  | 106 | -0.29 | -1.09 | 0.283 | 0.669 | 1.000 | 4587 | tags=17%, list=14%, signal=20% |
| 1007 | SPERMATID\_DIFFERENTIATION(GO:0048515) |  | 23 | -0.33 | -1.09 | 0.290 | 0.671 | 1.000 | 1026 | tags=9%, list=3%, signal=9% |
| 1008 | ORGANIC\_SUBSTANCE\_BIOSYNTHETIC\_PROCESS(GO:1901576) |  | 3987 | -0.15 | -1.09 | 0.249 | 0.671 | 1.000 | 6532 | tags=17%, list=20%, signal=19% |
| 1009 | REGULATION\_OF\_GROWTH(GO:0040008) |  | 150 | -0.23 | -1.09 | 0.317 | 0.671 | 1.000 | 5618 | tags=17%, list=18%, signal=20% |
| 1010 | ESTABLISHMENT\_OF\_MITOTIC\_SPINDLE\_ORIENTATION(GO:0000132) |  | 19 | -0.32 | -1.09 | 0.360 | 0.671 | 1.000 | 310 | tags=5%, list=1%, signal=5% |
| 1011 | RIBONUCLEOSIDE\_TRIPHOSPHATE\_METABOLIC\_PROCESS(GO:0009199) |  | 163 | -0.23 | -1.09 | 0.369 | 0.671 | 1.000 | 9107 | tags=34%, list=29%, signal=48% |
| 1012 | REGULATION\_OF\_ACTIN\_FILAMENT\_POLYMERIZATION(GO:0030833) |  | 136 | -0.20 | -1.09 | 0.329 | 0.671 | 1.000 | 5213 | tags=15%, list=16%, signal=18% |
| 1013 | CELLULAR\_BIOSYNTHETIC\_PROCESS(GO:0044249) |  | 3918 | -0.15 | -1.09 | 0.250 | 0.671 | 1.000 | 6532 | tags=17%, list=20%, signal=19% |
| 1014 | GLYCOPROTEIN\_BIOSYNTHETIC\_PROCESS(GO:0009101) |  | 233 | -0.22 | -1.09 | 0.336 | 0.672 | 1.000 | 6132 | tags=18%, list=19%, signal=23% |
| 1015 | PURINE\_NUCLEOTIDE\_BIOSYNTHETIC\_PROCESS(GO:0006164) |  | 164 | -0.20 | -1.09 | 0.303 | 0.672 | 1.000 | 6379 | tags=22%, list=20%, signal=27% |
| 1016 | CYTOSOLIC\_RIBOSOME(GO:0022626) |  | 78 | -0.36 | -1.09 | 0.324 | 0.672 | 1.000 | 11045 | tags=44%, list=35%, signal=67% |
| 1017 | HISTONE\_METHYLTRANSFERASE\_ACTIVITY(GO:0042054) |  | 46 | -0.26 | -1.09 | 0.371 | 0.673 | 1.000 | 123 | tags=2%, list=0%, signal=2% |
| 1018 | RHO\_PROTEIN\_SIGNAL\_TRANSDUCTION(GO:0007266) |  | 101 | -0.26 | -1.09 | 0.331 | 0.673 | 1.000 | 6123 | tags=22%, list=19%, signal=27% |
| 1019 | PROTEIN\_KINASE\_A\_BINDING(GO:0051018) |  | 26 | -0.28 | -1.09 | 0.411 | 0.673 | 1.000 | 9807 | tags=31%, list=31%, signal=44% |
| 1020 | PLATELET\_ACTIVATION(GO:0030168) |  | 18 | -0.40 | -1.09 | 0.337 | 0.675 | 1.000 | 5422 | tags=28%, list=17%, signal=33% |
| 1021 | REGULATION\_OF\_G\_PROTEIN\_COUPLED\_RECEPTOR\_PROTEIN\_SIGNALING\_PATHWAY(GO:0008277) |  | 32 | -0.40 | -1.09 | 0.338 | 0.677 | 1.000 | 5264 | tags=34%, list=17%, signal=41% |
| 1022 | CELLULAR\_RESPONSE\_TO\_ORGANIC\_CYCLIC\_COMPOUND(GO:0071407) |  | 67 | -0.24 | -1.09 | 0.379 | 0.676 | 1.000 | 2792 | tags=12%, list=9%, signal=13% |
| 1023 | CHOLESTEROL\_BINDING(GO:0015485) |  | 29 | -0.30 | -1.09 | 0.311 | 0.676 | 1.000 | 3236 | tags=21%, list=10%, signal=23% |
| 1024 | CIRCULATORY\_SYSTEM\_DEVELOPMENT(GO:0072359) |  | 976 | -0.17 | -1.09 | 0.316 | 0.676 | 1.000 | 4475 | tags=12%, list=14%, signal=13% |
| 1025 | BOUNDING\_MEMBRANE\_OF\_ORGANELLE(GO:0098588) |  | 891 | -0.16 | -1.08 | 0.266 | 0.677 | 1.000 | 5953 | tags=15%, list=19%, signal=18% |
| 1026 | NUCLEOPLASM(GO:0005654) |  | 648 | -0.15 | -1.08 | 0.338 | 0.677 | 1.000 | 7074 | tags=18%, list=22%, signal=23% |
| 1027 | SIALYLTRANSFERASE\_ACTIVITY(GO:0008373) |  | 29 | -0.33 | -1.08 | 0.318 | 0.676 | 1.000 | 5221 | tags=24%, list=16%, signal=29% |
| 1028 | ORGANELLE\_PART(GO:0044422) |  | 4431 | -0.15 | -1.08 | 0.260 | 0.676 | 1.000 | 6446 | tags=16%, list=20%, signal=17% |
| 1029 | EPITHELIUM\_DEVELOPMENT(GO:0060429) |  | 870 | -0.18 | -1.08 | 0.288 | 0.676 | 1.000 | 6039 | tags=16%, list=19%, signal=19% |
| 1030 | ESTABLISHMENT\_OF\_MITOTIC\_SPINDLE\_LOCALIZATION(GO:0040001) |  | 24 | -0.29 | -1.08 | 0.351 | 0.677 | 1.000 | 310 | tags=4%, list=1%, signal=4% |
| 1031 | RNA\_METABOLIC\_PROCESS(GO:0016070) |  | 3064 | -0.16 | -1.08 | 0.301 | 0.677 | 1.000 | 6145 | tags=15%, list=19%, signal=17% |
| 1032 | COENZYME\_METABOLIC\_PROCESS(GO:0006732) |  | 218 | -0.22 | -1.08 | 0.330 | 0.678 | 1.000 | 6219 | tags=22%, list=20%, signal=28% |
| 1033 | MORPHOGENESIS\_OF\_AN\_EPITHELIUM(GO:0002009) |  | 467 | -0.18 | -1.08 | 0.301 | 0.678 | 1.000 | 5018 | tags=13%, list=16%, signal=16% |
| 1034 | TRANSLATIONAL\_TERMINATION(GO:0006415) |  | 15 | -0.29 | -1.08 | 0.333 | 0.679 | 1.000 | 3813 | tags=20%, list=12%, signal=23% |
| 1035 | POSITIVE\_REGULATION\_OF\_PLASMA\_MEMBRANE\_BOUNDED\_CELL\_PROJECTION\_ASSEMBLY(GO:0120034) |  | 26 | -0.33 | -1.08 | 0.234 | 0.679 | 1.000 | 4083 | tags=23%, list=13%, signal=26% |
| 1036 | RESPONSE\_TO\_INSULIN(GO:0032868) |  | 57 | -0.27 | -1.08 | 0.200 | 0.680 | 1.000 | 3951 | tags=16%, list=12%, signal=18% |
| 1037 | TRANSFERASE\_ACTIVITY(GO:0016740) |  | 2377 | -0.16 | -1.08 | 0.231 | 0.680 | 1.000 | 6381 | tags=17%, list=20%, signal=19% |
| 1038 | PHOSPHORIC\_ESTER\_HYDROLASE\_ACTIVITY(GO:0042578) |  | 377 | -0.19 | -1.08 | 0.282 | 0.682 | 1.000 | 4405 | tags=14%, list=14%, signal=16% |
| 1039 | REGULATION\_OF\_CELLULAR\_RESPONSE\_TO\_GROWTH\_FACTOR\_STIMULUS(GO:0090287) |  | 149 | -0.21 | -1.08 | 0.302 | 0.686 | 1.000 | 2562 | tags=7%, list=8%, signal=8% |
| 1040 | CELLULAR\_POTASSIUM\_ION\_TRANSPORT(GO:0071804) |  | 160 | -0.24 | -1.08 | 0.197 | 0.686 | 1.000 | 4468 | tags=16%, list=14%, signal=19% |
| 1041 | POTASSIUM\_ION\_TRANSMEMBRANE\_TRANSPORT(GO:0071805) |  | 160 | -0.24 | -1.08 | 0.197 | 0.685 | 1.000 | 4468 | tags=16%, list=14%, signal=19% |
| 1042 | ESTABLISHMENT\_OF\_SPINDLE\_ORIENTATION(GO:0051294) |  | 21 | -0.31 | -1.08 | 0.364 | 0.685 | 1.000 | 310 | tags=5%, list=1%, signal=5% |
| 1043 | REGULATION\_OF\_RESPONSE\_TO\_STIMULUS(GO:0048583) |  | 1446 | -0.18 | -1.08 | 0.252 | 0.685 | 1.000 | 5470 | tags=15%, list=17%, signal=17% |
| 1044 | TRANSITION\_METAL\_ION\_TRANSMEMBRANE\_TRANSPORTER\_ACTIVITY(GO:0046915) |  | 32 | -0.31 | -1.08 | 0.331 | 0.685 | 1.000 | 5182 | tags=19%, list=16%, signal=22% |
| 1045 | AMINO\_ACID\_ACTIVATION(GO:0043038) |  | 38 | -0.24 | -1.08 | 0.465 | 0.687 | 1.000 | 10351 | tags=53%, list=32%, signal=78% |
| 1046 | TRNA\_AMINOACYLATION(GO:0043039) |  | 38 | -0.24 | -1.08 | 0.465 | 0.686 | 1.000 | 10351 | tags=53%, list=32%, signal=78% |
| 1047 | REGULATION\_OF\_CELLULAR\_COMPONENT\_BIOGENESIS(GO:0044087) |  | 298 | -0.18 | -1.08 | 0.293 | 0.686 | 1.000 | 3801 | tags=10%, list=12%, signal=12% |
| 1048 | METAL\_ION\_HOMEOSTASIS(GO:0055065) |  | 309 | -0.20 | -1.08 | 0.239 | 0.686 | 1.000 | 5936 | tags=19%, list=19%, signal=24% |
| 1049 | REGULATION\_OF\_NEUROTRANSMITTER\_RECEPTOR\_ACTIVITY(GO:0099601) |  | 27 | -0.34 | -1.08 | 0.317 | 0.686 | 1.000 | 11828 | tags=56%, list=37%, signal=88% |
| 1050 | REGULATION\_OF\_SECRETION\_BY\_CELL(GO:1903530) |  | 133 | -0.25 | -1.07 | 0.346 | 0.687 | 1.000 | 9283 | tags=36%, list=29%, signal=51% |
| 1051 | INTRACELLULAR\_ORGANELLE\_PART(GO:0044446) |  | 4251 | -0.14 | -1.07 | 0.260 | 0.687 | 1.000 | 6446 | tags=16%, list=20%, signal=17% |
| 1052 | RECEPTOR\_SIGNALING\_COMPLEX\_SCAFFOLD\_ACTIVITY(GO:0030159) |  | 19 | -0.36 | -1.07 | 0.324 | 0.687 | 1.000 | 7994 | tags=26%, list=25%, signal=35% |
| 1053 | REGULATION\_OF\_ACTIN\_FILAMENT\_LENGTH(GO:0030832) |  | 139 | -0.20 | -1.07 | 0.281 | 0.686 | 1.000 | 5213 | tags=14%, list=16%, signal=17% |
| 1054 | REGULATION\_OF\_ACTIN\_POLYMERIZATION\_OR\_DEPOLYMERIZATION(GO:0008064) |  | 139 | -0.20 | -1.07 | 0.281 | 0.686 | 1.000 | 5213 | tags=14%, list=16%, signal=17% |
| 1055 | PROTEIN\_CONTAINING\_COMPLEX(GO:0032991) |  | 3583 | -0.15 | -1.07 | 0.394 | 0.685 | 1.000 | 6611 | tags=16%, list=21%, signal=18% |
| 1056 | DNA\_DIRECTED\_RNA\_POLYMERASE\_II\_\_HOLOENZYME(GO:0016591) |  | 74 | -0.23 | -1.07 | 0.413 | 0.685 | 1.000 | 6949 | tags=22%, list=22%, signal=28% |
| 1057 | TUBULIN\_BINDING(GO:0015631) |  | 249 | -0.19 | -1.07 | 0.227 | 0.685 | 1.000 | 8746 | tags=28%, list=27%, signal=38% |
| 1058 | CELLULAR\_MACROMOLECULE\_BIOSYNTHETIC\_PROCESS(GO:0034645) |  | 3214 | -0.16 | -1.07 | 0.276 | 0.686 | 1.000 | 6145 | tags=15%, list=19%, signal=17% |
| 1059 | PLASMA\_MEMBRANE\_BOUNDED\_CELL\_PROJECTION\_ORGANIZATION(GO:0120036) |  | 911 | -0.18 | -1.07 | 0.383 | 0.686 | 1.000 | 6888 | tags=18%, list=22%, signal=22% |
| 1060 | PHOSPHOPROTEIN\_PHOSPHATASE\_ACTIVITY(GO:0004721) |  | 180 | -0.18 | -1.07 | 0.241 | 0.687 | 1.000 | 4125 | tags=12%, list=13%, signal=14% |
| 1061 | NEGATIVE\_REGULATION\_OF\_RESPONSE\_TO\_STIMULUS(GO:0048585) |  | 501 | -0.19 | -1.07 | 0.335 | 0.687 | 1.000 | 5222 | tags=15%, list=16%, signal=17% |
| 1062 | REGULATION\_OF\_AXONOGENESIS(GO:0050770) |  | 113 | -0.22 | -1.07 | 0.335 | 0.688 | 1.000 | 3976 | tags=12%, list=12%, signal=13% |
| 1063 | MACROMOLECULE\_DEACYLATION(GO:0098732) |  | 53 | -0.24 | -1.07 | 0.383 | 0.687 | 1.000 | 2931 | tags=11%, list=9%, signal=12% |
| 1064 | MICROTUBULE\_BASED\_MOVEMENT(GO:0007018) |  | 192 | -0.23 | -1.07 | 0.396 | 0.687 | 1.000 | 9307 | tags=33%, list=29%, signal=46% |
| 1065 | PROTEIN\_POLYUBIQUITINATION(GO:0000209) |  | 126 | -0.20 | -1.07 | 0.375 | 0.689 | 1.000 | 10434 | tags=37%, list=33%, signal=54% |
| 1066 | PDZ\_DOMAIN\_BINDING(GO:0030165) |  | 16 | -0.32 | -1.07 | 0.391 | 0.691 | 1.000 | 7205 | tags=25%, list=23%, signal=32% |
| 1067 | REGULATION\_OF\_MYOTUBE\_DIFFERENTIATION(GO:0010830) |  | 15 | -0.39 | -1.07 | 0.334 | 0.694 | 1.000 | 2888 | tags=13%, list=9%, signal=15% |
| 1068 | RESPONSE\_TO\_CARBOHYDRATE(GO:0009743) |  | 20 | -0.33 | -1.07 | 0.413 | 0.696 | 1.000 | 4800 | tags=25%, list=15%, signal=29% |
| 1069 | RESPONSE\_TO\_HEXOSE(GO:0009746) |  | 20 | -0.33 | -1.07 | 0.413 | 0.695 | 1.000 | 4800 | tags=25%, list=15%, signal=29% |
| 1070 | RESPONSE\_TO\_MONOSACCHARIDE(GO:0034284) |  | 20 | -0.33 | -1.07 | 0.413 | 0.694 | 1.000 | 4800 | tags=25%, list=15%, signal=29% |
| 1071 | CELL\_MIGRATION(GO:0016477) |  | 689 | -0.18 | -1.07 | 0.292 | 0.695 | 1.000 | 4471 | tags=12%, list=14%, signal=14% |
| 1072 | MACROMOLECULE\_BIOSYNTHETIC\_PROCESS(GO:0009059) |  | 3224 | -0.16 | -1.07 | 0.306 | 0.695 | 1.000 | 6145 | tags=15%, list=19%, signal=17% |
| 1073 | REGULATION\_OF\_MITOCHONDRION\_ORGANIZATION(GO:0010821) |  | 18 | -0.37 | -1.07 | 0.413 | 0.694 | 1.000 | 7544 | tags=33%, list=24%, signal=44% |
| 1074 | ASYMMETRIC\_SYNAPSE(GO:0032279) |  | 93 | -0.30 | -1.07 | 0.339 | 0.694 | 1.000 | 8983 | tags=40%, list=28%, signal=55% |
| 1075 | NEURON\_TO\_NEURON\_SYNAPSE(GO:0098984) |  | 93 | -0.30 | -1.07 | 0.339 | 0.694 | 1.000 | 8983 | tags=40%, list=28%, signal=55% |
| 1076 | CELLULAR\_ION\_HOMEOSTASIS(GO:0006873) |  | 300 | -0.21 | -1.07 | 0.282 | 0.694 | 1.000 | 5936 | tags=19%, list=19%, signal=23% |
| 1077 | KIDNEY\_MORPHOGENESIS(GO:0060993) |  | 34 | -0.32 | -1.07 | 0.344 | 0.694 | 1.000 | 537 | tags=6%, list=2%, signal=6% |
| 1078 | CHROMOSOME\_LOCALIZATION(GO:0050000) |  | 21 | -0.31 | -1.07 | 0.454 | 0.693 | 1.000 | 2248 | tags=10%, list=7%, signal=10% |
| 1079 | ESTABLISHMENT\_OF\_CHROMOSOME\_LOCALIZATION(GO:0051303) |  | 21 | -0.31 | -1.07 | 0.454 | 0.692 | 1.000 | 2248 | tags=10%, list=7%, signal=10% |
| 1080 | HEPARAN\_SULFATE\_2\_O\_SULFOTRANSFERASE\_ACTIVITY(GO:0004394) |  | 52 | -0.26 | -1.06 | 0.303 | 0.694 | 1.000 | 5477 | tags=19%, list=17%, signal=23% |
| 1081 | POSITIVE\_REGULATION\_OF\_CELL\_CELL\_ADHESION(GO:0022409) |  | 35 | -0.30 | -1.06 | 0.380 | 0.694 | 1.000 | 1287 | tags=14%, list=4%, signal=15% |
| 1082 | NUCLEAR\_PART(GO:0044428) |  | 1357 | -0.14 | -1.06 | 0.348 | 0.695 | 1.000 | 6882 | tags=16%, list=22%, signal=20% |
| 1083 | NUCLEOSIDE\_DIPHOSPHATE\_KINASE\_ACTIVITY(GO:0004550) |  | 19 | -0.38 | -1.06 | 0.382 | 0.694 | 1.000 | 7211 | tags=32%, list=23%, signal=41% |
| 1084 | EMBRYONIC\_CRANIAL\_SKELETON\_MORPHOGENESIS(GO:0048701) |  | 160 | -0.21 | -1.06 | 0.263 | 0.694 | 1.000 | 6543 | tags=19%, list=21%, signal=23% |
| 1085 | SARCOLEMMA(GO:0042383) |  | 46 | -0.24 | -1.06 | 0.345 | 0.694 | 1.000 | 9453 | tags=37%, list=30%, signal=52% |
| 1086 | HORMONE\_ACTIVITY(GO:0005179) |  | 115 | -0.29 | -1.06 | 0.356 | 0.696 | 1.000 | 6824 | tags=27%, list=21%, signal=34% |
| 1087 | NEUTROPHIL\_MIGRATION(GO:1990266) |  | 76 | -0.25 | -1.06 | 0.259 | 0.695 | 1.000 | 3746 | tags=13%, list=12%, signal=15% |
| 1088 | NUCLEOSIDE\_TRIPHOSPHATE\_METABOLIC\_PROCESS(GO:0009141) |  | 175 | -0.22 | -1.06 | 0.366 | 0.697 | 1.000 | 9107 | tags=34%, list=29%, signal=47% |
| 1089 | GTPASE\_BINDING(GO:0051020) |  | 186 | -0.22 | -1.06 | 0.340 | 0.697 | 1.000 | 4191 | tags=12%, list=13%, signal=14% |
| 1090 | PROTEIN\_HETERODIMERIZATION\_ACTIVITY(GO:0046982) |  | 129 | -0.25 | -1.06 | 0.375 | 0.697 | 1.000 | 8092 | tags=30%, list=25%, signal=40% |
| 1091 | CELLULAR\_HOMEOSTASIS(GO:0019725) |  | 371 | -0.19 | -1.06 | 0.235 | 0.697 | 1.000 | 5936 | tags=18%, list=19%, signal=21% |
| 1092 | ATP\_GENERATION\_FROM\_ADP(GO:0006757) |  | 41 | -0.34 | -1.06 | 0.349 | 0.696 | 1.000 | 4213 | tags=20%, list=13%, signal=22% |
| 1093 | ENDOSOME(GO:0005768) |  | 395 | -0.16 | -1.06 | 0.358 | 0.696 | 1.000 | 4229 | tags=11%, list=13%, signal=12% |
| 1094 | CELLULAR\_CHEMICAL\_HOMEOSTASIS(GO:0055082) |  | 316 | -0.20 | -1.06 | 0.276 | 0.696 | 1.000 | 5936 | tags=18%, list=19%, signal=22% |
| 1095 | COLUMNAR\_CUBOIDAL\_EPITHELIAL\_CELL\_DIFFERENTIATION(GO:0002065) |  | 66 | -0.25 | -1.06 | 0.308 | 0.695 | 1.000 | 5852 | tags=18%, list=18%, signal=22% |
| 1096 | REGULATION\_OF\_CYTOSKELETON\_ORGANIZATION(GO:0051493) |  | 305 | -0.17 | -1.06 | 0.312 | 0.695 | 1.000 | 6290 | tags=17%, list=20%, signal=21% |
| 1097 | OXIDOREDUCTASE\_ACTIVITY\_\_ACTING\_ON\_THE\_ALDEHYDE\_OR\_OXO\_GROUP\_OF\_DONORS\_\_NAD\_OR\_NADP\_AS\_ACCEPTOR(GO:0016620) |  | 26 | -0.27 | -1.06 | 0.305 | 0.694 | 1.000 | 17 | tags=4%, list=0%, signal=4% |
| 1098 | H4\_HISTONE\_ACETYLTRANSFERASE\_COMPLEX(GO:1902562) |  | 26 | -0.31 | -1.06 | 0.382 | 0.694 | 1.000 | 11281 | tags=38%, list=35%, signal=59% |
| 1099 | SULFUR\_COMPOUND\_METABOLIC\_PROCESS(GO:0006790) |  | 208 | -0.23 | -1.06 | 0.341 | 0.697 | 1.000 | 5851 | tags=21%, list=18%, signal=26% |
| 1100 | PYRIDOXAL\_PHOSPHATE\_BINDING(GO:0030170) |  | 50 | -0.25 | -1.06 | 0.280 | 0.699 | 1.000 | 5715 | tags=20%, list=18%, signal=24% |
| 1101 | VITAMIN\_B6\_BINDING(GO:0070279) |  | 50 | -0.25 | -1.06 | 0.280 | 0.699 | 1.000 | 5715 | tags=20%, list=18%, signal=24% |
| 1102 | MYOTUBE\_DIFFERENTIATION(GO:0014902) |  | 81 | -0.23 | -1.06 | 0.406 | 0.698 | 1.000 | 8014 | tags=25%, list=25%, signal=33% |
| 1103 | NUCLEOBASE\_CONTAINING\_SMALL\_MOLECULE\_METABOLIC\_PROCESS(GO:0055086) |  | 425 | -0.18 | -1.06 | 0.373 | 0.698 | 1.000 | 6446 | tags=21%, list=20%, signal=26% |
| 1104 | SEX\_DIFFERENTIATION(GO:0007548) |  | 26 | -0.31 | -1.06 | 0.436 | 0.698 | 1.000 | 3858 | tags=15%, list=12%, signal=17% |
| 1105 | DENDRITE\_MORPHOGENESIS(GO:0048813) |  | 31 | -0.30 | -1.06 | 0.361 | 0.700 | 1.000 | 5592 | tags=19%, list=18%, signal=23% |
| 1106 | INFLAMMATORY\_RESPONSE(GO:0006954) |  | 174 | -0.23 | -1.06 | 0.284 | 0.700 | 1.000 | 2890 | tags=11%, list=9%, signal=12% |
| 1107 | STRUCTURAL\_CONSTITUENT\_OF\_RIBOSOME(GO:0003735) |  | 152 | -0.29 | -1.06 | 0.412 | 0.701 | 1.000 | 11078 | tags=44%, list=35%, signal=67% |
| 1108 | PURINE\_NUCLEOSIDE\_TRIPHOSPHATE\_METABOLIC\_PROCESS(GO:0009144) |  | 156 | -0.22 | -1.05 | 0.425 | 0.703 | 1.000 | 9801 | tags=38%, list=31%, signal=54% |
| 1109 | RESPONSE\_TO\_GROWTH\_FACTOR(GO:0070848) |  | 280 | -0.18 | -1.05 | 0.359 | 0.703 | 1.000 | 2706 | tags=7%, list=8%, signal=8% |
| 1110 | CELLULAR\_RESPONSE\_TO\_GROWTH\_FACTOR\_STIMULUS(GO:0071363) |  | 279 | -0.19 | -1.05 | 0.360 | 0.703 | 1.000 | 2706 | tags=7%, list=8%, signal=8% |
| 1111 | PEPTIDYL\_SERINE\_MODIFICATION(GO:0018209) |  | 119 | -0.23 | -1.05 | 0.360 | 0.703 | 1.000 | 2550 | tags=9%, list=8%, signal=10% |
| 1112 | GOLGI\_SUBCOMPARTMENT(GO:0098791) |  | 427 | -0.17 | -1.05 | 0.310 | 0.703 | 1.000 | 6694 | tags=19%, list=21%, signal=23% |
| 1113 | SPERM\_FLAGELLUM(GO:0036126) |  | 15 | -0.40 | -1.05 | 0.359 | 0.702 | 1.000 | 4810 | tags=33%, list=15%, signal=39% |
| 1114 | 9+2\_MOTILE\_CILIUM(GO:0097729) |  | 15 | -0.40 | -1.05 | 0.359 | 0.702 | 1.000 | 4810 | tags=33%, list=15%, signal=39% |
| 1115 | SEQUESTERING\_OF\_CALCIUM\_ION(GO:0051208) |  | 25 | -0.29 | -1.05 | 0.385 | 0.705 | 1.000 | 5936 | tags=32%, list=19%, signal=39% |
| 1116 | REGULATION\_OF\_SEQUESTERING\_OF\_CALCIUM\_ION(GO:0051282) |  | 25 | -0.29 | -1.05 | 0.385 | 0.704 | 1.000 | 5936 | tags=32%, list=19%, signal=39% |
| 1117 | NEGATIVE\_REGULATION\_OF\_SEQUESTERING\_OF\_CALCIUM\_ION(GO:0051283) |  | 25 | -0.29 | -1.05 | 0.385 | 0.704 | 1.000 | 5936 | tags=32%, list=19%, signal=39% |
| 1118 | GLYCOSYLATION(GO:0070085) |  | 198 | -0.22 | -1.05 | 0.277 | 0.703 | 1.000 | 6132 | tags=19%, list=19%, signal=24% |
| 1119 | RIBONUCLEOSIDE\_MONOPHOSPHATE\_METABOLIC\_PROCESS(GO:0009161) |  | 183 | -0.20 | -1.05 | 0.367 | 0.704 | 1.000 | 9801 | tags=36%, list=31%, signal=52% |
| 1120 | ORGANIC\_CATION\_TRANSMEMBRANE\_TRANSPORTER\_ACTIVITY(GO:0015101) |  | 17 | -0.34 | -1.05 | 0.302 | 0.703 | 1.000 | 5296 | tags=24%, list=17%, signal=28% |
| 1121 | REGULATION\_OF\_BIOLOGICAL\_QUALITY(GO:0065008) |  | 1770 | -0.18 | -1.05 | 0.352 | 0.703 | 1.000 | 5966 | tags=17%, list=19%, signal=20% |
| 1122 | REGULATION\_OF\_TRANSPORT(GO:0051049) |  | 452 | -0.21 | -1.05 | 0.361 | 0.702 | 1.000 | 5953 | tags=20%, list=19%, signal=24% |
| 1123 | REGULATION\_OF\_SECRETION(GO:0051046) |  | 137 | -0.26 | -1.05 | 0.348 | 0.702 | 1.000 | 9283 | tags=36%, list=29%, signal=50% |
| 1124 | DENDRITIC\_SPINE\_DEVELOPMENT(GO:0060996) |  | 17 | -0.37 | -1.05 | 0.401 | 0.702 | 1.000 | 8934 | tags=35%, list=28%, signal=49% |
| 1125 | PURINE\_RIBONUCLEOTIDE\_BIOSYNTHETIC\_PROCESS(GO:0009152) |  | 161 | -0.20 | -1.05 | 0.341 | 0.703 | 1.000 | 6379 | tags=22%, list=20%, signal=27% |
| 1126 | POSITIVE\_REGULATION\_OF\_DEVELOPMENTAL\_PROCESS(GO:0051094) |  | 245 | -0.19 | -1.05 | 0.282 | 0.702 | 1.000 | 6240 | tags=18%, list=20%, signal=22% |
| 1127 | ENDODEOXYRIBONUCLEASE\_ACTIVITY\_\_PRODUCING\_5'\_PHOSPHOMONOESTERS(GO:0016888) |  | 15 | -0.30 | -1.05 | 0.361 | 0.704 | 1.000 | 7439 | tags=33%, list=23%, signal=43% |
| 1128 | MOVEMENT\_OF\_CELL\_OR\_SUBCELLULAR\_COMPONENT(GO:0006928) |  | 1107 | -0.17 | -1.05 | 0.366 | 0.705 | 1.000 | 6968 | tags=19%, list=22%, signal=24% |
| 1129 | NUCLEOSIDE\_BIOSYNTHETIC\_PROCESS(GO:0009163) |  | 41 | -0.26 | -1.05 | 0.348 | 0.705 | 1.000 | 8837 | tags=39%, list=28%, signal=54% |
| 1130 | RIBONUCLEOSIDE\_BIOSYNTHETIC\_PROCESS(GO:0042455) |  | 41 | -0.26 | -1.05 | 0.348 | 0.704 | 1.000 | 8837 | tags=39%, list=28%, signal=54% |
| 1131 | SIGNAL\_TRANSDUCTION\_IN\_RESPONSE\_TO\_DNA\_DAMAGE(GO:0042770) |  | 15 | -0.33 | -1.05 | 0.380 | 0.704 | 1.000 | 1567 | tags=13%, list=5%, signal=14% |
| 1132 | POSITIVE\_REGULATION\_OF\_CELL\_DIFFERENTIATION(GO:0045597) |  | 167 | -0.22 | -1.05 | 0.324 | 0.704 | 1.000 | 6240 | tags=20%, list=20%, signal=24% |
| 1133 | DNA\_DIRECTED\_RNA\_POLYMERASE\_III\_COMPLEX(GO:0005666) |  | 19 | -0.38 | -1.05 | 0.373 | 0.705 | 1.000 | 8909 | tags=42%, list=28%, signal=58% |
| 1134 | INTRINSIC\_COMPONENT\_OF\_MITOCHONDRIAL\_INNER\_MEMBRANE(GO:0031304) |  | 47 | -0.27 | -1.05 | 0.425 | 0.706 | 1.000 | 8070 | tags=30%, list=25%, signal=40% |
| 1135 | ORGANELLE\_MEMBRANE(GO:0031090) |  | 1272 | -0.17 | -1.05 | 0.349 | 0.706 | 1.000 | 6694 | tags=19%, list=21%, signal=23% |
| 1136 | I\_BAND(GO:0031674) |  | 68 | -0.25 | -1.05 | 0.474 | 0.705 | 1.000 | 6852 | tags=24%, list=21%, signal=30% |
| 1137 | METAL\_ION\_TRANSPORT(GO:0030001) |  | 525 | -0.21 | -1.05 | 0.298 | 0.705 | 1.000 | 5936 | tags=19%, list=19%, signal=23% |
| 1138 | MYELOID\_LEUKOCYTE\_DIFFERENTIATION(GO:0002573) |  | 55 | -0.25 | -1.05 | 0.352 | 0.706 | 1.000 | 4847 | tags=15%, list=15%, signal=17% |
| 1139 | SIGNAL\_TRANSDUCTION(GO:0007165) |  | 3465 | -0.17 | -1.05 | 0.361 | 0.705 | 1.000 | 4449 | tags=12%, list=14%, signal=12% |
| 1140 | ACTIN\_FILAMENT\_DEPOLYMERIZATION(GO:0030042) |  | 49 | -0.24 | -1.05 | 0.374 | 0.705 | 1.000 | 7160 | tags=27%, list=22%, signal=34% |
| 1141 | 2\_IRON\_\_2\_SULFUR\_CLUSTER\_BINDING(GO:0051537) |  | 26 | -0.32 | -1.05 | 0.436 | 0.705 | 1.000 | 5550 | tags=27%, list=17%, signal=33% |
| 1142 | ADP\_METABOLIC\_PROCESS(GO:0046031) |  | 45 | -0.32 | -1.04 | 0.385 | 0.707 | 1.000 | 4213 | tags=18%, list=13%, signal=20% |
| 1143 | RIBOSE\_PHOSPHATE\_BIOSYNTHETIC\_PROCESS(GO:0046390) |  | 181 | -0.19 | -1.04 | 0.327 | 0.707 | 1.000 | 6379 | tags=22%, list=20%, signal=27% |
| 1144 | FOREBRAIN\_DEVELOPMENT(GO:0030900) |  | 130 | -0.23 | -1.04 | 0.358 | 0.707 | 1.000 | 9157 | tags=29%, list=29%, signal=41% |
| 1145 | PEPTIDYL\_PROLINE\_DIOXYGENASE\_ACTIVITY(GO:0031543) |  | 15 | -0.35 | -1.04 | 0.437 | 0.708 | 1.000 | 5375 | tags=33%, list=17%, signal=40% |
| 1146 | NUCLEAR\_LUMEN(GO:0031981) |  | 950 | -0.15 | -1.04 | 0.379 | 0.708 | 1.000 | 7098 | tags=18%, list=22%, signal=22% |
| 1147 | INTRINSIC\_APOPTOTIC\_SIGNALING\_PATHWAY(GO:0097193) |  | 72 | -0.22 | -1.04 | 0.298 | 0.708 | 1.000 | 5229 | tags=21%, list=16%, signal=25% |
| 1148 | TRANSLATION\_REGULATOR\_ACTIVITY(GO:0045182) |  | 30 | -0.32 | -1.04 | 0.392 | 0.710 | 1.000 | 7768 | tags=30%, list=24%, signal=40% |
| 1149 | RESPONSE\_TO\_UV(GO:0009411) |  | 22 | -0.30 | -1.04 | 0.415 | 0.710 | 1.000 | 8202 | tags=45%, list=26%, signal=61% |
| 1150 | NUCLEOTIDE\_METABOLIC\_PROCESS(GO:0009117) |  | 344 | -0.19 | -1.04 | 0.478 | 0.710 | 1.000 | 6789 | tags=22%, list=21%, signal=28% |
| 1151 | REGULATION\_OF\_CELLULAR\_CATABOLIC\_PROCESS(GO:0031329) |  | 195 | -0.16 | -1.04 | 0.327 | 0.712 | 1.000 | 4407 | tags=10%, list=14%, signal=11% |
| 1152 | CALCIUM\_CHANNEL\_REGULATOR\_ACTIVITY(GO:0005246) |  | 15 | -0.31 | -1.04 | 0.331 | 0.712 | 1.000 | 2099 | tags=13%, list=7%, signal=14% |
| 1153 | SODIUM\_ION\_TRANSMEMBRANE\_TRANSPORT(GO:0035725) |  | 115 | -0.24 | -1.04 | 0.372 | 0.712 | 1.000 | 3781 | tags=12%, list=12%, signal=14% |
| 1154 | SULFUR\_COMPOUND\_BIOSYNTHETIC\_PROCESS(GO:0044272) |  | 75 | -0.24 | -1.04 | 0.432 | 0.712 | 1.000 | 5851 | tags=27%, list=18%, signal=33% |
| 1155 | REGULATION\_OF\_SUPRAMOLECULAR\_FIBER\_ORGANIZATION(GO:1902903) |  | 177 | -0.18 | -1.04 | 0.316 | 0.712 | 1.000 | 6290 | tags=18%, list=20%, signal=22% |
| 1156 | REGULATION\_OF\_ALTERNATIVE\_MRNA\_SPLICING\_\_VIA\_SPLICEOSOME(GO:0000381) |  | 52 | -0.23 | -1.04 | 0.414 | 0.712 | 1.000 | 61 | tags=2%, list=0%, signal=2% |
| 1157 | REGULATION\_OF\_CATABOLIC\_PROCESS(GO:0009894) |  | 224 | -0.16 | -1.04 | 0.381 | 0.712 | 1.000 | 4407 | tags=9%, list=14%, signal=11% |
| 1158 | CHLORIDE\_ION\_HOMEOSTASIS(GO:0055064) |  | 19 | -0.38 | -1.04 | 0.437 | 0.712 | 1.000 | 9907 | tags=58%, list=31%, signal=84% |
| 1159 | EXTRINSIC\_COMPONENT\_OF\_CYTOPLASMIC\_SIDE\_OF\_PLASMA\_MEMBRANE(GO:0031234) |  | 87 | -0.24 | -1.04 | 0.350 | 0.712 | 1.000 | 409 | tags=3%, list=1%, signal=3% |
| 1160 | SENSORY\_ORGAN\_MORPHOGENESIS(GO:0090596) |  | 284 | -0.19 | -1.04 | 0.467 | 0.712 | 1.000 | 9211 | tags=29%, list=29%, signal=40% |
| 1161 | POSITIVE\_REGULATION\_OF\_LYMPHOCYTE\_ACTIVATION(GO:0051251) |  | 41 | -0.29 | -1.04 | 0.466 | 0.713 | 1.000 | 1287 | tags=12%, list=4%, signal=13% |
| 1162 | TRANSITION\_METAL\_ION\_TRANSPORT(GO:0000041) |  | 68 | -0.25 | -1.04 | 0.448 | 0.714 | 1.000 | 2578 | tags=10%, list=8%, signal=11% |
| 1163 | MONOSACCHARIDE\_METABOLIC\_PROCESS(GO:0005996) |  | 104 | -0.27 | -1.04 | 0.423 | 0.714 | 1.000 | 6328 | tags=25%, list=20%, signal=31% |
| 1164 | REGULATION\_OF\_CELL\_CYCLE\_G1\_S\_PHASE\_TRANSITION(GO:1902806) |  | 25 | -0.29 | -1.04 | 0.433 | 0.713 | 1.000 | 4370 | tags=16%, list=14%, signal=19% |
| 1165 | CELLULAR\_RESPONSE\_TO\_LIPOPOLYSACCHARIDE(GO:0071222) |  | 25 | -0.35 | -1.04 | 0.448 | 0.715 | 1.000 | 3530 | tags=20%, list=11%, signal=22% |
| 1166 | ENDOPLASMIC\_RETICULUM\_LUMEN(GO:0005788) |  | 43 | -0.32 | -1.04 | 0.465 | 0.715 | 1.000 | 2958 | tags=14%, list=9%, signal=15% |
| 1167 | PATTERN\_RECOGNITION\_RECEPTOR\_SIGNALING\_PATHWAY(GO:0002221) |  | 38 | -0.29 | -1.04 | 0.412 | 0.714 | 1.000 | 5854 | tags=21%, list=18%, signal=26% |
| 1168 | INNATE\_IMMUNE\_RESPONSE\_ACTIVATING\_SIGNAL\_TRANSDUCTION(GO:0002758) |  | 38 | -0.29 | -1.04 | 0.412 | 0.714 | 1.000 | 5854 | tags=21%, list=18%, signal=26% |
| 1169 | CELL\_CHEMOTAXIS(GO:0060326) |  | 108 | -0.23 | -1.04 | 0.418 | 0.714 | 1.000 | 3615 | tags=12%, list=11%, signal=14% |
| 1170 | PURINE\_NUCLEOSIDE\_DIPHOSPHATE\_METABOLIC\_PROCESS(GO:0009135) |  | 46 | -0.31 | -1.03 | 0.426 | 0.715 | 1.000 | 4213 | tags=17%, list=13%, signal=20% |
| 1171 | PURINE\_RIBONUCLEOSIDE\_DIPHOSPHATE\_METABOLIC\_PROCESS(GO:0009179) |  | 46 | -0.31 | -1.03 | 0.426 | 0.715 | 1.000 | 4213 | tags=17%, list=13%, signal=20% |
| 1172 | CENTRAL\_NERVOUS\_SYSTEM\_NEURON\_AXONOGENESIS(GO:0021955) |  | 26 | -0.30 | -1.03 | 0.446 | 0.715 | 1.000 | 2761 | tags=15%, list=9%, signal=17% |
| 1173 | PROTEIN\_KINASE\_INHIBITOR\_ACTIVITY(GO:0004860) |  | 27 | -0.28 | -1.03 | 0.393 | 0.714 | 1.000 | 7406 | tags=26%, list=23%, signal=34% |
| 1174 | KINASE\_INHIBITOR\_ACTIVITY(GO:0019210) |  | 27 | -0.28 | -1.03 | 0.393 | 0.714 | 1.000 | 7406 | tags=26%, list=23%, signal=34% |
| 1175 | RIBOSOME(GO:0005840) |  | 176 | -0.28 | -1.03 | 0.408 | 0.713 | 1.000 | 11078 | tags=44%, list=35%, signal=67% |
| 1176 | RRNA\_METHYLATION(GO:0031167) |  | 15 | -0.33 | -1.03 | 0.436 | 0.714 | 1.000 | 21450 | tags=100%, list=67%, signal=305% |
| 1177 | REGULATION\_OF\_CATION\_CHANNEL\_ACTIVITY(GO:2001257) |  | 58 | -0.30 | -1.03 | 0.363 | 0.715 | 1.000 | 5831 | tags=28%, list=18%, signal=34% |
| 1178 | RESPONSE\_TO\_EXTERNAL\_STIMULUS(GO:0009605) |  | 903 | -0.18 | -1.03 | 0.395 | 0.715 | 1.000 | 4402 | tags=12%, list=14%, signal=14% |
| 1179 | POSTSYNAPTIC\_DENSITY(GO:0014069) |  | 92 | -0.29 | -1.03 | 0.378 | 0.714 | 1.000 | 8983 | tags=39%, list=28%, signal=54% |
| 1180 | SPLICEOSOMAL\_TRI\_SNRNP\_COMPLEX(GO:0097526) |  | 24 | -0.38 | -1.03 | 0.460 | 0.715 | 1.000 | 6548 | tags=25%, list=21%, signal=31% |
| 1181 | NUCLEOSIDE\_PHOSPHATE\_METABOLIC\_PROCESS(GO:0006753) |  | 356 | -0.18 | -1.03 | 0.487 | 0.716 | 1.000 | 6789 | tags=22%, list=21%, signal=28% |
| 1182 | SECRETION(GO:0046903) |  | 270 | -0.22 | -1.03 | 0.335 | 0.716 | 1.000 | 6704 | tags=24%, list=21%, signal=30% |
| 1183 | CELLULAR\_NITROGEN\_COMPOUND\_BIOSYNTHETIC\_PROCESS(GO:0044271) |  | 3162 | -0.16 | -1.03 | 0.372 | 0.716 | 1.000 | 6532 | tags=17%, list=20%, signal=19% |
| 1184 | NUCLEOBASE\_CONTAINING\_COMPOUND\_BIOSYNTHETIC\_PROCESS(GO:0034654) |  | 2624 | -0.17 | -1.03 | 0.345 | 0.716 | 1.000 | 6532 | tags=17%, list=20%, signal=20% |
| 1185 | SKELETAL\_MUSCLE\_FIBER\_DEVELOPMENT(GO:0048741) |  | 65 | -0.24 | -1.03 | 0.404 | 0.715 | 1.000 | 6106 | tags=17%, list=19%, signal=21% |
| 1186 | MYOTUBE\_CELL\_DEVELOPMENT(GO:0014904) |  | 65 | -0.24 | -1.03 | 0.404 | 0.715 | 1.000 | 6106 | tags=17%, list=19%, signal=21% |
| 1187 | MUSCLE\_FIBER\_DEVELOPMENT(GO:0048747) |  | 65 | -0.24 | -1.03 | 0.404 | 0.714 | 1.000 | 6106 | tags=17%, list=19%, signal=21% |
| 1188 | NAD\_METABOLIC\_PROCESS(GO:0019674) |  | 30 | -0.31 | -1.03 | 0.485 | 0.713 | 1.000 | 6698 | tags=37%, list=21%, signal=46% |
| 1189 | CELLULAR\_RESPONSE\_TO\_ACID\_CHEMICAL(GO:0071229) |  | 36 | -0.30 | -1.03 | 0.314 | 0.715 | 1.000 | 4352 | tags=17%, list=14%, signal=19% |
| 1190 | RESPONSE\_TO\_INORGANIC\_SUBSTANCE(GO:0010035) |  | 109 | -0.23 | -1.03 | 0.460 | 0.714 | 1.000 | 6675 | tags=25%, list=21%, signal=31% |
| 1191 | REGULATION\_OF\_LYMPHOCYTE\_DIFFERENTIATION(GO:0045619) |  | 18 | -0.32 | -1.03 | 0.379 | 0.714 | 1.000 | 1287 | tags=11%, list=4%, signal=12% |
| 1192 | UROGENITAL\_SYSTEM\_DEVELOPMENT(GO:0001655) |  | 189 | -0.21 | -1.03 | 0.348 | 0.715 | 1.000 | 6788 | tags=19%, list=21%, signal=24% |
| 1193 | TELOMERE\_MAINTENANCE(GO:0000723) |  | 44 | -0.25 | -1.03 | 0.315 | 0.715 | 1.000 | 1069 | tags=5%, list=3%, signal=5% |
| 1194 | TELOMERE\_ORGANIZATION(GO:0032200) |  | 44 | -0.25 | -1.03 | 0.315 | 0.714 | 1.000 | 1069 | tags=5%, list=3%, signal=5% |
| 1195 | ORGANIC\_CYCLIC\_COMPOUND\_BIOSYNTHETIC\_PROCESS(GO:1901362) |  | 2756 | -0.17 | -1.03 | 0.347 | 0.714 | 1.000 | 6532 | tags=17%, list=20%, signal=20% |
| 1196 | DIENCEPHALON\_DEVELOPMENT(GO:0021536) |  | 78 | -0.25 | -1.03 | 0.450 | 0.716 | 1.000 | 9157 | tags=29%, list=29%, signal=41% |
| 1197 | DETERMINATION\_OF\_LEFT\_RIGHT\_ASYMMETRY\_IN\_LATERAL\_MESODERM(GO:0003140) |  | 21 | -0.33 | -1.03 | 0.370 | 0.716 | 1.000 | 8128 | tags=29%, list=25%, signal=38% |
| 1198 | KIDNEY\_DEVELOPMENT(GO:0001822) |  | 185 | -0.21 | -1.03 | 0.342 | 0.715 | 1.000 | 6788 | tags=19%, list=21%, signal=24% |
| 1199 | RENAL\_SYSTEM\_DEVELOPMENT(GO:0072001) |  | 185 | -0.21 | -1.03 | 0.342 | 0.715 | 1.000 | 6788 | tags=19%, list=21%, signal=24% |
| 1200 | NUCLEOSIDE\_MONOPHOSPHATE\_METABOLIC\_PROCESS(GO:0009123) |  | 187 | -0.19 | -1.03 | 0.390 | 0.714 | 1.000 | 9801 | tags=35%, list=31%, signal=51% |
| 1201 | HETEROCYCLE\_BIOSYNTHETIC\_PROCESS(GO:0018130) |  | 2685 | -0.17 | -1.03 | 0.345 | 0.714 | 1.000 | 6145 | tags=16%, list=19%, signal=18% |
| 1202 | EMBRYONIC\_SKELETAL\_SYSTEM\_MORPHOGENESIS(GO:0048704) |  | 176 | -0.19 | -1.03 | 0.397 | 0.714 | 1.000 | 6543 | tags=18%, list=21%, signal=22% |
| 1203 | SPERM\_PART(GO:0097223) |  | 22 | -0.35 | -1.03 | 0.442 | 0.714 | 1.000 | 4810 | tags=27%, list=15%, signal=32% |
| 1204 | REGULATION\_OF\_MITOTIC\_METAPHASE\_ANAPHASE\_TRANSITION(GO:0030071) |  | 26 | -0.34 | -1.03 | 0.431 | 0.714 | 1.000 | 2255 | tags=8%, list=7%, signal=8% |
| 1205 | REGULATION\_OF\_METAPHASE\_ANAPHASE\_TRANSITION\_OF\_CELL\_CYCLE(GO:1902099) |  | 26 | -0.34 | -1.03 | 0.431 | 0.713 | 1.000 | 2255 | tags=8%, list=7%, signal=8% |
| 1206 | TRNA\_AMINOACYLATION\_FOR\_PROTEIN\_TRANSLATION(GO:0006418) |  | 35 | -0.23 | -1.03 | 0.498 | 0.713 | 1.000 | 10351 | tags=51%, list=32%, signal=76% |
| 1207 | CELL\_COMMUNICATION(GO:0007154) |  | 3767 | -0.17 | -1.03 | 0.404 | 0.712 | 1.000 | 5434 | tags=15%, list=17%, signal=16% |
| 1208 | ENDOCRINE\_PANCREAS\_DEVELOPMENT(GO:0031018) |  | 69 | -0.24 | -1.03 | 0.326 | 0.713 | 1.000 | 1287 | tags=6%, list=4%, signal=6% |
| 1209 | REGULATION\_OF\_CELL\_CYCLE\_PHASE\_TRANSITION(GO:1901987) |  | 92 | -0.24 | -1.03 | 0.355 | 0.713 | 1.000 | 6511 | tags=20%, list=20%, signal=25% |
| 1210 | REGULATION\_OF\_G1\_S\_TRANSITION\_OF\_MITOTIC\_CELL\_CYCLE(GO:2000045) |  | 23 | -0.30 | -1.03 | 0.383 | 0.713 | 1.000 | 4370 | tags=17%, list=14%, signal=20% |
| 1211 | INTEGRAL\_COMPONENT\_OF\_MITOCHONDRIAL\_INNER\_MEMBRANE(GO:0031305) |  | 46 | -0.27 | -1.03 | 0.464 | 0.713 | 1.000 | 7933 | tags=28%, list=25%, signal=38% |
| 1212 | POSITIVE\_REGULATION\_OF\_T\_CELL\_PROLIFERATION(GO:0042102) |  | 17 | -0.35 | -1.03 | 0.467 | 0.712 | 1.000 | 1287 | tags=18%, list=4%, signal=18% |
| 1213 | POSITIVE\_REGULATION\_OF\_DNA\_METABOLIC\_PROCESS(GO:0051054) |  | 47 | -0.22 | -1.03 | 0.439 | 0.712 | 1.000 | 4044 | tags=11%, list=13%, signal=12% |
| 1214 | CALCIUM\_ION\_TRANSPORT(GO:0006816) |  | 172 | -0.23 | -1.03 | 0.316 | 0.712 | 1.000 | 5936 | tags=20%, list=19%, signal=25% |
| 1215 | DIVALENT\_METAL\_ION\_TRANSPORT(GO:0070838) |  | 203 | -0.23 | -1.03 | 0.410 | 0.712 | 1.000 | 5936 | tags=20%, list=19%, signal=25% |
| 1216 | MELANOSOME\_ORGANIZATION(GO:0032438) |  | 15 | -0.39 | -1.02 | 0.425 | 0.712 | 1.000 | 9412 | tags=60%, list=30%, signal=85% |
| 1217 | ANATOMICAL\_STRUCTURE\_FORMATION\_INVOLVED\_IN\_MORPHOGENESIS(GO:0048646) |  | 816 | -0.16 | -1.02 | 0.401 | 0.712 | 1.000 | 5806 | tags=14%, list=18%, signal=17% |
| 1218 | CYTOSOLIC\_LARGE\_RIBOSOMAL\_SUBUNIT(GO:0022625) |  | 43 | -0.35 | -1.02 | 0.440 | 0.712 | 1.000 | 10371 | tags=44%, list=33%, signal=65% |
| 1219 | CELLULAR\_DIVALENT\_INORGANIC\_CATION\_HOMEOSTASIS(GO:0072503) |  | 209 | -0.21 | -1.02 | 0.365 | 0.714 | 1.000 | 5936 | tags=20%, list=19%, signal=24% |
| 1220 | PROTEIN\_LOCALIZATION\_TO\_MITOCHONDRION(GO:0070585) |  | 43 | -0.28 | -1.02 | 0.453 | 0.714 | 1.000 | 6343 | tags=23%, list=20%, signal=29% |
| 1221 | ESTABLISHMENT\_OF\_PROTEIN\_LOCALIZATION\_TO\_MITOCHONDRION(GO:0072655) |  | 43 | -0.28 | -1.02 | 0.453 | 0.713 | 1.000 | 6343 | tags=23%, list=20%, signal=29% |
| 1222 | IMPORT\_ACROSS\_PLASMA\_MEMBRANE(GO:0098739) |  | 86 | -0.27 | -1.02 | 0.406 | 0.713 | 1.000 | 3475 | tags=15%, list=11%, signal=17% |
| 1223 | PYRIDINE\_CONTAINING\_COMPOUND\_BIOSYNTHETIC\_PROCESS(GO:0072525) |  | 61 | -0.30 | -1.02 | 0.439 | 0.713 | 1.000 | 6925 | tags=33%, list=22%, signal=42% |
| 1224 | PONS\_DEVELOPMENT(GO:0021548) |  | 18 | -0.35 | -1.02 | 0.473 | 0.713 | 1.000 | 10930 | tags=61%, list=34%, signal=93% |
| 1225 | POSITIVE\_REGULATION\_OF\_AUTOPHAGY(GO:0010508) |  | 20 | -0.31 | -1.02 | 0.442 | 0.713 | 1.000 | 12142 | tags=55%, list=38%, signal=89% |
| 1226 | MONOSACCHARIDE\_BIOSYNTHETIC\_PROCESS(GO:0046364) |  | 27 | -0.28 | -1.02 | 0.500 | 0.713 | 1.000 | 2128 | tags=15%, list=7%, signal=16% |
| 1227 | NEUTROPHIL\_CHEMOTAXIS(GO:0030593) |  | 57 | -0.26 | -1.02 | 0.463 | 0.712 | 1.000 | 3347 | tags=12%, list=10%, signal=14% |
| 1228 | NUCLEOSIDE\_PHOSPHATE\_BIOSYNTHETIC\_PROCESS(GO:1901293) |  | 214 | -0.19 | -1.02 | 0.321 | 0.713 | 1.000 | 6379 | tags=22%, list=20%, signal=27% |
| 1229 | NEGATIVE\_REGULATION\_OF\_TRANSCRIPTION\_BY\_RNA\_POLYMERASE\_II(GO:0000122) |  | 197 | -0.18 | -1.02 | 0.416 | 0.713 | 1.000 | 6348 | tags=16%, list=20%, signal=20% |
| 1230 | PURINE\_NUCLEOTIDE\_METABOLIC\_PROCESS(GO:0006163) |  | 262 | -0.19 | -1.02 | 0.487 | 0.713 | 1.000 | 6446 | tags=21%, list=20%, signal=26% |
| 1231 | SOMITE\_DEVELOPMENT(GO:0061053) |  | 138 | -0.19 | -1.02 | 0.422 | 0.714 | 1.000 | 4502 | tags=12%, list=14%, signal=13% |
| 1232 | ACTIVATION\_OF\_INNATE\_IMMUNE\_RESPONSE(GO:0002218) |  | 39 | -0.29 | -1.02 | 0.466 | 0.714 | 1.000 | 5854 | tags=21%, list=18%, signal=25% |
| 1233 | BODY\_MORPHOGENESIS(GO:0010171) |  | 17 | -0.30 | -1.02 | 0.409 | 0.714 | 1.000 | 7720 | tags=24%, list=24%, signal=31% |
| 1234 | ORGANOPHOSPHATE\_BIOSYNTHETIC\_PROCESS(GO:0090407) |  | 350 | -0.19 | -1.02 | 0.357 | 0.714 | 1.000 | 6789 | tags=21%, list=21%, signal=27% |
| 1235 | PIGMENT\_GRANULE\_ORGANIZATION(GO:0048753) |  | 16 | -0.37 | -1.02 | 0.355 | 0.714 | 1.000 | 9412 | tags=56%, list=30%, signal=80% |
| 1236 | CELL\_MOTILITY(GO:0048870) |  | 746 | -0.17 | -1.02 | 0.394 | 0.714 | 1.000 | 4471 | tags=12%, list=14%, signal=13% |
| 1237 | LOCALIZATION\_OF\_CELL(GO:0051674) |  | 746 | -0.17 | -1.02 | 0.394 | 0.713 | 1.000 | 4471 | tags=12%, list=14%, signal=13% |
| 1238 | REGULATION\_OF\_MORPHOGENESIS\_OF\_AN\_EPITHELIUM(GO:1905330) |  | 39 | -0.26 | -1.02 | 0.360 | 0.713 | 1.000 | 6094 | tags=18%, list=19%, signal=22% |
| 1239 | Z\_DISC(GO:0030018) |  | 63 | -0.24 | -1.02 | 0.456 | 0.713 | 1.000 | 6852 | tags=24%, list=21%, signal=30% |
| 1240 | CELL\_CORTEX\_REGION(GO:0099738) |  | 18 | -0.38 | -1.02 | 0.407 | 0.714 | 1.000 | 1670 | tags=11%, list=5%, signal=12% |
| 1241 | PROTEIN\_SERINE\_THREONINE\_KINASE\_ACTIVITY(GO:0004674) |  | 455 | -0.17 | -1.02 | 0.428 | 0.714 | 1.000 | 6265 | tags=16%, list=20%, signal=20% |
| 1242 | PANCREAS\_DEVELOPMENT(GO:0031016) |  | 154 | -0.19 | -1.02 | 0.388 | 0.713 | 1.000 | 6514 | tags=18%, list=20%, signal=23% |
| 1243 | AMMONIUM\_TRANSMEMBRANE\_TRANSPORTER\_ACTIVITY(GO:0008519) |  | 27 | -0.32 | -1.02 | 0.364 | 0.713 | 1.000 | 3807 | tags=19%, list=12%, signal=21% |
| 1244 | ATP\_METABOLIC\_PROCESS(GO:0046034) |  | 138 | -0.22 | -1.02 | 0.408 | 0.714 | 1.000 | 9801 | tags=38%, list=31%, signal=54% |
| 1245 | HORMONE\_TRANSPORT(GO:0009914) |  | 40 | -0.33 | -1.02 | 0.338 | 0.714 | 1.000 | 4587 | tags=18%, list=14%, signal=20% |
| 1246 | AROMATIC\_COMPOUND\_BIOSYNTHETIC\_PROCESS(GO:0019438) |  | 2680 | -0.17 | -1.02 | 0.401 | 0.714 | 1.000 | 6532 | tags=17%, list=20%, signal=20% |
| 1247 | MRNA\_PROCESSING(GO:0006397) |  | 321 | -0.14 | -1.02 | 0.385 | 0.714 | 1.000 | 624 | tags=2%, list=2%, signal=2% |
| 1248 | PYRIDINE\_NUCLEOTIDE\_BIOSYNTHETIC\_PROCESS(GO:0019363) |  | 57 | -0.29 | -1.02 | 0.424 | 0.714 | 1.000 | 6789 | tags=32%, list=21%, signal=40% |
| 1249 | NICOTINAMIDE\_NUCLEOTIDE\_BIOSYNTHETIC\_PROCESS(GO:0019359) |  | 57 | -0.29 | -1.02 | 0.424 | 0.714 | 1.000 | 6789 | tags=32%, list=21%, signal=40% |
| 1250 | REGULATION\_OF\_GLUTAMATE\_RECEPTOR\_SIGNALING\_PATHWAY(GO:1900449) |  | 19 | -0.36 | -1.01 | 0.442 | 0.719 | 1.000 | 11828 | tags=63%, list=37%, signal=100% |
| 1251 | NEUTRAL\_LIPID\_METABOLIC\_PROCESS(GO:0006638) |  | 52 | -0.27 | -1.01 | 0.372 | 0.720 | 1.000 | 4125 | tags=13%, list=13%, signal=15% |
| 1252 | ACYLGLYCEROL\_METABOLIC\_PROCESS(GO:0006639) |  | 52 | -0.27 | -1.01 | 0.372 | 0.720 | 1.000 | 4125 | tags=13%, list=13%, signal=15% |
| 1253 | REGULATION\_OF\_TRANSMEMBRANE\_TRANSPORT(GO:0034762) |  | 226 | -0.23 | -1.01 | 0.454 | 0.722 | 1.000 | 5936 | tags=23%, list=19%, signal=28% |
| 1254 | CELL\_FATE\_COMMITMENT\_INVOLVED\_IN\_FORMATION\_OF\_PRIMARY\_GERM\_LAYER(GO:0060795) |  | 30 | -0.29 | -1.01 | 0.404 | 0.724 | 1.000 | 1567 | tags=10%, list=5%, signal=11% |
| 1255 | ENDOSOME\_MEMBRANE(GO:0010008) |  | 180 | -0.18 | -1.01 | 0.397 | 0.724 | 1.000 | 2453 | tags=7%, list=8%, signal=8% |
| 1256 | GLUTATHIONE\_PEROXIDASE\_ACTIVITY(GO:0004602) |  | 15 | -0.40 | -1.01 | 0.498 | 0.728 | 1.000 | 4356 | tags=27%, list=14%, signal=31% |
| 1257 | GOLGI\_ASSOCIATED\_VESICLE(GO:0005798) |  | 85 | -0.20 | -1.01 | 0.490 | 0.728 | 1.000 | 3643 | tags=12%, list=11%, signal=13% |
| 1258 | REGULATION\_OF\_WOUND\_HEALING(GO:0061041) |  | 27 | -0.31 | -1.01 | 0.305 | 0.728 | 1.000 | 5255 | tags=22%, list=16%, signal=27% |
| 1259 | REGULATION\_OF\_MITOTIC\_CELL\_CYCLE\_PHASE\_TRANSITION(GO:1901990) |  | 85 | -0.24 | -1.01 | 0.388 | 0.728 | 1.000 | 6511 | tags=19%, list=20%, signal=24% |
| 1260 | TRANSCRIPTION\_COACTIVATOR\_ACTIVITY(GO:0003713) |  | 100 | -0.20 | -1.01 | 0.453 | 0.729 | 1.000 | 3764 | tags=9%, list=12%, signal=10% |
| 1261 | SMALL\_NUCLEOLAR\_RIBONUCLEOPROTEIN\_COMPLEX(GO:0005732) |  | 18 | -0.33 | -1.01 | 0.401 | 0.729 | 1.000 | 12399 | tags=61%, list=39%, signal=100% |
| 1262 | SIGNALING(GO:0023052) |  | 3742 | -0.17 | -1.01 | 0.482 | 0.729 | 1.000 | 4587 | tags=12%, list=14%, signal=13% |
| 1263 | REGULATION\_OF\_GASTRULATION(GO:0010470) |  | 39 | -0.28 | -1.01 | 0.406 | 0.730 | 1.000 | 1834 | tags=8%, list=6%, signal=8% |
| 1264 | DORSAL\_AORTA\_DEVELOPMENT(GO:0035907) |  | 15 | -0.28 | -1.01 | 0.356 | 0.731 | 1.000 | 4277 | tags=20%, list=13%, signal=23% |
| 1265 | I\_KAPPAB\_KINASE\_NF\_KAPPAB\_SIGNALING(GO:0007249) |  | 65 | -0.23 | -1.01 | 0.449 | 0.732 | 1.000 | 4405 | tags=15%, list=14%, signal=18% |
| 1266 | LATERAL\_MESODERM\_DEVELOPMENT(GO:0048368) |  | 24 | -0.31 | -1.00 | 0.421 | 0.733 | 1.000 | 6451 | tags=25%, list=20%, signal=31% |
| 1267 | PROTEIN\_COMPLEX\_OLIGOMERIZATION(GO:0051259) |  | 107 | -0.20 | -1.00 | 0.408 | 0.733 | 1.000 | 7269 | tags=27%, list=23%, signal=35% |
| 1268 | PRECATALYTIC\_SPLICEOSOME(GO:0071011) |  | 22 | -0.30 | -1.00 | 0.428 | 0.733 | 1.000 | 508 | tags=5%, list=2%, signal=5% |
| 1269 | BRANCHING\_MORPHOGENESIS\_OF\_AN\_EPITHELIAL\_TUBE(GO:0048754) |  | 20 | -0.34 | -1.00 | 0.420 | 0.733 | 1.000 | 2777 | tags=20%, list=9%, signal=22% |
| 1270 | SKELETAL\_SYSTEM\_DEVELOPMENT(GO:0001501) |  | 352 | -0.18 | -1.00 | 0.427 | 0.733 | 1.000 | 9630 | tags=29%, list=30%, signal=41% |
| 1271 | REGULATION\_OF\_ACTIN\_FILAMENT\_BUNDLE\_ASSEMBLY(GO:0032231) |  | 19 | -0.39 | -1.00 | 0.551 | 0.732 | 1.000 | 8089 | tags=47%, list=25%, signal=63% |
| 1272 | PROTEIN\_MODIFICATION\_BY\_SMALL\_PROTEIN\_REMOVAL(GO:0070646) |  | 101 | -0.19 | -1.00 | 0.419 | 0.732 | 1.000 | 9526 | tags=32%, list=30%, signal=45% |
| 1273 | POSITIVE\_REGULATION\_OF\_PROTEIN\_UBIQUITINATION(GO:0031398) |  | 25 | -0.25 | -1.00 | 0.339 | 0.732 | 1.000 | 3560 | tags=12%, list=11%, signal=13% |
| 1274 | TRANSLATION\_INITIATION\_FACTOR\_BINDING(GO:0031369) |  | 15 | -0.32 | -1.00 | 0.478 | 0.732 | 1.000 | 21608 | tags=100%, list=68%, signal=310% |
| 1275 | PROTEIN\_METHYLATION(GO:0006479) |  | 80 | -0.19 | -1.00 | 0.485 | 0.731 | 1.000 | 1027 | tags=4%, list=3%, signal=4% |
| 1276 | PROTEIN\_ALKYLATION(GO:0008213) |  | 80 | -0.19 | -1.00 | 0.485 | 0.731 | 1.000 | 1027 | tags=4%, list=3%, signal=4% |
| 1277 | NEUROPEPTIDE\_SIGNALING\_PATHWAY(GO:0007218) |  | 70 | -0.31 | -1.00 | 0.464 | 0.730 | 1.000 | 5763 | tags=26%, list=18%, signal=31% |
| 1278 | REGULATION\_OF\_SIGNAL\_TRANSDUCTION(GO:0009966) |  | 1106 | -0.16 | -1.00 | 0.437 | 0.730 | 1.000 | 5377 | tags=14%, list=17%, signal=16% |
| 1279 | HORMONE\_BIOSYNTHETIC\_PROCESS(GO:0042446) |  | 26 | -0.31 | -1.00 | 0.446 | 0.730 | 1.000 | 3151 | tags=15%, list=10%, signal=17% |
| 1280 | OXYGEN\_TRANSPORT(GO:0015671) |  | 16 | -0.35 | -1.00 | 0.459 | 0.731 | 1.000 | 1987 | tags=19%, list=6%, signal=20% |
| 1281 | VASCULOGENESIS(GO:0001570) |  | 53 | -0.22 | -1.00 | 0.416 | 0.731 | 1.000 | 5844 | tags=23%, list=18%, signal=28% |
| 1282 | NEGATIVE\_REGULATION\_OF\_NUCLEOBASE\_CONTAINING\_COMPOUND\_METABOLIC\_PROCESS(GO:0045934) |  | 407 | -0.17 | -1.00 | 0.333 | 0.730 | 1.000 | 6367 | tags=15%, list=20%, signal=19% |
| 1283 | L\_ASCORBIC\_ACID\_BINDING(GO:0031418) |  | 19 | -0.30 | -1.00 | 0.530 | 0.730 | 1.000 | 6639 | tags=32%, list=21%, signal=40% |
| 1284 | DIVALENT\_INORGANIC\_CATION\_TRANSPORT(GO:0072511) |  | 204 | -0.22 | -1.00 | 0.410 | 0.731 | 1.000 | 5936 | tags=20%, list=19%, signal=25% |
| 1285 | CATION\_HOMEOSTASIS(GO:0055080) |  | 348 | -0.19 | -1.00 | 0.448 | 0.732 | 1.000 | 5936 | tags=19%, list=19%, signal=23% |
| 1286 | INNER\_EAR\_MORPHOGENESIS(GO:0042472) |  | 86 | -0.20 | -1.00 | 0.380 | 0.732 | 1.000 | 8983 | tags=27%, list=28%, signal=37% |
| 1287 | STEROL\_METABOLIC\_PROCESS(GO:0016125) |  | 71 | -0.27 | -1.00 | 0.435 | 0.732 | 1.000 | 3229 | tags=15%, list=10%, signal=17% |
| 1288 | MORPHOGENESIS\_OF\_A\_POLARIZED\_EPITHELIUM(GO:0001738) |  | 27 | -0.32 | -1.00 | 0.484 | 0.731 | 1.000 | 13112 | tags=59%, list=41%, signal=101% |
| 1289 | STEROID\_BIOSYNTHETIC\_PROCESS(GO:0006694) |  | 62 | -0.30 | -1.00 | 0.380 | 0.731 | 1.000 | 3622 | tags=19%, list=11%, signal=22% |
| 1290 | RIBOSOMAL\_SUBUNIT(GO:0044391) |  | 138 | -0.26 | -1.00 | 0.432 | 0.731 | 1.000 | 11045 | tags=42%, list=35%, signal=64% |
| 1291 | TRANSMEMBRANE\_RECEPTOR\_PROTEIN\_SERINE\_THREONINE\_KINASE\_SIGNALING\_PATHWAY(GO:0007178) |  | 197 | -0.19 | -1.00 | 0.371 | 0.731 | 1.000 | 2028 | tags=6%, list=6%, signal=6% |
| 1292 | NUCLEOBASE\_CONTAINING\_SMALL\_MOLECULE\_BIOSYNTHETIC\_PROCESS(GO:0034404) |  | 122 | -0.21 | -1.00 | 0.439 | 0.730 | 1.000 | 8837 | tags=33%, list=28%, signal=45% |
| 1293 | CELLULAR\_CALCIUM\_ION\_HOMEOSTASIS(GO:0006874) |  | 194 | -0.21 | -1.00 | 0.430 | 0.730 | 1.000 | 5936 | tags=21%, list=19%, signal=25% |
| 1294 | COLLAGEN\_METABOLIC\_PROCESS(GO:0032963) |  | 28 | -0.25 | -1.00 | 0.449 | 0.730 | 1.000 | 5265 | tags=25%, list=17%, signal=30% |
| 1295 | MATURATION\_OF\_LSU\_RRNA(GO:0000470) |  | 29 | -0.29 | -1.00 | 0.438 | 0.733 | 1.000 | 11997 | tags=59%, list=38%, signal=94% |
| 1296 | ORGANELLE\_ENVELOPE(GO:0031967) |  | 563 | -0.18 | -1.00 | 0.346 | 0.733 | 1.000 | 6488 | tags=19%, list=20%, signal=23% |
| 1297 | ENVELOPE(GO:0031975) |  | 563 | -0.18 | -1.00 | 0.346 | 0.733 | 1.000 | 6488 | tags=19%, list=20%, signal=23% |
| 1298 | U4\_U6\_X\_U5\_TRI\_SNRNP\_COMPLEX(GO:0046540) |  | 19 | -0.37 | -1.00 | 0.338 | 0.734 | 1.000 | 1996 | tags=11%, list=6%, signal=11% |
| 1299 | SYNAPTIC\_VESICLE\_MEMBRANE(GO:0030672) |  | 48 | -0.30 | -1.00 | 0.529 | 0.734 | 1.000 | 6894 | tags=31%, list=22%, signal=40% |
| 1300 | EXOCYTIC\_VESICLE\_MEMBRANE(GO:0099501) |  | 48 | -0.30 | -1.00 | 0.529 | 0.733 | 1.000 | 6894 | tags=31%, list=22%, signal=40% |
| 1301 | RNA\_SPLICING(GO:0008380) |  | 285 | -0.14 | -1.00 | 0.532 | 0.733 | 1.000 | 11700 | tags=36%, list=37%, signal=57% |
| 1302 | REGULATION\_OF\_JNK\_CASCADE(GO:0046328) |  | 30 | -0.28 | -1.00 | 0.444 | 0.734 | 1.000 | 2860 | tags=13%, list=9%, signal=15% |
| 1303 | SULFOTRANSFERASE\_ACTIVITY(GO:0008146) |  | 70 | -0.24 | -1.00 | 0.465 | 0.734 | 1.000 | 5477 | tags=19%, list=17%, signal=22% |
| 1304 | ENDOSOME\_TO\_LYSOSOME\_TRANSPORT(GO:0008333) |  | 30 | -0.29 | -0.99 | 0.463 | 0.736 | 1.000 | 8898 | tags=40%, list=28%, signal=55% |
| 1305 | FEEDING\_BEHAVIOR(GO:0007631) |  | 22 | -0.39 | -0.99 | 0.510 | 0.736 | 1.000 | 7167 | tags=41%, list=22%, signal=53% |
| 1306 | ARP2\_3\_COMPLEX\_MEDIATED\_ACTIN\_NUCLEATION(GO:0034314) |  | 34 | -0.23 | -0.99 | 0.438 | 0.736 | 1.000 | 5116 | tags=12%, list=16%, signal=14% |
| 1307 | TRANSCRIPTION\_\_DNA\_TEMPLATED(GO:0006351) |  | 2306 | -0.17 | -0.99 | 0.425 | 0.736 | 1.000 | 6145 | tags=16%, list=19%, signal=18% |
| 1308 | NUCLEIC\_ACID\_TEMPLATED\_TRANSCRIPTION(GO:0097659) |  | 2307 | -0.17 | -0.99 | 0.425 | 0.736 | 1.000 | 6145 | tags=16%, list=19%, signal=18% |
| 1309 | RNA\_BIOSYNTHETIC\_PROCESS(GO:0032774) |  | 2312 | -0.17 | -0.99 | 0.423 | 0.736 | 1.000 | 6145 | tags=16%, list=19%, signal=18% |
| 1310 | PEPTIDYL\_SERINE\_PHOSPHORYLATION(GO:0018105) |  | 114 | -0.22 | -0.99 | 0.466 | 0.736 | 1.000 | 2992 | tags=10%, list=9%, signal=11% |
| 1311 | APPENDAGE\_MORPHOGENESIS(GO:0035107) |  | 59 | -0.21 | -0.99 | 0.484 | 0.739 | 1.000 | 8153 | tags=31%, list=26%, signal=41% |
| 1312 | GLYCOSYL\_COMPOUND\_BIOSYNTHETIC\_PROCESS(GO:1901659) |  | 44 | -0.25 | -0.99 | 0.429 | 0.739 | 1.000 | 7271 | tags=32%, list=23%, signal=41% |
| 1313 | METHYLTRANSFERASE\_ACTIVITY(GO:0008168) |  | 193 | -0.20 | -0.99 | 0.353 | 0.740 | 1.000 | 4273 | tags=10%, list=13%, signal=11% |
| 1314 | CELL\_PART\_MORPHOGENESIS(GO:0032990) |  | 470 | -0.19 | -0.99 | 0.505 | 0.740 | 1.000 | 8289 | tags=24%, list=26%, signal=32% |
| 1315 | POSITIVE\_REGULATION\_OF\_MONONUCLEAR\_CELL\_PROLIFERATION(GO:0032946) |  | 19 | -0.34 | -0.99 | 0.438 | 0.740 | 1.000 | 1287 | tags=16%, list=4%, signal=16% |
| 1316 | POSITIVE\_REGULATION\_OF\_LYMPHOCYTE\_PROLIFERATION(GO:0050671) |  | 19 | -0.34 | -0.99 | 0.438 | 0.740 | 1.000 | 1287 | tags=16%, list=4%, signal=16% |
| 1317 | POSITIVE\_REGULATION\_OF\_LEUKOCYTE\_PROLIFERATION(GO:0070665) |  | 19 | -0.34 | -0.99 | 0.438 | 0.739 | 1.000 | 1287 | tags=16%, list=4%, signal=16% |
| 1318 | IMMUNE\_RESPONSE\_ACTIVATING\_SIGNAL\_TRANSDUCTION(GO:0002757) |  | 88 | -0.25 | -0.99 | 0.484 | 0.740 | 1.000 | 5470 | tags=20%, list=17%, signal=25% |
| 1319 | LIGASE\_ACTIVITY\_\_FORMING\_CARBON\_NITROGEN\_BONDS(GO:0016879) |  | 44 | -0.24 | -0.99 | 0.526 | 0.739 | 1.000 | 9373 | tags=43%, list=29%, signal=61% |
| 1320 | INO80\_TYPE\_COMPLEX(GO:0097346) |  | 17 | -0.28 | -0.99 | 0.468 | 0.739 | 1.000 | 12099 | tags=41%, list=38%, signal=66% |
| 1321 | CELL\_PROJECTION\_PART(GO:0044463) |  | 474 | -0.20 | -0.99 | 0.476 | 0.739 | 1.000 | 5901 | tags=20%, list=19%, signal=24% |
| 1322 | PLASMA\_MEMBRANE\_BOUNDED\_CELL\_PROJECTION\_PART(GO:0120038) |  | 474 | -0.20 | -0.99 | 0.476 | 0.739 | 1.000 | 5901 | tags=20%, list=19%, signal=24% |
| 1323 | PLASMA\_MEMBRANE\_RECEPTOR\_COMPLEX(GO:0098802) |  | 110 | -0.24 | -0.99 | 0.463 | 0.738 | 1.000 | 8302 | tags=29%, list=26%, signal=39% |
| 1324 | OXIDATIVE\_PHOSPHORYLATION(GO:0006119) |  | 71 | -0.23 | -0.99 | 0.424 | 0.739 | 1.000 | 11201 | tags=49%, list=35%, signal=76% |
| 1325 | CILIARY\_MEMBRANE(GO:0060170) |  | 20 | -0.31 | -0.99 | 0.506 | 0.739 | 1.000 | 4369 | tags=25%, list=14%, signal=29% |
| 1326 | ENDODERM\_FORMATION(GO:0001706) |  | 40 | -0.26 | -0.99 | 0.482 | 0.740 | 1.000 | 1743 | tags=10%, list=5%, signal=11% |
| 1327 | INTEGRAL\_COMPONENT\_OF\_MITOCHONDRIAL\_MEMBRANE(GO:0032592) |  | 61 | -0.24 | -0.99 | 0.480 | 0.740 | 1.000 | 7933 | tags=28%, list=25%, signal=37% |
| 1328 | GAS\_TRANSPORT(GO:0015669) |  | 20 | -0.34 | -0.99 | 0.427 | 0.739 | 1.000 | 4287 | tags=25%, list=13%, signal=29% |
| 1329 | RIBONUCLEOSIDE\_DIPHOSPHATE\_METABOLIC\_PROCESS(GO:0009185) |  | 49 | -0.29 | -0.99 | 0.480 | 0.740 | 1.000 | 4213 | tags=16%, list=13%, signal=19% |
| 1330 | ADENYLATE\_CYCLASE\_ACTIVATING\_ADRENERGIC\_RECEPTOR\_SIGNALING\_PATHWAY(GO:0071880) |  | 24 | -0.33 | -0.99 | 0.552 | 0.740 | 1.000 | 7226 | tags=38%, list=23%, signal=48% |
| 1331 | REGULATION\_OF\_DNA\_BINDING\_TRANSCRIPTION\_FACTOR\_ACTIVITY(GO:0051090) |  | 65 | -0.20 | -0.99 | 0.467 | 0.741 | 1.000 | 10018 | tags=34%, list=31%, signal=49% |
| 1332 | NERVE\_DEVELOPMENT(GO:0021675) |  | 74 | -0.21 | -0.99 | 0.465 | 0.743 | 1.000 | 3736 | tags=11%, list=12%, signal=12% |
| 1333 | CYTOSKELETON\_DEPENDENT\_INTRACELLULAR\_TRANSPORT(GO:0030705) |  | 105 | -0.22 | -0.99 | 0.511 | 0.742 | 1.000 | 3518 | tags=11%, list=11%, signal=13% |
| 1334 | PROTEIN\_BINDING\_\_BRIDGING(GO:0030674) |  | 46 | -0.26 | -0.99 | 0.449 | 0.742 | 1.000 | 6584 | tags=24%, list=21%, signal=30% |
| 1335 | PURINE\_RIBONUCLEOTIDE\_METABOLIC\_PROCESS(GO:0009150) |  | 251 | -0.18 | -0.99 | 0.573 | 0.742 | 1.000 | 6789 | tags=22%, list=21%, signal=27% |
| 1336 | MICROTUBULE\_BASED\_PROCESS(GO:0007017) |  | 501 | -0.17 | -0.99 | 0.459 | 0.742 | 1.000 | 3596 | tags=9%, list=11%, signal=10% |
| 1337 | REGULATION\_OF\_DEVELOPMENTAL\_PROCESS(GO:0050793) |  | 852 | -0.17 | -0.99 | 0.476 | 0.742 | 1.000 | 6299 | tags=17%, list=20%, signal=21% |
| 1338 | PLASMA\_MEMBRANE\_BOUNDED\_CELL\_PROJECTION\_ASSEMBLY(GO:0120031) |  | 331 | -0.17 | -0.99 | 0.515 | 0.741 | 1.000 | 5996 | tags=15%, list=19%, signal=18% |
| 1339 | ZINC\_ION\_TRANSMEMBRANE\_TRANSPORTER\_ACTIVITY(GO:0005385) |  | 22 | -0.31 | -0.98 | 0.544 | 0.741 | 1.000 | 5182 | tags=18%, list=16%, signal=22% |
| 1340 | DIVALENT\_INORGANIC\_CATION\_TRANSMEMBRANE\_TRANSPORTER\_ACTIVITY(GO:0072509) |  | 22 | -0.31 | -0.98 | 0.544 | 0.740 | 1.000 | 5182 | tags=18%, list=16%, signal=22% |
| 1341 | PROTON\_TRANSPORTING\_TWO\_SECTOR\_ATPASE\_COMPLEX\_\_PROTON\_TRANSPORTING\_DOMAIN(GO:0033177) |  | 30 | -0.29 | -0.98 | 0.431 | 0.742 | 1.000 | 6374 | tags=23%, list=20%, signal=29% |
| 1342 | TRANSFERASE\_ACTIVITY\_\_TRANSFERRING\_ONE\_CARBON\_GROUPS(GO:0016741) |  | 201 | -0.19 | -0.98 | 0.386 | 0.745 | 1.000 | 6424 | tags=15%, list=20%, signal=19% |
| 1343 | MOTOR\_NEURON\_AXON\_GUIDANCE(GO:0008045) |  | 53 | -0.23 | -0.98 | 0.507 | 0.746 | 1.000 | 8983 | tags=28%, list=28%, signal=39% |
| 1344 | BASEMENT\_MEMBRANE(GO:0005604) |  | 51 | -0.29 | -0.98 | 0.428 | 0.747 | 1.000 | 9706 | tags=43%, list=30%, signal=62% |
| 1345 | NUCLEOTIDE\_BIOSYNTHETIC\_PROCESS(GO:0009165) |  | 209 | -0.18 | -0.98 | 0.429 | 0.746 | 1.000 | 6789 | tags=23%, list=21%, signal=29% |
| 1346 | JAK\_STAT\_CASCADE(GO:0007259) |  | 36 | -0.28 | -0.98 | 0.514 | 0.748 | 1.000 | 3170 | tags=14%, list=10%, signal=15% |
| 1347 | STAT\_CASCADE(GO:0097696) |  | 36 | -0.28 | -0.98 | 0.514 | 0.747 | 1.000 | 3170 | tags=14%, list=10%, signal=15% |
| 1348 | TISSUE\_DEVELOPMENT(GO:0009888) |  | 1496 | -0.16 | -0.98 | 0.392 | 0.748 | 1.000 | 6039 | tags=16%, list=19%, signal=18% |
| 1349 | GONAD\_DEVELOPMENT(GO:0008406) |  | 16 | -0.34 | -0.98 | 0.416 | 0.747 | 1.000 | 350 | tags=6%, list=1%, signal=6% |
| 1350 | REPRODUCTIVE\_STRUCTURE\_DEVELOPMENT(GO:0048608) |  | 16 | -0.34 | -0.98 | 0.416 | 0.747 | 1.000 | 350 | tags=6%, list=1%, signal=6% |
| 1351 | REPRODUCTIVE\_SYSTEM\_DEVELOPMENT(GO:0061458) |  | 16 | -0.34 | -0.98 | 0.416 | 0.746 | 1.000 | 350 | tags=6%, list=1%, signal=6% |
| 1352 | REGULATION\_OF\_BIOSYNTHETIC\_PROCESS(GO:0009889) |  | 2496 | -0.16 | -0.98 | 0.415 | 0.746 | 1.000 | 6145 | tags=16%, list=19%, signal=18% |
| 1353 | REGULATION\_OF\_VOLTAGE\_GATED\_CALCIUM\_CHANNEL\_ACTIVITY(GO:1901385) |  | 18 | -0.36 | -0.98 | 0.537 | 0.746 | 1.000 | 5664 | tags=28%, list=18%, signal=34% |
| 1354 | EMBRYONIC\_MORPHOGENESIS(GO:0048598) |  | 700 | -0.16 | -0.98 | 0.431 | 0.745 | 1.000 | 5996 | tags=15%, list=19%, signal=18% |
| 1355 | POSITIVE\_REGULATION\_OF\_CHROMOSOME\_ORGANIZATION(GO:2001252) |  | 29 | -0.28 | -0.98 | 0.514 | 0.746 | 1.000 | 6665 | tags=21%, list=21%, signal=26% |
| 1356 | REGULATION\_OF\_AMPA\_RECEPTOR\_ACTIVITY(GO:2000311) |  | 15 | -0.38 | -0.98 | 0.535 | 0.747 | 1.000 | 10664 | tags=53%, list=33%, signal=80% |
| 1357 | POSITIVE\_REGULATION\_OF\_LIPID\_METABOLIC\_PROCESS(GO:0045834) |  | 22 | -0.31 | -0.98 | 0.448 | 0.747 | 1.000 | 4370 | tags=27%, list=14%, signal=32% |
| 1358 | TRANSPORT\_VESICLE\_MEMBRANE(GO:0030658) |  | 93 | -0.22 | -0.98 | 0.449 | 0.748 | 1.000 | 6894 | tags=25%, list=22%, signal=31% |
| 1359 | REGULATION\_OF\_ION\_TRANSMEMBRANE\_TRANSPORT(GO:0034765) |  | 219 | -0.22 | -0.98 | 0.517 | 0.747 | 1.000 | 5936 | tags=22%, list=19%, signal=27% |
| 1360 | CELLULAR\_RESPONSE\_TO\_METAL\_ION(GO:0071248) |  | 40 | -0.32 | -0.98 | 0.481 | 0.747 | 1.000 | 8725 | tags=43%, list=27%, signal=58% |
| 1361 | NEGATIVE\_REGULATION\_OF\_CYTOSKELETON\_ORGANIZATION(GO:0051494) |  | 70 | -0.21 | -0.98 | 0.563 | 0.747 | 1.000 | 2910 | tags=9%, list=9%, signal=9% |
| 1362 | OPTOKINETIC\_BEHAVIOR(GO:0007634) |  | 19 | -0.30 | -0.98 | 0.563 | 0.747 | 1.000 | 5768 | tags=21%, list=18%, signal=26% |
| 1363 | REGULATION\_OF\_CELLULAR\_MACROMOLECULE\_BIOSYNTHETIC\_PROCESS(GO:2000112) |  | 2428 | -0.16 | -0.98 | 0.415 | 0.746 | 1.000 | 6145 | tags=16%, list=19%, signal=18% |
| 1364 | CUL3\_RING\_UBIQUITIN\_LIGASE\_COMPLEX(GO:0031463) |  | 22 | -0.28 | -0.98 | 0.483 | 0.747 | 1.000 | 11205 | tags=55%, list=35%, signal=84% |
| 1365 | CELLULAR\_DEVELOPMENTAL\_PROCESS(GO:0048869) |  | 2432 | -0.15 | -0.98 | 0.475 | 0.749 | 1.000 | 6337 | tags=17%, list=20%, signal=19% |
| 1366 | REGULATION\_OF\_CELLULAR\_BIOSYNTHETIC\_PROCESS(GO:0031326) |  | 2479 | -0.16 | -0.98 | 0.415 | 0.749 | 1.000 | 6145 | tags=16%, list=19%, signal=18% |
| 1367 | DNA\_REPAIR\_COMPLEX(GO:1990391) |  | 20 | -0.31 | -0.98 | 0.463 | 0.750 | 1.000 | 92 | tags=5%, list=0%, signal=5% |
| 1368 | RIBOSE\_PHOSPHATE\_METABOLIC\_PROCESS(GO:0019693) |  | 280 | -0.18 | -0.98 | 0.565 | 0.749 | 1.000 | 6446 | tags=20%, list=20%, signal=25% |
| 1369 | PRERIBOSOME\_\_LARGE\_SUBUNIT\_PRECURSOR(GO:0030687) |  | 21 | -0.30 | -0.98 | 0.529 | 0.749 | 1.000 | 5860 | tags=19%, list=18%, signal=23% |
| 1370 | CILIUM(GO:0005929) |  | 278 | -0.21 | -0.97 | 0.541 | 0.750 | 1.000 | 5443 | tags=19%, list=17%, signal=22% |
| 1371 | CALCIUM\_ION\_BINDING(GO:0005509) |  | 632 | -0.19 | -0.97 | 0.390 | 0.751 | 1.000 | 3827 | tags=11%, list=12%, signal=13% |
| 1372 | CYTOPLASMIC\_VESICLE(GO:0031410) |  | 793 | -0.15 | -0.97 | 0.517 | 0.751 | 1.000 | 4248 | tags=11%, list=13%, signal=12% |
| 1373 | RIBONUCLEOTIDE\_BIOSYNTHETIC\_PROCESS(GO:0009260) |  | 177 | -0.18 | -0.97 | 0.548 | 0.751 | 1.000 | 6379 | tags=21%, list=20%, signal=26% |
| 1374 | PHOSPHATIDYLINOSITOL\_PHOSPHOLIPASE\_C\_ACTIVITY(GO:0004435) |  | 17 | -0.28 | -0.97 | 0.345 | 0.752 | 1.000 | 4144 | tags=18%, list=13%, signal=20% |
| 1375 | PHOSPHOLIPASE\_C\_ACTIVITY(GO:0004629) |  | 17 | -0.28 | -0.97 | 0.345 | 0.751 | 1.000 | 4144 | tags=18%, list=13%, signal=20% |
| 1376 | PROTEIN\_HYDROXYLATION(GO:0018126) |  | 22 | -0.29 | -0.97 | 0.431 | 0.752 | 1.000 | 6718 | tags=27%, list=21%, signal=35% |
| 1377 | EUKARYOTIC\_48S\_PREINITIATION\_COMPLEX(GO:0033290) |  | 17 | -0.27 | -0.97 | 0.588 | 0.752 | 1.000 | 10745 | tags=47%, list=34%, signal=71% |
| 1378 | POSITIVE\_REGULATION\_OF\_CYTOSOLIC\_CALCIUM\_ION\_CONCENTRATION(GO:0007204) |  | 114 | -0.22 | -0.97 | 0.593 | 0.757 | 1.000 | 6506 | tags=26%, list=20%, signal=33% |
| 1379 | INORGANIC\_ION\_HOMEOSTASIS(GO:0098771) |  | 358 | -0.18 | -0.97 | 0.589 | 0.758 | 1.000 | 5936 | tags=18%, list=19%, signal=22% |
| 1380 | MLL1\_COMPLEX(GO:0071339) |  | 15 | -0.24 | -0.97 | 0.480 | 0.758 | 1.000 | 24187 | tags=100%, list=76%, signal=414% |
| 1381 | MLL1\_2\_COMPLEX(GO:0044665) |  | 15 | -0.24 | -0.97 | 0.480 | 0.757 | 1.000 | 24187 | tags=100%, list=76%, signal=414% |
| 1382 | PATTERN\_BINDING(GO:0001871) |  | 20 | -0.30 | -0.97 | 0.466 | 0.757 | 1.000 | 10 | tags=5%, list=0%, signal=5% |
| 1383 | POLYSACCHARIDE\_BINDING(GO:0030247) |  | 20 | -0.30 | -0.97 | 0.466 | 0.757 | 1.000 | 10 | tags=5%, list=0%, signal=5% |
| 1384 | IMMUNE\_RESPONSE\_REGULATING\_SIGNALING\_PATHWAY(GO:0002764) |  | 92 | -0.25 | -0.97 | 0.542 | 0.756 | 1.000 | 5470 | tags=20%, list=17%, signal=24% |
| 1385 | IMP\_METABOLIC\_PROCESS(GO:0046040) |  | 17 | -0.30 | -0.97 | 0.527 | 0.756 | 1.000 | 8314 | tags=41%, list=26%, signal=56% |
| 1386 | CELLULAR\_RESPONSE\_TO\_INORGANIC\_SUBSTANCE(GO:0071241) |  | 41 | -0.31 | -0.97 | 0.481 | 0.756 | 1.000 | 8725 | tags=41%, list=27%, signal=57% |
| 1387 | ACTIN\_FILAMENT\_BUNDLE\_ORGANIZATION(GO:0061572) |  | 59 | -0.24 | -0.97 | 0.517 | 0.756 | 1.000 | 7160 | tags=31%, list=22%, signal=39% |
| 1388 | POSITIVE\_REGULATION\_OF\_PROTEIN\_POLYMERIZATION(GO:0032273) |  | 79 | -0.20 | -0.97 | 0.550 | 0.756 | 1.000 | 3541 | tags=11%, list=11%, signal=13% |
| 1389 | EAR\_MORPHOGENESIS(GO:0042471) |  | 87 | -0.20 | -0.97 | 0.437 | 0.756 | 1.000 | 8983 | tags=26%, list=28%, signal=37% |
| 1390 | SODIUM\_ION\_HOMEOSTASIS(GO:0055078) |  | 26 | -0.28 | -0.97 | 0.385 | 0.755 | 1.000 | 2181 | tags=12%, list=7%, signal=12% |
| 1391 | REGULATION\_OF\_ACTOMYOSIN\_STRUCTURE\_ORGANIZATION(GO:0110020) |  | 19 | -0.36 | -0.97 | 0.513 | 0.755 | 1.000 | 8089 | tags=47%, list=25%, signal=63% |
| 1392 | REGULATION\_OF\_MACROMOLECULE\_BIOSYNTHETIC\_PROCESS(GO:0010556) |  | 2434 | -0.16 | -0.97 | 0.477 | 0.755 | 1.000 | 6145 | tags=16%, list=19%, signal=18% |
| 1393 | CATION\_TRANSMEMBRANE\_TRANSPORT(GO:0098655) |  | 510 | -0.20 | -0.97 | 0.473 | 0.755 | 1.000 | 5996 | tags=19%, list=19%, signal=23% |
| 1394 | NEGATIVE\_REGULATION\_OF\_TRANSMEMBRANE\_RECEPTOR\_PROTEIN\_SERINE\_THREONINE\_KINASE\_SIGNALING\_PATHWAY(GO:0090101) |  | 61 | -0.23 | -0.97 | 0.506 | 0.755 | 1.000 | 1934 | tags=7%, list=6%, signal=7% |
| 1395 | TRANSFERASE\_ACTIVITY\_\_TRANSFERRING\_PENTOSYL\_GROUPS(GO:0016763) |  | 58 | -0.21 | -0.97 | 0.549 | 0.755 | 1.000 | 4387 | tags=16%, list=14%, signal=18% |
| 1396 | CELLULAR\_RESPONSE\_TO\_DRUG(GO:0035690) |  | 33 | -0.31 | -0.97 | 0.521 | 0.755 | 1.000 | 5141 | tags=27%, list=16%, signal=32% |
| 1397 | PROTEIN\_HOMOOLIGOMERIZATION(GO:0051260) |  | 88 | -0.20 | -0.97 | 0.630 | 0.754 | 1.000 | 7269 | tags=28%, list=23%, signal=37% |
| 1398 | CATION\_TRANSPORT(GO:0006812) |  | 684 | -0.20 | -0.97 | 0.558 | 0.756 | 1.000 | 5936 | tags=19%, list=19%, signal=23% |
| 1399 | ENDODERM\_DEVELOPMENT(GO:0007492) |  | 61 | -0.24 | -0.97 | 0.463 | 0.756 | 1.000 | 1743 | tags=8%, list=5%, signal=9% |
| 1400 | RNA\_POLYMERASE\_II\_TRANSCRIPTION\_FACTOR\_COMPLEX(GO:0090575) |  | 83 | -0.19 | -0.96 | 0.413 | 0.760 | 1.000 | 6949 | tags=24%, list=22%, signal=31% |
| 1401 | EUKARYOTIC\_TRANSLATION\_INITIATION\_FACTOR\_3\_COMPLEX(GO:0005852) |  | 16 | -0.29 | -0.96 | 0.505 | 0.760 | 1.000 | 10745 | tags=50%, list=34%, signal=75% |
| 1402 | SYSTEM\_PROCESS(GO:0003008) |  | 777 | -0.19 | -0.96 | 0.540 | 0.759 | 1.000 | 6334 | tags=18%, list=20%, signal=22% |
| 1403 | HOMEOSTASIS\_OF\_NUMBER\_OF\_CELLS(GO:0048872) |  | 143 | -0.19 | -0.96 | 0.476 | 0.759 | 1.000 | 9290 | tags=36%, list=29%, signal=50% |
| 1404 | METAPHASE\_ANAPHASE\_TRANSITION\_OF\_MITOTIC\_CELL\_CYCLE(GO:0007091) |  | 27 | -0.31 | -0.96 | 0.476 | 0.759 | 1.000 | 2255 | tags=7%, list=7%, signal=8% |
| 1405 | METAPHASE\_ANAPHASE\_TRANSITION\_OF\_CELL\_CYCLE(GO:0044784) |  | 27 | -0.31 | -0.96 | 0.476 | 0.758 | 1.000 | 2255 | tags=7%, list=7%, signal=8% |
| 1406 | ACTIN\_NUCLEATION(GO:0045010) |  | 51 | -0.22 | -0.96 | 0.456 | 0.759 | 1.000 | 9587 | tags=29%, list=30%, signal=42% |
| 1407 | NUCLEIC\_ACID\_BINDING(GO:0003676) |  | 2931 | -0.15 | -0.96 | 0.469 | 0.759 | 1.000 | 6143 | tags=15%, list=19%, signal=17% |
| 1408 | UNSATURATED\_FATTY\_ACID\_BIOSYNTHETIC\_PROCESS(GO:0006636) |  | 31 | -0.32 | -0.96 | 0.551 | 0.758 | 1.000 | 5844 | tags=32%, list=18%, signal=39% |
| 1409 | POSITIVE\_REGULATION\_OF\_JNK\_CASCADE(GO:0046330) |  | 20 | -0.34 | -0.96 | 0.493 | 0.758 | 1.000 | 2715 | tags=10%, list=9%, signal=11% |
| 1410 | TRANSFERASE\_ACTIVITY\_\_TRANSFERRING\_GLYCOSYL\_GROUPS(GO:0016757) |  | 335 | -0.21 | -0.96 | 0.407 | 0.758 | 1.000 | 5932 | tags=20%, list=19%, signal=25% |
| 1411 | BASOLATERAL\_PLASMA\_MEMBRANE(GO:0016323) |  | 69 | -0.20 | -0.96 | 0.487 | 0.757 | 1.000 | 3665 | tags=13%, list=11%, signal=15% |
| 1412 | GLUCOSE\_METABOLIC\_PROCESS(GO:0006006) |  | 69 | -0.24 | -0.96 | 0.533 | 0.758 | 1.000 | 4199 | tags=16%, list=13%, signal=18% |
| 1413 | REGULATION\_OF\_APOPTOTIC\_SIGNALING\_PATHWAY(GO:2001233) |  | 57 | -0.20 | -0.96 | 0.454 | 0.758 | 1.000 | 5118 | tags=16%, list=16%, signal=19% |
| 1414 | MITOCHONDRION(GO:0005739) |  | 991 | -0.17 | -0.96 | 0.375 | 0.760 | 1.000 | 6570 | tags=19%, list=21%, signal=24% |
| 1415 | MICROTUBULE\_BINDING(GO:0008017) |  | 207 | -0.17 | -0.96 | 0.597 | 0.760 | 1.000 | 9587 | tags=31%, list=30%, signal=44% |
| 1416 | NEURON\_PROJECTION\_MORPHOGENESIS(GO:0048812) |  | 459 | -0.18 | -0.96 | 0.505 | 0.762 | 1.000 | 8289 | tags=24%, list=26%, signal=31% |
| 1417 | REGULATION\_OF\_CHROMOSOME\_SEGREGATION(GO:0051983) |  | 42 | -0.30 | -0.96 | 0.442 | 0.762 | 1.000 | 2491 | tags=10%, list=8%, signal=10% |
| 1418 | EYE\_MORPHOGENESIS(GO:0048592) |  | 208 | -0.19 | -0.96 | 0.558 | 0.762 | 1.000 | 9211 | tags=29%, list=29%, signal=41% |
| 1419 | PEPTIDYL\_PROLINE\_MODIFICATION(GO:0018208) |  | 37 | -0.26 | -0.96 | 0.506 | 0.762 | 1.000 | 6231 | tags=22%, list=20%, signal=27% |
| 1420 | GOLGI\_APPARATUS\_PART(GO:0044431) |  | 457 | -0.16 | -0.96 | 0.490 | 0.761 | 1.000 | 6694 | tags=18%, list=21%, signal=23% |
| 1421 | PROTEIN\_DEPHOSPHORYLATION(GO:0006470) |  | 169 | -0.17 | -0.96 | 0.525 | 0.761 | 1.000 | 4125 | tags=11%, list=13%, signal=13% |
| 1422 | INORGANIC\_CATION\_TRANSMEMBRANE\_TRANSPORT(GO:0098662) |  | 471 | -0.19 | -0.96 | 0.531 | 0.765 | 1.000 | 6833 | tags=21%, list=21%, signal=27% |
| 1423 | CELL\_CYCLE\_PHASE\_TRANSITION(GO:0044770) |  | 137 | -0.18 | -0.96 | 0.442 | 0.765 | 1.000 | 2255 | tags=7%, list=7%, signal=7% |
| 1424 | ORGANOPHOSPHATE\_METABOLIC\_PROCESS(GO:0019637) |  | 665 | -0.17 | -0.96 | 0.625 | 0.765 | 1.000 | 6520 | tags=20%, list=20%, signal=24% |
| 1425 | N\_GLYCAN\_PROCESSING(GO:0006491) |  | 16 | -0.31 | -0.96 | 0.510 | 0.765 | 1.000 | 6199 | tags=25%, list=19%, signal=31% |
| 1426 | REGULATION\_OF\_TRANSCRIPTION\_\_DNA\_TEMPLATED(GO:0006355) |  | 2201 | -0.17 | -0.96 | 0.538 | 0.766 | 1.000 | 6145 | tags=16%, list=19%, signal=18% |
| 1427 | REGULATION\_OF\_NUCLEIC\_ACID\_TEMPLATED\_TRANSCRIPTION(GO:1903506) |  | 2202 | -0.17 | -0.96 | 0.538 | 0.766 | 1.000 | 6145 | tags=16%, list=19%, signal=18% |
| 1428 | REGULATION\_OF\_CELL\_COMMUNICATION(GO:0010646) |  | 1260 | -0.16 | -0.96 | 0.559 | 0.766 | 1.000 | 5713 | tags=14%, list=18%, signal=17% |
| 1429 | SCHWANN\_CELL\_DEVELOPMENT(GO:0014044) |  | 23 | -0.27 | -0.96 | 0.514 | 0.765 | 1.000 | 354 | tags=4%, list=1%, signal=4% |
| 1430 | REGULATION\_OF\_RNA\_BIOSYNTHETIC\_PROCESS(GO:2001141) |  | 2203 | -0.17 | -0.96 | 0.538 | 0.765 | 1.000 | 6145 | tags=16%, list=19%, signal=18% |
| 1431 | NEGATIVE\_REGULATION\_OF\_ACTIN\_FILAMENT\_POLYMERIZATION(GO:0030837) |  | 54 | -0.24 | -0.96 | 0.577 | 0.765 | 1.000 | 2910 | tags=11%, list=9%, signal=12% |
| 1432 | NEGATIVE\_REGULATION\_OF\_PROTEIN\_POLYMERIZATION(GO:0032272) |  | 54 | -0.24 | -0.96 | 0.577 | 0.764 | 1.000 | 2910 | tags=11%, list=9%, signal=12% |
| 1433 | REGULATION\_OF\_MITOTIC\_NUCLEAR\_DIVISION(GO:0007088) |  | 59 | -0.22 | -0.95 | 0.522 | 0.764 | 1.000 | 2255 | tags=7%, list=7%, signal=7% |
| 1434 | REGULATION\_OF\_SIGNALING(GO:0023051) |  | 1271 | -0.16 | -0.95 | 0.559 | 0.764 | 1.000 | 5713 | tags=14%, list=18%, signal=17% |
| 1435 | POSITIVE\_REGULATION\_OF\_CELL\_CYCLE\_PHASE\_TRANSITION(GO:1901989) |  | 23 | -0.28 | -0.95 | 0.486 | 0.764 | 1.000 | 5919 | tags=26%, list=19%, signal=32% |
| 1436 | NEGATIVE\_CHEMOTAXIS(GO:0050919) |  | 37 | -0.24 | -0.95 | 0.489 | 0.764 | 1.000 | 4156 | tags=14%, list=13%, signal=16% |
| 1437 | NUCLEOBASE\_CONTAINING\_COMPOUND\_CATABOLIC\_PROCESS(GO:0034655) |  | 244 | -0.17 | -0.95 | 0.422 | 0.764 | 1.000 | 6789 | tags=20%, list=21%, signal=25% |
| 1438 | RETROGRADE\_VESICLE\_MEDIATED\_TRANSPORT\_\_GOLGI\_TO\_ER(GO:0006890) |  | 33 | -0.25 | -0.95 | 0.526 | 0.764 | 1.000 | 3090 | tags=12%, list=10%, signal=13% |
| 1439 | REGULATION\_OF\_NUCLEOBASE\_CONTAINING\_COMPOUND\_METABOLIC\_PROCESS(GO:0019219) |  | 2478 | -0.16 | -0.95 | 0.570 | 0.765 | 1.000 | 4599 | tags=11%, list=14%, signal=12% |
| 1440 | CHROMATIN(GO:0000785) |  | 248 | -0.17 | -0.95 | 0.544 | 0.765 | 1.000 | 6986 | tags=18%, list=22%, signal=23% |
| 1441 | SPERMATOGENESIS(GO:0007283) |  | 79 | -0.23 | -0.95 | 0.568 | 0.764 | 1.000 | 7087 | tags=20%, list=22%, signal=26% |
| 1442 | MITOCHONDRIAL\_PART(GO:0044429) |  | 562 | -0.18 | -0.95 | 0.372 | 0.764 | 1.000 | 6512 | tags=18%, list=20%, signal=22% |
| 1443 | HISTONE\_MODIFICATION(GO:0016570) |  | 213 | -0.14 | -0.95 | 0.534 | 0.765 | 1.000 | 7005 | tags=15%, list=22%, signal=19% |
| 1444 | ANIMAL\_ORGAN\_REGENERATION(GO:0031100) |  | 23 | -0.29 | -0.95 | 0.590 | 0.764 | 1.000 | 212 | tags=4%, list=1%, signal=4% |
| 1445 | PLASMA\_MEMBRANE\_BOUNDED\_CELL\_PROJECTION\_MORPHOGENESIS(GO:0120039) |  | 460 | -0.18 | -0.95 | 0.505 | 0.764 | 1.000 | 8289 | tags=23%, list=26%, signal=31% |
| 1446 | CELL\_PROJECTION\_MORPHOGENESIS(GO:0048858) |  | 463 | -0.18 | -0.95 | 0.505 | 0.764 | 1.000 | 8289 | tags=24%, list=26%, signal=31% |
| 1447 | MONOSACCHARIDE\_BINDING(GO:0048029) |  | 38 | -0.26 | -0.95 | 0.567 | 0.765 | 1.000 | 3477 | tags=16%, list=11%, signal=18% |
| 1448 | INTRINSIC\_COMPONENT\_OF\_MITOCHONDRIAL\_MEMBRANE(GO:0098573) |  | 64 | -0.23 | -0.95 | 0.514 | 0.767 | 1.000 | 8070 | tags=28%, list=25%, signal=38% |
| 1449 | PRIMARY\_ACTIVE\_TRANSMEMBRANE\_TRANSPORTER\_ACTIVITY(GO:0015399) |  | 92 | -0.19 | -0.95 | 0.526 | 0.767 | 1.000 | 2382 | tags=8%, list=7%, signal=8% |
| 1450 | REGULATION\_OF\_SYNAPTIC\_TRANSMISSION\_\_GLUTAMATERGIC(GO:0051966) |  | 30 | -0.33 | -0.95 | 0.579 | 0.770 | 1.000 | 9325 | tags=40%, list=29%, signal=56% |
| 1451 | ACID\_SECRETION(GO:0046717) |  | 15 | -0.33 | -0.95 | 0.387 | 0.769 | 1.000 | 5373 | tags=40%, list=17%, signal=48% |
| 1452 | EMBRYO\_DEVELOPMENT(GO:0009790) |  | 1134 | -0.14 | -0.95 | 0.446 | 0.769 | 1.000 | 6029 | tags=14%, list=19%, signal=17% |
| 1453 | POSITIVE\_REGULATION\_OF\_PROTEIN\_MODIFICATION\_BY\_SMALL\_PROTEIN\_CONJUGATION\_OR\_REMOVAL(GO:1903322) |  | 28 | -0.23 | -0.95 | 0.419 | 0.770 | 1.000 | 3560 | tags=11%, list=11%, signal=12% |
| 1454 | EMBRYO\_DEVELOPMENT\_ENDING\_IN\_BIRTH\_OR\_EGG\_HATCHING(GO:0009792) |  | 501 | -0.14 | -0.95 | 0.398 | 0.772 | 1.000 | 6421 | tags=15%, list=20%, signal=18% |
| 1455 | REGULATION\_OF\_MYELOID\_CELL\_DIFFERENTIATION(GO:0045637) |  | 30 | -0.30 | -0.95 | 0.576 | 0.772 | 1.000 | 4847 | tags=27%, list=15%, signal=31% |
| 1456 | MITOTIC\_CELL\_CYCLE\_PHASE\_TRANSITION(GO:0044772) |  | 130 | -0.17 | -0.95 | 0.479 | 0.772 | 1.000 | 1606 | tags=5%, list=5%, signal=6% |
| 1457 | COVALENT\_CHROMATIN\_MODIFICATION(GO:0016569) |  | 215 | -0.14 | -0.95 | 0.488 | 0.772 | 1.000 | 7005 | tags=14%, list=22%, signal=18% |
| 1458 | RENAL\_TUBULE\_DEVELOPMENT(GO:0061326) |  | 25 | -0.31 | -0.95 | 0.472 | 0.771 | 1.000 | 9242 | tags=44%, list=29%, signal=62% |
| 1459 | SIGNALING\_RECEPTOR\_BINDING(GO:0005102) |  | 772 | -0.18 | -0.95 | 0.471 | 0.771 | 1.000 | 4649 | tags=13%, list=15%, signal=15% |
| 1460 | OXYGEN\_CARRIER\_ACTIVITY(GO:0005344) |  | 17 | -0.34 | -0.95 | 0.509 | 0.771 | 1.000 | 1987 | tags=18%, list=6%, signal=19% |
| 1461 | OXYGEN\_BINDING(GO:0019825) |  | 17 | -0.34 | -0.95 | 0.509 | 0.771 | 1.000 | 1987 | tags=18%, list=6%, signal=19% |
| 1462 | TRANSCRIPTION\_BY\_RNA\_POLYMERASE\_II(GO:0006366) |  | 1794 | -0.17 | -0.95 | 0.544 | 0.771 | 1.000 | 6145 | tags=16%, list=19%, signal=19% |
| 1463 | RESPONSE\_TO\_METAL\_ION(GO:0010038) |  | 86 | -0.24 | -0.95 | 0.551 | 0.771 | 1.000 | 6675 | tags=26%, list=21%, signal=32% |
| 1464 | ATPASE\_ACTIVITY\_\_COUPLED\_TO\_TRANSMEMBRANE\_MOVEMENT\_OF\_SUBSTANCES(GO:0042626) |  | 91 | -0.19 | -0.95 | 0.590 | 0.770 | 1.000 | 2382 | tags=8%, list=7%, signal=8% |
| 1465 | P\_P\_BOND\_HYDROLYSIS\_DRIVEN\_TRANSMEMBRANE\_TRANSPORTER\_ACTIVITY(GO:0015405) |  | 91 | -0.19 | -0.95 | 0.590 | 0.770 | 1.000 | 2382 | tags=8%, list=7%, signal=8% |
| 1466 | ATPASE\_ACTIVITY\_\_COUPLED\_TO\_MOVEMENT\_OF\_SUBSTANCES(GO:0043492) |  | 91 | -0.19 | -0.95 | 0.590 | 0.769 | 1.000 | 2382 | tags=8%, list=7%, signal=8% |
| 1467 | HEMOGLOBIN\_METABOLIC\_PROCESS(GO:0020027) |  | 17 | -0.26 | -0.94 | 0.535 | 0.772 | 1.000 | 9290 | tags=47%, list=29%, signal=66% |
| 1468 | CHORDATE\_EMBRYONIC\_DEVELOPMENT(GO:0043009) |  | 499 | -0.14 | -0.94 | 0.479 | 0.772 | 1.000 | 6421 | tags=14%, list=20%, signal=18% |
| 1469 | CYCLIC\_NUCLEOTIDE\_BINDING(GO:0030551) |  | 25 | -0.29 | -0.94 | 0.515 | 0.772 | 1.000 | 5865 | tags=32%, list=18%, signal=39% |
| 1470 | OXIDOREDUCTASE\_ACTIVITY\_\_ACTING\_ON\_NAD(P)H\_\_QUINONE\_OR\_SIMILAR\_COMPOUND\_AS\_ACCEPTOR(GO:0016655) |  | 22 | -0.29 | -0.94 | 0.616 | 0.772 | 1.000 | 344 | tags=5%, list=1%, signal=5% |
| 1471 | ANTERIOR\_POSTERIOR\_AXIS\_SPECIFICATION(GO:0009948) |  | 31 | -0.26 | -0.94 | 0.519 | 0.773 | 1.000 | 1253 | tags=6%, list=4%, signal=7% |
| 1472 | RNA\_SPLICING\_\_VIA\_TRANSESTERIFICATION\_REACTIONS(GO:0000375) |  | 218 | -0.15 | -0.94 | 0.554 | 0.775 | 1.000 | 11700 | tags=35%, list=37%, signal=55% |
| 1473 | RNA\_SPLICING\_\_VIA\_TRANSESTERIFICATION\_REACTIONS\_WITH\_BULGED\_ADENOSINE\_AS\_NUCLEOPHILE(GO:0000377) |  | 218 | -0.15 | -0.94 | 0.554 | 0.774 | 1.000 | 11700 | tags=35%, list=37%, signal=55% |
| 1474 | MRNA\_SPLICING\_\_VIA\_SPLICEOSOME(GO:0000398) |  | 218 | -0.15 | -0.94 | 0.554 | 0.774 | 1.000 | 11700 | tags=35%, list=37%, signal=55% |
| 1475 | POTASSIUM\_ION\_IMPORT\_ACROSS\_PLASMA\_MEMBRANE(GO:1990573) |  | 55 | -0.26 | -0.94 | 0.549 | 0.774 | 1.000 | 7949 | tags=29%, list=25%, signal=39% |
| 1476 | MICROTUBULE\_BUNDLE\_FORMATION(GO:0001578) |  | 56 | -0.27 | -0.94 | 0.532 | 0.773 | 1.000 | 8258 | tags=34%, list=26%, signal=46% |
| 1477 | PROTEIN\_DEMETHYLATION(GO:0006482) |  | 21 | -0.25 | -0.94 | 0.508 | 0.773 | 1.000 | 6718 | tags=19%, list=21%, signal=24% |
| 1478 | PROTEIN\_DEALKYLATION(GO:0008214) |  | 21 | -0.25 | -0.94 | 0.508 | 0.772 | 1.000 | 6718 | tags=19%, list=21%, signal=24% |
| 1479 | OXIDOREDUCTASE\_ACTIVITY\_\_ACTING\_ON\_PEROXIDE\_AS\_ACCEPTOR(GO:0016684) |  | 48 | -0.25 | -0.94 | 0.330 | 0.772 | 1.000 | 5844 | tags=23%, list=18%, signal=28% |
| 1480 | TUBE\_FORMATION(GO:0035148) |  | 69 | -0.22 | -0.94 | 0.586 | 0.771 | 1.000 | 10821 | tags=38%, list=34%, signal=57% |
| 1481 | ACTIN\_FILAMENT\_BUNDLE\_ASSEMBLY(GO:0051017) |  | 55 | -0.23 | -0.94 | 0.515 | 0.772 | 1.000 | 7160 | tags=31%, list=22%, signal=40% |
| 1482 | G2\_M\_TRANSITION\_OF\_MITOTIC\_CELL\_CYCLE(GO:0000086) |  | 37 | -0.24 | -0.94 | 0.470 | 0.772 | 1.000 | 3517 | tags=14%, list=11%, signal=15% |
| 1483 | ANIMAL\_ORGAN\_DEVELOPMENT(GO:0048513) |  | 2582 | -0.14 | -0.94 | 0.607 | 0.772 | 1.000 | 6428 | tags=16%, list=20%, signal=18% |
| 1484 | REGULATION\_OF\_GENE\_EXPRESSION(GO:0010468) |  | 2664 | -0.15 | -0.94 | 0.605 | 0.772 | 1.000 | 5792 | tags=14%, list=18%, signal=16% |
| 1485 | PHOSPHOLIPID\_BINDING(GO:0005543) |  | 272 | -0.16 | -0.94 | 0.567 | 0.771 | 1.000 | 3414 | tags=8%, list=11%, signal=9% |
| 1486 | ORGANELLE\_INNER\_MEMBRANE(GO:0019866) |  | 307 | -0.18 | -0.94 | 0.423 | 0.773 | 1.000 | 7159 | tags=22%, list=22%, signal=29% |
| 1487 | DIGESTIVE\_TRACT\_DEVELOPMENT(GO:0048565) |  | 93 | -0.18 | -0.94 | 0.519 | 0.776 | 1.000 | 3715 | tags=9%, list=12%, signal=10% |
| 1488 | REGULATION\_OF\_RESPONSE\_TO\_ENDOPLASMIC\_RETICULUM\_STRESS(GO:1905897) |  | 15 | -0.24 | -0.94 | 0.589 | 0.776 | 1.000 | 7982 | tags=27%, list=25%, signal=36% |
| 1489 | GLYCOLIPID\_METABOLIC\_PROCESS(GO:0006664) |  | 67 | -0.21 | -0.94 | 0.629 | 0.776 | 1.000 | 240 | tags=3%, list=1%, signal=3% |
| 1490 | LIPOSACCHARIDE\_METABOLIC\_PROCESS(GO:1903509) |  | 67 | -0.21 | -0.94 | 0.629 | 0.776 | 1.000 | 240 | tags=3%, list=1%, signal=3% |
| 1491 | HEPARAN\_SULFATE\_6\_O\_SULFOTRANSFERASE\_ACTIVITY(GO:0017095) |  | 50 | -0.23 | -0.94 | 0.688 | 0.776 | 1.000 | 5477 | tags=18%, list=17%, signal=22% |
| 1492 | CHOLESTEROL\_SULFOTRANSFERASE\_ACTIVITY(GO:0051922) |  | 50 | -0.23 | -0.94 | 0.688 | 0.775 | 1.000 | 5477 | tags=18%, list=17%, signal=22% |
| 1493 | ALCOHOL\_SULFOTRANSFERASE\_ACTIVITY(GO:0004027) |  | 50 | -0.23 | -0.94 | 0.688 | 0.775 | 1.000 | 5477 | tags=18%, list=17%, signal=22% |
| 1494 | CARBONATE\_DEHYDRATASE\_ACTIVITY(GO:0004089) |  | 18 | -0.34 | -0.94 | 0.490 | 0.775 | 1.000 | 3637 | tags=17%, list=11%, signal=19% |
| 1495 | GOLGI\_MEMBRANE(GO:0000139) |  | 352 | -0.16 | -0.94 | 0.575 | 0.775 | 1.000 | 6694 | tags=18%, list=21%, signal=22% |
| 1496 | PROTEIN\_HOMODIMERIZATION\_ACTIVITY(GO:0042803) |  | 92 | -0.18 | -0.94 | 0.483 | 0.776 | 1.000 | 11029 | tags=42%, list=35%, signal=65% |
| 1497 | REGULATION\_OF\_TRANSCRIPTION\_BY\_RNA\_POLYMERASE\_II(GO:0006357) |  | 1743 | -0.17 | -0.94 | 0.545 | 0.776 | 1.000 | 6532 | tags=17%, list=20%, signal=20% |
| 1498 | PROTEASOME\_COMPLEX(GO:0000502) |  | 55 | -0.23 | -0.93 | 0.518 | 0.777 | 1.000 | 2107 | tags=9%, list=7%, signal=10% |
| 1499 | ENDOPEPTIDASE\_COMPLEX(GO:1905369) |  | 55 | -0.23 | -0.93 | 0.518 | 0.776 | 1.000 | 2107 | tags=9%, list=7%, signal=10% |
| 1500 | TRANSCRIPTION\_FACTOR\_TFIID\_COMPLEX(GO:0005669) |  | 16 | -0.25 | -0.93 | 0.513 | 0.776 | 1.000 | 11270 | tags=44%, list=35%, signal=68% |
| 1501 | REGULATION\_OF\_EXOCYTOSIS(GO:0017157) |  | 80 | -0.22 | -0.93 | 0.629 | 0.777 | 1.000 | 4370 | tags=18%, list=14%, signal=20% |
| 1502 | WNT\_PROTEIN\_BINDING(GO:0017147) |  | 27 | -0.28 | -0.93 | 0.513 | 0.777 | 1.000 | 1822 | tags=7%, list=6%, signal=8% |
| 1503 | PYRIMIDINE\_NUCLEOBASE\_METABOLIC\_PROCESS(GO:0006206) |  | 18 | -0.31 | -0.93 | 0.503 | 0.777 | 1.000 | 8837 | tags=39%, list=28%, signal=54% |
| 1504 | CILIUM\_ORGANIZATION(GO:0044782) |  | 292 | -0.18 | -0.93 | 0.583 | 0.777 | 1.000 | 5996 | tags=15%, list=19%, signal=18% |
| 1505 | MIDBRAIN\_DEVELOPMENT(GO:0030901) |  | 34 | -0.27 | -0.93 | 0.495 | 0.776 | 1.000 | 2477 | tags=9%, list=8%, signal=10% |
| 1506 | MITOCHONDRION\_ORGANIZATION(GO:0007005) |  | 224 | -0.19 | -0.93 | 0.480 | 0.776 | 1.000 | 6351 | tags=18%, list=20%, signal=22% |
| 1507 | OXIDOREDUCTION\_COENZYME\_METABOLIC\_PROCESS(GO:0006733) |  | 94 | -0.22 | -0.93 | 0.458 | 0.777 | 1.000 | 6789 | tags=24%, list=21%, signal=31% |
| 1508 | HEXOSE\_METABOLIC\_PROCESS(GO:0019318) |  | 97 | -0.24 | -0.93 | 0.507 | 0.777 | 1.000 | 6328 | tags=24%, list=20%, signal=29% |
| 1509 | CELL\_MIGRATION\_INVOLVED\_IN\_HEART\_FORMATION(GO:0060974) |  | 15 | -0.27 | -0.93 | 0.544 | 0.777 | 1.000 | 7777 | tags=27%, list=24%, signal=35% |
| 1510 | AMINOACYL\_TRNA\_LIGASE\_ACTIVITY(GO:0004812) |  | 37 | -0.20 | -0.93 | 0.535 | 0.777 | 1.000 | 10351 | tags=49%, list=32%, signal=72% |
| 1511 | LIGASE\_ACTIVITY\_\_FORMING\_CARBON\_OXYGEN\_BONDS(GO:0016875) |  | 37 | -0.20 | -0.93 | 0.535 | 0.777 | 1.000 | 10351 | tags=49%, list=32%, signal=72% |
| 1512 | SUBSTRATE\_ADHESION\_DEPENDENT\_CELL\_SPREADING(GO:0034446) |  | 40 | -0.23 | -0.93 | 0.670 | 0.776 | 1.000 | 7129 | tags=25%, list=22%, signal=32% |
| 1513 | LOCOMOTION(GO:0040011) |  | 947 | -0.16 | -0.93 | 0.633 | 0.778 | 1.000 | 4471 | tags=11%, list=14%, signal=13% |
| 1514 | GLUTAMATE\_RECEPTOR\_SIGNALING\_PATHWAY(GO:0007215) |  | 40 | -0.31 | -0.93 | 0.507 | 0.778 | 1.000 | 9325 | tags=43%, list=29%, signal=60% |
| 1515 | NEGATIVE\_REGULATION\_OF\_DEVELOPMENTAL\_GROWTH(GO:0048640) |  | 45 | -0.27 | -0.93 | 0.517 | 0.777 | 1.000 | 3976 | tags=13%, list=12%, signal=15% |
| 1516 | DIVALENT\_INORGANIC\_CATION\_HOMEOSTASIS(GO:0072507) |  | 221 | -0.19 | -0.93 | 0.658 | 0.777 | 1.000 | 5936 | tags=19%, list=19%, signal=24% |
| 1517 | REGULATION\_OF\_MUSCLE\_CELL\_DIFFERENTIATION(GO:0051147) |  | 23 | -0.27 | -0.93 | 0.527 | 0.778 | 1.000 | 3858 | tags=13%, list=12%, signal=15% |
| 1518 | NON\_CANONICAL\_WNT\_SIGNALING\_PATHWAY(GO:0035567) |  | 42 | -0.24 | -0.93 | 0.470 | 0.777 | 1.000 | 1822 | tags=5%, list=6%, signal=5% |
| 1519 | TRANSLATION\_PREINITIATION\_COMPLEX(GO:0070993) |  | 19 | -0.27 | -0.93 | 0.625 | 0.778 | 1.000 | 10745 | tags=42%, list=34%, signal=63% |
| 1520 | REGULATION\_OF\_EMBRYONIC\_DEVELOPMENT(GO:0045995) |  | 87 | -0.22 | -0.93 | 0.535 | 0.778 | 1.000 | 8645 | tags=28%, list=27%, signal=38% |
| 1521 | CELL\_DIFFERENTIATION\_INVOLVED\_IN\_KIDNEY\_DEVELOPMENT(GO:0061005) |  | 41 | -0.26 | -0.93 | 0.515 | 0.778 | 1.000 | 10981 | tags=44%, list=34%, signal=67% |
| 1522 | RESPONSE\_TO\_WOUNDING(GO:0009611) |  | 272 | -0.18 | -0.93 | 0.533 | 0.779 | 1.000 | 5720 | tags=17%, list=18%, signal=20% |
| 1523 | POSITIVE\_REGULATION\_OF\_ACTIN\_FILAMENT\_BUNDLE\_ASSEMBLY(GO:0032233) |  | 15 | -0.35 | -0.93 | 0.463 | 0.779 | 1.000 | 8089 | tags=47%, list=25%, signal=62% |
| 1524 | MONONUCLEAR\_CELL\_MIGRATION(GO:0071674) |  | 26 | -0.31 | -0.93 | 0.558 | 0.778 | 1.000 | 446 | tags=8%, list=1%, signal=8% |
| 1525 | PROTEINACEOUS\_EXTRACELLULAR\_MATRIX(GO:0005578) |  | 62 | -0.28 | -0.93 | 0.470 | 0.779 | 1.000 | 9339 | tags=40%, list=29%, signal=57% |
| 1526 | NUCLEOSOME(GO:0000786) |  | 61 | -0.25 | -0.93 | 0.682 | 0.781 | 1.000 | 7662 | tags=31%, list=24%, signal=41% |
| 1527 | DELAYED\_RECTIFIER\_POTASSIUM\_CHANNEL\_ACTIVITY(GO:0005251) |  | 23 | -0.26 | -0.92 | 0.679 | 0.784 | 1.000 | 8164 | tags=43%, list=26%, signal=58% |
| 1528 | NEGATIVE\_REGULATION\_OF\_SUPRAMOLECULAR\_FIBER\_ORGANIZATION(GO:1902904) |  | 65 | -0.22 | -0.92 | 0.550 | 0.786 | 1.000 | 2910 | tags=9%, list=9%, signal=10% |
| 1529 | CAMERA\_TYPE\_EYE\_MORPHOGENESIS(GO:0048593) |  | 167 | -0.19 | -0.92 | 0.661 | 0.785 | 1.000 | 11023 | tags=40%, list=35%, signal=60% |
| 1530 | PANCREAS\_REGENERATION(GO:1990798) |  | 19 | -0.31 | -0.92 | 0.568 | 0.787 | 1.000 | 212 | tags=5%, list=1%, signal=5% |
| 1531 | POSITIVE\_REGULATION\_OF\_TOR\_SIGNALING(GO:0032008) |  | 21 | -0.27 | -0.92 | 0.620 | 0.787 | 1.000 | 2831 | tags=10%, list=9%, signal=10% |
| 1532 | REGULATION\_OF\_STRIATED\_MUSCLE\_CELL\_DIFFERENTIATION(GO:0051153) |  | 19 | -0.30 | -0.92 | 0.477 | 0.787 | 1.000 | 2888 | tags=11%, list=9%, signal=12% |
| 1533 | MITOCHONDRIAL\_INNER\_MEMBRANE(GO:0005743) |  | 278 | -0.18 | -0.92 | 0.483 | 0.787 | 1.000 | 7159 | tags=22%, list=22%, signal=28% |
| 1534 | NEGATIVE\_REGULATION\_OF\_GROWTH(GO:0045926) |  | 53 | -0.27 | -0.92 | 0.545 | 0.788 | 1.000 | 3976 | tags=13%, list=12%, signal=15% |
| 1535 | ATP\_DEPENDENT\_MICROTUBULE\_MOTOR\_ACTIVITY(GO:1990939) |  | 30 | -0.25 | -0.92 | 0.603 | 0.787 | 1.000 | 9291 | tags=40%, list=29%, signal=56% |
| 1536 | DNA\_BINDING(GO:0003677) |  | 1961 | -0.16 | -0.92 | 0.554 | 0.789 | 1.000 | 6143 | tags=16%, list=19%, signal=18% |
| 1537 | REGULATION\_OF\_BLOOD\_PRESSURE(GO:0008217) |  | 55 | -0.25 | -0.92 | 0.601 | 0.789 | 1.000 | 6334 | tags=24%, list=20%, signal=29% |
| 1538 | RESPONSE\_TO\_MOLECULE\_OF\_BACTERIAL\_ORIGIN(GO:0002237) |  | 47 | -0.26 | -0.92 | 0.577 | 0.788 | 1.000 | 4643 | tags=19%, list=15%, signal=22% |
| 1539 | NUCLEAR\_MEMBRANE(GO:0031965) |  | 61 | -0.24 | -0.92 | 0.596 | 0.790 | 1.000 | 6762 | tags=26%, list=21%, signal=33% |
| 1540 | FATTY\_ACYL\_COA\_METABOLIC\_PROCESS(GO:0035337) |  | 20 | -0.32 | -0.92 | 0.570 | 0.791 | 1.000 | 2610 | tags=15%, list=8%, signal=16% |
| 1541 | MAINTENANCE\_OF\_PROTEIN\_LOCATION\_IN\_CELL(GO:0032507) |  | 21 | -0.28 | -0.92 | 0.663 | 0.790 | 1.000 | 1833 | tags=14%, list=6%, signal=15% |
| 1542 | TRANSCRIPTION\_REGULATOR\_ACTIVITY(GO:0140110) |  | 1576 | -0.17 | -0.92 | 0.553 | 0.792 | 1.000 | 6532 | tags=18%, list=20%, signal=21% |
| 1543 | REGULATION\_OF\_CELL\_MORPHOGENESIS\_INVOLVED\_IN\_DIFFERENTIATION(GO:0010769) |  | 133 | -0.19 | -0.92 | 0.545 | 0.791 | 1.000 | 3976 | tags=11%, list=12%, signal=13% |
| 1544 | CILIARY\_PART(GO:0044441) |  | 187 | -0.21 | -0.92 | 0.675 | 0.794 | 1.000 | 5984 | tags=21%, list=19%, signal=26% |
| 1545 | PROTEIN\_DISULFIDE\_OXIDOREDUCTASE\_ACTIVITY(GO:0015035) |  | 19 | -0.26 | -0.92 | 0.505 | 0.793 | 1.000 | 4866 | tags=21%, list=15%, signal=25% |
| 1546 | RNA\_POLYMERASE\_II\_TRANSCRIPTION\_FACTOR\_ACTIVITY\_\_SEQUENCE\_SPECIFIC\_DNA\_BINDING(GO:0000981) |  | 1186 | -0.18 | -0.92 | 0.577 | 0.793 | 1.000 | 6143 | tags=18%, list=19%, signal=21% |
| 1547 | PEROXIDASE\_ACTIVITY(GO:0004601) |  | 43 | -0.25 | -0.92 | 0.514 | 0.793 | 1.000 | 5844 | tags=23%, list=18%, signal=28% |
| 1548 | NUCLEIC\_ACID\_PHOSPHODIESTER\_BOND\_HYDROLYSIS(GO:0090305) |  | 80 | -0.25 | -0.91 | 0.549 | 0.796 | 1.000 | 8634 | tags=40%, list=27%, signal=55% |
| 1549 | RIBONUCLEOTIDE\_METABOLIC\_PROCESS(GO:0009259) |  | 269 | -0.17 | -0.91 | 0.673 | 0.796 | 1.000 | 6446 | tags=20%, list=20%, signal=24% |
| 1550 | STEROID\_METABOLIC\_PROCESS(GO:0008202) |  | 106 | -0.25 | -0.91 | 0.527 | 0.797 | 1.000 | 3622 | tags=16%, list=11%, signal=18% |
| 1551 | REGULATION\_OF\_ION\_TRANSPORT(GO:0043269) |  | 257 | -0.21 | -0.91 | 0.657 | 0.797 | 1.000 | 5938 | tags=21%, list=19%, signal=26% |
| 1552 | CELLULAR\_RESPONSE\_TO\_HYPOXIA(GO:0071456) |  | 21 | -0.28 | -0.91 | 0.597 | 0.797 | 1.000 | 7768 | tags=33%, list=24%, signal=44% |
| 1553 | REGULATION\_OF\_UBIQUITIN\_PROTEIN\_TRANSFERASE\_ACTIVITY(GO:0051438) |  | 15 | -0.29 | -0.91 | 0.501 | 0.798 | 1.000 | 659 | tags=7%, list=2%, signal=7% |
| 1554 | RECEPTOR\_COMPLEX(GO:0043235) |  | 271 | -0.18 | -0.91 | 0.549 | 0.798 | 1.000 | 5366 | tags=15%, list=17%, signal=18% |
| 1555 | NEGATIVE\_REGULATION\_OF\_G2\_M\_TRANSITION\_OF\_MITOTIC\_CELL\_CYCLE(GO:0010972) |  | 22 | -0.29 | -0.91 | 0.527 | 0.797 | 1.000 | 3207 | tags=14%, list=10%, signal=15% |
| 1556 | NEGATIVE\_REGULATION\_OF\_CELL\_CYCLE\_G2\_M\_PHASE\_TRANSITION(GO:1902750) |  | 22 | -0.29 | -0.91 | 0.527 | 0.797 | 1.000 | 3207 | tags=14%, list=10%, signal=15% |
| 1557 | PRODUCTION\_OF\_MIRNAS\_INVOLVED\_IN\_GENE\_SILENCING\_BY\_MIRNA(GO:0035196) |  | 16 | -0.24 | -0.91 | 0.458 | 0.798 | 1.000 | 2792 | tags=13%, list=9%, signal=14% |
| 1558 | MULTICELLULAR\_ORGANISM\_DEVELOPMENT(GO:0007275) |  | 4030 | -0.14 | -0.91 | 0.594 | 0.797 | 1.000 | 6332 | tags=16%, list=20%, signal=17% |
| 1559 | REGULATION\_OF\_TRANSMEMBRANE\_RECEPTOR\_PROTEIN\_SERINE\_THREONINE\_KINASE\_SIGNALING\_PATHWAY(GO:0090092) |  | 139 | -0.17 | -0.91 | 0.561 | 0.798 | 1.000 | 2028 | tags=6%, list=6%, signal=6% |
| 1560 | NEPHRON\_TUBULE\_DEVELOPMENT(GO:0072080) |  | 24 | -0.30 | -0.91 | 0.522 | 0.799 | 1.000 | 9242 | tags=42%, list=29%, signal=59% |
| 1561 | MISFOLDED\_PROTEIN\_BINDING(GO:0051787) |  | 18 | -0.31 | -0.91 | 0.460 | 0.799 | 1.000 | 2689 | tags=17%, list=8%, signal=18% |
| 1562 | DNA\_DUPLEX\_UNWINDING(GO:0032508) |  | 25 | -0.24 | -0.91 | 0.532 | 0.801 | 1.000 | 6205 | tags=20%, list=19%, signal=25% |
| 1563 | DNA\_GEOMETRIC\_CHANGE(GO:0032392) |  | 25 | -0.24 | -0.91 | 0.532 | 0.800 | 1.000 | 6205 | tags=20%, list=19%, signal=25% |
| 1564 | REGULATION\_OF\_HISTONE\_MODIFICATION(GO:0031056) |  | 30 | -0.26 | -0.91 | 0.602 | 0.800 | 1.000 | 7457 | tags=30%, list=23%, signal=39% |
| 1565 | REGULATION\_OF\_SYNAPTIC\_PLASTICITY(GO:0048167) |  | 41 | -0.28 | -0.91 | 0.567 | 0.799 | 1.000 | 8524 | tags=32%, list=27%, signal=43% |
| 1566 | FLUID\_TRANSPORT(GO:0042044) |  | 15 | -0.34 | -0.91 | 0.509 | 0.799 | 1.000 | 6303 | tags=40%, list=20%, signal=50% |
| 1567 | CALCIUM\_ION\_HOMEOSTASIS(GO:0055074) |  | 202 | -0.19 | -0.91 | 0.646 | 0.802 | 1.000 | 5936 | tags=20%, list=19%, signal=25% |
| 1568 | CALCIUM\_CHANNEL\_ACTIVITY(GO:0005262) |  | 100 | -0.21 | -0.91 | 0.590 | 0.802 | 1.000 | 5349 | tags=16%, list=17%, signal=19% |
| 1569 | REGULATION\_OF\_POLYSACCHARIDE\_BIOSYNTHETIC\_PROCESS(GO:0032885) |  | 15 | -0.33 | -0.91 | 0.516 | 0.801 | 1.000 | 10 | tags=7%, list=0%, signal=7% |
| 1570 | BEHAVIOR(GO:0007610) |  | 181 | -0.19 | -0.91 | 0.548 | 0.801 | 1.000 | 6248 | tags=17%, list=20%, signal=21% |
| 1571 | JNK\_CASCADE(GO:0007254) |  | 40 | -0.23 | -0.91 | 0.657 | 0.801 | 1.000 | 2860 | tags=10%, list=9%, signal=11% |
| 1572 | REGULATION\_OF\_RNA\_METABOLIC\_PROCESS(GO:0051252) |  | 2357 | -0.15 | -0.91 | 0.624 | 0.801 | 1.000 | 6145 | tags=15%, list=19%, signal=17% |
| 1573 | SYSTEM\_DEVELOPMENT(GO:0048731) |  | 3576 | -0.14 | -0.91 | 0.625 | 0.800 | 1.000 | 5996 | tags=14%, list=19%, signal=16% |
| 1574 | ENZYME\_LINKED\_RECEPTOR\_PROTEIN\_SIGNALING\_PATHWAY(GO:0007167) |  | 522 | -0.17 | -0.91 | 0.689 | 0.800 | 1.000 | 2562 | tags=6%, list=8%, signal=6% |
| 1575 | STEROL\_TRANSPORT(GO:0015918) |  | 31 | -0.26 | -0.91 | 0.494 | 0.801 | 1.000 | 2629 | tags=13%, list=8%, signal=14% |
| 1576 | CRANIAL\_NERVE\_DEVELOPMENT(GO:0021545) |  | 56 | -0.19 | -0.91 | 0.544 | 0.801 | 1.000 | 3736 | tags=11%, list=12%, signal=12% |
| 1577 | REGULATION\_OF\_RAS\_PROTEIN\_SIGNAL\_TRANSDUCTION(GO:0046578) |  | 105 | -0.22 | -0.90 | 0.584 | 0.802 | 1.000 | 5939 | tags=20%, list=19%, signal=24% |
| 1578 | REGULATION\_OF\_GLUCOSE\_METABOLIC\_PROCESS(GO:0010906) |  | 32 | -0.27 | -0.90 | 0.580 | 0.802 | 1.000 | 3373 | tags=16%, list=11%, signal=17% |
| 1579 | MESODERMAL\_CELL\_FATE\_SPECIFICATION(GO:0007501) |  | 15 | -0.30 | -0.90 | 0.660 | 0.803 | 1.000 | 1567 | tags=13%, list=5%, signal=14% |
| 1580 | TRANSFERASE\_ACTIVITY\_\_TRANSFERRING\_SULFUR\_CONTAINING\_GROUPS(GO:0016782) |  | 84 | -0.20 | -0.90 | 0.564 | 0.803 | 1.000 | 5477 | tags=15%, list=17%, signal=19% |
| 1581 | POSITIVE\_REGULATION\_OF\_SECRETION\_BY\_CELL(GO:1903532) |  | 23 | -0.29 | -0.90 | 0.509 | 0.805 | 1.000 | 1804 | tags=13%, list=6%, signal=14% |
| 1582 | IRON\_SULFUR\_CLUSTER\_BINDING(GO:0051536) |  | 67 | -0.21 | -0.90 | 0.558 | 0.805 | 1.000 | 5902 | tags=21%, list=19%, signal=26% |
| 1583 | METAL\_CLUSTER\_BINDING(GO:0051540) |  | 67 | -0.21 | -0.90 | 0.558 | 0.804 | 1.000 | 5902 | tags=21%, list=19%, signal=26% |
| 1584 | ACETYLGALACTOSAMINYLTRANSFERASE\_ACTIVITY(GO:0008376) |  | 16 | -0.37 | -0.90 | 0.561 | 0.804 | 1.000 | 6096 | tags=31%, list=19%, signal=39% |
| 1585 | MALE\_GAMETE\_GENERATION(GO:0048232) |  | 82 | -0.22 | -0.90 | 0.680 | 0.804 | 1.000 | 7087 | tags=20%, list=22%, signal=25% |
| 1586 | AXON\_DEVELOPMENT(GO:0061564) |  | 431 | -0.17 | -0.90 | 0.644 | 0.804 | 1.000 | 8289 | tags=24%, list=26%, signal=32% |
| 1587 | FATTY\_ACID\_DERIVATIVE\_METABOLIC\_PROCESS(GO:1901568) |  | 52 | -0.29 | -0.90 | 0.581 | 0.805 | 1.000 | 3437 | tags=17%, list=11%, signal=19% |
| 1588 | CATION\_TRANSPORTING\_ATPASE\_ACTIVITY(GO:0019829) |  | 41 | -0.22 | -0.90 | 0.515 | 0.806 | 1.000 | 9425 | tags=41%, list=30%, signal=59% |
| 1589 | ACTIVE\_ION\_TRANSMEMBRANE\_TRANSPORTER\_ACTIVITY(GO:0022853) |  | 41 | -0.22 | -0.90 | 0.515 | 0.805 | 1.000 | 9425 | tags=41%, list=30%, signal=59% |
| 1590 | ATPASE\_COUPLED\_ION\_TRANSMEMBRANE\_TRANSPORTER\_ACTIVITY(GO:0042625) |  | 41 | -0.22 | -0.90 | 0.515 | 0.805 | 1.000 | 9425 | tags=41%, list=30%, signal=59% |
| 1591 | CELL\_DIFFERENTIATION(GO:0030154) |  | 2346 | -0.14 | -0.90 | 0.638 | 0.804 | 1.000 | 6337 | tags=16%, list=20%, signal=19% |
| 1592 | AMMONIUM\_TRANSPORT(GO:0015696) |  | 51 | -0.26 | -0.90 | 0.622 | 0.804 | 1.000 | 9561 | tags=39%, list=30%, signal=56% |
| 1593 | PROTEIN\_DEACETYLATION(GO:0006476) |  | 40 | -0.21 | -0.90 | 0.567 | 0.804 | 1.000 | 2931 | tags=10%, list=9%, signal=11% |
| 1594 | RECEPTOR\_LOCALIZATION\_TO\_SYNAPSE(GO:0097120) |  | 39 | -0.24 | -0.90 | 0.548 | 0.803 | 1.000 | 9031 | tags=38%, list=28%, signal=54% |
| 1595 | ACTININ\_BINDING(GO:0042805) |  | 16 | -0.31 | -0.90 | 0.548 | 0.804 | 1.000 | 8725 | tags=44%, list=27%, signal=60% |
| 1596 | CORTICAL\_ACTIN\_CYTOSKELETON\_ORGANIZATION(GO:0030866) |  | 27 | -0.26 | -0.90 | 0.493 | 0.804 | 1.000 | 9982 | tags=37%, list=31%, signal=54% |
| 1597 | CILIARY\_TRANSITION\_ZONE(GO:0035869) |  | 30 | -0.24 | -0.90 | 0.656 | 0.808 | 1.000 | 7059 | tags=17%, list=22%, signal=21% |
| 1598 | TRANSFERASE\_ACTIVITY\_\_TRANSFERRING\_HEXOSYL\_GROUPS(GO:0016758) |  | 222 | -0.21 | -0.90 | 0.612 | 0.808 | 1.000 | 6132 | tags=22%, list=19%, signal=27% |
| 1599 | SCHWANN\_CELL\_DIFFERENTIATION(GO:0014037) |  | 24 | -0.26 | -0.90 | 0.582 | 0.811 | 1.000 | 354 | tags=4%, list=1%, signal=4% |
| 1600 | PROTEIN\_DEACYLATION(GO:0035601) |  | 52 | -0.20 | -0.90 | 0.645 | 0.810 | 1.000 | 2931 | tags=10%, list=9%, signal=11% |
| 1601 | CELL\_VOLUME\_HOMEOSTASIS(GO:0006884) |  | 17 | -0.34 | -0.89 | 0.531 | 0.811 | 1.000 | 9907 | tags=47%, list=31%, signal=68% |
| 1602 | NEURON\_RECOGNITION(GO:0008038) |  | 39 | -0.21 | -0.89 | 0.568 | 0.811 | 1.000 | 1419 | tags=5%, list=4%, signal=5% |
| 1603 | INWARD\_RECTIFIER\_POTASSIUM\_CHANNEL\_ACTIVITY(GO:0005242) |  | 26 | -0.30 | -0.89 | 0.687 | 0.813 | 1.000 | 5160 | tags=23%, list=16%, signal=28% |
| 1604 | CARBOHYDRATE\_BINDING(GO:0030246) |  | 156 | -0.21 | -0.89 | 0.729 | 0.813 | 1.000 | 4821 | tags=15%, list=15%, signal=18% |
| 1605 | REGULATION\_OF\_ANATOMICAL\_STRUCTURE\_SIZE(GO:0090066) |  | 296 | -0.16 | -0.89 | 0.579 | 0.813 | 1.000 | 5213 | tags=13%, list=16%, signal=15% |
| 1606 | AXIS\_ELONGATION(GO:0003401) |  | 48 | -0.17 | -0.89 | 0.566 | 0.814 | 1.000 | 4289 | tags=8%, list=13%, signal=10% |
| 1607 | LEUKOCYTE\_HOMEOSTASIS(GO:0001776) |  | 15 | -0.27 | -0.89 | 0.628 | 0.813 | 1.000 | 4402 | tags=27%, list=14%, signal=31% |
| 1608 | MITOTIC\_G2\_M\_TRANSITION\_CHECKPOINT(GO:0044818) |  | 15 | -0.33 | -0.89 | 0.545 | 0.815 | 1.000 | 3207 | tags=20%, list=10%, signal=22% |
| 1609 | NEUTRAL\_LIPID\_CATABOLIC\_PROCESS(GO:0046461) |  | 18 | -0.29 | -0.89 | 0.598 | 0.815 | 1.000 | 5968 | tags=22%, list=19%, signal=27% |
| 1610 | ACYLGLYCEROL\_CATABOLIC\_PROCESS(GO:0046464) |  | 18 | -0.29 | -0.89 | 0.598 | 0.815 | 1.000 | 5968 | tags=22%, list=19%, signal=27% |
| 1611 | SULFUR\_COMPOUND\_BINDING(GO:1901681) |  | 114 | -0.18 | -0.89 | 0.609 | 0.814 | 1.000 | 4471 | tags=11%, list=14%, signal=13% |
| 1612 | CLEAVAGE\_INVOLVED\_IN\_RRNA\_PROCESSING(GO:0000469) |  | 22 | -0.32 | -0.89 | 0.588 | 0.814 | 1.000 | 8956 | tags=59%, list=28%, signal=82% |
| 1613 | ZINC\_ION\_TRANSPORT(GO:0006829) |  | 23 | -0.29 | -0.89 | 0.622 | 0.814 | 1.000 | 5182 | tags=17%, list=16%, signal=21% |
| 1614 | CELL\_MORPHOGENESIS\_INVOLVED\_IN\_NEURON\_DIFFERENTIATION(GO:0048667) |  | 442 | -0.17 | -0.89 | 0.661 | 0.815 | 1.000 | 9060 | tags=27%, list=28%, signal=37% |
| 1615 | EXTRACELLULAR\_MATRIX\_COMPONENT(GO:0044420) |  | 58 | -0.26 | -0.89 | 0.557 | 0.815 | 1.000 | 9339 | tags=38%, list=29%, signal=54% |
| 1616 | MITOCHONDRIAL\_MEMBRANE(GO:0031966) |  | 404 | -0.17 | -0.89 | 0.529 | 0.816 | 1.000 | 6636 | tags=19%, list=21%, signal=24% |
| 1617 | REGULATION\_OF\_CELLULAR\_COMPONENT\_SIZE(GO:0032535) |  | 243 | -0.16 | -0.89 | 0.568 | 0.815 | 1.000 | 5213 | tags=12%, list=16%, signal=15% |
| 1618 | CHOLESTEROL\_TRANSPORT(GO:0030301) |  | 30 | -0.26 | -0.89 | 0.512 | 0.815 | 1.000 | 2629 | tags=13%, list=8%, signal=15% |
| 1619 | ANTIOXIDANT\_ACTIVITY(GO:0016209) |  | 63 | -0.24 | -0.89 | 0.610 | 0.815 | 1.000 | 5844 | tags=24%, list=18%, signal=29% |
| 1620 | CYTOCHROME\_COMPLEX(GO:0070069) |  | 26 | -0.28 | -0.89 | 0.499 | 0.815 | 1.000 | 11201 | tags=62%, list=35%, signal=95% |
| 1621 | PRONEPHRIC\_DUCT\_DEVELOPMENT(GO:0039022) |  | 18 | -0.30 | -0.89 | 0.549 | 0.816 | 1.000 | 10981 | tags=44%, list=34%, signal=68% |
| 1622 | RNA\_POLYMERASE\_II\_REGULATORY\_REGION\_SEQUENCE\_SPECIFIC\_DNA\_BINDING(GO:0000977) |  | 1176 | -0.17 | -0.89 | 0.648 | 0.815 | 1.000 | 6143 | tags=17%, list=19%, signal=20% |
| 1623 | RNA\_POLYMERASE\_II\_REGULATORY\_REGION\_DNA\_BINDING(GO:0001012) |  | 1176 | -0.17 | -0.89 | 0.648 | 0.815 | 1.000 | 6143 | tags=17%, list=19%, signal=20% |
| 1624 | POSITIVE\_REGULATION\_OF\_STRESS\_ACTIVATED\_MAPK\_CASCADE(GO:0032874) |  | 23 | -0.29 | -0.89 | 0.614 | 0.816 | 1.000 | 2715 | tags=9%, list=9%, signal=9% |
| 1625 | POSITIVE\_REGULATION\_OF\_STRESS\_ACTIVATED\_PROTEIN\_KINASE\_SIGNALING\_CASCADE(GO:0070304) |  | 23 | -0.29 | -0.89 | 0.614 | 0.815 | 1.000 | 2715 | tags=9%, list=9%, signal=9% |
| 1626 | CELL\_MORPHOGENESIS\_INVOLVED\_IN\_DIFFERENTIATION(GO:0000904) |  | 519 | -0.16 | -0.89 | 0.575 | 0.815 | 1.000 | 6303 | tags=16%, list=20%, signal=20% |
| 1627 | REGULATION\_OF\_HEART\_RATE(GO:0002027) |  | 18 | -0.27 | -0.89 | 0.567 | 0.817 | 1.000 | 3791 | tags=17%, list=12%, signal=19% |
| 1628 | NUCLEAR\_TRANSCRIPTION\_FACTOR\_COMPLEX(GO:0044798) |  | 111 | -0.17 | -0.89 | 0.587 | 0.817 | 1.000 | 6949 | tags=21%, list=22%, signal=26% |
| 1629 | GROWTH\_CONE(GO:0030426) |  | 32 | -0.24 | -0.89 | 0.569 | 0.816 | 1.000 | 9411 | tags=38%, list=30%, signal=53% |
| 1630 | WATER\_SOLUBLE\_VITAMIN\_METABOLIC\_PROCESS(GO:0006767) |  | 19 | -0.31 | -0.89 | 0.637 | 0.816 | 1.000 | 6931 | tags=42%, list=22%, signal=54% |
| 1631 | NEURON\_DEVELOPMENT(GO:0048666) |  | 741 | -0.16 | -0.89 | 0.590 | 0.817 | 1.000 | 6888 | tags=18%, list=22%, signal=22% |
| 1632 | POSITIVE\_REGULATION\_OF\_CELL\_CYCLE(GO:0045787) |  | 49 | -0.18 | -0.88 | 0.616 | 0.818 | 1.000 | 6924 | tags=20%, list=22%, signal=26% |
| 1633 | POSTSYNAPTIC\_DENSITY\_MEMBRANE(GO:0098839) |  | 32 | -0.29 | -0.88 | 0.470 | 0.818 | 1.000 | 9594 | tags=38%, list=30%, signal=54% |
| 1634 | PROTEIN\_TARGETING\_TO\_MITOCHONDRION(GO:0006626) |  | 38 | -0.26 | -0.88 | 0.548 | 0.818 | 1.000 | 5267 | tags=16%, list=17%, signal=19% |
| 1635 | MITOCHONDRIAL\_ENVELOPE(GO:0005740) |  | 426 | -0.17 | -0.88 | 0.485 | 0.818 | 1.000 | 6488 | tags=19%, list=20%, signal=24% |
| 1636 | PROTON\_TRANSPORTING\_TWO\_SECTOR\_ATPASE\_COMPLEX(GO:0016469) |  | 54 | -0.24 | -0.88 | 0.565 | 0.820 | 1.000 | 9801 | tags=44%, list=31%, signal=64% |
| 1637 | PERIPHERAL\_NERVOUS\_SYSTEM\_AXON\_ENSHEATHMENT(GO:0032292) |  | 19 | -0.24 | -0.88 | 0.588 | 0.822 | 1.000 | 689 | tags=5%, list=2%, signal=5% |
| 1638 | EPITHELIAL\_TUBE\_FORMATION(GO:0072175) |  | 64 | -0.21 | -0.88 | 0.631 | 0.825 | 1.000 | 10821 | tags=38%, list=34%, signal=57% |
| 1639 | DSRNA\_FRAGMENTATION(GO:0031050) |  | 18 | -0.23 | -0.88 | 0.664 | 0.825 | 1.000 | 2792 | tags=11%, list=9%, signal=12% |
| 1640 | PRODUCTION\_OF\_SMALL\_RNA\_INVOLVED\_IN\_GENE\_SILENCING\_BY\_RNA(GO:0070918) |  | 18 | -0.23 | -0.88 | 0.664 | 0.824 | 1.000 | 2792 | tags=11%, list=9%, signal=12% |
| 1641 | PHAGOPHORE\_ASSEMBLY\_SITE(GO:0000407) |  | 18 | -0.27 | -0.88 | 0.624 | 0.824 | 1.000 | 23309 | tags=100%, list=73%, signal=371% |
| 1642 | EMBRYONIC\_PATTERN\_SPECIFICATION(GO:0009880) |  | 53 | -0.22 | -0.88 | 0.622 | 0.824 | 1.000 | 7204 | tags=23%, list=23%, signal=29% |
| 1643 | CELLULAR\_RESPONSE\_TO\_AMINO\_ACID\_STIMULUS(GO:0071230) |  | 21 | -0.29 | -0.88 | 0.588 | 0.824 | 1.000 | 8994 | tags=38%, list=28%, signal=53% |
| 1644 | ENZYME\_ACTIVATOR\_ACTIVITY(GO:0008047) |  | 310 | -0.16 | -0.88 | 0.625 | 0.825 | 1.000 | 4365 | tags=11%, list=14%, signal=12% |
| 1645 | REGULATION\_OF\_NUCLEAR\_DIVISION(GO:0051783) |  | 61 | -0.21 | -0.88 | 0.594 | 0.825 | 1.000 | 2255 | tags=7%, list=7%, signal=7% |
| 1646 | MICROTUBULE\_CYTOSKELETON\_ORGANIZATION(GO:0000226) |  | 347 | -0.16 | -0.88 | 0.766 | 0.825 | 1.000 | 5901 | tags=14%, list=19%, signal=16% |
| 1647 | RESPONSE\_TO\_ACID\_CHEMICAL(GO:0001101) |  | 57 | -0.22 | -0.88 | 0.626 | 0.826 | 1.000 | 7768 | tags=30%, list=24%, signal=39% |
| 1648 | AXONOGENESIS(GO:0007409) |  | 402 | -0.17 | -0.88 | 0.692 | 0.826 | 1.000 | 8406 | tags=24%, list=26%, signal=33% |
| 1649 | GUANYL\_NUCLEOTIDE\_EXCHANGE\_FACTOR\_ACTIVITY(GO:0005085) |  | 168 | -0.19 | -0.88 | 0.572 | 0.825 | 1.000 | 4191 | tags=11%, list=13%, signal=12% |
| 1650 | NEURON\_PROJECTION\_DEVELOPMENT(GO:0031175) |  | 587 | -0.17 | -0.88 | 0.595 | 0.825 | 1.000 | 6888 | tags=18%, list=22%, signal=22% |
| 1651 | MONOVALENT\_INORGANIC\_CATION\_TRANSPORT(GO:0015672) |  | 368 | -0.19 | -0.88 | 0.763 | 0.825 | 1.000 | 6833 | tags=21%, list=21%, signal=27% |
| 1652 | SEQUENCE\_SPECIFIC\_DOUBLE\_STRANDED\_DNA\_BINDING(GO:1990837) |  | 1251 | -0.17 | -0.88 | 0.680 | 0.825 | 1.000 | 6143 | tags=17%, list=19%, signal=20% |
| 1653 | CHONDROCYTE\_DIFFERENTIATION(GO:0002062) |  | 30 | -0.22 | -0.88 | 0.702 | 0.825 | 1.000 | 259 | tags=3%, list=1%, signal=3% |
| 1654 | OTIC\_VESICLE\_DEVELOPMENT(GO:0071599) |  | 49 | -0.22 | -0.88 | 0.595 | 0.826 | 1.000 | 8866 | tags=31%, list=28%, signal=42% |
| 1655 | EARLY\_ENDOSOME\_MEMBRANE(GO:0031901) |  | 47 | -0.18 | -0.87 | 0.678 | 0.826 | 1.000 | 9694 | tags=30%, list=30%, signal=43% |
| 1656 | PSEUDOURIDINE\_SYNTHESIS(GO:0001522) |  | 16 | -0.29 | -0.87 | 0.660 | 0.826 | 1.000 | 6895 | tags=38%, list=22%, signal=48% |
| 1657 | ACETYLGLUCOSAMINYLTRANSFERASE\_ACTIVITY(GO:0008375) |  | 44 | -0.22 | -0.87 | 0.647 | 0.825 | 1.000 | 3739 | tags=14%, list=12%, signal=15% |
| 1658 | EXTRINSIC\_COMPONENT\_OF\_PLASMA\_MEMBRANE(GO:0019897) |  | 121 | -0.19 | -0.87 | 0.522 | 0.826 | 1.000 | 409 | tags=2%, list=1%, signal=3% |
| 1659 | NEPHRON\_DEVELOPMENT(GO:0072006) |  | 85 | -0.19 | -0.87 | 0.702 | 0.826 | 1.000 | 9640 | tags=28%, list=30%, signal=40% |
| 1660 | REGULATION\_OF\_CARBOHYDRATE\_METABOLIC\_PROCESS(GO:0006109) |  | 37 | -0.24 | -0.87 | 0.633 | 0.825 | 1.000 | 3959 | tags=16%, list=12%, signal=18% |
| 1661 | SODIUM\_ION\_TRANSPORT(GO:0006814) |  | 157 | -0.21 | -0.87 | 0.557 | 0.825 | 1.000 | 3781 | tags=11%, list=12%, signal=13% |
| 1662 | DORSAL\_VENTRAL\_PATTERN\_FORMATION(GO:0009953) |  | 143 | -0.16 | -0.87 | 0.652 | 0.827 | 1.000 | 5549 | tags=13%, list=17%, signal=16% |
| 1663 | RESPONSE\_TO\_LIPOPOLYSACCHARIDE(GO:0032496) |  | 43 | -0.25 | -0.87 | 0.699 | 0.826 | 1.000 | 4643 | tags=19%, list=15%, signal=22% |
| 1664 | REGULATORY\_REGION\_NUCLEIC\_ACID\_BINDING(GO:0001067) |  | 1224 | -0.17 | -0.87 | 0.684 | 0.828 | 1.000 | 6143 | tags=17%, list=19%, signal=20% |
| 1665 | TRANSCRIPTION\_REGULATORY\_REGION\_DNA\_BINDING(GO:0044212) |  | 1224 | -0.17 | -0.87 | 0.684 | 0.827 | 1.000 | 6143 | tags=17%, list=19%, signal=20% |
| 1666 | TRANSCRIPTION\_REGULATORY\_REGION\_SEQUENCE\_SPECIFIC\_DNA\_BINDING(GO:0000976) |  | 1223 | -0.17 | -0.87 | 0.684 | 0.827 | 1.000 | 6143 | tags=17%, list=19%, signal=20% |
| 1667 | REGULATION\_OF\_NEUROTRANSMITTER\_TRANSPORT(GO:0051588) |  | 29 | -0.26 | -0.87 | 0.599 | 0.828 | 1.000 | 8796 | tags=41%, list=28%, signal=57% |
| 1668 | ANATOMICAL\_STRUCTURE\_MATURATION(GO:0071695) |  | 64 | -0.19 | -0.87 | 0.667 | 0.828 | 1.000 | 12003 | tags=50%, list=38%, signal=80% |
| 1669 | REGULATION\_OF\_POSTSYNAPTIC\_MEMBRANE\_NEUROTRANSMITTER\_RECEPTOR\_LEVELS(GO:0099072) |  | 29 | -0.26 | -0.87 | 0.527 | 0.828 | 1.000 | 9031 | tags=38%, list=28%, signal=53% |
| 1670 | REGULATION\_OF\_SYSTEM\_PROCESS(GO:0044057) |  | 127 | -0.18 | -0.87 | 0.723 | 0.830 | 1.000 | 3791 | tags=13%, list=12%, signal=14% |
| 1671 | PLASMA\_MEMBRANE\_REGION(GO:0098590) |  | 467 | -0.19 | -0.87 | 0.619 | 0.831 | 1.000 | 4370 | tags=13%, list=14%, signal=15% |
| 1672 | REGULATION\_OF\_CYTOSOLIC\_CALCIUM\_ION\_CONCENTRATION(GO:0051480) |  | 142 | -0.20 | -0.87 | 0.795 | 0.831 | 1.000 | 6506 | tags=23%, list=20%, signal=29% |
| 1673 | CARBON\_OXYGEN\_LYASE\_ACTIVITY(GO:0016835) |  | 65 | -0.23 | -0.87 | 0.555 | 0.831 | 1.000 | 4147 | tags=18%, list=13%, signal=21% |
| 1674 | PHOSPHATIDYLINOSITOL\_MEDIATED\_SIGNALING(GO:0048015) |  | 50 | -0.23 | -0.87 | 0.529 | 0.831 | 1.000 | 9541 | tags=40%, list=30%, signal=57% |
| 1675 | PROTEIN\_CONTAINING\_COMPLEX\_SCAFFOLD\_ACTIVITY(GO:0032947) |  | 22 | -0.26 | -0.87 | 0.611 | 0.831 | 1.000 | 2860 | tags=9%, list=9%, signal=10% |
| 1676 | CALCIUM\_ION\_IMPORT(GO:0070509) |  | 40 | -0.25 | -0.87 | 0.502 | 0.831 | 1.000 | 9633 | tags=45%, list=30%, signal=64% |
| 1677 | REGULATION\_OF\_BLOOD\_VESSEL\_ENDOTHELIAL\_CELL\_MIGRATION(GO:0043535) |  | 17 | -0.28 | -0.87 | 0.553 | 0.832 | 1.000 | 6528 | tags=29%, list=20%, signal=37% |
| 1678 | CILIUM\_ASSEMBLY(GO:0060271) |  | 275 | -0.16 | -0.87 | 0.692 | 0.831 | 1.000 | 5996 | tags=15%, list=19%, signal=19% |
| 1679 | NON\_MOTILE\_CILIUM\_ASSEMBLY(GO:1905515) |  | 23 | -0.25 | -0.87 | 0.575 | 0.831 | 1.000 | 7059 | tags=30%, list=22%, signal=39% |
| 1680 | IMMUNE\_RESPONSE\_ACTIVATING\_CELL\_SURFACE\_RECEPTOR\_SIGNALING\_PATHWAY(GO:0002429) |  | 48 | -0.25 | -0.87 | 0.638 | 0.831 | 1.000 | 2821 | tags=15%, list=9%, signal=16% |
| 1681 | PROTEIN\_PEPTIDYL\_PROLYL\_ISOMERIZATION(GO:0000413) |  | 23 | -0.24 | -0.87 | 0.595 | 0.831 | 1.000 | 9244 | tags=26%, list=29%, signal=37% |
| 1682 | DISTAL\_AXON(GO:0150034) |  | 60 | -0.25 | -0.87 | 0.671 | 0.831 | 1.000 | 9964 | tags=42%, list=31%, signal=60% |
| 1683 | POTASSIUM:CHLORIDE\_SYMPORTER\_ACTIVITY(GO:0015379) |  | 15 | -0.35 | -0.87 | 0.667 | 0.830 | 1.000 | 9907 | tags=53%, list=31%, signal=77% |
| 1684 | CATION:CHLORIDE\_SYMPORTER\_ACTIVITY(GO:0015377) |  | 15 | -0.35 | -0.87 | 0.667 | 0.830 | 1.000 | 9907 | tags=53%, list=31%, signal=77% |
| 1685 | SYNAPTIC\_TRANSMISSION\_\_GLUTAMATERGIC(GO:0035249) |  | 48 | -0.28 | -0.87 | 0.676 | 0.829 | 1.000 | 9325 | tags=40%, list=29%, signal=56% |
| 1686 | NEPHRON\_EPITHELIUM\_DEVELOPMENT(GO:0072009) |  | 53 | -0.21 | -0.86 | 0.610 | 0.831 | 1.000 | 8725 | tags=30%, list=27%, signal=41% |
| 1687 | REGULATION\_OF\_VESICLE\_MEDIATED\_TRANSPORT(GO:0060627) |  | 139 | -0.16 | -0.86 | 0.812 | 0.831 | 1.000 | 4549 | tags=14%, list=14%, signal=16% |
| 1688 | REGULATION\_OF\_SMALL\_GTPASE\_MEDIATED\_SIGNAL\_TRANSDUCTION(GO:0051056) |  | 124 | -0.20 | -0.86 | 0.659 | 0.831 | 1.000 | 5939 | tags=18%, list=19%, signal=22% |
| 1689 | NEPHRIC\_DUCT\_DEVELOPMENT(GO:0072176) |  | 19 | -0.29 | -0.86 | 0.576 | 0.832 | 1.000 | 12385 | tags=53%, list=39%, signal=86% |
| 1690 | PHOSPHATIDATE\_PHOSPHATASE\_ACTIVITY(GO:0008195) |  | 19 | -0.28 | -0.86 | 0.707 | 0.833 | 1.000 | 4001 | tags=21%, list=13%, signal=24% |
| 1691 | PROTEIN\_DIMERIZATION\_ACTIVITY(GO:0046983) |  | 368 | -0.15 | -0.86 | 0.752 | 0.833 | 1.000 | 7218 | tags=19%, list=23%, signal=24% |
| 1692 | REGULATION\_OF\_CARBOHYDRATE\_BIOSYNTHETIC\_PROCESS(GO:0043255) |  | 23 | -0.27 | -0.86 | 0.688 | 0.833 | 1.000 | 3959 | tags=17%, list=12%, signal=20% |
| 1693 | DOUBLE\_STRANDED\_DNA\_BINDING(GO:0003690) |  | 1311 | -0.16 | -0.86 | 0.713 | 0.834 | 1.000 | 6143 | tags=16%, list=19%, signal=19% |
| 1694 | CTP\_BIOSYNTHETIC\_PROCESS(GO:0006241) |  | 17 | -0.29 | -0.86 | 0.582 | 0.834 | 1.000 | 8837 | tags=47%, list=28%, signal=65% |
| 1695 | CTP\_METABOLIC\_PROCESS(GO:0046036) |  | 17 | -0.29 | -0.86 | 0.582 | 0.834 | 1.000 | 8837 | tags=47%, list=28%, signal=65% |
| 1696 | MESODERMAL\_CELL\_FATE\_COMMITMENT(GO:0001710) |  | 16 | -0.30 | -0.86 | 0.710 | 0.834 | 1.000 | 1567 | tags=13%, list=5%, signal=13% |
| 1697 | CONVERGENT\_EXTENSION\_INVOLVED\_IN\_AXIS\_ELONGATION(GO:0060028) |  | 35 | -0.20 | -0.86 | 0.637 | 0.835 | 1.000 | 1834 | tags=6%, list=6%, signal=6% |
| 1698 | POLYMERIC\_CYTOSKELETAL\_FIBER(GO:0099513) |  | 363 | -0.15 | -0.86 | 0.737 | 0.835 | 1.000 | 6380 | tags=17%, list=20%, signal=21% |
| 1699 | REGULATION\_OF\_CELL\_FATE\_COMMITMENT(GO:0010453) |  | 25 | -0.28 | -0.86 | 0.687 | 0.835 | 1.000 | 1206 | tags=8%, list=4%, signal=8% |
| 1700 | INTEGRAL\_COMPONENT\_OF\_ORGANELLE\_MEMBRANE(GO:0031301) |  | 147 | -0.18 | -0.86 | 0.726 | 0.837 | 1.000 | 6611 | tags=20%, list=21%, signal=25% |
| 1701 | OXIDOREDUCTASE\_ACTIVITY\_\_ACTING\_ON\_NAD(P)H(GO:0016651) |  | 57 | -0.17 | -0.86 | 0.579 | 0.836 | 1.000 | 1817 | tags=5%, list=6%, signal=6% |
| 1702 | MITOCHONDRION\_LOCALIZATION(GO:0051646) |  | 16 | -0.23 | -0.86 | 0.467 | 0.837 | 1.000 | 3518 | tags=13%, list=11%, signal=14% |
| 1703 | REGULATION\_OF\_INTRACELLULAR\_PROTEIN\_TRANSPORT(GO:0033157) |  | 19 | -0.27 | -0.86 | 0.685 | 0.837 | 1.000 | 1585 | tags=11%, list=5%, signal=11% |
| 1704 | ICOSANOID\_METABOLIC\_PROCESS(GO:0006690) |  | 28 | -0.31 | -0.86 | 0.597 | 0.837 | 1.000 | 3437 | tags=21%, list=11%, signal=24% |
| 1705 | POSITIVE\_REGULATION\_OF\_SECRETION(GO:0051047) |  | 25 | -0.28 | -0.86 | 0.696 | 0.836 | 1.000 | 1804 | tags=12%, list=6%, signal=13% |
| 1706 | MYOSIN\_BINDING(GO:0017022) |  | 20 | -0.29 | -0.86 | 0.764 | 0.838 | 1.000 | 10011 | tags=45%, list=31%, signal=66% |
| 1707 | FORMATION\_OF\_CYTOPLASMIC\_TRANSLATION\_INITIATION\_COMPLEX(GO:0001732) |  | 17 | -0.25 | -0.86 | 0.775 | 0.838 | 1.000 | 10745 | tags=41%, list=34%, signal=62% |
| 1708 | SODIUM\_CHANNEL\_REGULATOR\_ACTIVITY(GO:0017080) |  | 22 | -0.28 | -0.85 | 0.721 | 0.839 | 1.000 | 6295 | tags=36%, list=20%, signal=45% |
| 1709 | SYNCYTIUM\_FORMATION\_BY\_PLASMA\_MEMBRANE\_FUSION(GO:0000768) |  | 23 | -0.22 | -0.85 | 0.616 | 0.839 | 1.000 | 4213 | tags=13%, list=13%, signal=15% |
| 1710 | SYNCYTIUM\_FORMATION(GO:0006949) |  | 23 | -0.22 | -0.85 | 0.616 | 0.838 | 1.000 | 4213 | tags=13%, list=13%, signal=15% |
| 1711 | TISSUE\_MORPHOGENESIS(GO:0048729) |  | 551 | -0.15 | -0.85 | 0.678 | 0.839 | 1.000 | 5018 | tags=12%, list=16%, signal=14% |
| 1712 | G\_PROTEIN\_COUPLED\_GLUTAMATE\_RECEPTOR\_SIGNALING\_PATHWAY(GO:0007216) |  | 15 | -0.37 | -0.85 | 0.566 | 0.838 | 1.000 | 5966 | tags=33%, list=19%, signal=41% |
| 1713 | INORGANIC\_ION\_TRANSMEMBRANE\_TRANSPORT(GO:0098660) |  | 522 | -0.17 | -0.85 | 0.785 | 0.841 | 1.000 | 5996 | tags=18%, list=19%, signal=21% |
| 1714 | CALCIUM\_CHANNEL\_COMPLEX(GO:0034704) |  | 47 | -0.22 | -0.85 | 0.779 | 0.841 | 1.000 | 5032 | tags=17%, list=16%, signal=20% |
| 1715 | CHEMOTAXIS(GO:0006935) |  | 384 | -0.16 | -0.85 | 0.814 | 0.843 | 1.000 | 4402 | tags=10%, list=14%, signal=12% |
| 1716 | REGULATION\_OF\_NUCLEOTIDE\_METABOLIC\_PROCESS(GO:0006140) |  | 26 | -0.24 | -0.85 | 0.783 | 0.842 | 1.000 | 6196 | tags=31%, list=19%, signal=38% |
| 1717 | REGULATION\_OF\_PURINE\_NUCLEOTIDE\_METABOLIC\_PROCESS(GO:1900542) |  | 26 | -0.24 | -0.85 | 0.783 | 0.842 | 1.000 | 6196 | tags=31%, list=19%, signal=38% |
| 1718 | EUKARYOTIC\_43S\_PREINITIATION\_COMPLEX(GO:0016282) |  | 17 | -0.26 | -0.85 | 0.685 | 0.842 | 1.000 | 10745 | tags=41%, list=34%, signal=62% |
| 1719 | BMP\_SIGNALING\_PATHWAY(GO:0030509) |  | 127 | -0.17 | -0.85 | 0.664 | 0.844 | 1.000 | 8477 | tags=24%, list=27%, signal=32% |
| 1720 | RESPONSE\_TO\_BMP(GO:0071772) |  | 127 | -0.17 | -0.85 | 0.664 | 0.843 | 1.000 | 8477 | tags=24%, list=27%, signal=32% |
| 1721 | CELLULAR\_RESPONSE\_TO\_BMP\_STIMULUS(GO:0071773) |  | 127 | -0.17 | -0.85 | 0.664 | 0.843 | 1.000 | 8477 | tags=24%, list=27%, signal=32% |
| 1722 | RESPONSE\_TO\_CALCIUM\_ION(GO:0051592) |  | 37 | -0.29 | -0.85 | 0.589 | 0.843 | 1.000 | 8725 | tags=38%, list=27%, signal=52% |
| 1723 | EMBRYONIC\_ORGAN\_MORPHOGENESIS(GO:0048562) |  | 412 | -0.15 | -0.85 | 0.624 | 0.843 | 1.000 | 8983 | tags=25%, list=28%, signal=34% |
| 1724 | NUCLEOBASE\_CONTAINING\_COMPOUND\_TRANSMEMBRANE\_TRANSPORTER\_ACTIVITY(GO:0015932) |  | 39 | -0.19 | -0.85 | 0.805 | 0.843 | 1.000 | 3772 | tags=10%, list=12%, signal=12% |
| 1725 | DENDRITIC\_SPINE(GO:0043197) |  | 48 | -0.22 | -0.85 | 0.708 | 0.845 | 1.000 | 8190 | tags=33%, list=26%, signal=45% |
| 1726 | NEURON\_SPINE(GO:0044309) |  | 48 | -0.22 | -0.85 | 0.708 | 0.844 | 1.000 | 8190 | tags=33%, list=26%, signal=45% |
| 1727 | T\_CELL\_ACTIVATION\_INVOLVED\_IN\_IMMUNE\_RESPONSE(GO:0002286) |  | 17 | -0.28 | -0.85 | 0.657 | 0.846 | 1.000 | 1287 | tags=12%, list=4%, signal=12% |
| 1728 | EXPORT\_ACROSS\_PLASMA\_MEMBRANE(GO:0140115) |  | 21 | -0.24 | -0.85 | 0.657 | 0.845 | 1.000 | 7720 | tags=29%, list=24%, signal=38% |
| 1729 | OTIC\_VESICLE\_MORPHOGENESIS(GO:0071600) |  | 32 | -0.23 | -0.85 | 0.609 | 0.845 | 1.000 | 8866 | tags=34%, list=28%, signal=48% |
| 1730 | NAD+\_ADP\_RIBOSYLTRANSFERASE\_ACTIVITY(GO:0003950) |  | 27 | -0.23 | -0.85 | 0.788 | 0.845 | 1.000 | 4387 | tags=15%, list=14%, signal=17% |
| 1731 | TUMOR\_NECROSIS\_FACTOR\_RECEPTOR\_BINDING(GO:0005164) |  | 24 | -0.29 | -0.85 | 0.609 | 0.845 | 1.000 | 1364 | tags=8%, list=4%, signal=9% |
| 1732 | ADRENERGIC\_RECEPTOR\_ACTIVITY(GO:0004935) |  | 15 | -0.30 | -0.85 | 0.662 | 0.845 | 1.000 | 6334 | tags=27%, list=20%, signal=33% |
| 1733 | PYRIMIDINE\_RIBONUCLEOSIDE\_TRIPHOSPHATE\_METABOLIC\_PROCESS(GO:0009208) |  | 18 | -0.27 | -0.84 | 0.576 | 0.846 | 1.000 | 8837 | tags=44%, list=28%, signal=61% |
| 1734 | PYRIMIDINE\_RIBONUCLEOSIDE\_TRIPHOSPHATE\_BIOSYNTHETIC\_PROCESS(GO:0009209) |  | 18 | -0.27 | -0.84 | 0.576 | 0.846 | 1.000 | 8837 | tags=44%, list=28%, signal=61% |
| 1735 | STEROL\_BIOSYNTHETIC\_PROCESS(GO:0016126) |  | 28 | -0.25 | -0.84 | 0.696 | 0.847 | 1.000 | 2692 | tags=14%, list=8%, signal=16% |
| 1736 | REGULATION\_OF\_G2\_M\_TRANSITION\_OF\_MITOTIC\_CELL\_CYCLE(GO:0010389) |  | 29 | -0.24 | -0.84 | 0.646 | 0.847 | 1.000 | 3517 | tags=14%, list=11%, signal=15% |
| 1737 | POTASSIUM\_ION\_HOMEOSTASIS(GO:0055075) |  | 30 | -0.27 | -0.84 | 0.624 | 0.850 | 1.000 | 7949 | tags=30%, list=25%, signal=40% |
| 1738 | SITE\_OF\_POLARIZED\_GROWTH(GO:0030427) |  | 41 | -0.20 | -0.84 | 0.582 | 0.852 | 1.000 | 9411 | tags=34%, list=30%, signal=48% |
| 1739 | CELL\_PROJECTION(GO:0042995) |  | 980 | -0.17 | -0.84 | 0.758 | 0.852 | 1.000 | 6086 | tags=18%, list=19%, signal=22% |
| 1740 | VOLTAGE\_GATED\_POTASSIUM\_CHANNEL\_COMPLEX(GO:0008076) |  | 44 | -0.22 | -0.84 | 0.851 | 0.852 | 1.000 | 4468 | tags=23%, list=14%, signal=26% |
| 1741 | MEMBRANE\_INVAGINATION(GO:0010324) |  | 36 | -0.25 | -0.84 | 0.573 | 0.852 | 1.000 | 5804 | tags=25%, list=18%, signal=31% |
| 1742 | ATP\_SYNTHESIS\_COUPLED\_ELECTRON\_TRANSPORT(GO:0042773) |  | 48 | -0.20 | -0.84 | 0.695 | 0.853 | 1.000 | 10812 | tags=48%, list=34%, signal=72% |
| 1743 | SEQUENCE\_SPECIFIC\_DNA\_BINDING(GO:0043565) |  | 1349 | -0.16 | -0.84 | 0.713 | 0.853 | 1.000 | 6143 | tags=16%, list=19%, signal=19% |
| 1744 | CELLULAR\_RESPONSE\_TO\_DSRNA(GO:0071359) |  | 19 | -0.22 | -0.84 | 0.669 | 0.853 | 1.000 | 2792 | tags=11%, list=9%, signal=12% |
| 1745 | NEGATIVE\_REGULATION\_OF\_LOCOMOTION(GO:0040013) |  | 70 | -0.21 | -0.84 | 0.702 | 0.853 | 1.000 | 2317 | tags=7%, list=7%, signal=8% |
| 1746 | NEGATIVE\_REGULATION\_OF\_CELLULAR\_COMPONENT\_MOVEMENT(GO:0051271) |  | 70 | -0.21 | -0.84 | 0.702 | 0.853 | 1.000 | 2317 | tags=7%, list=7%, signal=8% |
| 1747 | CELLULAR\_RESPONSE\_TO\_DECREASED\_OXYGEN\_LEVELS(GO:0036294) |  | 22 | -0.24 | -0.84 | 0.679 | 0.853 | 1.000 | 7768 | tags=32%, list=24%, signal=42% |
| 1748 | PHOSPHOLIPID\_BIOSYNTHETIC\_PROCESS(GO:0008654) |  | 100 | -0.18 | -0.84 | 0.700 | 0.852 | 1.000 | 3226 | tags=8%, list=10%, signal=9% |
| 1749 | KIDNEY\_EPITHELIUM\_DEVELOPMENT(GO:0072073) |  | 64 | -0.21 | -0.84 | 0.641 | 0.852 | 1.000 | 8725 | tags=28%, list=27%, signal=39% |
| 1750 | MUSCLE\_TISSUE\_MORPHOGENESIS(GO:0060415) |  | 20 | -0.26 | -0.84 | 0.673 | 0.853 | 1.000 | 4131 | tags=15%, list=13%, signal=17% |
| 1751 | NEGATIVE\_REGULATION\_OF\_CELLULAR\_BIOSYNTHETIC\_PROCESS(GO:0031327) |  | 466 | -0.14 | -0.84 | 0.890 | 0.852 | 1.000 | 8014 | tags=21%, list=25%, signal=28% |
| 1752 | POSITIVE\_REGULATION\_OF\_PROTEIN\_CATABOLIC\_PROCESS(GO:0045732) |  | 53 | -0.17 | -0.84 | 0.656 | 0.852 | 1.000 | 6359 | tags=19%, list=20%, signal=24% |
| 1753 | HYALURONAN\_METABOLIC\_PROCESS(GO:0030212) |  | 16 | -0.30 | -0.84 | 0.737 | 0.854 | 1.000 | 6188 | tags=38%, list=19%, signal=47% |
| 1754 | ION\_TRANSMEMBRANE\_TRANSPORT(GO:0034220) |  | 674 | -0.17 | -0.83 | 0.784 | 0.854 | 1.000 | 5936 | tags=18%, list=19%, signal=21% |
| 1755 | NAD\_BIOSYNTHETIC\_PROCESS(GO:0009435) |  | 18 | -0.29 | -0.83 | 0.745 | 0.855 | 1.000 | 6698 | tags=39%, list=21%, signal=49% |
| 1756 | MAINTENANCE\_OF\_PROTEIN\_LOCATION(GO:0045185) |  | 24 | -0.24 | -0.83 | 0.710 | 0.856 | 1.000 | 1833 | tags=13%, list=6%, signal=13% |
| 1757 | MICROTUBULE\_ASSOCIATED\_COMPLEX(GO:0005875) |  | 117 | -0.17 | -0.83 | 0.762 | 0.856 | 1.000 | 9553 | tags=35%, list=30%, signal=50% |
| 1758 | NEGATIVE\_REGULATION\_OF\_ANGIOGENESIS(GO:0016525) |  | 28 | -0.24 | -0.83 | 0.636 | 0.857 | 1.000 | 3585 | tags=14%, list=11%, signal=16% |
| 1759 | INOSITOL\_LIPID\_MEDIATED\_SIGNALING(GO:0048017) |  | 54 | -0.22 | -0.83 | 0.596 | 0.856 | 1.000 | 8786 | tags=35%, list=28%, signal=48% |
| 1760 | DNA\_BINDING\_TRANSCRIPTION\_FACTOR\_ACTIVITY(GO:0003700) |  | 1278 | -0.16 | -0.83 | 0.764 | 0.856 | 1.000 | 6143 | tags=17%, list=19%, signal=20% |
| 1761 | ENSHEATHMENT\_OF\_NEURONS(GO:0007272) |  | 71 | -0.18 | -0.83 | 0.649 | 0.856 | 1.000 | 3649 | tags=8%, list=11%, signal=10% |
| 1762 | AXON\_ENSHEATHMENT(GO:0008366) |  | 71 | -0.18 | -0.83 | 0.649 | 0.856 | 1.000 | 3649 | tags=8%, list=11%, signal=10% |
| 1763 | CILIUM\_MOVEMENT(GO:0003341) |  | 66 | -0.24 | -0.83 | 0.731 | 0.857 | 1.000 | 8595 | tags=38%, list=27%, signal=52% |
| 1764 | NEGATIVE\_REGULATION\_OF\_BIOSYNTHETIC\_PROCESS(GO:0009890) |  | 469 | -0.14 | -0.83 | 0.890 | 0.857 | 1.000 | 8014 | tags=21%, list=25%, signal=28% |
| 1765 | NEGATIVE\_REGULATION\_OF\_VASCULATURE\_DEVELOPMENT(GO:1901343) |  | 30 | -0.23 | -0.83 | 0.664 | 0.857 | 1.000 | 3585 | tags=13%, list=11%, signal=15% |
| 1766 | TRANSMEMBRANE\_TRANSPORT(GO:0055085) |  | 1058 | -0.17 | -0.83 | 0.806 | 0.858 | 1.000 | 5936 | tags=17%, list=19%, signal=20% |
| 1767 | HEMOGLOBIN\_BIOSYNTHETIC\_PROCESS(GO:0042541) |  | 16 | -0.23 | -0.83 | 0.726 | 0.859 | 1.000 | 9290 | tags=44%, list=29%, signal=62% |
| 1768 | TISSUE\_REGENERATION(GO:0042246) |  | 132 | -0.16 | -0.83 | 0.693 | 0.859 | 1.000 | 5720 | tags=14%, list=18%, signal=17% |
| 1769 | NEUROTRANSMITTER\_TRANSPORT(GO:0006836) |  | 113 | -0.22 | -0.83 | 0.741 | 0.859 | 1.000 | 9031 | tags=38%, list=28%, signal=53% |
| 1770 | PLASMA\_MEMBRANE\_INVAGINATION(GO:0099024) |  | 35 | -0.25 | -0.83 | 0.629 | 0.858 | 1.000 | 5804 | tags=26%, list=18%, signal=31% |
| 1771 | GLYCOSPHINGOLIPID\_METABOLIC\_PROCESS(GO:0006687) |  | 30 | -0.25 | -0.83 | 0.734 | 0.858 | 1.000 | 99 | tags=3%, list=0%, signal=3% |
| 1772 | SNRNA\_BINDING(GO:0017069) |  | 28 | -0.19 | -0.83 | 0.739 | 0.858 | 1.000 | 25909 | tags=100%, list=81%, signal=533% |
| 1773 | APOPTOTIC\_MITOCHONDRIAL\_CHANGES(GO:0008637) |  | 20 | -0.27 | -0.83 | 0.592 | 0.862 | 1.000 | 3925 | tags=15%, list=12%, signal=17% |
| 1774 | CELL\_BODY(GO:0044297) |  | 82 | -0.21 | -0.82 | 0.665 | 0.863 | 1.000 | 5629 | tags=18%, list=18%, signal=22% |
| 1775 | PROTEIN\_PHOSPHATASE\_TYPE\_2A\_COMPLEX(GO:0000159) |  | 19 | -0.24 | -0.82 | 0.687 | 0.862 | 1.000 | 3000 | tags=11%, list=9%, signal=12% |
| 1776 | KINASE\_BINDING(GO:0019900) |  | 159 | -0.15 | -0.82 | 0.840 | 0.864 | 1.000 | 8738 | tags=26%, list=27%, signal=36% |
| 1777 | STEROID\_DEHYDROGENASE\_ACTIVITY\_\_ACTING\_ON\_THE\_CH\_OH\_GROUP\_OF\_DONORS\_\_NAD\_OR\_NADP\_AS\_ACCEPTOR(GO:0033764) |  | 19 | -0.31 | -0.82 | 0.701 | 0.867 | 1.000 | 9284 | tags=47%, list=29%, signal=67% |
| 1778 | PROTEIN\_LOCALIZATION\_TO\_NUCLEUS(GO:0034504) |  | 60 | -0.15 | -0.82 | 0.665 | 0.867 | 1.000 | 1561 | tags=3%, list=5%, signal=3% |
| 1779 | OTOLITH\_DEVELOPMENT(GO:0048840) |  | 49 | -0.19 | -0.82 | 0.671 | 0.867 | 1.000 | 7782 | tags=22%, list=24%, signal=30% |
| 1780 | RIBOSOMAL\_SMALL\_SUBUNIT\_ASSEMBLY(GO:0000028) |  | 15 | -0.33 | -0.82 | 0.734 | 0.867 | 1.000 | 8377 | tags=33%, list=26%, signal=45% |
| 1781 | ANIMAL\_ORGAN\_MORPHOGENESIS(GO:0009887) |  | 797 | -0.14 | -0.82 | 0.740 | 0.867 | 1.000 | 6583 | tags=17%, list=21%, signal=21% |
| 1782 | GTPASE\_ACTIVATOR\_ACTIVITY(GO:0005096) |  | 207 | -0.16 | -0.82 | 0.651 | 0.867 | 1.000 | 6174 | tags=15%, list=19%, signal=18% |
| 1783 | RETINOID\_BINDING(GO:0005501) |  | 16 | -0.29 | -0.82 | 0.667 | 0.868 | 1.000 | 3696 | tags=19%, list=12%, signal=21% |
| 1784 | ISOPRENOID\_BINDING(GO:0019840) |  | 16 | -0.29 | -0.82 | 0.667 | 0.868 | 1.000 | 3696 | tags=19%, list=12%, signal=21% |
| 1785 | TRANSMEMBRANE\_TRANSPORTER\_COMPLEX(GO:1902495) |  | 211 | -0.20 | -0.82 | 0.752 | 0.867 | 1.000 | 5655 | tags=18%, list=18%, signal=22% |
| 1786 | FIN\_DEVELOPMENT(GO:0033333) |  | 99 | -0.18 | -0.82 | 0.624 | 0.867 | 1.000 | 8995 | tags=30%, list=28%, signal=42% |
| 1787 | PLASMA\_MEMBRANE\_BOUNDED\_CELL\_PROJECTION(GO:0120025) |  | 899 | -0.17 | -0.82 | 0.762 | 0.868 | 1.000 | 5462 | tags=16%, list=17%, signal=19% |
| 1788 | PROTON\_TRANSPORTING\_V\_TYPE\_ATPASE\_COMPLEX(GO:0033176) |  | 29 | -0.29 | -0.82 | 0.627 | 0.868 | 1.000 | 6833 | tags=38%, list=21%, signal=48% |
| 1789 | GLOMERULAR\_FILTRATION(GO:0003094) |  | 23 | -0.20 | -0.82 | 0.788 | 0.868 | 1.000 | 3956 | tags=13%, list=12%, signal=15% |
| 1790 | OTIC\_VESICLE\_FORMATION(GO:0030916) |  | 25 | -0.24 | -0.82 | 0.630 | 0.868 | 1.000 | 10821 | tags=48%, list=34%, signal=73% |
| 1791 | ATRIOVENTRICULAR\_VALVE\_DEVELOPMENT(GO:0003171) |  | 25 | -0.23 | -0.82 | 0.739 | 0.868 | 1.000 | 5013 | tags=20%, list=16%, signal=24% |
| 1792 | MULTICELLULAR\_ORGANISMAL\_SIGNALING(GO:0035637) |  | 42 | -0.25 | -0.82 | 0.643 | 0.869 | 1.000 | 7645 | tags=33%, list=24%, signal=44% |
| 1793 | DEVELOPMENTAL\_MATURATION(GO:0021700) |  | 92 | -0.18 | -0.82 | 0.750 | 0.869 | 1.000 | 9407 | tags=30%, list=29%, signal=43% |
| 1794 | RESPIRATORY\_ELECTRON\_TRANSPORT\_CHAIN(GO:0022904) |  | 61 | -0.19 | -0.82 | 0.653 | 0.869 | 1.000 | 10812 | tags=46%, list=34%, signal=69% |
| 1795 | IRON\_SULFUR\_CLUSTER\_ASSEMBLY(GO:0016226) |  | 19 | -0.28 | -0.81 | 0.653 | 0.870 | 1.000 | 6476 | tags=26%, list=20%, signal=33% |
| 1796 | METALLO\_SULFUR\_CLUSTER\_ASSEMBLY(GO:0031163) |  | 19 | -0.28 | -0.81 | 0.653 | 0.869 | 1.000 | 6476 | tags=26%, list=20%, signal=33% |
| 1797 | MUCOPOLYSACCHARIDE\_METABOLIC\_PROCESS(GO:1903510) |  | 38 | -0.24 | -0.81 | 0.737 | 0.872 | 1.000 | 6188 | tags=29%, list=19%, signal=36% |
| 1798 | POSTSYNAPTIC\_SPECIALIZATION(GO:0099572) |  | 108 | -0.22 | -0.81 | 0.753 | 0.871 | 1.000 | 8983 | tags=36%, list=28%, signal=50% |
| 1799 | SELECTIVE\_AUTOPHAGY(GO:0061912) |  | 18 | -0.26 | -0.81 | 0.745 | 0.873 | 1.000 | 9694 | tags=44%, list=30%, signal=64% |
| 1800 | DENDRITE(GO:0030425) |  | 191 | -0.20 | -0.81 | 0.778 | 0.873 | 1.000 | 5841 | tags=20%, list=18%, signal=24% |
| 1801 | DENDRITIC\_TREE(GO:0097447) |  | 191 | -0.20 | -0.81 | 0.778 | 0.872 | 1.000 | 5841 | tags=20%, list=18%, signal=24% |
| 1802 | MEDIATOR\_COMPLEX(GO:0016592) |  | 39 | -0.19 | -0.81 | 0.833 | 0.874 | 1.000 | 7649 | tags=21%, list=24%, signal=27% |
| 1803 | REGULATION\_OF\_MACROAUTOPHAGY(GO:0016241) |  | 24 | -0.24 | -0.81 | 0.614 | 0.874 | 1.000 | 13661 | tags=71%, list=43%, signal=124% |
| 1804 | AXON\_PART(GO:0033267) |  | 70 | -0.22 | -0.81 | 0.815 | 0.874 | 1.000 | 9964 | tags=37%, list=31%, signal=54% |
| 1805 | POSITIVE\_REGULATION\_OF\_CATABOLIC\_PROCESS(GO:0009896) |  | 96 | -0.16 | -0.81 | 0.918 | 0.874 | 1.000 | 6494 | tags=19%, list=20%, signal=23% |
| 1806 | TRANSPORTER\_COMPLEX(GO:1990351) |  | 217 | -0.19 | -0.81 | 0.772 | 0.875 | 1.000 | 5655 | tags=18%, list=18%, signal=21% |
| 1807 | POSITIVE\_REGULATION\_OF\_DNA\_TEMPLATED\_TRANSCRIPTION\_\_ELONGATION(GO:0032786) |  | 15 | -0.25 | -0.81 | 0.573 | 0.875 | 1.000 | 6864 | tags=33%, list=22%, signal=42% |
| 1808 | NEGATIVE\_REGULATION\_OF\_BLOOD\_VESSEL\_MORPHOGENESIS(GO:2000181) |  | 29 | -0.23 | -0.81 | 0.672 | 0.875 | 1.000 | 3585 | tags=14%, list=11%, signal=16% |
| 1809 | POSITIVE\_REGULATION\_OF\_MITOTIC\_CELL\_CYCLE\_PHASE\_TRANSITION(GO:1901992) |  | 20 | -0.26 | -0.81 | 0.579 | 0.875 | 1.000 | 5919 | tags=25%, list=19%, signal=31% |
| 1810 | PHOSPHATIDYLINOSITOL\_BIOSYNTHETIC\_PROCESS(GO:0006661) |  | 42 | -0.20 | -0.81 | 0.664 | 0.876 | 1.000 | 3226 | tags=7%, list=10%, signal=8% |
| 1811 | INTRINSIC\_COMPONENT\_OF\_ORGANELLE\_MEMBRANE(GO:0031300) |  | 152 | -0.17 | -0.81 | 0.835 | 0.876 | 1.000 | 4021 | tags=12%, list=13%, signal=13% |
| 1812 | PITUITARY\_GLAND\_DEVELOPMENT(GO:0021983) |  | 17 | -0.22 | -0.81 | 0.695 | 0.876 | 1.000 | 24923 | tags=100%, list=78%, signal=457% |
| 1813 | POSITIVE\_REGULATION\_OF\_CELLULAR\_PROTEIN\_LOCALIZATION(GO:1903829) |  | 25 | -0.24 | -0.81 | 0.645 | 0.876 | 1.000 | 9792 | tags=48%, list=31%, signal=69% |
| 1814 | POTASSIUM\_CHANNEL\_COMPLEX(GO:0034705) |  | 46 | -0.21 | -0.80 | 0.847 | 0.877 | 1.000 | 4468 | tags=22%, list=14%, signal=25% |
| 1815 | AMINOGLYCAN\_BIOSYNTHETIC\_PROCESS(GO:0006023) |  | 41 | -0.23 | -0.80 | 0.685 | 0.878 | 1.000 | 5488 | tags=22%, list=17%, signal=26% |
| 1816 | NEGATIVE\_REGULATION\_OF\_MACROMOLECULE\_BIOSYNTHETIC\_PROCESS(GO:0010558) |  | 444 | -0.14 | -0.80 | 0.834 | 0.877 | 1.000 | 8014 | tags=21%, list=25%, signal=27% |
| 1817 | NEGATIVE\_REGULATION\_OF\_CELLULAR\_MACROMOLECULE\_BIOSYNTHETIC\_PROCESS(GO:2000113) |  | 443 | -0.14 | -0.80 | 0.834 | 0.877 | 1.000 | 8014 | tags=21%, list=25%, signal=27% |
| 1818 | DETERMINATION\_OF\_VENTRAL\_IDENTITY(GO:0048264) |  | 20 | -0.20 | -0.80 | 0.688 | 0.877 | 1.000 | 1117 | tags=5%, list=4%, signal=5% |
| 1819 | SOMATODENDRITIC\_COMPARTMENT(GO:0036477) |  | 229 | -0.19 | -0.80 | 0.705 | 0.877 | 1.000 | 5841 | tags=20%, list=18%, signal=24% |
| 1820 | STEROID\_DEHYDROGENASE\_ACTIVITY(GO:0016229) |  | 25 | -0.30 | -0.80 | 0.698 | 0.877 | 1.000 | 3622 | tags=20%, list=11%, signal=23% |
| 1821 | STRUCTURAL\_CONSTITUENT\_OF\_NUCLEAR\_PORE(GO:0017056) |  | 18 | -0.21 | -0.80 | 0.725 | 0.877 | 1.000 | 25158 | tags=100%, list=79%, signal=473% |
| 1822 | PROTON\_TRANSMEMBRANE\_TRANSPORTER\_ACTIVITY(GO:0015078) |  | 100 | -0.18 | -0.80 | 0.629 | 0.878 | 1.000 | 9074 | tags=34%, list=28%, signal=47% |
| 1823 | AXONEME\_ASSEMBLY(GO:0035082) |  | 45 | -0.25 | -0.80 | 0.684 | 0.877 | 1.000 | 9659 | tags=42%, list=30%, signal=60% |
| 1824 | MUSCLE\_ORGAN\_MORPHOGENESIS(GO:0048644) |  | 21 | -0.26 | -0.80 | 0.697 | 0.878 | 1.000 | 214 | tags=5%, list=1%, signal=5% |
| 1825 | IMMUNE\_RESPONSE\_REGULATING\_CELL\_SURFACE\_RECEPTOR\_SIGNALING\_PATHWAY(GO:0002768) |  | 53 | -0.23 | -0.80 | 0.822 | 0.880 | 1.000 | 2821 | tags=13%, list=9%, signal=14% |
| 1826 | MEDIAL\_FIN\_DEVELOPMENT(GO:0033338) |  | 19 | -0.22 | -0.80 | 0.692 | 0.880 | 1.000 | 9443 | tags=37%, list=30%, signal=52% |
| 1827 | MITOCHONDRIAL\_RNA\_METABOLIC\_PROCESS(GO:0000959) |  | 24 | -0.25 | -0.80 | 0.630 | 0.883 | 1.000 | 357 | tags=4%, list=1%, signal=4% |
| 1828 | REGULATION\_OF\_LEUKOCYTE\_DIFFERENTIATION(GO:1902105) |  | 34 | -0.24 | -0.80 | 0.719 | 0.885 | 1.000 | 4847 | tags=18%, list=15%, signal=21% |
| 1829 | EMBRYONIC\_CAMERA\_TYPE\_EYE\_FORMATION(GO:0060900) |  | 32 | -0.23 | -0.80 | 0.610 | 0.885 | 1.000 | 10972 | tags=38%, list=34%, signal=57% |
| 1830 | CYCLIN\_DEPENDENT\_PROTEIN\_SERINE\_THREONINE\_KINASE\_ACTIVITY(GO:0004693) |  | 18 | -0.26 | -0.80 | 0.707 | 0.885 | 1.000 | 6984 | tags=33%, list=22%, signal=43% |
| 1831 | CYCLIN\_DEPENDENT\_PROTEIN\_KINASE\_ACTIVITY(GO:0097472) |  | 18 | -0.26 | -0.80 | 0.707 | 0.884 | 1.000 | 6984 | tags=33%, list=22%, signal=43% |
| 1832 | MITOTIC\_SISTER\_CHROMATID\_COHESION(GO:0007064) |  | 18 | -0.25 | -0.79 | 0.787 | 0.888 | 1.000 | 6795 | tags=28%, list=21%, signal=35% |
| 1833 | STRUCTURAL\_MOLECULE\_ACTIVITY(GO:0005198) |  | 505 | -0.18 | -0.79 | 0.600 | 0.888 | 1.000 | 8752 | tags=27%, list=27%, signal=36% |
| 1834 | PRONEPHROS\_MORPHOGENESIS(GO:0072114) |  | 25 | -0.24 | -0.79 | 0.729 | 0.890 | 1.000 | 4347 | tags=12%, list=14%, signal=14% |
| 1835 | NERVOUS\_SYSTEM\_DEVELOPMENT(GO:0007399) |  | 1676 | -0.14 | -0.79 | 0.811 | 0.891 | 1.000 | 6888 | tags=17%, list=22%, signal=20% |
| 1836 | TAXIS(GO:0042330) |  | 401 | -0.15 | -0.79 | 0.867 | 0.891 | 1.000 | 8353 | tags=23%, list=26%, signal=31% |
| 1837 | DENDRITE\_MEMBRANE(GO:0032590) |  | 21 | -0.27 | -0.79 | 0.816 | 0.892 | 1.000 | 5629 | tags=24%, list=18%, signal=29% |
| 1838 | NEURONAL\_CELL\_BODY(GO:0043025) |  | 78 | -0.20 | -0.79 | 0.730 | 0.891 | 1.000 | 6844 | tags=22%, list=21%, signal=28% |
| 1839 | GTPASE\_REGULATOR\_ACTIVITY(GO:0030695) |  | 213 | -0.15 | -0.79 | 0.651 | 0.895 | 1.000 | 4365 | tags=10%, list=14%, signal=12% |
| 1840 | CALCIUM\_DEPENDENT\_PHOSPHOLIPID\_BINDING(GO:0005544) |  | 58 | -0.22 | -0.79 | 0.778 | 0.895 | 1.000 | 7788 | tags=28%, list=24%, signal=36% |
| 1841 | ELECTRON\_TRANSPORT\_CHAIN(GO:0022900) |  | 63 | -0.19 | -0.78 | 0.760 | 0.895 | 1.000 | 11287 | tags=48%, list=35%, signal=74% |
| 1842 | CELLULAR\_RESPONSE\_TO\_OXYGEN\_LEVELS(GO:0071453) |  | 23 | -0.22 | -0.78 | 0.857 | 0.902 | 1.000 | 7768 | tags=30%, list=24%, signal=40% |
| 1843 | CELL\_MIGRATION\_INVOLVED\_IN\_HEART\_DEVELOPMENT(GO:0060973) |  | 26 | -0.22 | -0.78 | 0.722 | 0.902 | 1.000 | 9576 | tags=31%, list=30%, signal=44% |
| 1844 | APPENDAGE\_DEVELOPMENT(GO:0048736) |  | 107 | -0.17 | -0.78 | 0.733 | 0.906 | 1.000 | 8995 | tags=31%, list=28%, signal=43% |
| 1845 | NEGATIVE\_REGULATION\_OF\_TRANSCRIPTION\_\_DNA\_TEMPLATED(GO:0045892) |  | 324 | -0.14 | -0.78 | 0.871 | 0.907 | 1.000 | 6367 | tags=15%, list=20%, signal=18% |
| 1846 | NEGATIVE\_REGULATION\_OF\_RNA\_BIOSYNTHETIC\_PROCESS(GO:1902679) |  | 325 | -0.14 | -0.78 | 0.871 | 0.907 | 1.000 | 6367 | tags=15%, list=20%, signal=18% |
| 1847 | NEGATIVE\_REGULATION\_OF\_NUCLEIC\_ACID\_TEMPLATED\_TRANSCRIPTION(GO:1903507) |  | 325 | -0.14 | -0.78 | 0.871 | 0.906 | 1.000 | 6367 | tags=15%, list=20%, signal=18% |
| 1848 | METHYLATION(GO:0032259) |  | 210 | -0.14 | -0.77 | 0.838 | 0.908 | 1.000 | 4273 | tags=9%, list=13%, signal=10% |
| 1849 | SMALL\_NUCLEAR\_RIBONUCLEOPROTEIN\_COMPLEX(GO:0030532) |  | 48 | -0.18 | -0.77 | 0.583 | 0.908 | 1.000 | 5565 | tags=10%, list=17%, signal=13% |
| 1850 | CARBOHYDRATE\_DERIVATIVE\_TRANSMEMBRANE\_TRANSPORTER\_ACTIVITY(GO:1901505) |  | 37 | -0.20 | -0.77 | 0.782 | 0.909 | 1.000 | 3772 | tags=11%, list=12%, signal=12% |
| 1851 | TRANS\_GOLGI\_NETWORK\_TRANSPORT\_VESICLE(GO:0030140) |  | 18 | -0.24 | -0.77 | 0.794 | 0.911 | 1.000 | 3210 | tags=11%, list=10%, signal=12% |
| 1852 | VOLTAGE\_GATED\_CHANNEL\_ACTIVITY(GO:0022832) |  | 197 | -0.17 | -0.77 | 0.919 | 0.910 | 1.000 | 5347 | tags=17%, list=17%, signal=20% |
| 1853 | ANIMAL\_ORGAN\_FORMATION(GO:0048645) |  | 34 | -0.22 | -0.77 | 0.750 | 0.911 | 1.000 | 5939 | tags=21%, list=19%, signal=25% |
| 1854 | PHOSPHOLIPASE\_C\_ACTIVATING\_G\_PROTEIN\_COUPLED\_RECEPTOR\_SIGNALING\_PATHWAY(GO:0007200) |  | 62 | -0.19 | -0.77 | 0.942 | 0.914 | 1.000 | 8123 | tags=31%, list=25%, signal=41% |
| 1855 | NEUROPEPTIDE\_BINDING(GO:0042923) |  | 18 | -0.28 | -0.77 | 0.762 | 0.914 | 1.000 | 3302 | tags=17%, list=10%, signal=19% |
| 1856 | CYTOPLASMIC\_MICROTUBULE\_ORGANIZATION(GO:0031122) |  | 30 | -0.20 | -0.77 | 0.836 | 0.915 | 1.000 | 4881 | tags=13%, list=15%, signal=16% |
| 1857 | SPLICEOSOMAL\_COMPLEX(GO:0005681) |  | 130 | -0.16 | -0.77 | 0.619 | 0.915 | 1.000 | 7923 | tags=18%, list=25%, signal=24% |
| 1858 | POSITIVE\_REGULATION\_OF\_EPITHELIAL\_CELL\_PROLIFERATION(GO:0050679) |  | 20 | -0.25 | -0.77 | 0.746 | 0.915 | 1.000 | 1743 | tags=10%, list=5%, signal=11% |
| 1859 | POSITIVE\_REGULATION\_OF\_ACTIN\_FILAMENT\_POLYMERIZATION(GO:0030838) |  | 71 | -0.16 | -0.76 | 0.940 | 0.917 | 1.000 | 3541 | tags=10%, list=11%, signal=11% |
| 1860 | CALCIUM\_ION\_TRANSMEMBRANE\_TRANSPORT(GO:0070588) |  | 144 | -0.18 | -0.76 | 0.874 | 0.916 | 1.000 | 5936 | tags=19%, list=19%, signal=24% |
| 1861 | EXTRINSIC\_APOPTOTIC\_SIGNALING\_PATHWAY\_VIA\_DEATH\_DOMAIN\_RECEPTORS(GO:0008625) |  | 15 | -0.26 | -0.76 | 0.700 | 0.917 | 1.000 | 9978 | tags=47%, list=31%, signal=68% |
| 1862 | NUCLEAR\_INNER\_MEMBRANE(GO:0005637) |  | 29 | -0.26 | -0.76 | 0.651 | 0.917 | 1.000 | 10465 | tags=52%, list=33%, signal=77% |
| 1863 | REGULATION\_OF\_WNT\_SIGNALING\_PATHWAY(GO:0030111) |  | 167 | -0.15 | -0.76 | 0.803 | 0.917 | 1.000 | 9712 | tags=32%, list=30%, signal=46% |
| 1864 | INNER\_EAR\_DEVELOPMENT(GO:0048839) |  | 165 | -0.13 | -0.76 | 0.854 | 0.916 | 1.000 | 8983 | tags=24%, list=28%, signal=34% |
| 1865 | ADENOHYPOPHYSIS\_DEVELOPMENT(GO:0021984) |  | 15 | -0.22 | -0.76 | 0.703 | 0.917 | 1.000 | 24923 | tags=100%, list=78%, signal=457% |
| 1866 | G\_PROTEIN\_COUPLED\_RECEPTOR\_SIGNALING\_PATHWAY(GO:0007186) |  | 774 | -0.17 | -0.76 | 0.949 | 0.917 | 1.000 | 5329 | tags=15%, list=17%, signal=18% |
| 1867 | SIGNAL\_RELEASE(GO:0023061) |  | 114 | -0.21 | -0.76 | 0.848 | 0.918 | 1.000 | 9283 | tags=34%, list=29%, signal=48% |
| 1868 | PHOTORECEPTOR\_CELL\_DIFFERENTIATION(GO:0046530) |  | 94 | -0.20 | -0.76 | 0.814 | 0.918 | 1.000 | 7786 | tags=26%, list=24%, signal=34% |
| 1869 | SISTER\_CHROMATID\_COHESION(GO:0007062) |  | 35 | -0.20 | -0.76 | 0.758 | 0.918 | 1.000 | 6795 | tags=26%, list=21%, signal=33% |
| 1870 | NEURON\_DIFFERENTIATION(GO:0030182) |  | 926 | -0.14 | -0.76 | 0.777 | 0.917 | 1.000 | 8289 | tags=22%, list=26%, signal=29% |
| 1871 | POTASSIUM\_ION\_TRANSMEMBRANE\_TRANSPORTER\_ACTIVITY(GO:0015079) |  | 166 | -0.17 | -0.76 | 0.907 | 0.918 | 1.000 | 8251 | tags=28%, list=26%, signal=38% |
| 1872 | ADRENERGIC\_RECEPTOR\_SIGNALING\_PATHWAY(GO:0071875) |  | 28 | -0.23 | -0.76 | 0.815 | 0.920 | 1.000 | 7226 | tags=32%, list=23%, signal=42% |
| 1873 | CELL\_CELL\_SIGNALING(GO:0007267) |  | 715 | -0.16 | -0.75 | 0.886 | 0.924 | 1.000 | 9517 | tags=31%, list=30%, signal=43% |
| 1874 | REGIONALIZATION(GO:0003002) |  | 384 | -0.13 | -0.75 | 0.873 | 0.925 | 1.000 | 6094 | tags=14%, list=19%, signal=17% |
| 1875 | PRESYNAPTIC\_ACTIVE\_ZONE(GO:0048786) |  | 38 | -0.23 | -0.75 | 0.752 | 0.925 | 1.000 | 9031 | tags=39%, list=28%, signal=55% |
| 1876 | NUCLEOBASE\_METABOLIC\_PROCESS(GO:0009112) |  | 38 | -0.21 | -0.75 | 0.792 | 0.925 | 1.000 | 7606 | tags=26%, list=24%, signal=35% |
| 1877 | REGULATION\_OF\_EPITHELIAL\_CELL\_PROLIFERATION(GO:0050678) |  | 30 | -0.19 | -0.75 | 0.726 | 0.926 | 1.000 | 1743 | tags=7%, list=5%, signal=7% |
| 1878 | GENERATION\_OF\_NEURONS(GO:0048699) |  | 998 | -0.14 | -0.75 | 0.772 | 0.925 | 1.000 | 8289 | tags=22%, list=26%, signal=29% |
| 1879 | POSITIVE\_REGULATION\_OF\_WNT\_SIGNALING\_PATHWAY(GO:0030177) |  | 63 | -0.16 | -0.75 | 0.953 | 0.926 | 1.000 | 9712 | tags=30%, list=30%, signal=43% |
| 1880 | LYMPHOCYTE\_ACTIVATION\_INVOLVED\_IN\_IMMUNE\_RESPONSE(GO:0002285) |  | 22 | -0.24 | -0.75 | 0.803 | 0.926 | 1.000 | 1287 | tags=9%, list=4%, signal=9% |
| 1881 | N\_TERMINAL\_PROTEIN\_AMINO\_ACID\_MODIFICATION(GO:0031365) |  | 18 | -0.20 | -0.75 | 0.774 | 0.927 | 1.000 | 6370 | tags=17%, list=20%, signal=21% |
| 1882 | PHARYNGEAL\_SYSTEM\_DEVELOPMENT(GO:0060037) |  | 30 | -0.16 | -0.75 | 0.790 | 0.926 | 1.000 | 6217 | tags=13%, list=19%, signal=17% |
| 1883 | REGULATION\_OF\_RHO\_PROTEIN\_SIGNAL\_TRANSDUCTION(GO:0035023) |  | 62 | -0.21 | -0.75 | 0.822 | 0.926 | 1.000 | 5877 | tags=21%, list=18%, signal=26% |
| 1884 | PYRIMIDINE\_NUCLEOSIDE\_TRIPHOSPHATE\_METABOLIC\_PROCESS(GO:0009147) |  | 22 | -0.25 | -0.75 | 0.820 | 0.930 | 1.000 | 8837 | tags=41%, list=28%, signal=57% |
| 1885 | DISULFIDE\_OXIDOREDUCTASE\_ACTIVITY(GO:0015036) |  | 30 | -0.19 | -0.74 | 0.794 | 0.931 | 1.000 | 5364 | tags=17%, list=17%, signal=20% |
| 1886 | FLAGELLATED\_SPERM\_MOTILITY(GO:0030317) |  | 21 | -0.25 | -0.74 | 0.782 | 0.931 | 1.000 | 4959 | tags=24%, list=16%, signal=28% |
| 1887 | SPERM\_MOTILITY(GO:0097722) |  | 21 | -0.25 | -0.74 | 0.782 | 0.930 | 1.000 | 4959 | tags=24%, list=16%, signal=28% |
| 1888 | RNA\_POLYMERASE\_CORE\_ENZYME\_BINDING(GO:0043175) |  | 28 | -0.15 | -0.74 | 0.825 | 0.930 | 1.000 | 7074 | tags=21%, list=22%, signal=28% |
| 1889 | ION\_CHANNEL\_COMPLEX(GO:0034702) |  | 202 | -0.18 | -0.74 | 0.828 | 0.931 | 1.000 | 5655 | tags=18%, list=18%, signal=22% |
| 1890 | POSITIVE\_REGULATION\_OF\_DNA\_BINDING\_TRANSCRIPTION\_FACTOR\_ACTIVITY(GO:0051091) |  | 47 | -0.16 | -0.74 | 0.888 | 0.932 | 1.000 | 10018 | tags=36%, list=31%, signal=53% |
| 1891 | CYCLIC\_NUCLEOTIDE\_PHOSPHODIESTERASE\_ACTIVITY(GO:0004112) |  | 29 | -0.23 | -0.74 | 0.766 | 0.932 | 1.000 | 10713 | tags=38%, list=34%, signal=57% |
| 1892 | MITOCHONDRIAL\_ATP\_SYNTHESIS\_COUPLED\_ELECTRON\_TRANSPORT(GO:0042775) |  | 44 | -0.19 | -0.74 | 0.800 | 0.932 | 1.000 | 10812 | tags=48%, list=34%, signal=72% |
| 1893 | PRONEPHROS\_DEVELOPMENT(GO:0048793) |  | 128 | -0.15 | -0.74 | 0.895 | 0.932 | 1.000 | 6681 | tags=18%, list=21%, signal=23% |
| 1894 | REGULATION\_OF\_CALCIUM\_ION\_TRANSMEMBRANE\_TRANSPORTER\_ACTIVITY(GO:1901019) |  | 24 | -0.24 | -0.74 | 0.823 | 0.932 | 1.000 | 5831 | tags=25%, list=18%, signal=31% |
| 1895 | CALCIUM\_DEPENDENT\_PROTEIN\_BINDING(GO:0048306) |  | 17 | -0.29 | -0.74 | 0.700 | 0.931 | 1.000 | 5054 | tags=29%, list=16%, signal=35% |
| 1896 | REGULATION\_OF\_MICROTUBULE\_POLYMERIZATION\_OR\_DEPOLYMERIZATION(GO:0031110) |  | 37 | -0.19 | -0.74 | 0.884 | 0.932 | 1.000 | 3272 | tags=11%, list=10%, signal=12% |
| 1897 | AXON\_GUIDANCE(GO:0007411) |  | 261 | -0.15 | -0.74 | 0.822 | 0.933 | 1.000 | 8353 | tags=23%, list=26%, signal=30% |
| 1898 | EMBRYONIC\_EYE\_MORPHOGENESIS(GO:0048048) |  | 50 | -0.19 | -0.74 | 0.815 | 0.933 | 1.000 | 11005 | tags=38%, list=35%, signal=58% |
| 1899 | SPLICEOSOMAL\_SNRNP\_COMPLEX(GO:0097525) |  | 47 | -0.18 | -0.74 | 0.650 | 0.933 | 1.000 | 5565 | tags=11%, list=17%, signal=13% |
| 1900 | DORSAL\_CONVERGENCE(GO:0060030) |  | 15 | -0.26 | -0.74 | 0.683 | 0.933 | 1.000 | 755 | tags=7%, list=2%, signal=7% |
| 1901 | NEURAL\_TUBE\_DEVELOPMENT(GO:0021915) |  | 105 | -0.14 | -0.73 | 0.853 | 0.935 | 1.000 | 8983 | tags=23%, list=28%, signal=32% |
| 1902 | CALCIUM\_ION\_TRANSMEMBRANE\_TRANSPORTER\_ACTIVITY(GO:0015085) |  | 126 | -0.17 | -0.73 | 0.926 | 0.935 | 1.000 | 5349 | tags=14%, list=17%, signal=17% |
| 1903 | REGULATION\_OF\_AMINE\_TRANSPORT(GO:0051952) |  | 29 | -0.24 | -0.73 | 0.815 | 0.935 | 1.000 | 8469 | tags=41%, list=27%, signal=56% |
| 1904 | HYDROLASE\_ACTIVITY\_\_HYDROLYZING\_N\_GLYCOSYL\_COMPOUNDS(GO:0016799) |  | 16 | -0.27 | -0.73 | 0.876 | 0.935 | 1.000 | 3211 | tags=19%, list=10%, signal=21% |
| 1905 | CATION\_CHANNEL\_ACTIVITY(GO:0005261) |  | 320 | -0.17 | -0.73 | 0.913 | 0.935 | 1.000 | 7990 | tags=26%, list=25%, signal=35% |
| 1906 | AXON\_TERMINUS(GO:0043679) |  | 28 | -0.27 | -0.73 | 0.767 | 0.936 | 1.000 | 9964 | tags=46%, list=31%, signal=67% |
| 1907 | NEURON\_PROJECTION\_GUIDANCE(GO:0097485) |  | 262 | -0.15 | -0.73 | 0.822 | 0.937 | 1.000 | 8353 | tags=23%, list=26%, signal=30% |
| 1908 | RNA\_POLYMERASE\_BINDING(GO:0070063) |  | 29 | -0.14 | -0.73 | 0.860 | 0.938 | 1.000 | 7074 | tags=21%, list=22%, signal=27% |
| 1909 | PHOTORECEPTOR\_OUTER\_SEGMENT(GO:0001750) |  | 33 | -0.29 | -0.73 | 0.762 | 0.938 | 1.000 | 4369 | tags=21%, list=14%, signal=25% |
| 1910 | OSTEOBLAST\_DIFFERENTIATION(GO:0001649) |  | 23 | -0.20 | -0.73 | 0.807 | 0.938 | 1.000 | 6029 | tags=22%, list=19%, signal=27% |
| 1911 | NEUROPEPTIDE\_RECEPTOR\_ACTIVITY(GO:0008188) |  | 37 | -0.24 | -0.73 | 0.865 | 0.938 | 1.000 | 5193 | tags=22%, list=16%, signal=26% |
| 1912 | REGULATION\_OF\_CELL\_GROWTH(GO:0001558) |  | 104 | -0.17 | -0.73 | 0.891 | 0.939 | 1.000 | 4400 | tags=12%, list=14%, signal=13% |
| 1913 | EAR\_DEVELOPMENT(GO:0043583) |  | 167 | -0.13 | -0.73 | 0.854 | 0.939 | 1.000 | 8983 | tags=24%, list=28%, signal=33% |
| 1914 | VOLTAGE\_GATED\_ION\_CHANNEL\_ACTIVITY(GO:0005244) |  | 192 | -0.17 | -0.73 | 0.953 | 0.939 | 1.000 | 5347 | tags=17%, list=17%, signal=20% |
| 1915 | ENDODEOXYRIBONUCLEASE\_ACTIVITY(GO:0004520) |  | 27 | -0.21 | -0.73 | 0.788 | 0.939 | 1.000 | 6354 | tags=26%, list=20%, signal=32% |
| 1916 | NEURON\_PROJECTION\_TERMINUS(GO:0044306) |  | 29 | -0.27 | -0.73 | 0.788 | 0.938 | 1.000 | 9964 | tags=45%, list=31%, signal=65% |
| 1917 | NEURON\_PROJECTION(GO:0043005) |  | 559 | -0.17 | -0.73 | 0.807 | 0.939 | 1.000 | 9056 | tags=30%, list=28%, signal=41% |
| 1918 | SYNAPSE(GO:0045202) |  | 523 | -0.17 | -0.72 | 0.853 | 0.939 | 1.000 | 9056 | tags=31%, list=28%, signal=43% |
| 1919 | PROTEIN\_AUTOPHOSPHORYLATION(GO:0046777) |  | 72 | -0.18 | -0.72 | 0.888 | 0.939 | 1.000 | 3714 | tags=10%, list=12%, signal=11% |
| 1920 | MICROTUBULE\_MOTOR\_ACTIVITY(GO:0003777) |  | 62 | -0.16 | -0.72 | 0.760 | 0.940 | 1.000 | 9553 | tags=29%, list=30%, signal=41% |
| 1921 | MONOAMINE\_TRANSPORT(GO:0015844) |  | 32 | -0.23 | -0.72 | 0.786 | 0.940 | 1.000 | 8469 | tags=38%, list=27%, signal=51% |
| 1922 | PHOTORECEPTOR\_CELL\_DEVELOPMENT(GO:0042461) |  | 70 | -0.20 | -0.72 | 0.780 | 0.943 | 1.000 | 7486 | tags=24%, list=23%, signal=32% |
| 1923 | NEURON\_PART(GO:0097458) |  | 758 | -0.16 | -0.72 | 0.864 | 0.942 | 1.000 | 9056 | tags=30%, list=28%, signal=40% |
| 1924 | HEART\_JOGGING(GO:0003146) |  | 42 | -0.20 | -0.72 | 0.842 | 0.942 | 1.000 | 9659 | tags=36%, list=30%, signal=51% |
| 1925 | AMINE\_TRANSPORT(GO:0015837) |  | 30 | -0.23 | -0.72 | 0.836 | 0.942 | 1.000 | 8469 | tags=40%, list=27%, signal=54% |
| 1926 | HAIR\_CELL\_DIFFERENTIATION(GO:0035315) |  | 57 | -0.16 | -0.72 | 0.845 | 0.942 | 1.000 | 1842 | tags=4%, list=6%, signal=4% |
| 1927 | ENDOCRINE\_SYSTEM\_DEVELOPMENT(GO:0035270) |  | 115 | -0.16 | -0.72 | 0.854 | 0.942 | 1.000 | 5764 | tags=14%, list=18%, signal=17% |
| 1928 | NUCLEOTIDE\_CATABOLIC\_PROCESS(GO:0009166) |  | 71 | -0.20 | -0.72 | 0.804 | 0.942 | 1.000 | 8735 | tags=31%, list=27%, signal=43% |
| 1929 | REGULATION\_OF\_PROTEIN\_LOCALIZATION\_TO\_MEMBRANE(GO:1905475) |  | 28 | -0.19 | -0.72 | 0.898 | 0.942 | 1.000 | 9792 | tags=43%, list=31%, signal=62% |
| 1930 | RENAL\_SYSTEM\_PROCESS(GO:0003014) |  | 38 | -0.18 | -0.72 | 0.904 | 0.943 | 1.000 | 10505 | tags=37%, list=33%, signal=55% |
| 1931 | INTRAHEPATIC\_BILE\_DUCT\_DEVELOPMENT(GO:0035622) |  | 16 | -0.22 | -0.71 | 0.768 | 0.944 | 1.000 | 7903 | tags=31%, list=25%, signal=42% |
| 1932 | G\_PROTEIN\_COUPLED\_NEUROTRANSMITTER\_RECEPTOR\_ACTIVITY(GO:0099528) |  | 48 | -0.24 | -0.71 | 0.887 | 0.943 | 1.000 | 6418 | tags=27%, list=20%, signal=34% |
| 1933 | SM\_LIKE\_PROTEIN\_FAMILY\_COMPLEX(GO:0120114) |  | 52 | -0.17 | -0.71 | 0.764 | 0.944 | 1.000 | 5565 | tags=10%, list=17%, signal=12% |
| 1934 | NODAL\_SIGNALING\_PATHWAY(GO:0038092) |  | 17 | -0.21 | -0.71 | 0.865 | 0.944 | 1.000 | 5377 | tags=18%, list=17%, signal=21% |
| 1935 | MITOCHONDRIAL\_ELECTRON\_TRANSPORT\_\_NADH\_TO\_UBIQUINONE(GO:0006120) |  | 21 | -0.21 | -0.71 | 0.776 | 0.944 | 1.000 | 10721 | tags=33%, list=34%, signal=50% |
| 1936 | OTIC\_PLACODE\_FORMATION(GO:0043049) |  | 21 | -0.22 | -0.71 | 0.837 | 0.944 | 1.000 | 10821 | tags=48%, list=34%, signal=72% |
| 1937 | REGULATION\_OF\_HEART\_MORPHOGENESIS(GO:2000826) |  | 19 | -0.23 | -0.71 | 0.832 | 0.944 | 1.000 | 5996 | tags=21%, list=19%, signal=26% |
| 1938 | PROTEIN\_STABILIZATION(GO:0050821) |  | 23 | -0.18 | -0.71 | 0.833 | 0.944 | 1.000 | 6343 | tags=17%, list=20%, signal=22% |
| 1939 | GLYCEROLIPID\_CATABOLIC\_PROCESS(GO:0046503) |  | 29 | -0.24 | -0.71 | 0.786 | 0.943 | 1.000 | 5968 | tags=21%, list=19%, signal=25% |
| 1940 | UDP\_GLYCOSYLTRANSFERASE\_ACTIVITY(GO:0008194) |  | 139 | -0.19 | -0.71 | 0.811 | 0.944 | 1.000 | 5776 | tags=18%, list=18%, signal=22% |
| 1941 | SYNAPTIC\_SIGNALING(GO:0099536) |  | 360 | -0.18 | -0.71 | 0.853 | 0.944 | 1.000 | 9517 | tags=35%, list=30%, signal=50% |
| 1942 | MAP\_KINASE\_KINASE\_KINASE\_ACTIVITY(GO:0004709) |  | 18 | -0.22 | -0.71 | 0.771 | 0.946 | 1.000 | 9182 | tags=39%, list=29%, signal=55% |
| 1943 | CELL\_MIGRATION\_TO\_THE\_MIDLINE\_INVOLVED\_IN\_HEART\_DEVELOPMENT(GO:0003318) |  | 19 | -0.21 | -0.71 | 0.859 | 0.946 | 1.000 | 25274 | tags=100%, list=79%, signal=482% |
| 1944 | GLYCEROPHOSPHOLIPID\_CATABOLIC\_PROCESS(GO:0046475) |  | 15 | -0.30 | -0.70 | 0.716 | 0.951 | 1.000 | 7756 | tags=33%, list=24%, signal=44% |
| 1945 | MECHANORECEPTOR\_DIFFERENTIATION(GO:0042490) |  | 62 | -0.15 | -0.70 | 0.853 | 0.951 | 1.000 | 1842 | tags=3%, list=6%, signal=3% |
| 1946 | RUFFLE\_MEMBRANE(GO:0032587) |  | 29 | -0.18 | -0.70 | 0.889 | 0.952 | 1.000 | 4083 | tags=14%, list=13%, signal=16% |
| 1947 | GLOMERULAR\_VISCERAL\_EPITHELIAL\_CELL\_DEVELOPMENT(GO:0072015) |  | 25 | -0.18 | -0.70 | 0.890 | 0.953 | 1.000 | 3956 | tags=16%, list=12%, signal=18% |
| 1948 | GLOMERULAR\_EPITHELIAL\_CELL\_DEVELOPMENT(GO:0072310) |  | 25 | -0.18 | -0.70 | 0.890 | 0.952 | 1.000 | 3956 | tags=16%, list=12%, signal=18% |
| 1949 | LIPID\_TRANSLOCATION(GO:0034204) |  | 18 | -0.24 | -0.70 | 0.808 | 0.952 | 1.000 | 1894 | tags=11%, list=6%, signal=12% |
| 1950 | TRANS\_SYNAPTIC\_SIGNALING(GO:0099537) |  | 352 | -0.18 | -0.70 | 0.850 | 0.953 | 1.000 | 9507 | tags=35%, list=30%, signal=50% |
| 1951 | PHOSPHOLIPID\_TRANSLOCATION(GO:0045332) |  | 17 | -0.25 | -0.70 | 0.869 | 0.954 | 1.000 | 1894 | tags=12%, list=6%, signal=13% |
| 1952 | CELL\_FATE\_SPECIFICATION(GO:0001708) |  | 107 | -0.17 | -0.69 | 0.953 | 0.954 | 1.000 | 8266 | tags=26%, list=26%, signal=35% |
| 1953 | PYRIMIDINE\_NUCLEOSIDE\_TRIPHOSPHATE\_BIOSYNTHETIC\_PROCESS(GO:0009148) |  | 20 | -0.23 | -0.69 | 0.841 | 0.954 | 1.000 | 8837 | tags=40%, list=28%, signal=55% |
| 1954 | VOLTAGE\_GATED\_CALCIUM\_CHANNEL\_COMPLEX(GO:0005891) |  | 37 | -0.20 | -0.69 | 0.803 | 0.954 | 1.000 | 5032 | tags=16%, list=16%, signal=19% |
| 1955 | NEGATIVE\_REGULATION\_OF\_RNA\_METABOLIC\_PROCESS(GO:0051253) |  | 350 | -0.12 | -0.69 | 0.953 | 0.958 | 1.000 | 6367 | tags=14%, list=20%, signal=17% |
| 1956 | CYTOPLASMIC\_MICROTUBULE(GO:0005881) |  | 27 | -0.18 | -0.69 | 0.887 | 0.958 | 1.000 | 9222 | tags=33%, list=29%, signal=47% |
| 1957 | HISTONE\_DEACETYLATION(GO:0016575) |  | 37 | -0.17 | -0.69 | 0.894 | 0.958 | 1.000 | 2931 | tags=8%, list=9%, signal=9% |
| 1958 | RNA\_PHOSPHODIESTER\_BOND\_HYDROLYSIS(GO:0090501) |  | 47 | -0.19 | -0.69 | 0.820 | 0.958 | 1.000 | 10421 | tags=49%, list=33%, signal=73% |
| 1959 | POSITIVE\_REGULATION\_OF\_PROTEIN\_SERINE\_THREONINE\_KINASE\_ACTIVITY(GO:0071902) |  | 26 | -0.20 | -0.69 | 0.882 | 0.958 | 1.000 | 2376 | tags=12%, list=7%, signal=12% |
| 1960 | AXON(GO:0030424) |  | 185 | -0.17 | -0.69 | 0.851 | 0.958 | 1.000 | 9473 | tags=35%, list=30%, signal=49% |
| 1961 | NUCLEOSIDE\_PHOSPHATE\_CATABOLIC\_PROCESS(GO:1901292) |  | 83 | -0.18 | -0.69 | 0.915 | 0.958 | 1.000 | 4213 | tags=13%, list=13%, signal=15% |
| 1962 | 3'\_5'\_CYCLIC\_NUCLEOTIDE\_PHOSPHODIESTERASE\_ACTIVITY(GO:0004114) |  | 27 | -0.22 | -0.69 | 0.807 | 0.957 | 1.000 | 10713 | tags=37%, list=34%, signal=56% |
| 1963 | PHOSPHOLIPID\_CATABOLIC\_PROCESS(GO:0009395) |  | 29 | -0.24 | -0.68 | 0.838 | 0.957 | 1.000 | 7756 | tags=24%, list=24%, signal=32% |
| 1964 | PROTEIN\_DEUBIQUITINATION(GO:0016579) |  | 88 | -0.13 | -0.68 | 0.888 | 0.959 | 1.000 | 9526 | tags=31%, list=30%, signal=44% |
| 1965 | RENAL\_FILTRATION(GO:0097205) |  | 28 | -0.17 | -0.68 | 0.888 | 0.961 | 1.000 | 26429 | tags=100%, list=83%, signal=583% |
| 1966 | ESTABLISHMENT\_OF\_PLANAR\_POLARITY(GO:0001736) |  | 19 | -0.24 | -0.68 | 0.785 | 0.960 | 1.000 | 24313 | tags=100%, list=76%, signal=421% |
| 1967 | ESTABLISHMENT\_OF\_TISSUE\_POLARITY(GO:0007164) |  | 19 | -0.24 | -0.68 | 0.785 | 0.960 | 1.000 | 24313 | tags=100%, list=76%, signal=421% |
| 1968 | MONOVALENT\_INORGANIC\_CATION\_HOMEOSTASIS(GO:0055067) |  | 76 | -0.15 | -0.68 | 0.882 | 0.962 | 1.000 | 2201 | tags=8%, list=7%, signal=8% |
| 1969 | THIOL\_DEPENDENT\_UBIQUITIN\_SPECIFIC\_PROTEASE\_ACTIVITY(GO:0004843) |  | 84 | -0.14 | -0.68 | 0.905 | 0.963 | 1.000 | 9526 | tags=32%, list=30%, signal=46% |
| 1970 | THIOL\_DEPENDENT\_UBIQUITINYL\_HYDROLASE\_ACTIVITY(GO:0036459) |  | 84 | -0.14 | -0.68 | 0.905 | 0.962 | 1.000 | 9526 | tags=32%, list=30%, signal=46% |
| 1971 | UBIQUITINYL\_HYDROLASE\_ACTIVITY(GO:0101005) |  | 84 | -0.14 | -0.68 | 0.905 | 0.962 | 1.000 | 9526 | tags=32%, list=30%, signal=46% |
| 1972 | NUCLEOBASE\_CONTAINING\_COMPOUND\_KINASE\_ACTIVITY(GO:0019205) |  | 39 | -0.20 | -0.67 | 0.834 | 0.962 | 1.000 | 8814 | tags=28%, list=28%, signal=39% |
| 1973 | SYNAPSE\_PART(GO:0044456) |  | 421 | -0.16 | -0.67 | 0.853 | 0.962 | 1.000 | 9056 | tags=31%, list=28%, signal=43% |
| 1974 | CHEMICAL\_SYNAPTIC\_TRANSMISSION(GO:0007268) |  | 349 | -0.18 | -0.67 | 0.886 | 0.962 | 1.000 | 9507 | tags=35%, list=30%, signal=49% |
| 1975 | ANTEROGRADE\_TRANS\_SYNAPTIC\_SIGNALING(GO:0098916) |  | 349 | -0.18 | -0.67 | 0.886 | 0.961 | 1.000 | 9507 | tags=35%, list=30%, signal=49% |
| 1976 | CATECHOLAMINE\_TRANSPORT(GO:0051937) |  | 28 | -0.22 | -0.67 | 0.848 | 0.963 | 1.000 | 8469 | tags=39%, list=27%, signal=53% |
| 1977 | PHOTORECEPTOR\_CELL\_CILIUM(GO:0097733) |  | 42 | -0.26 | -0.67 | 0.746 | 0.962 | 1.000 | 4590 | tags=19%, list=14%, signal=22% |
| 1978 | TERMINAL\_BOUTON(GO:0043195) |  | 17 | -0.24 | -0.67 | 0.888 | 0.962 | 1.000 | 7931 | tags=35%, list=25%, signal=47% |
| 1979 | VOLTAGE\_GATED\_CATION\_CHANNEL\_ACTIVITY(GO:0022843) |  | 157 | -0.17 | -0.67 | 0.938 | 0.962 | 1.000 | 5347 | tags=17%, list=17%, signal=20% |
| 1980 | CELLULAR\_RESPONSE\_TO\_CALCIUM\_ION(GO:0071277) |  | 34 | -0.23 | -0.67 | 0.767 | 0.962 | 1.000 | 9283 | tags=41%, list=29%, signal=58% |
| 1981 | VISUAL\_BEHAVIOR(GO:0007632) |  | 24 | -0.20 | -0.67 | 0.934 | 0.963 | 1.000 | 5768 | tags=21%, list=18%, signal=25% |
| 1982 | REGULATION\_OF\_SIGNALING\_RECEPTOR\_ACTIVITY(GO:0010469) |  | 43 | -0.19 | -0.67 | 0.889 | 0.964 | 1.000 | 7645 | tags=26%, list=24%, signal=34% |
| 1983 | AMPA\_GLUTAMATE\_RECEPTOR\_COMPLEX(GO:0032281) |  | 25 | -0.23 | -0.67 | 0.832 | 0.963 | 1.000 | 5347 | tags=24%, list=17%, signal=29% |
| 1984 | PHOSPHATIDYLINOSITOL\_3\_KINASE\_SIGNALING(GO:0014065) |  | 33 | -0.22 | -0.67 | 0.921 | 0.964 | 1.000 | 9541 | tags=42%, list=30%, signal=60% |
| 1985 | CADHERIN\_BINDING(GO:0045296) |  | 37 | -0.16 | -0.66 | 0.880 | 0.967 | 1.000 | 8983 | tags=30%, list=28%, signal=41% |
| 1986 | PYRIMIDINE\_CONTAINING\_COMPOUND\_BIOSYNTHETIC\_PROCESS(GO:0072528) |  | 31 | -0.19 | -0.66 | 0.957 | 0.967 | 1.000 | 7211 | tags=26%, list=23%, signal=33% |
| 1987 | SMAD\_BINDING(GO:0046332) |  | 36 | -0.18 | -0.66 | 0.824 | 0.968 | 1.000 | 5377 | tags=11%, list=17%, signal=13% |
| 1988 | NEGATIVE\_REGULATION\_OF\_TRANSMEMBRANE\_TRANSPORT(GO:0034763) |  | 18 | -0.23 | -0.66 | 0.885 | 0.968 | 1.000 | 5664 | tags=22%, list=18%, signal=27% |
| 1989 | MITOCHONDRIAL\_RESPIRATORY\_CHAIN\_COMPLEX\_I\_ASSEMBLY(GO:0032981) |  | 21 | -0.22 | -0.66 | 0.797 | 0.969 | 1.000 | 8562 | tags=29%, list=27%, signal=39% |
| 1990 | NADH\_DEHYDROGENASE\_COMPLEX\_ASSEMBLY(GO:0010257) |  | 21 | -0.22 | -0.66 | 0.797 | 0.969 | 1.000 | 8562 | tags=29%, list=27%, signal=39% |
| 1991 | ANION:CATION\_SYMPORTER\_ACTIVITY(GO:0015296) |  | 18 | -0.25 | -0.66 | 0.921 | 0.968 | 1.000 | 9907 | tags=50%, list=31%, signal=72% |
| 1992 | STEROID\_HORMONE\_RECEPTOR\_ACTIVITY(GO:0003707) |  | 26 | -0.20 | -0.66 | 0.940 | 0.968 | 1.000 | 5197 | tags=15%, list=16%, signal=18% |
| 1993 | AMP\_METABOLIC\_PROCESS(GO:0046033) |  | 18 | -0.17 | -0.66 | 0.880 | 0.968 | 1.000 | 3137 | tags=11%, list=10%, signal=12% |
| 1994 | GLUCOSE\_6\_PHOSPHATE\_METABOLIC\_PROCESS(GO:0051156) |  | 18 | -0.22 | -0.66 | 0.913 | 0.967 | 1.000 | 4973 | tags=17%, list=16%, signal=20% |
| 1995 | INTRAMOLECULAR\_TRANSFERASE\_ACTIVITY(GO:0016866) |  | 24 | -0.19 | -0.65 | 0.917 | 0.969 | 1.000 | 9571 | tags=42%, list=30%, signal=59% |
| 1996 | TUMOR\_NECROSIS\_FACTOR\_RECEPTOR\_SUPERFAMILY\_BINDING(GO:0032813) |  | 30 | -0.22 | -0.65 | 0.860 | 0.969 | 1.000 | 1364 | tags=7%, list=4%, signal=7% |
| 1997 | U12\_TYPE\_SPLICEOSOMAL\_COMPLEX(GO:0005689) |  | 17 | -0.23 | -0.65 | 0.787 | 0.968 | 1.000 | 4961 | tags=18%, list=16%, signal=21% |
| 1998 | REGULATION\_OF\_PHOSPHATIDYLINOSITOL\_3\_KINASE\_SIGNALING(GO:0014066) |  | 18 | -0.26 | -0.65 | 0.811 | 0.969 | 1.000 | 9541 | tags=44%, list=30%, signal=63% |
| 1999 | MODULATION\_OF\_CHEMICAL\_SYNAPTIC\_TRANSMISSION(GO:0050804) |  | 146 | -0.17 | -0.65 | 0.936 | 0.969 | 1.000 | 9031 | tags=32%, list=28%, signal=44% |
| 2000 | REGULATION\_OF\_TRANS\_SYNAPTIC\_SIGNALING(GO:0099177) |  | 146 | -0.17 | -0.65 | 0.936 | 0.969 | 1.000 | 9031 | tags=32%, list=28%, signal=44% |
| 2001 | TRANSMEMBRANE\_RECEPTOR\_PROTEIN\_SERINE\_THREONINE\_KINASE\_ACTIVITY(GO:0004675) |  | 20 | -0.17 | -0.65 | 0.880 | 0.969 | 1.000 | 8310 | tags=30%, list=26%, signal=41% |
| 2002 | REGULATION\_OF\_COAGULATION(GO:0050818) |  | 19 | -0.21 | -0.65 | 0.957 | 0.970 | 1.000 | 7942 | tags=32%, list=25%, signal=42% |
| 2003 | 9+0\_NON\_MOTILE\_CILIUM(GO:0097731) |  | 43 | -0.25 | -0.65 | 0.797 | 0.970 | 1.000 | 4590 | tags=19%, list=14%, signal=22% |
| 2004 | DAMAGED\_DNA\_BINDING(GO:0003684) |  | 31 | -0.17 | -0.64 | 0.954 | 0.973 | 1.000 | 9214 | tags=32%, list=29%, signal=45% |
| 2005 | 5'\_3'\_EXONUCLEASE\_ACTIVITY(GO:0008409) |  | 15 | -0.20 | -0.64 | 0.799 | 0.973 | 1.000 | 25507 | tags=100%, list=80%, signal=499% |
| 2006 | REGULATION\_OF\_MEMBRANE\_POTENTIAL(GO:0042391) |  | 155 | -0.16 | -0.64 | 0.977 | 0.972 | 1.000 | 4184 | tags=12%, list=13%, signal=14% |
| 2007 | NEGATIVE\_REGULATION\_OF\_PROTEIN\_CATABOLIC\_PROCESS(GO:0042177) |  | 16 | -0.15 | -0.64 | 0.910 | 0.972 | 1.000 | 2392 | tags=6%, list=8%, signal=7% |
| 2008 | G\_PROTEIN\_COUPLED\_AMINE\_RECEPTOR\_ACTIVITY(GO:0008227) |  | 77 | -0.19 | -0.64 | 0.977 | 0.972 | 1.000 | 7533 | tags=27%, list=24%, signal=36% |
| 2009 | EPITHELIAL\_CELL\_DIFFERENTIATION\_INVOLVED\_IN\_KIDNEY\_DEVELOPMENT(GO:0035850) |  | 40 | -0.18 | -0.64 | 0.842 | 0.971 | 1.000 | 10981 | tags=43%, list=34%, signal=65% |
| 2010 | PROTON\_TRANSPORTING\_ATP\_SYNTHASE\_COMPLEX(GO:0045259) |  | 23 | -0.22 | -0.64 | 0.889 | 0.971 | 1.000 | 11107 | tags=52%, list=35%, signal=80% |
| 2011 | MITOCHONDRIAL\_PROTON\_TRANSPORTING\_ATP\_SYNTHASE\_COMPLEX(GO:0005753) |  | 23 | -0.22 | -0.64 | 0.889 | 0.971 | 1.000 | 11107 | tags=52%, list=35%, signal=80% |
| 2012 | NERVOUS\_SYSTEM\_PROCESS(GO:0050877) |  | 423 | -0.15 | -0.64 | 0.942 | 0.971 | 1.000 | 6248 | tags=16%, list=20%, signal=20% |
| 2013 | OTOLITH\_MORPHOGENESIS(GO:0032474) |  | 21 | -0.19 | -0.64 | 0.947 | 0.971 | 1.000 | 25897 | tags=100%, list=81%, signal=532% |
| 2014 | MITOCHONDRIAL\_GENE\_EXPRESSION(GO:0140053) |  | 53 | -0.16 | -0.63 | 0.850 | 0.972 | 1.000 | 4331 | tags=9%, list=14%, signal=11% |
| 2015 | LEFT\_RIGHT\_PATTERN\_FORMATION(GO:0060972) |  | 28 | -0.20 | -0.63 | 0.946 | 0.974 | 1.000 | 5338 | tags=21%, list=17%, signal=26% |
| 2016 | ORGANOPHOSPHATE\_CATABOLIC\_PROCESS(GO:0046434) |  | 129 | -0.15 | -0.63 | 0.922 | 0.975 | 1.000 | 4230 | tags=12%, list=13%, signal=14% |
| 2017 | LYSOSOME\_ORGANIZATION(GO:0007040) |  | 26 | -0.17 | -0.63 | 0.977 | 0.975 | 1.000 | 11673 | tags=35%, list=37%, signal=55% |
| 2018 | LYTIC\_VACUOLE\_ORGANIZATION(GO:0080171) |  | 26 | -0.17 | -0.63 | 0.977 | 0.975 | 1.000 | 11673 | tags=35%, list=37%, signal=55% |
| 2019 | POSITIVE\_REGULATION\_OF\_BMP\_SIGNALING\_PATHWAY(GO:0030513) |  | 20 | -0.18 | -0.62 | 0.952 | 0.977 | 1.000 | 1117 | tags=5%, list=4%, signal=5% |
| 2020 | RENAL\_FILTRATION\_CELL\_DIFFERENTIATION(GO:0061318) |  | 30 | -0.17 | -0.62 | 0.871 | 0.977 | 1.000 | 11811 | tags=50%, list=37%, signal=79% |
| 2021 | GLOMERULAR\_EPITHELIUM\_DEVELOPMENT(GO:0072010) |  | 30 | -0.17 | -0.62 | 0.871 | 0.976 | 1.000 | 11811 | tags=50%, list=37%, signal=79% |
| 2022 | GLOMERULAR\_VISCERAL\_EPITHELIAL\_CELL\_DIFFERENTIATION(GO:0072112) |  | 30 | -0.17 | -0.62 | 0.871 | 0.976 | 1.000 | 11811 | tags=50%, list=37%, signal=79% |
| 2023 | GLOMERULAR\_EPITHELIAL\_CELL\_DIFFERENTIATION(GO:0072311) |  | 30 | -0.17 | -0.62 | 0.871 | 0.975 | 1.000 | 11811 | tags=50%, list=37%, signal=79% |
| 2024 | NUCLEOSOME\_ASSEMBLY(GO:0006334) |  | 59 | -0.17 | -0.62 | 0.959 | 0.978 | 1.000 | 3703 | tags=12%, list=12%, signal=13% |
| 2025 | POSTSYNAPSE(GO:0098794) |  | 252 | -0.16 | -0.62 | 0.952 | 0.978 | 1.000 | 9056 | tags=32%, list=28%, signal=44% |
| 2026 | VASCULAR\_PROCESS\_IN\_CIRCULATORY\_SYSTEM(GO:0003018) |  | 63 | -0.16 | -0.61 | 0.976 | 0.979 | 1.000 | 6334 | tags=17%, list=20%, signal=22% |
| 2027 | PRONEPHRIC\_NEPHRON\_TUBULE\_DEVELOPMENT(GO:0039020) |  | 15 | -0.23 | -0.61 | 0.891 | 0.979 | 1.000 | 9242 | tags=40%, list=29%, signal=56% |
| 2028 | HISTONE\_H4\_ACETYLATION(GO:0043967) |  | 15 | -0.20 | -0.61 | 0.922 | 0.979 | 1.000 | 25497 | tags=100%, list=80%, signal=499% |
| 2029 | AXONEMAL\_DYNEIN\_COMPLEX\_ASSEMBLY(GO:0070286) |  | 24 | -0.23 | -0.61 | 0.870 | 0.979 | 1.000 | 9467 | tags=42%, list=30%, signal=59% |
| 2030 | REGULATION\_OF\_POSTSYNAPSE\_ORGANIZATION(GO:0099175) |  | 15 | -0.21 | -0.61 | 0.909 | 0.979 | 1.000 | 9083 | tags=33%, list=28%, signal=47% |
| 2031 | REGULATION\_OF\_EPITHELIAL\_CELL\_DIFFERENTIATION(GO:0030856) |  | 17 | -0.21 | -0.61 | 0.920 | 0.979 | 1.000 | 6039 | tags=29%, list=19%, signal=36% |
| 2032 | NON\_MOTILE\_CILIUM(GO:0097730) |  | 56 | -0.20 | -0.61 | 0.928 | 0.980 | 1.000 | 5031 | tags=16%, list=16%, signal=19% |
| 2033 | CRANIAL\_NERVE\_MORPHOGENESIS(GO:0021602) |  | 21 | -0.16 | -0.60 | 0.892 | 0.980 | 1.000 | 9443 | tags=33%, list=30%, signal=47% |
| 2034 | TRANSMISSION\_OF\_NERVE\_IMPULSE(GO:0019226) |  | 27 | -0.20 | -0.60 | 0.924 | 0.982 | 1.000 | 7645 | tags=33%, list=24%, signal=44% |
| 2035 | RESPONSE\_TO\_ACTIVITY(GO:0014823) |  | 16 | -0.19 | -0.59 | 0.925 | 0.984 | 1.000 | 1312 | tags=6%, list=4%, signal=7% |
| 2036 | POSITIVE\_REGULATION\_OF\_TRANSMEMBRANE\_RECEPTOR\_PROTEIN\_SERINE\_THREONINE\_KINASE\_SIGNALING\_PATHWAY(GO:0090100) |  | 52 | -0.14 | -0.59 | 0.950 | 0.985 | 1.000 | 8447 | tags=25%, list=26%, signal=34% |
| 2037 | RAC\_PROTEIN\_SIGNAL\_TRANSDUCTION(GO:0016601) |  | 15 | -0.21 | -0.59 | 0.811 | 0.985 | 1.000 | 11511 | tags=40%, list=36%, signal=63% |
| 2038 | LEUKOCYTE\_ACTIVATION\_INVOLVED\_IN\_IMMUNE\_RESPONSE(GO:0002366) |  | 35 | -0.16 | -0.59 | 0.979 | 0.985 | 1.000 | 10383 | tags=40%, list=33%, signal=59% |
| 2039 | SARCOPLASM(GO:0016528) |  | 30 | -0.16 | -0.58 | 0.909 | 0.986 | 1.000 | 9772 | tags=37%, list=31%, signal=53% |
| 2040 | VOLTAGE\_GATED\_CALCIUM\_CHANNEL\_ACTIVITY(GO:0005245) |  | 47 | -0.17 | -0.58 | 0.922 | 0.987 | 1.000 | 5347 | tags=15%, list=17%, signal=18% |
| 2041 | LENS\_DEVELOPMENT\_IN\_CAMERA\_TYPE\_EYE(GO:0002088) |  | 99 | -0.19 | -0.58 | 0.979 | 0.987 | 1.000 | 11023 | tags=38%, list=35%, signal=58% |
| 2042 | POSITIVE\_REGULATION\_OF\_PHOSPHATIDYLINOSITOL\_3\_KINASE\_SIGNALING(GO:0014068) |  | 16 | -0.25 | -0.58 | 0.831 | 0.987 | 1.000 | 8786 | tags=38%, list=28%, signal=52% |
| 2043 | PROTEIN\_TRANSMEMBRANE\_IMPORT\_INTO\_INTRACELLULAR\_ORGANELLE(GO:0044743) |  | 35 | -0.18 | -0.58 | 0.873 | 0.987 | 1.000 | 6343 | tags=20%, list=20%, signal=25% |
| 2044 | KINESIN\_COMPLEX(GO:0005871) |  | 52 | -0.12 | -0.58 | 0.979 | 0.987 | 1.000 | 10048 | tags=35%, list=32%, signal=50% |
| 2045 | IONOTROPIC\_GLUTAMATE\_RECEPTOR\_COMPLEX(GO:0008328) |  | 35 | -0.18 | -0.58 | 0.915 | 0.987 | 1.000 | 6156 | tags=20%, list=19%, signal=25% |
| 2046 | NEUROTRANSMITTER\_RECEPTOR\_COMPLEX(GO:0098878) |  | 35 | -0.18 | -0.58 | 0.915 | 0.986 | 1.000 | 6156 | tags=20%, list=19%, signal=25% |
| 2047 | SECONDARY\_METABOLIC\_PROCESS(GO:0019748) |  | 17 | -0.20 | -0.57 | 0.864 | 0.986 | 1.000 | 9412 | tags=41%, list=30%, signal=58% |
| 2048 | MITOCHONDRIAL\_TRANSLATION(GO:0032543) |  | 35 | -0.15 | -0.57 | 0.921 | 0.986 | 1.000 | 9814 | tags=31%, list=31%, signal=45% |
| 2049 | INNER\_EAR\_RECEPTOR\_CELL\_DEVELOPMENT(GO:0060119) |  | 34 | -0.14 | -0.57 | 0.951 | 0.986 | 1.000 | 11695 | tags=47%, list=37%, signal=74% |
| 2050 | CELL\_CELL\_ADHESION\_MEDIATOR\_ACTIVITY(GO:0098632) |  | 17 | -0.17 | -0.57 | 0.914 | 0.986 | 1.000 | 26472 | tags=100%, list=83%, signal=588% |
| 2051 | REGULATION\_OF\_LIPID\_LOCALIZATION(GO:1905952) |  | 20 | -0.18 | -0.57 | 0.920 | 0.987 | 1.000 | 26057 | tags=100%, list=82%, signal=546% |
| 2052 | NEUROTRANSMITTER\_RECEPTOR\_ACTIVITY(GO:0030594) |  | 139 | -0.17 | -0.56 | 0.977 | 0.987 | 1.000 | 5808 | tags=19%, list=18%, signal=23% |
| 2053 | PRENYLTRANSFERASE\_ACTIVITY(GO:0004659) |  | 18 | -0.18 | -0.55 | 0.942 | 0.991 | 1.000 | 10216 | tags=39%, list=32%, signal=57% |
| 2054 | POSITIVE\_REGULATION\_OF\_CHROMATIN\_ORGANIZATION(GO:1905269) |  | 17 | -0.18 | -0.54 | 0.978 | 0.992 | 1.000 | 9717 | tags=29%, list=30%, signal=42% |
| 2055 | RESPONSE\_TO\_DSRNA(GO:0043331) |  | 27 | -0.15 | -0.53 | 0.978 | 0.995 | 1.000 | 2792 | tags=7%, list=9%, signal=8% |
| 2056 | REGULATION\_OF\_GENE\_SILENCING(GO:0060968) |  | 19 | -0.19 | -0.52 | 0.951 | 0.995 | 1.000 | 25808 | tags=100%, list=81%, signal=524% |
| 2057 | POTASSIUM\_ION\_LEAK\_CHANNEL\_ACTIVITY(GO:0022841) |  | 20 | -0.19 | -0.52 | 0.927 | 0.995 | 1.000 | 8508 | tags=30%, list=27%, signal=41% |
| 2058 | LEAK\_CHANNEL\_ACTIVITY(GO:0022840) |  | 20 | -0.19 | -0.52 | 0.927 | 0.994 | 1.000 | 8508 | tags=30%, list=27%, signal=41% |
| 2059 | NARROW\_PORE\_CHANNEL\_ACTIVITY(GO:0022842) |  | 20 | -0.19 | -0.52 | 0.927 | 0.994 | 1.000 | 8508 | tags=30%, list=27%, signal=41% |
| 2060 | PROTON\_TRANSPORTING\_ATP\_SYNTHASE\_COMPLEX\_\_COUPLING\_FACTOR\_F(O)(GO:0045263) |  | 17 | -0.18 | -0.52 | 0.886 | 0.994 | 1.000 | 11084 | tags=47%, list=35%, signal=72% |
| 2061 | POSITIVE\_REGULATION\_OF\_SYNAPTIC\_TRANSMISSION(GO:0050806) |  | 33 | -0.18 | -0.52 | 0.977 | 0.994 | 1.000 | 9672 | tags=33%, list=30%, signal=48% |
| 2062 | PATHWAY\_RESTRICTED\_SMAD\_PROTEIN\_PHOSPHORYLATION(GO:0060389) |  | 39 | -0.14 | -0.51 | 0.977 | 0.995 | 1.000 | 8447 | tags=23%, list=26%, signal=31% |
| 2063 | CALMODULIN\_DEPENDENT\_PROTEIN\_KINASE\_ACTIVITY(GO:0004683) |  | 28 | -0.15 | -0.51 | 0.978 | 0.994 | 1.000 | 6012 | tags=21%, list=19%, signal=26% |
| 2064 | TRACE\_AMINE\_RECEPTOR\_ACTIVITY(GO:0001594) |  | 21 | -0.20 | -0.50 | 0.979 | 0.995 | 1.000 | 8398 | tags=33%, list=26%, signal=45% |
| 2065 | GLUTAMATE\_RECEPTOR\_ACTIVITY(GO:0008066) |  | 36 | -0.18 | -0.49 | 0.953 | 0.996 | 1.000 | 9325 | tags=33%, list=29%, signal=47% |
| 2066 | PROTEIN\_K48\_LINKED\_DEUBIQUITINATION(GO:0071108) |  | 17 | -0.15 | -0.47 | 0.978 | 0.998 | 1.000 | 27195 | tags=100%, list=85%, signal=679% |
| 2067 | SPINDLE\_MIDZONE(GO:0051233) |  | 21 | -0.15 | -0.47 | 0.979 | 0.998 | 1.000 | 27193 | tags=100%, list=85%, signal=678% |
| 2068 | VENTRICULAR\_SYSTEM\_DEVELOPMENT(GO:0021591) |  | 27 | -0.13 | -0.44 | 0.978 | 0.999 | 1.000 | 11996 | tags=44%, list=38%, signal=71% |
| 2069 | OLIGODENDROCYTE\_DEVELOPMENT(GO:0014003) |  | 28 | -0.12 | -0.42 | 0.977 | 1.000 | 1.000 | 10731 | tags=32%, list=34%, signal=48% |
| 2070 | STEM\_CELL\_POPULATION\_MAINTENANCE(GO:0019827) |  | 20 | -0.13 | -0.42 | 0.945 | 0.999 | 1.000 | 5764 | tags=15%, list=18%, signal=18% |
| 2071 | GABA\_RECEPTOR\_ACTIVITY(GO:0016917) |  | 25 | -0.15 | -0.40 | 0.978 | 0.999 | 1.000 | 5088 | tags=12%, list=16%, signal=14% |
| 2072 | METALLOAMINOPEPTIDASE\_ACTIVITY(GO:0070006) |  | 22 | -0.14 | -0.39 | 0.978 | 0.999 | 1.000 | 10242 | tags=36%, list=32%, signal=54% |
Table: Gene sets enriched in phenotype
 **WT (4 samples)
 **[plain text format]****
